# Identification and validation of DNA methylation-driven gene OSR1 as a novel tumor suppressor for the diagnosis and prognosis of breast cancer

**DOI:** 10.3389/fgene.2025.1583620

**Published:** 2025-07-07

**Authors:** Jian Xu, Biao Yang, Ling Cheng, Chen Gan, Runze Huang, Anlong Li, Han Ge, Longyu Hu, Meiwen Ling, Xinyi Zheng, Bao Zhao, Mingjun Zhang, Huaidong Cheng

**Affiliations:** ^1^ Department of Oncology, Second Affiliated Hospital of Anhui Medical University, Hefei, Anhui, China; ^2^ Institute of Health and Medicine, Hefei Comprehensive National Science Center, Hefei, Anhui, China; ^3^ Medical Intensive Care Unit, The First Affiliated Hospital of Anhui University of Chinese Medicine, Hefei, Anhui, China; ^4^ Department of Oncology, Shenzhen Hospital of Southern Medical University, Shenzhen, Guangdong, China

**Keywords:** breast cancer, DNA methylation, TCGA, OSR1, methylation-driven genes

## Abstract

**Introduction:**

Aberrant DNA methylation plays a critical role in the initiation and progression of cancer, yet its association with breast cancer remains inadequately defined. This study aims to clarify the link between methylation-driven genes and breast cancer pathogenesis.

**Methods:**

RNA sequencing and DNA methylation data for breast cancer were retrieved from The Cancer Genome Atlas (TCGA). Prognostically relevant methylation-driven genes were identified by integrating the methylation R package with univariate Cox regression analysis, and OSR1 emerged as the primary candidate. Gene expression profiles and corresponding clinical data were subsequently obtained from TCGA. Differential expression analysis using the Wilcoxon rank-sum test revealed significantly reduced OSR1 expression in breast cancer tissues compared to normal counterparts. Kaplan–Meier survival curves and Cox regression models were applied to assess the prognostic significance of OSR1. Bioinformatic analyses investigated associations between OSR1 expression and clinicopathological features, pathway enrichment, and immune cell infiltration. Experimental validation was conducted by generating OSR1-overexpressing breast cancer cell lines to examine effects on cell viability, migration, and proliferation *via* phenotypic assays.

**Results:**

OSR1 expression was significantly reduced in breast cancer tissues and correlated negatively with breast cancer progression. Low OSR1 expression was significantly associated with M stage, HER2 status, PAM50 subtypes, and histological classification, and linked to poorer overall survival outcomes. Functional enrichment implicated OSR1 in pathways related to peptide hormone secretion, peptide transport, metal ion response, and forebrain development. Elevated OSR1 expression was positively correlated with increased infiltration of NK cells, B cells, CD8^+^ T cells, and dendritic cells. Both *in vitro* and *in vivo* studies demonstrated that OSR1 overexpression markedly suppressed breast cancer cell proliferation and migration.

**Discussion:**

These findings confirm OSR1 as a methylation-regulated tumor suppressor gene and underscore its potential as a promising biomarker for individualized therapeutic strategies in breast cancer.

## Introduction

In 2022, approximately 2.3 million new cases of breast cancer were diagnosed globally, representing 11.6% of all cancer diagnoses and ranking as the second-most prevalent malignancy and the fourth leading cause of cancer-related mortality worldwide (Bray et al., 2024). Notable advancements in 5-year survival rates have been achieved over recent decades, attributed to the widespread implementation of early detection programs, improvements in therapeutic modalities, and shifts in demographic and socioeconomic factors. Despite these gains, emerging evidence highlights a persistent risk of recurrence extending beyond a decade following initial diagnosis (Fillon, 2022), underscoring the ongoing need for refinement in screening, diagnostic, and therapeutic strategies. As the molecular understanding of cancer deepens, the clinical utility of biomarkers has grown significantly. An expanding repertoire of biomarkers is being incorporated into routine oncologic practice, driven by their potential to enhance diagnostic accuracy and inform therapeutic decision-making (de Gramont et al., 2015). In particular, predictive biomarkers offer the ability to identify patients most likely to benefit from specific treatments, thereby facilitating personalized approaches and optimizing clinical outcomes.

DNA methylation, a widespread and functionally important epigenetic modification, plays a pivotal role in gene regulation (Jones, 2012). Tumorigenesis is commonly characterized by global DNA hypomethylation in conjunction with focal hypermethylation at CpG island promoters. Hypermethylation of promoter-associated CpG islands in tumor suppressor genes is especially critical in the initiation and progression of malignancies (Downs et al., 2019; Halperin et al., 2022). Although the underlying mechanisms remain incompletely elucidated, DNA methylation alterations appear to exert early and profound effects during oncogenesis (Feinberg et al., 2006). Recent studies have implicated aberrant methylation patterns not only in breast cancer risk but also in mediating resistance to chemotherapy (Gómez-Miragaya et al., 2019; Ruiz-De et al., 2024). These findings highlight the importance of further investigation into methylation-driven genes, which may enhance the mechanistic understanding of tumor progression and inform the development of targeted therapeutic strategies. Clinically actionable methylation-based biomarkers could offer significant prognostic and therapeutic value, paving the way for more precise and individualized treatment paradigms.

OSR1, a Ste20-related protein kinase implicated in ion transport regulation, is evolutionarily conserved across both plant and animal kingdoms and plays a pivotal role in mammalian signal transduction pathways (Gagnon and Delpire, 2012). OSR1 mRNA exhibits widespread tissue distribution, while its protein localizes predominantly to the nuclei of cells across diverse tissue types (Chen et al., 2004). Given its critical role in intracellular signaling, OSR1 modulates a broad spectrum of biological processes. Its involvement was first recognized in the proper segmentation during Drosophila embryogenesis (Nüsslein-Volhard and Wieschaus, 1980; So and Danielian, 1999). Subsequent studies have demonstrated its significance in metabolic regulation, notably influencing the pathogenesis of non-alcoholic fatty liver disease by modulating lipid homeostasis and hepatic inflammation (Lynch et al., 2022). Emerging evidence highlights OSR1’s multifaceted functions in tumorigenesis. In renal cell carcinoma, OSR1 acts as a tumor suppressor by attenuating cellular invasiveness and proliferation (Zhang et al., 2017). In tongue squamous cell carcinoma, it inhibits tumor growth by suppressing NF-κB signaling (Chen et al., 2018). In lung cancer, OSR1 downregulates SOX9 and β-catenin expression, thereby modulating Wnt pathway activity (Wang et al., 2018). In breast cancer, elevated OSR1 expression has been associated with unfavorable prognoses and enhanced metastatic potential of endothelial cells, potentially mediated by altered TGF-β1 secretion through phosphorylation of the Smad2/3 linker region (Li et al., 2021; Li et al., 2020). These findings underscore the diverse oncological roles of OSR1 across multiple malignancies. Nonetheless, due to the inherent heterogeneity of breast cancer, the oncogenic relevance, clinical implications, and immunological context of OSR1 dysregulation remain incompletely characterized.

In the present study, OSR1 was identified as a methylation-driven gene in breast cancer through integrated analysis of the TCGA and GEO datasets. Comprehensive bioinformatics approaches were subsequently employed to assess its expression patterns, clinicopathological correlations, and prognostic value. The association between OSR1 expression, underlying molecular mechanisms, and immune cell infiltration was further elucidated to delineate its clinical relevance, inform targeted therapeutic strategies, and improve patient outcomes.

## Materials and methods

### Cell lines and culture conditions

The MCF-7 and MDA-MB-231 cell lines were purchased from Shanghai Zhongqiao Xinzhou Biotechnology Co., Ltd. After resuspension, MDA-MB-231 and MCF-7 cells were cultured in RPMI-1640 medium (Gibco, United States) supplemented with 10% fetal bovine serum (Clark, United States). These cells were passaged every 2–3 days in an incubator maintained at 37°C with 5% CO_2_.

## Construction of lentivirus packaged cell lines

MDA-MB-231 and MCF-7 cells were seeded into 24-well plates under optimal growth conditions. Lv-NC and Lv-OSR1 were added to the corresponding wells, and the cells were transfected overnight. On the second day post-transfection, the cell culture medium was replaced with fresh complete medium, and the cells were further cultured at 37°C. Fluorescence intensity was preliminarily evaluated under a fluorescence microscope. After 48 h of infection, cells were cultured in medium containing puromycin for 7 days to select for stable integrant cell lines (4 μg/mL for MDA-MB-231 and 2 μg/mL for MCF-7).

### Cell viability analysis

MDA-MB-231 and MCF-7 cells were seeded in 96-well plates at a density of 3 × 10^3^ cells per well. Cell viability was measured at 24 h, 48 h, and 72 h using the Cell Counting Kit-8 (CCK-8, BestBio, Shanghai, China). After incubation with the CCK-8 reagent at 37°C for 2 h, the optical density (OD) was measured at 450 nm.

### Colony formation assay

The virus-infected MDA-MB-231 and MCF-7 cells were prepared into a cell suspension at a concentration of 1.5 × 10^3^ cells/mL and seeded into six-well plates, followed by incubation at 37°C for 15 days to allow each cell colony to reach approximately 100 cells. The old medium was discarded, and the cells were washed twice with PBS. The cells were then fixed with 1 mL of paraformaldehyde per well. Subsequently, 1 mL of 0.1% crystal violet was added to each well, and the number of cell colonies was counted.

### Transwell assay

MCF-7 and MDA-MB-231 cells were resuspended in medium containing 5% FBS and placed in the upper chamber. The lower chamber was filled with medium containing 20% FBS. After 24 h, the migrated cells were fixed with paraformaldehyde and stained with crystal violet for counting.

### Western blot

Protease inhibitors were added to the protein lysis buffer to lyse the cells and obtain total protein samples. Subsequently, the protein concentrations were quantified using a bicinchoninic acid (BCA) assay kit, followed by electrophoresis and membrane transfer. The membrane was incubated overnight at 4°C with primary antibodies against Flag and β-actin (Proteintech, Wuhan, China), followed by incubation with secondary antibodies at room temperature for 2 h. Visualization was performed using an enhanced chemiluminescence (ECL) kit (Boster, United States). All antibodies were purchased from Proteintech (Wuhan, China).

### Xenograft tumor model

All animal experiments were conducted in accordance with the guidelines of the Animal Ethics Committee of the Institute of Health at the Hefei Comprehensive National Science Center. Female BALB/cA-nu nude mice (3–4 weeks old) were purchased from Nanjing Jicui Biotechnology Co., Ltd. MDA-MB-231 cells transfected with Lv-NC or Lv-OSR1 lentivirus (1 × 10^6^) were resuspended in 100 μL of PBS and injected subcutaneously into the mice. The mice were euthanized, and tumors were collected within 1 month for subsequent analyses, including weight and volume measurements, as well as immunohistochemistry.

### Immunohistochemistry (IHC)

Tumors were fixed in 10% formalin solution, embedded in paraffin, and sectioned into 5-μm-thick consecutive slices. After deparaffinization in xylene and dehydration in a graded series of ethanol, antigen retrieval was performed using citrate buffer. The sections were blocked with 5% goat serum and then incubated overnight at 4°C with the primary antibody. Subsequently, the sections were incubated with a peroxidase-conjugated goat anti-rabbit secondary antibody (Abcam, United States) at room temperature for 2 h and stained using a 3,3′-diaminobenzidine (DAB) kit (ZSGB-BIO, China).

### Data collection and processing

Methylation and mRNA expression data for breast cancer patients were downloaded from the TCGA database. This dataset included methylation data from 507 samples, consisting of 32 normal samples and 475 cancer samples, as well as mRNA expression data from 1,226 samples, including 113 normal samples. Initially, the LIMMA package was used to normalize and perform differential analysis on the downloaded data, identifying abnormally methylated genes and differentially expressed genes. The MethylMix algorithm, implemented in R, was subsequently used to calculate the correlation between gene methylation levels and gene expression. A β-mixture model was then constructed to identify significantly correlated genes and to determine disease-specific hypo- and hypermethylated genes. Finally, methylation-driven genes were screened. Additionally, RNA-seq data in the TPM format were obtained from the GTEx database for pan-cancer analysis.

### Differentially expressed gene analysis

Breast cancer patients in the TCGA database were divided into high- and low-expression groups based on the median expression score of OSR1. Differentially expressed genes (DEGs) between the two groups were analyzed using the DESeq2 package in R, with an adjusted p-value <0.05 and a |log2-fold-change (FC)| > 1 set as the threshold for significance. Spearman correlation analysis was employed to evaluate the correlation between the expression of the top 10 DEGs and OSR1.

### Functional enrichment analysis

Functional enrichment analysis of the differentially expressed genes was performed using the GOplot package (version 1.0.2) in R, encompassing both Gene Ontology (GO) and Kyoto Encyclopedia of Genes and Genomes (KEGG) analyses. Gene set enrichment analysis (GSEA) was conducted using the clusterProfiler package in R. An adjusted p-value <0.05 and a false discovery rate (FDR) < 0.25 were considered indicative of statistically significant enrichment of functional terms or pathways.

### Protein–protein interaction network analysis

Based on the differentially expressed genes, a protein–protein interaction (PPI) network was constructed using the online STRING database with a confidence score > 0.7, while all other parameters were set to the default. The PPI network was visualized using Cytoscape software (version 3.5.1).

### Immune infiltration analysis

Immune infiltration levels were calculated for 24 immune cell types, with the relative enrichment scores of these immune cells in breast cancer assessed via single-sample GSEA using the GSVA package in R. Spearman correlation analysis was employed to explore the relationship between OSR1 expression and these immune cells. The Wilcoxon rank-sum test was used to evaluate differences in immune infiltration levels between the OSR1 high-expression and low-expression groups.

### Survival analysis

Survival analysis was conducted using the Kaplan–Meier method and the log-rank test, with the cutoff value set at the median expression level of OSR1. Univariate and multivariate Cox regression analyses were employed to assess the impact of clinical variables on patient prognosis. Prognostic variables with a p-value <0.05 in the univariate Cox regression analysis were included in the multivariate Cox regression analysis. Visualization was performed using the ggplot2 package in R.

### Statistical analysis

All statistical analyses were performed using R (version 3.6.3). The Wilcoxon rank-sum test and paired sample t-test were used to evaluate the statistical significance of OSR1 expression in non-paired and paired tissues, respectively. The Wilcoxon rank-sum test and logistic regression were employed to assess the correlation between clinical characteristics and OSR1 expression. All tests were two-sided, and the primary analyses were conducted using Xiantao Academic (https://www.xiantao). In this study, *p < 0.05, **p < 0.01, and ***p < 0.001 were considered statistically significant.

## Results

### Identification of methylation-driven genes

**FIGURE 1:**
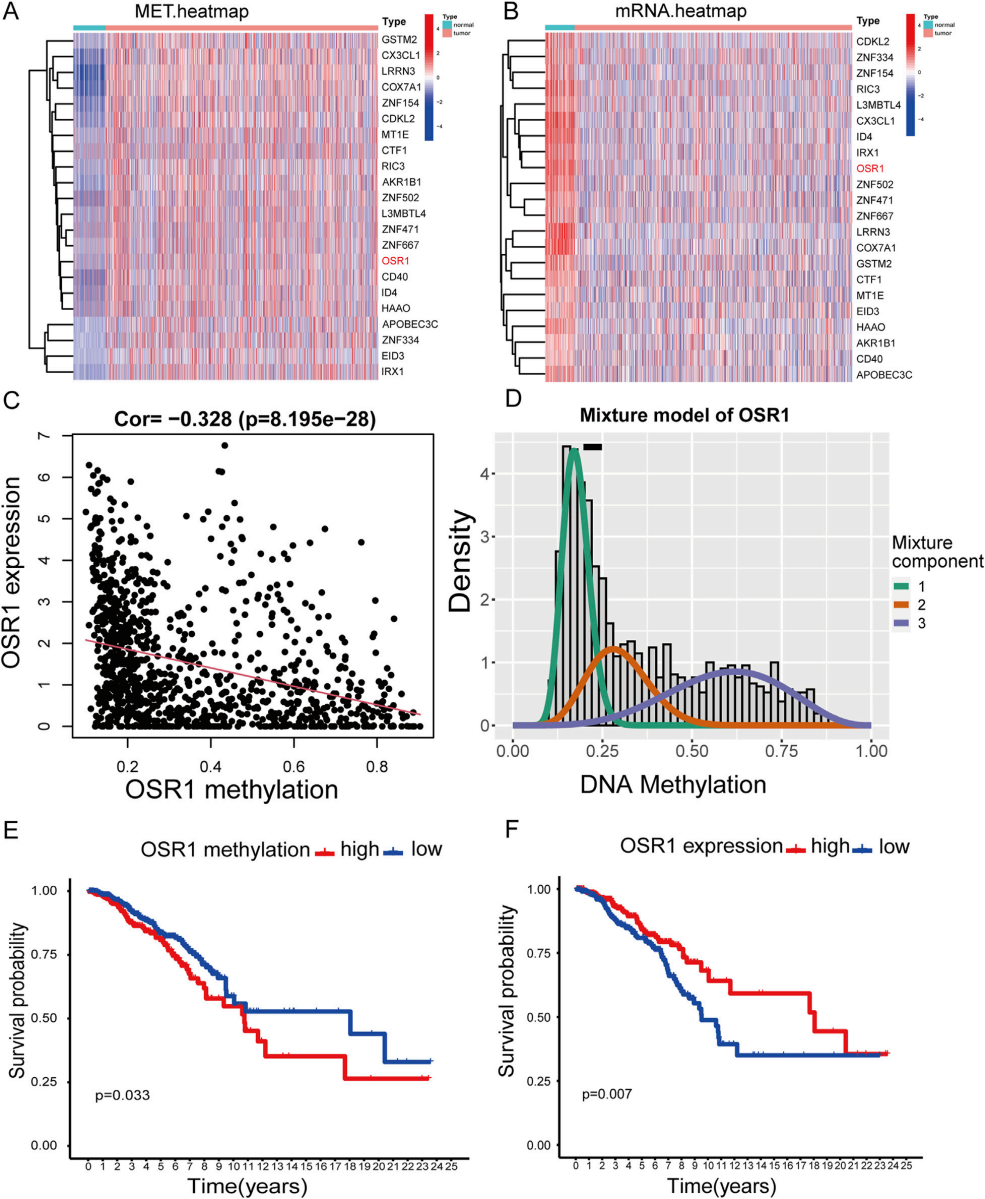
Heatmaps of methylation-driven genes and corresponding survival analysis. **(A)** Clustered heatmap illustrating methylation levels of genes in breast cancer, with a gradient from blue to red representing low to high methylation intensity. **(B)** Clustered heatmap of gene expression profiles, with red to blue indicating a transition from high- to low-expression levels. **(C,D)** Correlation analysis between OSR1 methylation status and its transcript abundance. **(E)** Kaplan–Meier (K–M) survival analysis of overall survival (OS) of patients with breast cancer in TCGA stratified by OSR1 methylation levels. **(F)** K–M survival curve of OS based on OSR1 expression levels in the TCGA cohort.

The study cohort consisted of 1,226 patients with breast cancer with available clinical annotations and RNA sequencing data, including 113 individuals with matched adjacent normal tissue samples from TCGA. To expand the normal tissue reference, gene expression data from an additional 179 normal breast tissue samples were retrieved from the GTEx database. The clinicopathological characteristics of the patient cohort are summarized in Supplementary Table S1. Aberrant methylation and gene expression data for BREAST CANCER were extracted from TCGA and analyzed using the LIMMA package. Relevant data were subsequently integrated for correlation analysis through the MethylMix package. Differential methylation was assessed *via* a mixture model framework and the Wilcoxon rank-sum test, with selection thresholds defined as |logFC| > 0, p < 0.05, and |Cor| > 0.3. This analysis identified 22 methylation-driven genes (Figures 1A,B). To further validate the potential clinical significance of these methylation-driven genes, we performed survival analysis and found that OSR1 expression was clinically relevant to the prognosis of breast cancer patients. (Figures 1C–F).

### Low expression of OSR1 in breast cancer

**FIGURE 2:**
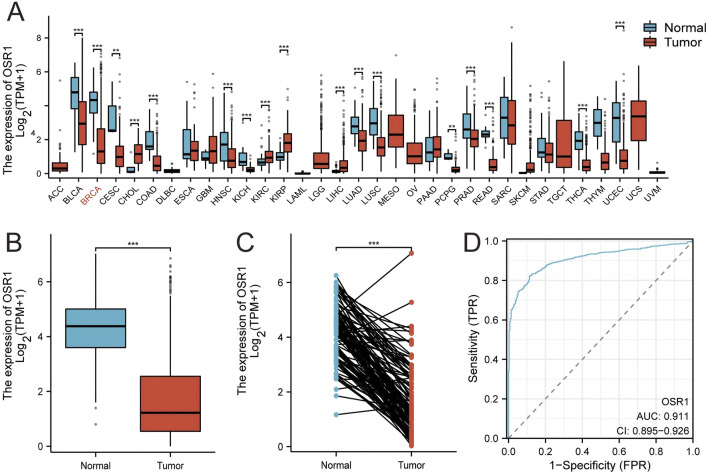
OSR1 expression patterns across tumor types and in breast cancer. **(A)** Comparative analysis of OSR1 expression across various tumor types and corresponding normal tissues using the TCGA and GTEx datasets. **(B)** OSR1 expression in breast cancer versus non-matched normal tissues from TCGA and GTEx. **(C)** Differential expression of OSR1 between breast cancer and matched adjacent normal tissues within TCGA. **(D)** ROC curve assessing the discriminative power of OSR1 expression between tumor and normal breast tissue in TCGA. TCGA: The Cancer Genome Atlas; GTEx: Genotype-tissue expression project; ROC: Receiver operating characteristic. *p < 0.05, **p < 0.01, ***p < 0.001.

Pan-cancer analysis revealed OSR1 underexpression in the majority of tumor types, including bladder urothelial carcinoma, cervical squamous cell carcinoma, colon cancer, lung adenocarcinoma, and lung squamous cell carcinoma, while overexpression was noted in select cancers such as cholangiocarcinoma (Figure 2A). OSR1 expression was significantly downregulated in breast cancer tissues relative to normal breast tissues (p < 0.001) (Figure 2B), a trend that was further validated in 113 paired tumor-normal samples (p < 0.001) (Figure 2C). Receiver operating characteristic (ROC) curve analysis demonstrated robust discriminatory power of OSR1 expression for distinguishing tumors from normal tissues (Figure 2D).

### Associations between OSR1 expression and clinicopathologic variables

**FIGURE 3:**
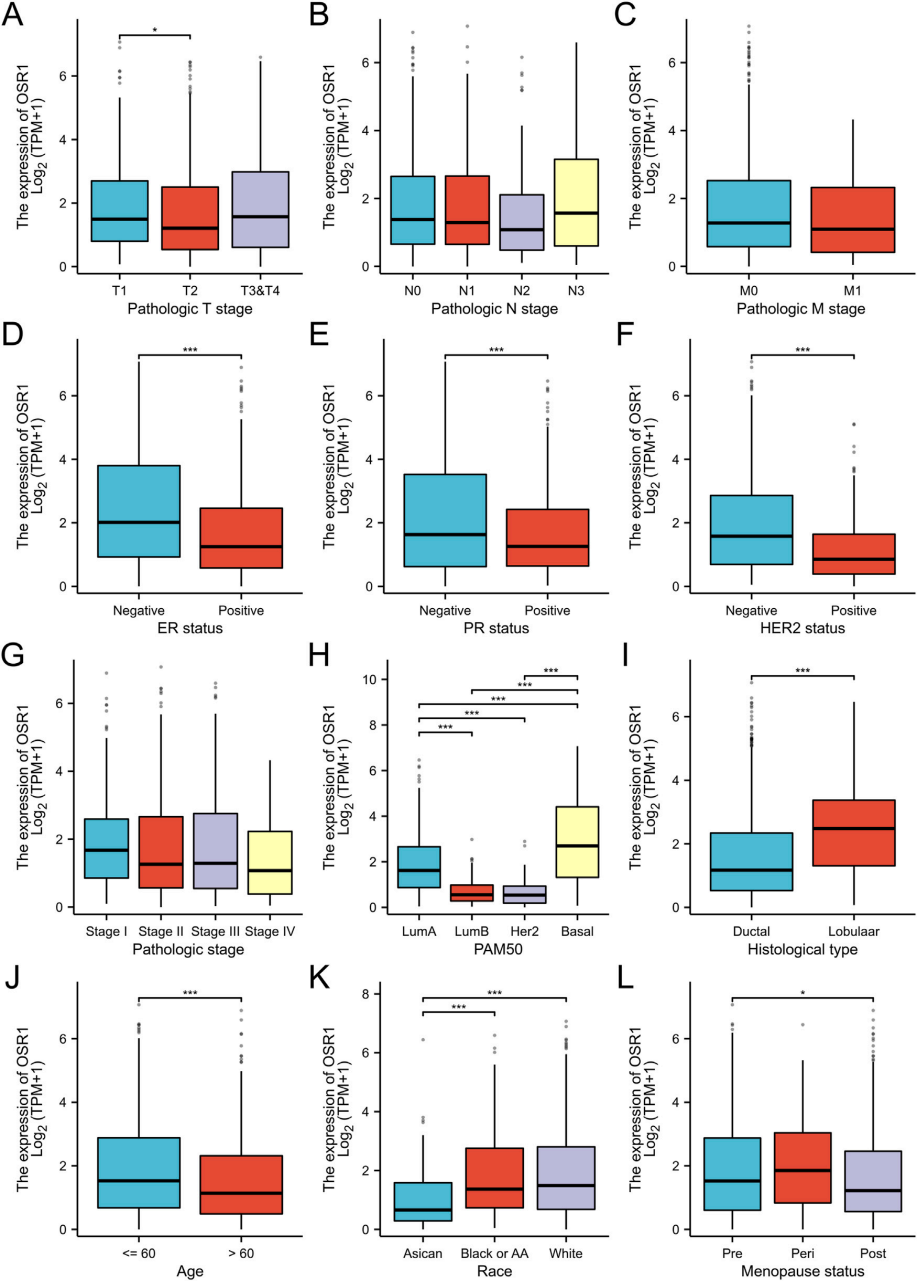
Association between OSR1 expression and clinicopathological parameters. **(A–L)** Stratification of OSR1 expression by T stage **(A)**, N stage **(B)**, M stage **(C)**, ER status **(D)**, PR status **(E)**, HER2 status **(F)**, pathological stage **(G)**, PAM50 subtype **(H)**, histological classification **(I)**, patient age **(J)**, race **(K)**, and menopausal status **(L)**. LumA: Luminal A; LumB: Luminal B; ER: Estrogen receptor; PR: Progesterone receptor; HER2: Human epidermal growth factor receptor 2.

As shown in Table 1 and Figure 3, elevated OSR1 expression was significantly associated with multiple clinicopathological parameters, including pathological stage II versus stage I (p < 0.001), estrogen receptor (ER), progesterone receptor (PR), and human epidermal growth factor receptor 2 (HER2) status (p < 0.001), PAM50 molecular subtype (p < 0.001), histological type (p < 0.001), patient age (p < 0.001), race (p < 0.001), and menopausal status (p = 0.023). Univariate logistic regression analysis identified notable associations between OSR1 expression levels and clinical characteristics. Specifically, significant differences were observed in T stage (OR = 2.134, 95% CI = 1.075–4.238, p = 0.030), race (OR = 3.723, 95% CI = 1.630–8.502, p = 0.002), histological type (OR = 4.135, 95% CI = 2.249–7.603, p < 0.001), HER2 status (OR = 0.261, 95% CI = 0.155–0.439, p < 0.001), and PAM50 subtype classification (OR = 3.723, 95% CI = 2.106–6.582, p < 0.001), as detailed in Table 2.

**TABLE 1:** Clinicopathological characteristics of high- and low-OSR1 expression groups.

Characteristics	Low expression of OSR1	High expression of OSR1	p-value
n	543	544	
Pathologic T stage, n (%)			< 0.001
T1	125 (11.5%)	153 (14.1%)	
T2	338 (31.2%)	293 (27%)	
T3	52 (4.8%)	88 (8.1%)	
T4	26 (2.4%)	9 (0.8%)	
Pathologic N stage, n (%)			0.151
N0	244 (22.8%)	272 (25.5%)	
N1	182 (17%)	177 (16.6%)	
N2	68 (6.4%)	48 (4.5%)	
N3	36 (3.4%)	41 (3.8%)	
Pathologic M stage, n (%)			0.749
M0	465 (50.3%)	440 (47.6%)	
M1	11 (1.2%)	9 (1%)	
Pathologic stage, n (%)			0.130
Stage I	76 (7.1%)	106 (10%)	
Stage II	316 (29.7%)	303 (28.5%)	
Stage III	126 (11.9%)	118 (11.1%)	
Stage IV	10 (0.9%)	8 (0.8%)	
Race, n (%)			<0.001
Asian	43 (4.3%)	17 (1.7%)	
Black or African American	88 (8.8%)	94 (9.4%)	
White	341 (34.2%)	414 (41.5%)	
Age, n (%)			<0.001
≤60	270 (24.8%)	333 (30.6%)	
>60	273 (25.1%)	211 (19.4%)	
Histological type, n (%)			<0.001
Infiltrating ductal carcinoma	431 (43.9%)	345 (35.2%)	
Infiltrating lobular carcinoma	51 (5.2%)	154 (15.7%)	
ER status, n (%)			<0.001
Negative	88 (8.5%)	152 (14.7%)	
Positive	419 (40.4%)	378 (36.5%)	
PR status, n (%)			0.002
Negative	145 (14%)	197 (19.1%)	
Positive	363 (35.1%)	329 (31.8%)	
HER2 status, n (%)			< 0.001
Negative	246 (34.3%)	314 (43.8%)	
Positive	108 (15.1%)	49 (6.8%)	
PAM50, n (%)			<0.001
LumA	237 (22.6%)	327 (31.2%)	
LumB	182 (17.4%)	24 (2.3%)	
Her2	72 (6.9%)	10 (1%)	
Basal	49 (4.7%)	146 (13.9%)	
Menopause status, n (%)			0.023
Pre	103 (11%)	127 (13.6%)	
Post	377 (40.3%)	329 (35.1%)	

**TABLE 2:** Associations of OSR1 expression with clinicopathological characteristics of patients.

Characteristics	Total (N)	OR (95% CI)	p-value
Pathologic T stage (T3&T4 vs. T1&T2)	404	2.134 (1.075–4.238)	**0.030**
Pathologic N stage (N2&N3&N1 vs. N0)	404	0.689 (0.465–1.020)	0.063
Pathologic M stage (M1 vs. M0)	404	0.887 (0.055–14.284)	0.933
Pathologic stage (Stage III & Stage IV vs. Stage I & Stage II)	404	0.810 (0.503–1.305)	0.387
Age (> 60 vs. ≤ 60)	404	0.857 (0.574–1.279)	0.451
Race (Black or African American & White vs. Asian)	404	3.723 (1.630–8.502)	**0.002**
Histological type (Infiltrating lobular carcinoma vs. Infiltrating ductal carcinoma)	404	4.135 (2.249–7.603)	**<0.001**
PR status (Positive vs. Negative)	404	0.904 (0.600–1.362)	0.629
ER status (Positive vs. Negative)	404	0.723 (0.457–1.142)	0.165
HER2 status (Positive vs. Negative)	404	0.261 (0.155–0.439)	**<0.001**
PAM50 (Basal vs. LumA & LumB & Her2)	404	3.723 (2.106–6.582)	**<0.001**
Menopause status (Post vs. Pre)	404	0.856 (0.553–1.325)	0.485

Bold values denote two-sided p < 0.05.

### Identification of DEGs and functional enrichment analysis

**FIGURE 4:**
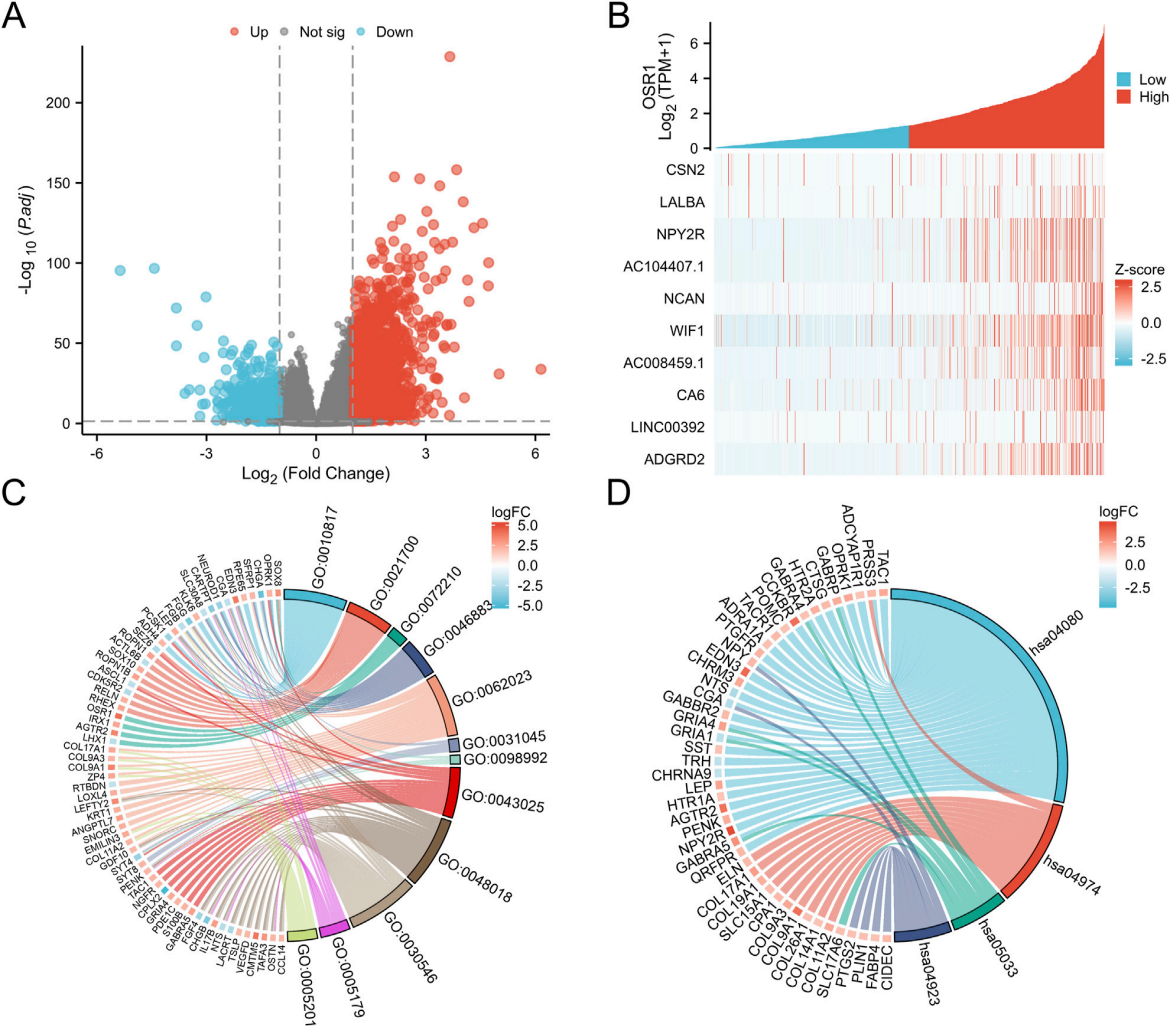
Differentially expressed genes (DEGs) associated with OSR1 and functional enrichment in breast cancer. **(A)** Volcano plot of OSR1-related DEGs, where red and blue dots denote significantly upregulated and downregulated genes, respectively. **(B)** Heatmap displaying correlations between OSR1 expression and the top 10 DEGs. **(C)** GO enrichment analysis of DEGs. **(D)** KEGG pathway enrichment analysis of DEGs. GO: Gene Ontology; KEGG: Kyoto Encyclopedia of Genes and Genomes; DEGs: differentially expressed genes. *p < 0.05, **p < 0.01, ***p < 0.001.

**FIGURE 5:**
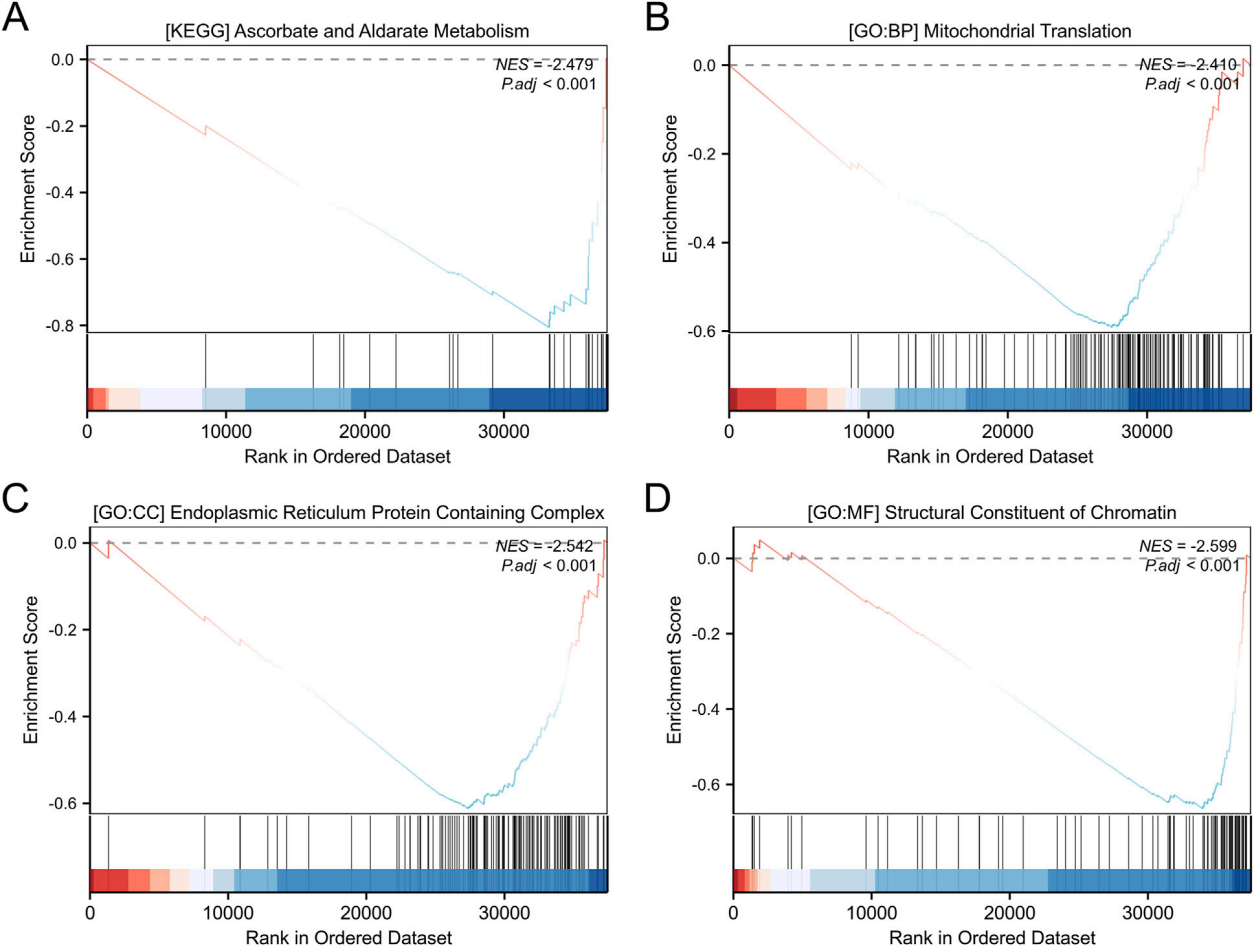
GO and KEGG enrichment analyses of differentially expressed genes. **(A)** KEGG pathway enrichment analysis of genes stratified by high and low expression levels. **(B–D)** GO functional annotation of DEGs, including biological processes **(B)**, cellular components **(C)**, and molecular functions **(D)**. GSEA: gene set enrichment analysis; NES: normalized enrichment score. A positive NES indicates that the gene set is enriched at the top of the ranked list (e.g., upregulated pathways), while a negative NES indicates enrichment at the bottom of the ranked list (e.g., downregulated pathways).

A total of 3,488 differentially expressed genes (DEGs) were identified between the high- and low-OSR1 expression groups, comprising 2,890 upregulated genes (82.86%) and 598 downregulated genes (17.14%) (adjusted p-value <0.05, |Log2-FC| > 1) (Figure 4A; Supplementary Table S2). The top 10 DEGs—CSN2, LALBA, NPY2R, AC104407.1, NCAN, WIF1, AC008459.1, CA6, LINC00392, and ADGRD2—exhibited notable correlations with OSR1 expression, as illustrated in Figure 4B. To investigate potential functional associations among the DEGs, combined Gene Ontology (GO) and Kyoto Encyclopedia of Genes and Genomes (KEGG) enrichment analyses were performed using logFC-based ranking. GO enrichment revealed significant involvement of DEGs in biological processes, such as peptide hormone secretion, peptide transport, peptide secretion, metal ion response, and forebrain development. KEGG analysis indicated enrichment in pathways that included neuroactive ligand–receptor interaction, protein digestion and absorption, nicotine addiction, and the regulation of lipolysis in adipocytes (Figures 4C,D). Gene set enrichment analysis (GSEA) further characterized the functional differences between the high- and low-OSR1 expression groups. GSEA analysis between the OSR1 high- and low-expression groups revealed that more organelle-related signaling pathways were downregulated in the OSR1 low-expression group (Figures 5A–D).

### Correlation between OSR1 expression and immune infiltration

**FIGURE 6:**
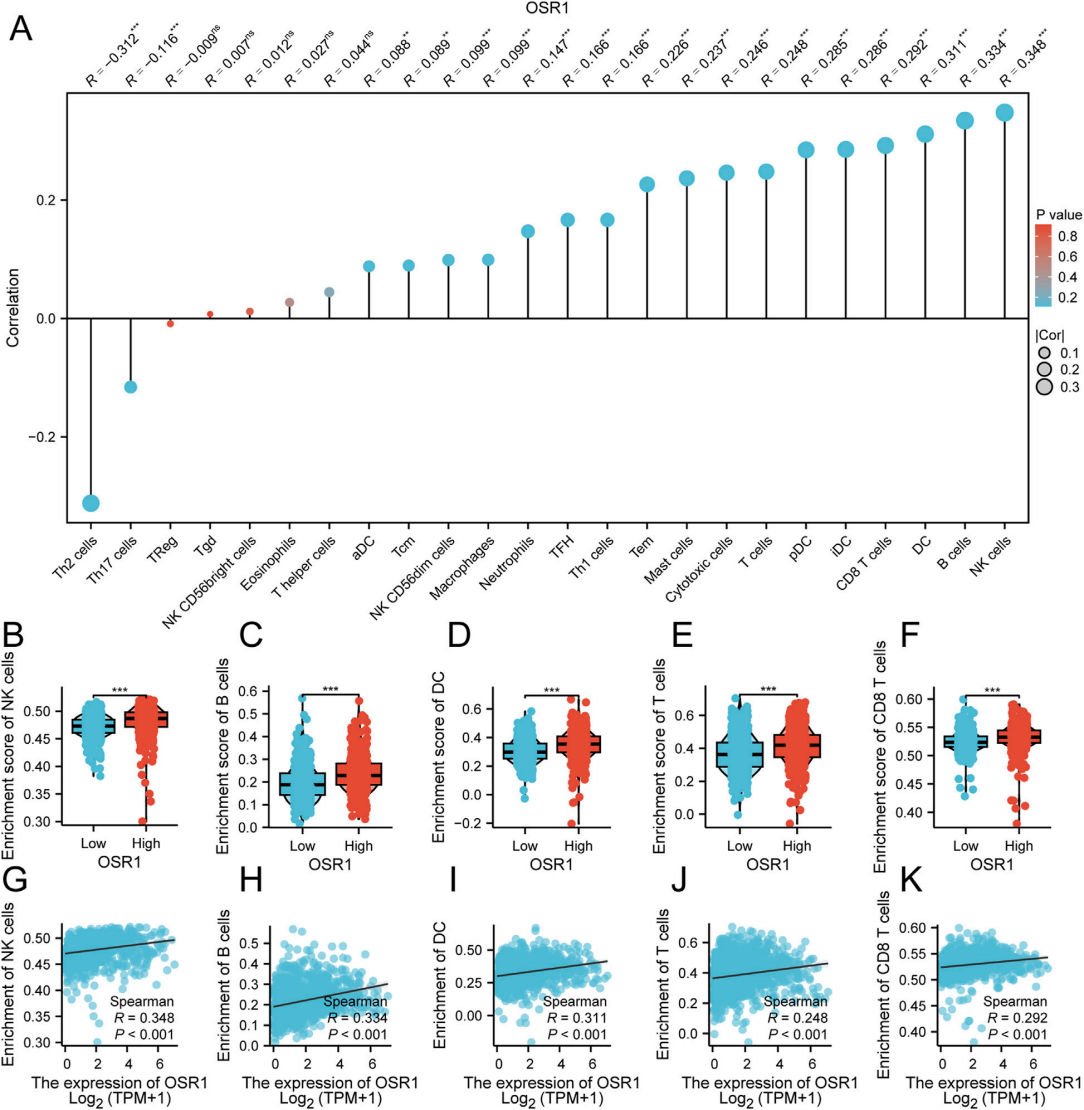
Association between OSR1 expression and immune cell infiltration in breast cancer. **(A)** Correlation matrix depicting associations between OSR1 expression and the relative abundance of 24 immune cell types. The dot size indicates the magnitude of the Spearman correlation coefficient. **(B–F)** Comparative analysis of immune infiltration levels between OSR1 high- and low-expression groups for NK cells, B cells, DC cells, T cells, and CD8^+^ T cells. **(G–K)** Correlation plots between OSR1 expression and enrichment scores for the same immune cell subsets. NK cells: Natural killer cells; DC cells: Dendritic cells.

The relationship between OSR1 expression and immune cell infiltration was also examined. Significant positive correlations were identified between OSR1 expression and the infiltration of natural killer (NK) cells (r = 0.341, p < 0.001), B cells (r = 0.328, p < 0.001), dendritic cells (DCs) (r = 0.307, p < 0.001), and plasmacytoid DCs (pDCs) (r = 0.291, p < 0.001) (Figure 6A). Comparative analysis between the two expression groups revealed significantly higher enrichment scores for B cells, T cells, NK cells, DCs, and CD8^+^ T cells in the OSR1 high-expression group (all p < 0.001) (Figures 6B–K). These results imply that elevated OSR1 expression is associated with a more active immune landscape and a potentially enhanced antitumor immune microenvironment.

### OSR1 suppresses tumorigenic capacity of breast cancer cell lines *in vitro*

**FIGURE 7:**
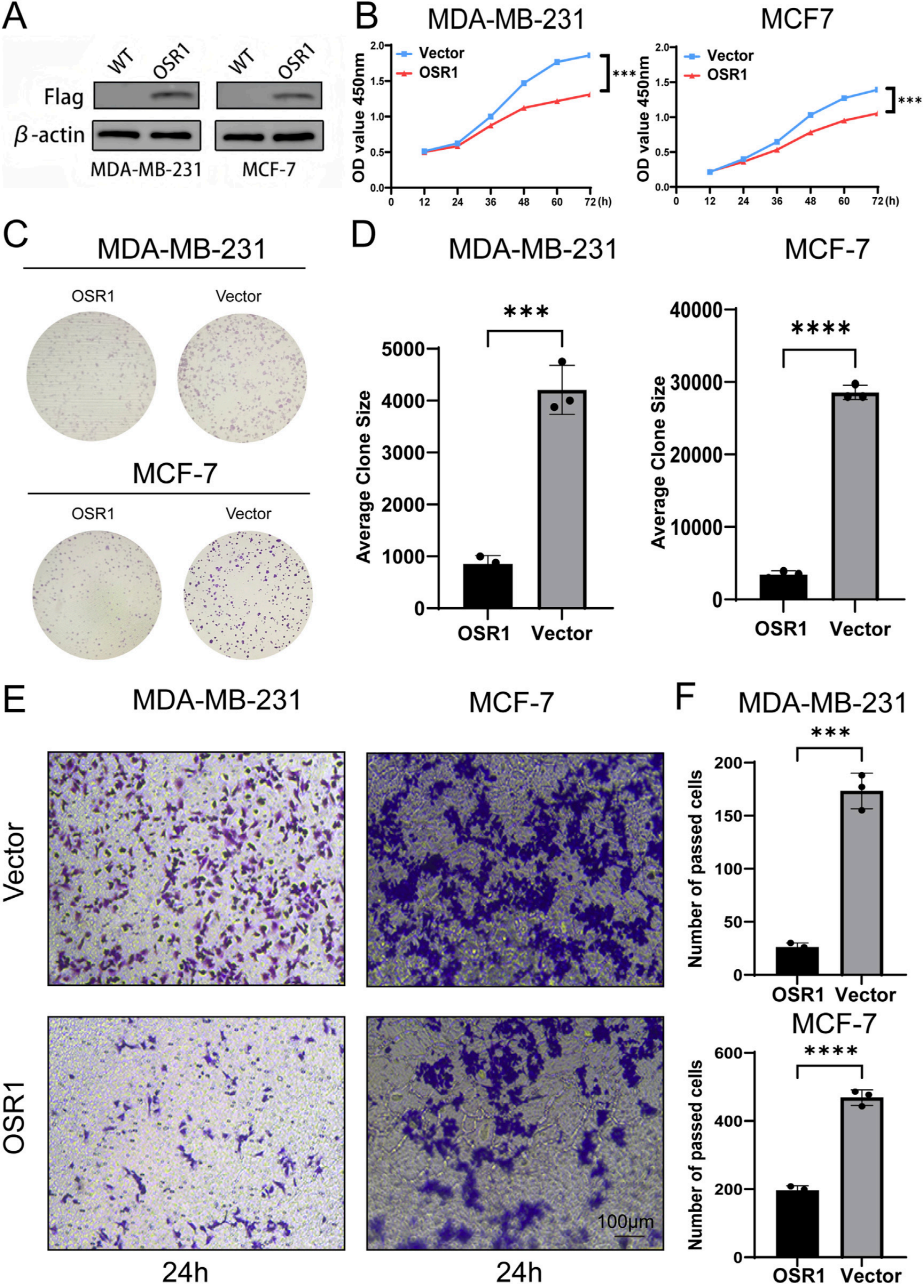
Functional effects of OSR1 overexpression on proliferation and migration in breast cancer cell lines. **(A)** Western blot validation of OSR1 overexpression *via* Flag-tagged lentiviral vectors in MDA-MB-231 and MCF-7 cells. **(B)** Cell proliferation rates were quantified using the CCK-8 assay. **(C,D)** Colony formation assays were performed to evaluate the impact of OSR1 overexpression on clonogenic potential. **(E,F)** Transwell migration assays assessed the migratory capability of OSR1-overexpressing MDA-MB-231 and MCF-7 cells. Scale bar: 100 μm. Data are presented as mean ± SD from three independent experiments unless otherwise stated. ***p < 0.001.

To assess the functional role of OSR1, overexpression experiments were performed in MDA-MB-231 and MCF-7 breast cancer cell lines. OSR1 plasmids tagged with Flag and containing a puromycin resistance gene were packaged into lentiviral vectors and used to infect both cell lines. Following 48 h of infection, puromycin selection was applied to establish stable overexpressing clones. Successful OSR1 overexpression was confirmed *via* Western blot analysis (Figure 7A). Short-term cell proliferation assays and long-term colony formation assays demonstrated that OSR1 overexpression significantly inhibited the proliferative capacity of MDA-MB-231 and MCF-7 cells (Figures 7B–D). Furthermore, *in vitro* Transwell migration assays revealed that OSR1 overexpression markedly suppressed the migratory potential of breast cancer cells (Figures 7E,F). These results suggest that OSR1 functions as a negative regulator of breast cancer cell proliferation and migration *in vitro*.

### OSR1 promotes breast cancer tumor progression *in vivo*

**FIGURE 8:**
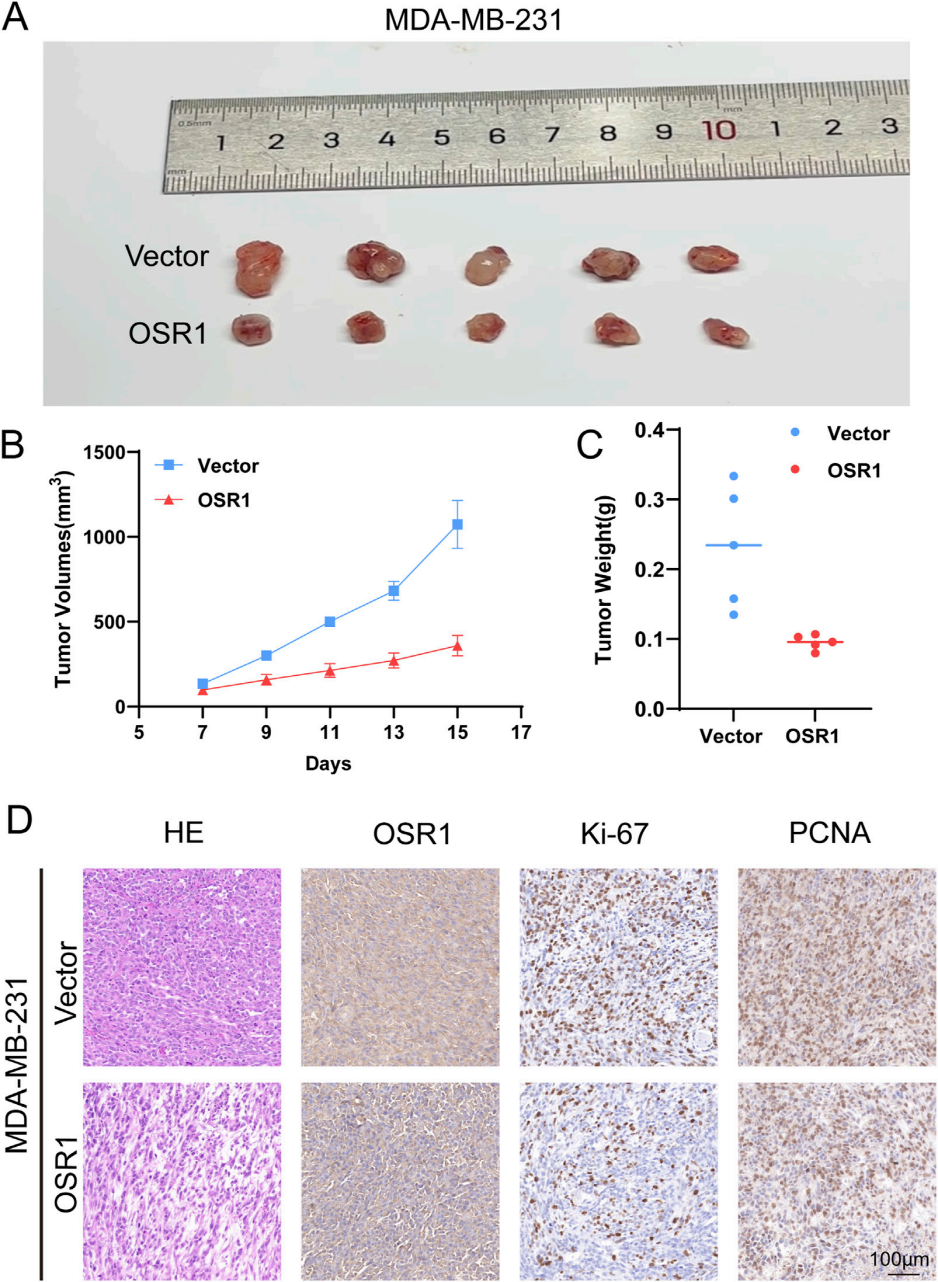
OSR1-mediated tumor promotion *in vivo*. **(A–D)** *In vivo* tumorigenicity following subcutaneous implantation of MDA-MB-231 cells into nude mice. **(A)** Representative images of excised tumors. **(B)** Tumor volume measurements. **(C)** Final tumor weights. **(D)** Histological evaluation of tumor sections by H&E staining and IHC detection of OSR1, Ki-67, and PCNA. Scale bar: 100 μm.

To further evaluate OSR1 function *in vivo*, xenograft experiments were conducted using nude mice. OSR1-overexpressing MDA-MB-231 cells were subcutaneously injected into animals stratified into control and OSR1 overexpression groups. Tumor growth was significantly suppressed in the OSR1 overexpression group, as evidenced by reduced tumor volume and weight relative to controls (Figures 8A–C). Histological examination through H&E staining and immunohistochemistry of tumor sections indicated a reduction in tumor malignancy following OSR1 overexpression. Notably, the expression of proliferation markers Ki-67 and PCNA (proliferating cell nuclear antigen) was substantially decreased in OSR1-overexpressing tumors (Figure 8D). Collectively, these results demonstrate that OSR1 upregulation inhibits malignant tumor progression *in vivo*.

## Discussion

Due to the intrinsic heterogeneity of breast cancer and the multifactorial nature of its prognosis, significant limitations persist in accurately predicting disease outcomes and identifying effective therapeutic targets. Consequently, the discovery of novel tumor biomarkers is essential for improving prognostic evaluation and advancing personalized treatment strategies. DNA methylation, a prevalent epigenetic modification, frequently leads to transcriptional silencing and plays a critical role in tumorigenesis. In this study, OSR1 expression and methylation status in breast cancer were analyzed using TCGA datasets. The results demonstrated significantly reduced OSR1 expression and elevated methylation levels in breast cancer tissues compared to normal counterparts, suggesting that OSR1 functions as a methylation-driven tumor suppressor gene. Previous investigations on methylated genes in breast cancer have predominantly emphasized gene panel construction for prognostic prediction. For example, a panel of 50 methylation-driven genes associated with patient survival was previously identified (Kuang et al., 2020). Other studies have integrated methylation profiling with drug target discovery to identify therapeutic gene loci for high-risk breast cancer subgroups (Tian et al., 2021). However, limited research has focused on elucidating the specific clinical associations and functional roles of individual methylated genes in tumor progression. This study not only identifies OSR1 as a methylation-driven gene but also delineates its functional and biological relevance in breast cancer.

The findings of this study confirmed that OSR1 is broadly underexpressed across multiple tumor types, including breast cancer, bladder urothelial carcinoma, lung adenocarcinoma, and lung squamous cell carcinoma, while elevated expression is observed in select cancers such as clear cell and papillary renal cell carcinomas. Notably, reduced OSR1 expression is associated with poorer survival outcomes in breast cancer, indicating its potential as an independent prognostic biomarker. Supporting evidence from ovarian cancer studies further corroborates the prognostic value of OSR1 downregulation (Yu and Ouyang, 2023). Additionally, OSR1 expression correlates significantly with hormone receptor status, molecular subtypes, and histopathological classifications in patients with breast cancer, underscoring its relevance to clinicopathological heterogeneity and reinforcing its potential as a clinically significant molecular target.

OSR1 plays a pivotal role in the regulation and transduction of intracellular signaling. Identified as the mammalian homolog of the yeast Ste20p serine/threonine kinase, OSR1 has been implicated in a variety of signaling cascades (Tamari et al., 1999). In gliomas, reduced OSR1 expression has been shown to influence cell migration by modulating the phosphorylation state of the Na^+^-K^+^-2Cl^–^cotransporter isoform 1 (NKCC1), subsequently altering intracellular Cl^−^and K^+^ concentrations (Zhu et al., 2014). Additionally, a well-established interaction exists between OSR1 and the WNK1 signaling pathway, as reported in multiple studies (Alessi et al., 2014; Dbouk et al., 2014; Taylor et al., 2018). Despite these insights, the functional role of OSR1 in breast cancer remains insufficiently characterized. GSEA enrichment analysis conducted in this study identified several pathways significantly associated with high OSR1 expression in breast cancer, including peptide hormone secretion, peptide transport, peptide secretion, metal ion response, forebrain development, neuroactive ligand-receptor interaction, protein digestion and absorption, and regulation of lipolysis in adipocytes. These enriched pathways align with previously reported functions of OSR1, particularly its role in regulating cation-chloride cotransporters (Alessi et al., 2014). Furthermore, OSR1 has been implicated in the progression of hepatic steatosis to non-alcoholic fatty liver disease, reinforcing its involvement in metabolic regulation (Zhou et al., 2021). Collectively, these findings support the validity of the current analytical results. However, the precise molecular mechanisms by which OSR1 contributes to breast cancer biology remain to be elucidated. Further experimental studies are warranted to clarify its functional impact and to uncover the underlying signaling pathways involved.

The tumor microenvironment (TME) plays a critical role in breast cancer, exerting a profound influence on tumor angiogenesis, immune evasion, early metastatic dissemination, prognosis, and therapeutic response prediction. Comprising tumor cells, infiltrating immune cells, and stromal components, the TME is a key determinant of tumor progression and therapeutic outcomes. Infiltrating immune cells have been recognized as predictors of response to neoadjuvant chemotherapy and immune checkpoint inhibitor (ICI) therapy (Havel et al., 2019). The impact of these immune cells is modulated by factors such as cell type, density, and spatial distribution (Widowati et al., 2020). Thus, comprehensive profiling of immune cell infiltration in breast cancer may not only enhance the optimization of ICI-based combination therapies but also provide prognostic and predictive insights for immunotherapeutic interventions. In this study, the relationship between OSR1 expression and immune cell infiltration was systematically evaluated, with differential analysis conducted between high and low OSR1 expression groups. A strong positive association was identified between OSR1 expression and tumor-infiltrating immune cells, particularly NK cells, DCs, and T cells. Activation of NK and DCs, as key components of the innate immune system, has been shown to suppress tumor growth, while elevated T cell infiltration is associated with enhanced antitumor immunity. These findings suggest that OSR1 may facilitate immune cell recruitment or activation, thereby modulating breast cancer progression and influencing patient prognosis.

To validate the bioinformatics predictions, OSR1-overexpressing cell lines were generated and subjected to both *in vitro* and *in vivo* functional assays. The experimental results were consistent with database-derived observations, confirming the inhibitory role of OSR1 in tumor proliferation. Despite the compelling evidence presented, several limitations must be acknowledged. The analysis was conducted using a single dataset, potentially introducing dataset-specific bias and limiting the generalizability of the findings. Furthermore, clinical information derived exclusively from publicly available databases lacked multicenter validation, thereby restricting the depth of clinical correlation analyses. Lastly, the experimental investigation focused primarily on the proliferative role of OSR1 without extensively addressing its involvement in immune modulation or downstream molecular signaling pathways.

In conclusion, the study demonstrated that elevated OSR1 expression is associated with reduced proliferation of breast cancer cells and enhanced immune cell infiltration within the TME. These findings highlight the potential of OSR1 as a novel prognostic biomarker and suggest that modulation of OSR1 expression could offer a promising therapeutic strategy in breast cancer management.

## Data Availability

Publicly available datasets were analyzed in this study. The mRNA expression profiles and clinical data of breast cancer patients from the Cancer Genome Atlas (TCGA) database can be found here:portal.gdc.cancer.gov/projects/TCGA-BRCA (TCGA-BRCA.). Additionally, data for pan-cancer analysis were obtained from the UCSC Xena database and the GTEx database here: https://xenabrowser.net/datapages/?host=https%3A%2F%2Ftoil.xenahubs.net.
