# Supplementary material for: Identification and validation of DNA methylation-driven gene OSR1 as a novel tumor suppressor for the diagnosis and prognosis of breast cancer
Source: Front Genet. 2025 Jul 7;16:1583620. doi: 10.3389/fgene.2025.1583620 (PMC12277919; doi:10.3389/fgene.2025.1583620)
Supplement: Supplementary file 1 [file Table1.docx]

TableS1

| Id | futime | fustat | Age | Gender | Grade | Stage | T | M | N |
| --- | --- | --- | --- | --- | --- | --- | --- | --- | --- |
| TCGA-A2-A0CT | 2289 | 0 | 71 | FEMALE | unknow | Stage IIA | T2 | M0 | N0 (i-) |
| TCGA-GM-A2DD | 2282 | 0 | 53 | FEMALE | unknow | Stage I | T1c | M0 | N0 (i-) |
| TCGA-D8-A1JM | 590 | 0 | 59 | FEMALE | unknow | Stage IIB | T2 | M0 | N1a |
| TCGA-BH-A18Q | 1692 | 1 | 56 | FEMALE | unknow | Stage IIB | T2 | M0 | N1b |
| TCGA-C8-A12P | 358 | 0 | 55 | FEMALE | unknow | Stage IIB | T2 | M0 | N1 |
| TCGA-S3-A6ZF | 572 | 0 | 64 | FEMALE | unknow | Stage IIA | T2 | M0 | N0 |
| TCGA-BH-A18K | 2763 | 1 | 46 | FEMALE | unknow | Stage I | T1 | M0 | N0 |
| TCGA-AN-A0FW | 11 | 0 | 67 | FEMALE | unknow | Stage IIIA | T2 | M0 | N2 |
| TCGA-D8-A140 | 403 | 0 | 62 | FEMALE | unknow | Stage IIB | T2 | M0 | N1a |
| TCGA-E2-A14R | 1174 | 0 | 62 | FEMALE | unknow | Stage IIA | T2 | M0 | N0 |
| TCGA-A8-A08C | 881 | 0 | 65 | FEMALE | unknow | Stage IIA | T2 | M0 | N0 |
| TCGA-D8-A3Z6 | 563 | 0 | 56 | FEMALE | unknow | Stage IIIC | T3 | M0 | N3a |
| TCGA-C8-A133 | 0 | 0 | 65 | FEMALE | unknow | Stage IIIA | T3 | M0 | N1 |
| TCGA-AN-A0FF | 172 | 0 | 32 | FEMALE | unknow | Stage IA | T1b | M0 | N0 |
| TCGA-D8-A27N | 519 | 0 | 36 | FEMALE | unknow | Stage IIIA | T2 | M0 | N2a |
| TCGA-D8-A1X5 | 565 | 0 | 81 | FEMALE | unknow | Stage IIIC | T2 | MX | N3a |
| TCGA-AR-A0TT | 3316 | 0 | 53 | FEMALE | unknow | Stage IIIA | T2 | M0 | N2 |
| TCGA-A8-A08F | 1004 | 0 | 59 | FEMALE | unknow | Stage IIIC | T2 | M0 | N3a |
| TCGA-BH-A0BA | 1132 | 0 | 51 | FEMALE | unknow | Stage IIIC | T3 | M0 | N3a |
| TCGA-EW-A3E8 | 1035 | 0 | 60 | FEMALE | unknow | Stage IIB | T3 | MX | N0 |
| TCGA-D8-A1XG | 448 | 0 | 86 | FEMALE | unknow | Stage IIIB | T4b | M0 | N1a |
| TCGA-E2-A15L | 626 | 0 | 65 | FEMALE | unknow | Stage IIA | T2 | M0 | N0 |
| TCGA-C8-A1HG | 345 | 0 | 50 | FEMALE | unknow | Stage IIA | T2 | M0 | N0 |
| TCGA-AO-A0JI | 1528 | 0 | 56 | FEMALE | unknow | Stage IIA | T1c | M0 | N1 |
| TCGA-LL-A73Z | 227 | 1 | 55 | FEMALE | unknow | Stage IV | T1c | M1 | NX |
| TCGA-E2-A1LL | 1309 | 0 | 73 | FEMALE | unknow | Stage IIIA | T3 | MX | N2a |
| TCGA-EW-A1PC | 187 | 0 | 66 | FEMALE | unknow | Stage IIB | T3 | MX | N0 (i-) |
| TCGA-AQ-A04H | 754 | 0 | 61 | FEMALE | unknow | Stage IIIA | T2 | MX | N2a |
| TCGA-AR-A251 | 3030 | 0 | 51 | FEMALE | unknow | Stage IIIA | T2 | M0 | N2 |
| TCGA-BH-A0AZ | 1919 | 0 | 47 | FEMALE | unknow | Stage IIIA | T3 | cM0 (i+) | N1a |
| TCGA-E9-A245 | 26 | 0 | 47 | FEMALE | unknow | Stage IIB | T2 | M0 | N1 |
| TCGA-BH-A0BQ | 2255 | 0 | 39 | FEMALE | unknow | Stage I | T1c | M0 | N0 |
| TCGA-A8-A09N | 31 | 0 | 57 | FEMALE | unknow | Stage IIIC | T2 | M0 | N3a |
| TCGA-E9-A1N4 | 1000 | 0 | 41 | FEMALE | unknow | Stage IIIA | T2 | M0 | N2 |
| TCGA-B6-A0IG | 4456 | 1 | 50 | FEMALE | unknow | Stage IIB | T2 | M0 | N1b |
| TCGA-AR-A0TV | 2288 | 0 | 66 | FEMALE | unknow | Stage IIA | T2 | M0 | N0 |
| TCGA-AC-A2FO | 2255 | 0 | 65 | FEMALE | unknow | Stage IIB | T2 | MX | N1 |
| TCGA-AR-A24P | 84 | 0 | 47 | FEMALE | unknow | Stage I | T1 | M0 | N0 |
| TCGA-EW-A1IZ | 554 | 0 | 53 | FEMALE | unknow | Stage IIIA | T2 | MX | N2a |
| TCGA-BH-A18F | 1001 | 0 | 50 | FEMALE | unknow | Stage IIA | T2 | M0 | N0 |
| TCGA-OK-A5Q2 | 64 | 0 | 59 | FEMALE | unknow | Stage II | T2 | MX | N1a |
| TCGA-A7-A26H | 724 | 0 | 72 | FEMALE | unknow | Stage IIA | T1 | M0 | N1a |
| TCGA-AN-A0FZ | 10 | 0 | 45 | FEMALE | unknow | Stage IIIA | T2 | M0 | N2 |
| TCGA-GM-A4E0 | 2191 | 0 | 67 | FEMALE | unknow | Stage IIIC | T3 | M0 | N3a |
| TCGA-A7-A4SD | 441 | 0 | 52 | FEMALE | unknow | Stage IIA | T2 | M0 | N0 |
| TCGA-A2-A0T7 | 631 | 0 | 51 | FEMALE | unknow | Stage IIA | T2 | M0 | N0 (i+) |
| TCGA-BH-A0B1 | 1148 | 0 | 66 | FEMALE | unknow | Stage IIB | T2 | M0 | N1a |
| TCGA-E2-A152 | 2128 | 0 | 56 | FEMALE | unknow | Stage I | T1c | M0 | N0 |
| TCGA-EW-A1J3 | 504 | 0 | 61 | FEMALE | unknow | Stage IA | T1c | M0 | N0 |
| TCGA-A8-A097 | 365 | 0 | 65 | FEMALE | unknow | Stage IIB | T2 | M0 | N1 |
| TCGA-BH-A1ET | 2520 | 1 | 55 | FEMALE | unknow | Stage I | T1 | M0 | N0 |
| TCGA-AR-A2LQ | 1233 | 0 | 59 | FEMALE | unknow | Stage IIB | T3 | M0 | N0 |
| TCGA-E9-A5FK | 812 | 0 | 60 | FEMALE | unknow | Stage IIIC | T2 | M0 | N3 |
| TCGA-E9-A22G | 1239 | 0 | 47 | FEMALE | unknow | Stage IIA | T2 | M0 | N0 |
| TCGA-GI-A2C8 | 225 | 0 | 63 | FEMALE | unknow | Stage IIIB | T4b | MX | N0 |
| TCGA-AC-A8OS | 70 | 0 | 71 | FEMALE | unknow | Stage IIA | T2 | MX | N0 (i-) |
| TCGA-AC-A7VB | 250 | 0 | 51 | FEMALE | unknow | Stage IIA | T1c | MX | N1a |
| TCGA-A2-A0T1 | 521 | 0 | 55 | FEMALE | unknow | Stage IIIC | T3 | M0 | N3 |
| TCGA-OL-A5RW | 1106 | 0 | 40 | FEMALE | unknow | Stage IIA | T1c | MX | N1 |
| TCGA-AR-A1AJ | 2383 | 0 | 83 | FEMALE | unknow | Stage I | T1 | M0 | N0 |
| TCGA-E9-A243 | 612 | 0 | 52 | FEMALE | unknow | Stage IIA | T2 | M0 | N0 (i-) |
| TCGA-A2-A0EP | 3603 | 0 | 56 | FEMALE | unknow | Stage I | T1 | M0 | N0 (i+) |
| TCGA-AC-A2FF | 2759 | 0 | 40 | FEMALE | unknow | Stage IIB | T2 | MX | N1 |
| TCGA-AR-A1AL | 2971 | 0 | 60 | FEMALE | unknow | Stage IIIA | T3 | M0 | N1 |
| TCGA-A8-A0A4 | 396 | 0 | 73 | FEMALE | unknow | Stage IIA | T2 | M0 | N0 |
| TCGA-D8-A1JA | 502 | 0 | 60 | FEMALE | unknow | unknow | T4b | M0 | NX |
| TCGA-A8-A07G | 577 | 0 | 65 | FEMALE | unknow | Stage IIA | T1c | M0 | N1a |
| TCGA-E2-A10B | 1141 | 0 | 67 | FEMALE | unknow | Stage IIB | T2 | M0 | N1a |
| TCGA-AR-A24U | 3128 | 0 | 47 | FEMALE | unknow | Stage IIA | T1 | M0 | N1 |
| TCGA-B6-A40B | 3152 | 0 | 76 | FEMALE | unknow | Stage I | T1b | M0 | N0 (i-) |
| TCGA-E2-A15K | 275 | 0 | 58 | FEMALE | unknow | Stage IIB | T2 | M0 | N1a |
| TCGA-AC-A5XS | 588 | 0 | 74 | FEMALE | unknow | Stage IIA | T2 | MX | N0 |
| TCGA-A7-A4SE | 644 | 0 | 54 | FEMALE | unknow | Stage IIA | T2 | M0 | N0 |
| TCGA-GM-A5PV | 412 | 0 | 63 | FEMALE | unknow | Stage IIB | T2 | M0 | N1a |
| TCGA-E9-A295 | 375 | 0 | 71 | FEMALE | unknow | Stage IIA | T2 | M0 | N0 (i-) |
| TCGA-AR-A2LO | 1198 | 0 | 46 | FEMALE | unknow | Stage IIB | T2 | M0 | N1 |
| TCGA-E9-A1N8 | 1039 | 0 | 48 | FEMALE | unknow | Stage IIA | T2 | M0 | N0 |
| TCGA-A2-A0CW | 3283 | 0 | 67 | FEMALE | unknow | Stage IIB | T2 | M0 | N1a |
| TCGA-A7-A0DC | 906 | 0 | 63 | FEMALE | unknow | Stage IA | T1c | M0 | N0 (i-) |
| TCGA-AO-A0J9 | 1613 | 0 | 61 | FEMALE | unknow | Stage IIIC | T2 | M0 | N3 |
| TCGA-A8-A09Q | 761 | 0 | 83 | FEMALE | unknow | Stage IIIB | T4b | M0 | N2a |
| TCGA-C8-A26V | 616 | 0 | 47 | FEMALE | unknow | Stage IIIA | T3 | M0 | N2 |
| TCGA-EW-A6S9 | 463 | 0 | 34 | FEMALE | unknow | Stage IIA | T1c | M0 | N1 |
| TCGA-BH-A1F2 | 959 | 1 | 53 | FEMALE | unknow | Stage IIIB | T4b | M0 | N1b |
| TCGA-E2-A1B5 | 984 | 0 | 46 | FEMALE | unknow | Stage IIA | T2 | M0 | N0 |
| TCGA-AO-A0JD | 2190 | 0 | 59 | FEMALE | unknow | Stage IIIA | T3 | M0 | N1a |
| TCGA-E9-A1RI | 1449 | 0 | 43 | FEMALE | unknow | Stage IIIA | T1c | M0 | N2 |
| TCGA-E2-A15E | 630 | 0 | 40 | FEMALE | unknow | Stage IIA | T1c | M0 | N1a |
| TCGA-A8-A09A | 304 | 0 | 40 | FEMALE | unknow | Stage X | T2 | M0 | NX |
| TCGA-AR-A0TZ | 3262 | 1 | 43 | FEMALE | unknow | unknow | T2 | M1 | N2 |
| TCGA-W8-A86G | 347 | 0 | 66 | FEMALE | unknow | Stage IIA | T2a | MX | N0 (i-) |
| TCGA-E2-A15S | 428 | 0 | 34 | FEMALE | unknow | Stage IIB | T2 | M0 | N1 |
| TCGA-A2-A0YK | 588 | 0 | 61 | FEMALE | unknow | Stage IIA | T2 | M0 | N0 (i-) |
| TCGA-BH-A18V | 1556 | 1 | 48 | FEMALE | unknow | Stage IIB | T2 | M0 | N1b |
| TCGA-OL-A5RU | 1219 | 0 | 63 | FEMALE | unknow | Stage IIA | T1c | MX | N1 |
| TCGA-BH-A0EB | 745 | 0 | 69 | FEMALE | unknow | Stage IA | T1c | M0 | N0 (i-) |
| TCGA-BH-A0HK | 178 | 0 | 81 | FEMALE | unknow | Stage IIB | T2 | M0 | N1 |
| TCGA-AQ-A1H3 | 989 | 0 | 49 | FEMALE | unknow | Stage IIIC | T2 | MX | N3a |
| TCGA-AR-A1AM | 2991 | 0 | 52 | FEMALE | unknow | Stage IIIA | T3 | M0 | N1 |
| TCGA-LL-A5YN | 447 | 0 | 46 | FEMALE | unknow | Stage IIA | T2 | M0 | N0 (i-) |
| TCGA-E2-A1LB | 2306 | 0 | 41 | FEMALE | unknow | Stage IIB | T2 | M0 | N1 |
| TCGA-B6-A0IJ | 7106 | 0 | 42 | FEMALE | unknow | Stage IIB | T3 | M0 | N0 (i-) |
| TCGA-XX-A899 | 467 | 0 | 46 | FEMALE | unknow | Stage IIIA | T1c | MX | N2a |
| TCGA-E2-A14Z | 563 | 1 | 64 | FEMALE | unknow | Stage I | T1c | M0 | N0 |
| TCGA-AO-A0J6 | 1140 | 0 | 61 | FEMALE | unknow | Stage IIA | T2 | M0 | N0 (i-) |
| TCGA-GM-A5PX | 551 | 0 | 65 | FEMALE | unknow | Stage IIB | T2 | M0 | N1mi |
| TCGA-AO-A0JM | 2184 | 0 | 40 | FEMALE | unknow | Stage IIB | T2 | M0 | N1 |
| TCGA-E9-A1QZ | 755 | 0 | 61 | FEMALE | unknow | Stage IIA | T2 | M0 | N0 |
| TCGA-BH-A1EY | 538 | 1 | 79 | FEMALE | unknow | Stage IIA | T2 | M0 | N0 |
| TCGA-E2-A1BD | 1133 | 0 | 53 | FEMALE | unknow | Stage IIA | T2 | M0 | N0 |
| TCGA-AR-A0U2 | 2551 | 1 | 47 | FEMALE | unknow | Stage IIIA | T2 | M0 | N2 |
| TCGA-UL-AAZ6 | 518 | 0 | 73 | FEMALE | unknow | Stage IIA | T2 | MX | N0 |
| TCGA-BH-A28Q | 1119 | 0 | 46 | FEMALE | unknow | Stage IIB | T2 | M0 | N1a |
| TCGA-AC-A3YI | 707 | 0 | 74 | FEMALE | unknow | Stage IIIC | T1c | MX | N3a |
| TCGA-C8-A3M8 | 394 | 0 | 68 | FEMALE | unknow | Stage IA | T1c | M0 | N0 |
| TCGA-E9-A248 | 59 | 0 | 51 | FEMALE | unknow | Stage IIA | T2 | M0 | N0 (i-) |
| TCGA-A7-A3J1 | 343 | 0 | 63 | FEMALE | unknow | Stage IA | T1c | MX | N0 |
| TCGA-AO-A03N | 2031 | 0 | 59 | FEMALE | unknow | Stage IIB | T2 | M0 | N1a |
| TCGA-A2-A0SY | 1347 | 0 | 62 | FEMALE | unknow | Stage IIIA | T3 | M0 | N1 |
| TCGA-A8-A099 | 304 | 0 | 76 | FEMALE | unknow | Stage X | T4b | MX | N3a |
| TCGA-AR-A0TQ | 2991 | 0 | 27 | FEMALE | unknow | Stage IIIA | T3 | M0 | N1 |
| TCGA-AO-A12B | 2989 | 0 | 63 | FEMALE | unknow | Stage IIA | T2 | M0 | N0 (i-) |
| TCGA-A2-A3XV | 996 | 0 | 46 | FEMALE | unknow | Stage IIA | T2 | M0 | N0 |
| TCGA-D8-A27T | 398 | 0 | 53 | FEMALE | unknow | Stage IIIC | T2 | M0 | N3a |
| TCGA-GM-A2DM | 3226 | 0 | 57 | FEMALE | unknow | Stage IIA | T2 | M0 | N0 (i-) |
| TCGA-C8-A12N | 358 | 0 | 58 | FEMALE | unknow | Stage IIA | T2 | M0 | N0 (i-) |
| TCGA-C8-A12V | 385 | 0 | 55 | FEMALE | unknow | Stage IIA | T2 | M0 | N0 |
| TCGA-AO-A03T | 2124 | 0 | 42 | FEMALE | unknow | Stage IIB | T2 | M0 | N1a |
| TCGA-D8-A1X6 | 541 | 0 | 80 | FEMALE | unknow | Stage IIIA | T3 | MX | N2a |
| TCGA-AR-A252 | 2838 | 0 | 50 | FEMALE | unknow | Stage I | T1 | M0 | N0 |
| TCGA-LL-A5YP | 450 | 0 | 49 | FEMALE | unknow | Stage IIB | T2 | M0 | N1a |
| TCGA-A7-A6VV | 313 | 0 | 51 | FEMALE | unknow | Stage IIA | T2 | M0 | N0 |
| TCGA-E2-A1L7 | 1836 | 0 | 40 | FEMALE | unknow | Stage IIIA | T2 | M0 | N2 |
| TCGA-E9-A5UP | 803 | 0 | 63 | FEMALE | unknow | Stage IIA | T2 | M0 | N0 |
| TCGA-A2-A259 | 1596 | 0 | 70 | FEMALE | unknow | Stage I | T1c | M0 | N0 (i-) |
| TCGA-BH-A0AY | 777 | 0 | 62 | FEMALE | unknow | Stage IIA | T2 | M0 | N0 (i-) |
| TCGA-E2-A1B1 | 2653 | 0 | 45 | FEMALE | unknow | Stage IIB | T2 | M0 | N1 |
| TCGA-EW-A1P8 | 239 | 1 | 58 | FEMALE | unknow | Stage IIIC | T2 | M0 | N3b |
| TCGA-E2-A10A | 1229 | 0 | 41 | FEMALE | unknow | Stage IIB | T3 | M0 | N0 (i-) |
| TCGA-D8-A1X7 | 509 | 0 | 40 | FEMALE | unknow | Stage IIA | T2 | MX | N0 |
| TCGA-E9-A1RH | 1417 | 0 | 63 | FEMALE | unknow | Stage IIA | T2 | M0 | N0 |
| TCGA-AR-A0TW | 3009 | 0 | 50 | FEMALE | unknow | Stage IIIA | T3 | M0 | N1 |
| TCGA-A2-A0CX | 1728 | 0 | 52 | FEMALE | unknow | Stage IIA | T2 | M0 | N0 (i-) |
| TCGA-AR-A5QM | 2231 | 0 | 62 | FEMALE | unknow | Stage IIA | T2 | M0 | N0 |
| TCGA-A8-A09E | 1492 | 0 | 73 | FEMALE | unknow | Stage IIIB | T4b | M0 | N1a |
| TCGA-AN-A03Y | 10 | 0 | 66 | FEMALE | unknow | Stage IIA | T2 | M0 | N0 |
| TCGA-HN-A2OB | 1900 | 1 | 45 | FEMALE | unknow | Stage IIB | T2 | M0 | N1 |
| TCGA-E2-A15O | 289 | 0 | 89 | FEMALE | unknow | Stage I | T1c | M0 | NX |
| TCGA-GM-A2DK | 2645 | 0 | 58 | FEMALE | unknow | Stage I | T1c | M0 | N0 (i-) |
| TCGA-E2-A576 | 1043 | 0 | 69 | FEMALE | unknow | Stage IA | T1c | MX | N0 |
| TCGA-E2-A150 | 1935 | 0 | 48 | FEMALE | unknow | Stage IIA | T2 | M0 | N0 |
| TCGA-LD-A74U | 402 | 0 | 79 | FEMALE | unknow | Stage IIIC | T3 | M0 | N3a |
| TCGA-A7-A26J | 627 | 0 | 49 | FEMALE | unknow | Stage IIA | T2 | M0 | N0 |
| TCGA-BH-A18L | 811 | 1 | 50 | FEMALE | unknow | Stage IIIA | T3 | M0 | N1mi |
| TCGA-B6-A0IP | 3926 | 1 | 74 | FEMALE | unknow | Stage IA | T1c | M0 | N0 (i-) |
| TCGA-5T-A9QA | 303 | 0 | 52 | FEMALE | unknow | Stage IIA | T2 | MX | NX |
| TCGA-BH-A0BJ | 660 | 0 | 41 | FEMALE | unknow | Stage IIB | T2 | M0 | N1a |
| TCGA-D8-A1J9 | 532 | 0 | 48 | FEMALE | unknow | Stage IA | T1c | M0 | N0 |
| TCGA-B6-A0WV | 2417 | 1 | 67 | FEMALE | unknow | Stage IIB | T2 | M0 | N1b |
| TCGA-EW-A1OX | 911 | 0 | 43 | FEMALE | unknow | Stage IIA | T2 | MX | N0 |
| TCGA-D8-A1XO | 1682 | 0 | 56 | FEMALE | unknow | Stage IIB | T2 | M0 | N1a |
| TCGA-D8-A1JT | 405 | 0 | 70 | FEMALE | unknow | Stage IIA | T2 | M0 | N0 |
| TCGA-A2-A25A | 3276 | 0 | 44 | FEMALE | unknow | Stage IIA | T2 | M0 | N0 (i-) |
| TCGA-E9-A54Y | 725 | 0 | 63 | FEMALE | unknow | Stage IIIA | T2 | M0 | N2 |
| TCGA-AN-A0XS | 10 | 0 | 63 | FEMALE | unknow | Stage IIIA | T2 | M0 | N2 |
| TCGA-BH-A0BR | 2330 | 0 | 59 | FEMALE | unknow | Stage I | T1c | M0 | N0 |
| TCGA-AC-A3QP | 675 | 0 | 79 | FEMALE | unknow | Stage IIB | T2 | MX | N1mi |
| TCGA-AN-A041 | 7 | 0 | 29 | FEMALE | unknow | Stage IIB | T2 | M0 | N1a |
| TCGA-BH-A0GY | 923 | 0 | 67 | FEMALE | unknow | Stage IIA | T2 | M0 | N1 |
| TCGA-E2-A1IL | 118 | 0 | 78 | FEMALE | unknow | Stage IIA | T1c | M0 | N1a |
| TCGA-A8-A0A7 | 30 | 0 | 57 | FEMALE | unknow | Stage IIB | T2 | M0 | N1a |
| TCGA-BH-A0EE | 943 | 0 | 68 | FEMALE | unknow | Stage IIB | T3 | M0 | N0 (i-) |
| TCGA-BH-A0EA | 991 | 1 | 72 | FEMALE | unknow | Stage IIA | T1c | M0 | N1a |
| TCGA-A8-A082 | 549 | 0 | 58 | FEMALE | unknow | Stage IIB | T2 | M0 | N1a |
| TCGA-D8-A1JK | 0 | 0 | 90 | FEMALE | unknow | Stage IIA | T2 | M0 | N0 |
| TCGA-A8-A08S | 1004 | 0 | 71 | FEMALE | unknow | Stage IIA | T1c | M0 | N1 |
| TCGA-AN-A0XT | 10 | 0 | 54 | FEMALE | unknow | Stage IIIA | T2 | M0 | N2 |
| TCGA-E2-A1IN | 675 | 0 | 60 | FEMALE | unknow | Stage I | T1c | M0 | N0 |
| TCGA-B6-A0I9 | 362 | 1 | 62 | FEMALE | unknow | Stage IV | T3 | M1 | NX |
| TCGA-A2-A4S3 | 666 | 0 | 59 | FEMALE | unknow | Stage IIB | T2 | M0 | N1a |
| TCGA-C8-A12L | 363 | 0 | 67 | FEMALE | unknow | Stage IIA | T2 | M0 | N0 (i-) |
| TCGA-AC-A23H | 0 | 0 | 90 | FEMALE | unknow | Stage IIA | T2 | M0 | NX |
| TCGA-AC-A2FB | 1234 | 0 | 65 | FEMALE | unknow | Stage IIA | T2 | MX | N0 |
| TCGA-E2-A14P | 1246 | 0 | 79 | FEMALE | unknow | Stage IIIC | T2 | M0 | N3 |
| TCGA-C8-A12Z | 382 | 0 | 45 | FEMALE | unknow | Stage IIB | T2 | M0 | N1 |
| TCGA-A8-A09W | 30 | 0 | 70 | FEMALE | unknow | Stage IIIB | T2 | M0 | N3 |
| TCGA-AO-A0JA | 655 | 0 | 36 | FEMALE | unknow | Stage IIIC | T2 | M0 | N3 |
| TCGA-A8-A0AB | 518 | 0 | 54 | FEMALE | unknow | Stage IIA | T2 | M0 | N0 |
| TCGA-BH-A1F5 | 2712 | 1 | 62 | FEMALE | unknow | Stage IIA | T1c | M0 | N1a |
| TCGA-AN-A0FS | 210 | 0 | 55 | FEMALE | unknow | Stage IA | T1c | M0 | N0 |
| TCGA-A2-A0T3 | 1516 | 0 | 37 | FEMALE | unknow | Stage IB | T1c | M0 | N1mi |
| TCGA-B6-A0RS | 3063 | 1 | 38 | FEMALE | unknow | Stage IIA | T2 | M0 | N0 (i-) |
| TCGA-A8-A08H | 0 | 0 | 66 | FEMALE | unknow | Stage IIA | T2 | M0 | N0 |
| TCGA-A2-A04N | 4354 | 0 | 66 | FEMALE | unknow | Stage IA | T1c | M0 | N0 (i-) |
| TCGA-E9-A1R3 | 78 | 0 | 70 | FEMALE | unknow | Stage IIIC | T2 | M0 | N3 |
| TCGA-BH-A0GZ | 328 | 0 | 62 | FEMALE | unknow | Stage IIA | T1c | M0 | N1a |
| TCGA-A8-A08P | 943 | 0 | 70 | FEMALE | unknow | Stage IIIA | T2 | M0 | N2a |
| TCGA-A7-A425 | 447 | 0 | 70 | FEMALE | unknow | Stage IIIC | T3 | MX | N3a |
| TCGA-E2-A15A | 710 | 0 | 45 | FEMALE | unknow | Stage IIIC | T2 | M0 | N3a |
| TCGA-MS-A51U | 681 | 0 | 44 | FEMALE | unknow | Stage IIB | T2 | M0 | N1 |
| TCGA-A7-A5ZX | 336 | 0 | 48 | FEMALE | unknow | Stage IIIC | T2 | M0 | N3a |
| TCGA-A8-A09B | 365 | 0 | 58 | FEMALE | unknow | Stage IIIB | T4b | M0 | N1a |
| TCGA-S3-A6ZH | 641 | 0 | 29 | FEMALE | unknow | Stage IIIC | T3 | MX | N3a |
| TCGA-A2-A0YF | 1535 | 0 | 67 | FEMALE | unknow | Stage I | T1b | M0 | N0 (i+) |
| TCGA-A2-A1FW | 528 | 0 | 62 | FEMALE | unknow | Stage IIIA | T3 | M0 | N2a |
| TCGA-BH-A0E2 | 435 | 0 | 49 | FEMALE | unknow | Stage IIIA | T2 | M0 | N2a |
| TCGA-E9-A1ND | 1266 | 0 | 75 | FEMALE | unknow | Stage IIB | T2 | M0 | N1 |
| TCGA-S3-AA10 | 586 | 0 | 65 | FEMALE | unknow | Stage IIA | T2 | M0 | N0 |
| TCGA-B6-A0RH | 6456 | 1 | 51 | FEMALE | unknow | Stage IIA | T2 | M0 | N0 (i-) |
| TCGA-A7-A4SB | 418 | 0 | 56 | FEMALE | unknow | Stage IIIA | T3 | M0 | N1mi |
| TCGA-E2-A15P | 595 | 0 | 61 | FEMALE | unknow | Stage IA | T1c | M0 | N0 |
| TCGA-A7-A0CG | 1043 | 0 | 78 | FEMALE | unknow | Stage IIA | T2 | M0 | N0 (i-) |
| TCGA-3C-AALK | 1448 | 0 | 52 | FEMALE | unknow | Stage IA | T1c | M0 | N0 (i+) |
| TCGA-E9-A244 | 21 | 0 | 54 | FEMALE | unknow | Stage IIA | T2 | M0 | N0 (i-) |
| TCGA-AO-A03L | 2442 | 0 | 34 | FEMALE | unknow | Stage IIIA | T3 | M0 | N2 |
| TCGA-OL-A5S0 | 620 | 0 | 66 | FEMALE | unknow | Stage IIB | T2 | MX | N1a |
| TCGA-E2-A570 | 931 | 0 | 47 | FEMALE | unknow | Stage IB | T1c | M0 | N1mi |
| TCGA-AR-A0TY | 1699 | 1 | 54 | FEMALE | unknow | Stage IIA | T2 | M0 | N0 |
| TCGA-AR-A256 | 2854 | 1 | 45 | FEMALE | unknow | Stage IIA | T2 | M0 | N0 |
| TCGA-BH-A1EU | 1286 | 1 | 83 | FEMALE | unknow | Stage I | T1 | M0 | N0 |
| TCGA-LL-A6FQ | 80 | 0 | 77 | FEMALE | unknow | Stage IIIA | T2 | MX | N2a |
| TCGA-GM-A2DI | 2590 | 0 | 52 | FEMALE | unknow | Stage I | T1c | M0 | N0 (i+) |
| TCGA-A7-A0DB | 1007 | 0 | 56 | FEMALE | unknow | Stage IIA | T2 | M0 | N0 (i-) |
| TCGA-BH-A0BM | 1876 | 0 | 54 | FEMALE | unknow | Stage IIB | T2 | M0 | N1a |
| TCGA-A8-A081 | 0 | 0 | 80 | FEMALE | unknow | Stage IIA | T2 | M0 | N0 |
| TCGA-3C-AAAU | 4047 | 0 | 55 | FEMALE | unknow | Stage X | TX | MX | NX |
| TCGA-D8-A1XF | 463 | 0 | 45 | FEMALE | unknow | Stage IIA | T2 | M0 | N0 |
| TCGA-E9-A229 | 1148 | 0 | 37 | FEMALE | unknow | Stage IA | T1c | M0 | N0 |
| TCGA-D8-A4Z1 | 659 | 0 | 68 | FEMALE | unknow | Stage IA | T1c | M0 | N0 |
| TCGA-OL-A5RY | 752 | 0 | 52 | FEMALE | unknow | Stage IIA | T1c | MX | N1 |
| TCGA-AC-A23G | 2248 | 0 | 76 | FEMALE | unknow | Stage IIA | T1c | MX | N1 |
| TCGA-E9-A1R7 | 1467 | 0 | 64 | FEMALE | unknow | Stage IIA | T2 | M0 | N0 |
| TCGA-C8-A27B | 439 | 0 | 48 | FEMALE | unknow | Stage IIB | T3 | M0 | N0 |
| TCGA-B6-A409 | 573 | 1 | 44 | FEMALE | unknow | Stage IIIA | T1c | M0 | N2a |
| TCGA-E2-A109 | 1417 | 0 | 64 | FEMALE | unknow | Stage IIA | T2 | M0 | N0 (i-) |
| TCGA-AC-A3BB | 987 | 0 | 46 | FEMALE | unknow | Stage IIIA | T3 | MX | N2a |
| TCGA-EW-A3U0 | 532 | 0 | 61 | FEMALE | unknow | Stage IIIA | T3 | M0 | N1a |
| TCGA-A2-A04P | 548 | 1 | 36 | FEMALE | unknow | Stage IIIC | T2 | M0 | N3c |
| TCGA-A8-A083 | 0 | 0 | 67 | FEMALE | unknow | Stage IIB | T3 | M0 | N0 |
| TCGA-E2-A14O | 1359 | 0 | 76 | FEMALE | unknow | Stage IIIA | T3 | M0 | N1a |
| TCGA-BH-A0B0 | 2477 | 0 | 56 | FEMALE | unknow | Stage I | T1c | M0 | N0 |
| TCGA-AN-A0AJ | 303 | 0 | 79 | FEMALE | unknow | Stage IIB | T3 | M0 | N0 |
| TCGA-C8-A26X | 376 | 0 | 58 | FEMALE | unknow | Stage IIA | T1 | M0 | N1 |
| TCGA-D8-A27P | 49 | 0 | 64 | FEMALE | unknow | Stage IA | T1c | M0 | N0 |
| TCGA-BH-A8FY | 295 | 1 | 87 | FEMALE | unknow | Stage IA | T1c | M0 | N0 (i+) |
| TCGA-OL-A5RZ | 679 | 0 | 57 | FEMALE | unknow | Stage IA | T1b | MX | N0 |
| TCGA-EW-A1OV | 789 | 0 | 56 | FEMALE | unknow | Stage IIB | T2 | MX | N1mi |
| TCGA-AR-A1AO | 2618 | 0 | 47 | FEMALE | unknow | Stage IIA | T1 | M0 | N1 |
| TCGA-AN-A04C | 54 | 0 | 51 | FEMALE | unknow | Stage IIB | T2 | M0 | N1 |
| TCGA-A2-A25E | 3204 | 0 | 34 | FEMALE | unknow | Stage IIIA | T2 | M0 | N2a |
| TCGA-A2-A1G6 | 501 | 0 | 50 | FEMALE | unknow | Stage IIIA | T2 | M0 | N2a |
| TCGA-AR-A1AK | 3159 | 0 | 70 | FEMALE | unknow | Stage I | T1 | M0 | N0 |
| TCGA-AC-A3W6 | 0 | 0 | 90 | FEMALE | unknow | Stage IIIA | T3 | MX | N1 |
| TCGA-BH-A0HO | 76 | 0 | 48 | FEMALE | unknow | Stage IIA | T1c | M0 | N1a |
| TCGA-S3-A6ZG | 562 | 0 | 71 | FEMALE | unknow | Stage IIB | T2 | M0 | N1 |
| TCGA-BH-A1FJ | 1927 | 1 | 66 | FEMALE | unknow | Stage IIIA | T3 | M0 | N1b |
| TCGA-E2-A1IG | 2140 | 0 | 45 | FEMALE | unknow | Stage IIB | T2 | M0 | N1mi |
| TCGA-BH-A0B6 | 2483 | 0 | 47 | FEMALE | unknow | Stage I | T1c | M0 | N0 |
| TCGA-AN-A0FT | 214 | 0 | 63 | FEMALE | unknow | Stage IIB | T2 | M0 | N1 |
| TCGA-EW-A6SB | 760 | 0 | 62 | FEMALE | unknow | Stage II | T2 | M0 | N0 |
| TCGA-B6-A0IM | 3873 | 1 | 75 | FEMALE | unknow | Stage IIB | T3 | M0 | N0 (i-) |
| TCGA-AO-A03O | 2483 | 1 | 69 | FEMALE | unknow | Stage IIA | T2 | M0 | N0 (i-) |
| TCGA-BH-A0EI | 1926 | 0 | 51 | FEMALE | unknow | Stage IIA | T1c | M0 | N1a |
| TCGA-BH-A18J | 612 | 1 | 56 | FEMALE | unknow | Stage IV | T4b | M1 | N2 |
| TCGA-C8-A1HN | 394 | 0 | 56 | FEMALE | unknow | Stage IIA | T2 | M0 | N0 |
| TCGA-GM-A2DA | 6593 | 1 | 46 | FEMALE | unknow | Stage IIB | T2 | M0 | N1b |
| TCGA-A8-A08J | 1127 | 1 | 52 | FEMALE | unknow | Stage IV | T4b | M1 | N3 |
| TCGA-EW-A1PH | 607 | 0 | 52 | FEMALE | unknow | Stage IIA | T1c | M0 | N1a |
| TCGA-B6-A0X5 | 2097 | 1 | 61 | FEMALE | unknow | Stage IIB | T2 | M0 | N1b |
| TCGA-EW-A1OY | 908 | 0 | 63 | FEMALE | unknow | Stage IIA | T2 | MX | N0 |
| TCGA-BH-A2L8 | 612 | 0 | 45 | FEMALE | unknow | Stage IIA | T2 | M0 | N0 (i-) |
| TCGA-BH-A1FL | 1673 | 1 | 69 | FEMALE | unknow | Stage IIB | T2 | M0 | N1b |
| TCGA-C8-A9FZ | 592 | 0 | 49 | FEMALE | unknow | Stage IIB | T2 | M0 | N1 |
| TCGA-B6-A0X4 | 860 | 1 | 62 | FEMALE | unknow | Stage IIB | T2 | M0 | N1b |
| TCGA-AO-A03M | 1866 | 0 | 29 | FEMALE | unknow | Stage I | T1c | M0 | N0 (i-) |
| TCGA-PL-A8LZ | 302 | 0 | 29 | FEMALE | unknow | Stage IIIB | T4b | M0 | N2 |
| TCGA-A2-A0CR | 3283 | 0 | 54 | FEMALE | unknow | Stage IIB | T3 | M0 | N0 |
| TCGA-A8-A0A2 | 579 | 0 | 66 | FEMALE | unknow | Stage IIA | T2 | M0 | N0 |
| TCGA-A7-A0CD | 1165 | 0 | 66 | FEMALE | unknow | Stage I | T1 | M0 | N0 |
| TCGA-EW-A1P5 | 703 | 0 | 77 | FEMALE | unknow | Stage IIB | T2 | M0 | N1a |
| TCGA-E2-A1IO | 1855 | 0 | 37 | FEMALE | unknow | Stage I | T1c | M0 | N0 |
| TCGA-A8-A08L | 30 | 1 | 89 | FEMALE | unknow | Stage IIIA | T3 | M0 | N2a |
| TCGA-AR-A2LJ | 2632 | 0 | 40 | FEMALE | unknow | Stage IIIA | T3 | MX | N1 |
| TCGA-BH-A1FC | 3472 | 1 | 78 | FEMALE | unknow | Stage IIA | T1c | M0 | N1b |
| TCGA-A8-A08A | 30 | 0 | 89 | FEMALE | unknow | Stage I | T1c | M0 | N0 |
| TCGA-PE-A5DD | 1953 | 0 | 64 | FEMALE | unknow | Stage IIA | T2 | M0 | N0 |
| TCGA-GI-A2C9 | 3342 | 0 | 58 | FEMALE | unknow | Stage IIB | T3 | MX | N0 |
| TCGA-D8-A27M | 410 | 0 | 59 | FEMALE | unknow | Stage IA | T1c | M0 | N0 |
| TCGA-E9-A1RF | 200 | 0 | 68 | FEMALE | unknow | Stage IIIA | T2 | M0 | N2 |
| TCGA-E9-A1RB | 976 | 1 | 40 | FEMALE | unknow | Stage IIA | T2 | M0 | N0 |
| TCGA-A8-A096 | 0 | 0 | 73 | FEMALE | unknow | Stage IIA | T2 | M0 | N0 |
| TCGA-D8-A1JG | 1612 | 0 | 62 | FEMALE | unknow | Stage IIA | T2 | M0 | N0 |
| TCGA-BH-A0DO | 1644 | 0 | 78 | FEMALE | unknow | Stage I | T1c | M0 | N0 |
| TCGA-A1-A0SQ | 554 | 0 | 45 | FEMALE | unknow | Stage IIB | T2 | MX | N1 |
| TCGA-LL-A50Y | 762 | 0 | 84 | FEMALE | unknow | Stage IIA | T2 | MX | N0 |
| TCGA-E2-A1L8 | 2240 | 0 | 52 | FEMALE | unknow | Stage IIB | T2 | M0 | N1a |
| TCGA-C8-A132 | 383 | 0 | 56 | FEMALE | unknow | Stage IIB | T2 | M0 | N1 |
| TCGA-EW-A2FR | 1673 | 0 | 59 | FEMALE | unknow | Stage IIIC | T1b | MX | N3a |
| TCGA-AR-A0U4 | 3261 | 0 | 54 | FEMALE | unknow | Stage IIA | T2 | M0 | N0 |
| TCGA-A2-A0D3 | 1873 | 0 | 42 | FEMALE | unknow | Stage IA | T1c | M0 | N0 (i-) |
| TCGA-BH-A1FU | 1688 | 1 | 44 | FEMALE | unknow | Stage IA | T1c | M0 | N0 |
| TCGA-E9-A1N6 | 678 | 1 | 52 | FEMALE | unknow | Stage IIB | T2 | M0 | N1 |
| TCGA-BH-A0DZ | 495 | 0 | 43 | FEMALE | unknow | Stage IIB | T2 | M0 | N1a |
| TCGA-A2-A0YE | 554 | 0 | 48 | FEMALE | unknow | Stage IIB | T2 | M0 | N1a |
| TCGA-D8-A3Z5 | 1015 | 0 | 54 | FEMALE | unknow | Stage IIIC | T2 | M0 | N3a |
| TCGA-LL-A740 | 441 | 0 | 61 | FEMALE | unknow | Stage IA | T1c | MX | N0 (i-) |
| TCGA-AC-A3TN | 456 | 0 | 75 | FEMALE | unknow | Stage IIB | T3 | MX | N0 |
| TCGA-A8-A07W | 304 | 0 | 76 | FEMALE | unknow | Stage IV | T2 | M1 | NX |
| TCGA-GM-A2DH | 2193 | 0 | 58 | FEMALE | unknow | Stage I | T1c | M0 | N0 (i+) |
| TCGA-E9-A22B | 1167 | 0 | 71 | FEMALE | unknow | Stage IA | T1c | M0 | N0 |
| TCGA-C8-A12Q | 385 | 1 | 78 | FEMALE | unknow | Stage IIIA | T1 | M0 | N2 |
| TCGA-A8-A06T | 1614 | 0 | 75 | FEMALE | unknow | Stage IIIA | T3 | M0 | N1a |
| TCGA-BH-A18N | 468 | 1 | 88 | FEMALE | unknow | Stage IIA | T2 | M0 | N1b |
| TCGA-BH-A0DS | 78 | 0 | 71 | FEMALE | unknow | Stage IIIA | T2 | M0 | N2a |
| TCGA-AR-A0U1 | 4052 | 0 | 36 | FEMALE | unknow | Stage IIB | T2 | M0 | N1 |
| TCGA-B6-A0X1 | 7455 | 1 | 48 | FEMALE | unknow | unknow | T2 | M1 | N1 |
| TCGA-A1-A0SH | 1437 | 0 | 39 | FEMALE | unknow | Stage IIA | T2 | M0 | N0 (i-) |
| TCGA-BH-A0H9 | 1247 | 0 | 69 | FEMALE | unknow | Stage IIA | T2 | M0 | N0 (i-) |
| TCGA-BH-A0HL | 72 | 0 | 56 | FEMALE | unknow | Stage IIB | T2 | M0 | N1a |
| TCGA-E2-A1AZ | 2329 | 0 | 63 | FEMALE | unknow | Stage IIB | T2 | M0 | N1a |
| TCGA-BH-A42T | 320 | 1 | 75 | FEMALE | unknow | Stage IIA | T2 | M0 | N0 (i+) |
| TCGA-D8-A27G | 409 | 0 | 75 | FEMALE | unknow | Stage IIA | T2 | M0 | N0 |
| TCGA-BH-A42U | 3364 | 0 | 80 | FEMALE | unknow | Stage IIA | T2 | M0 | N0 |
| TCGA-A8-A06X | 943 | 1 | 77 | FEMALE | unknow | Stage IIB | T3 | M0 | N0 |
| TCGA-AO-A1KT | 541 | 0 | 78 | FEMALE | unknow | Stage IIA | T1 | M0 | N1 |
| TCGA-WT-AB44 | 883 | 0 | 77 | FEMALE | unknow | Stage IA | T1c | MX | N0 (i-) |
| TCGA-AN-A0FJ | 242 | 0 | 59 | FEMALE | unknow | Stage IV | T2 | M1 | N2 |
| TCGA-BH-A0H6 | 747 | 0 | 82 | FEMALE | unknow | Stage I | T1b | M0 | NX |
| TCGA-B6-A0IC | 0 | 1 | 90 | FEMALE | unknow | Stage X | T2 | MX | NX |
| TCGA-EW-A1IW | 371 | 0 | 80 | FEMALE | unknow | Stage IIB | T2 | MX | N1a |
| TCGA-D8-A1JL | 611 | 0 | 72 | FEMALE | unknow | Stage IIA | T2 | M0 | N0 |
| TCGA-A7-A3IZ | 322 | 0 | 62 | FEMALE | unknow | Stage IIA | T2 | M0 | N0 (i+) |
| TCGA-A2-A3XW | 1712 | 0 | 42 | FEMALE | unknow | Stage IIB | T2 | M0 | N1a |
| TCGA-BH-A1EW | 1694 | 1 | 38 | FEMALE | unknow | Stage IIA | T2 | M0 | N1b |
| TCGA-E9-A1R4 | 186 | 0 | 66 | FEMALE | unknow | Stage IA | T1c | M0 | N0 |
| TCGA-EW-A1J2 | 403 | 0 | 50 | FEMALE | unknow | unknow | T1c | MX | N1mi |
| TCGA-BH-A0HB | 806 | 0 | 55 | FEMALE | unknow | Stage IA | T1c | M0 | N0 (i-) |
| TCGA-A8-A08R | 30 | 0 | 52 | FEMALE | unknow | Stage IIB | T2 | M0 | N1a |
| TCGA-EW-A1P0 | 1251 | 0 | 55 | FEMALE | unknow | Stage IIB | T2 | MX | N1b |
| TCGA-A7-A5ZW | 326 | 0 | 47 | FEMALE | unknow | Stage IIA | T1c | M0 | N1a |
| TCGA-AR-A0U0 | 1988 | 0 | 73 | FEMALE | unknow | Stage IIB | T2 | M0 | N1 |
| TCGA-D8-A1X8 | 783 | 0 | 62 | FEMALE | unknow | Stage IIIA | T1c | M0 | N2a |
| TCGA-C8-A130 | 370 | 0 | 52 | FEMALE | unknow | Stage IIIA | T3 | M0 | N2 |
| TCGA-E2-A1LH | 3247 | 0 | 59 | FEMALE | unknow | Stage I | T1c | M0 | N0 |
| TCGA-AR-A1AY | 1026 | 0 | 65 | FEMALE | unknow | Stage I | T1 | M0 | N0 |
| TCGA-5L-AAT1 | 1471 | 0 | 63 | FEMALE | unknow | Stage IV | T2 | M1 | N0 |
| TCGA-E9-A22H | 1232 | 0 | 42 | FEMALE | unknow | Stage IIB | T2 | M0 | N1 |
| TCGA-EW-A1J1 | 575 | 0 | 38 | FEMALE | unknow | Stage IIB | T2 | M0 | N1a |
| TCGA-LL-A7T0 | 376 | 0 | 70 | FEMALE | unknow | Stage IIB | T2 | M0 | N1a |
| TCGA-B6-A0IO | 5042 | 0 | 66 | FEMALE | unknow | Stage IIA | T2 | M0 | N0 (i-) |
| TCGA-BH-A28O | 1120 | 0 | 50 | FEMALE | unknow | Stage IIIC | T3a | M0 | N3 |
| TCGA-EW-A6SC | 952 | 0 | 60 | FEMALE | unknow | Stage IIA | T2 | M0 | N0 |
| TCGA-4H-AAAK | 348 | 0 | 50 | FEMALE | unknow | Stage IIIA | T2 | M0 | N2a |
| TCGA-A2-A0EN | 4088 | 0 | 70 | FEMALE | unknow | Stage IIA | T2 | M0 | N0 (i+) |
| TCGA-E2-A1IF | 1138 | 0 | 74 | FEMALE | unknow | Stage I | T1c | M0 | N0 |
| TCGA-A8-A06Y | 791 | 0 | 66 | FEMALE | unknow | Stage IIA | T2 | M0 | N0 |
| TCGA-A2-A0D4 | 767 | 0 | 37 | FEMALE | unknow | Stage IIB | T2 | M0 | N1a |
| TCGA-OL-A5D6 | 1104 | 1 | 71 | FEMALE | unknow | Stage IIA | T2 | MX | N0 |
| TCGA-AC-A62Y | 530 | 0 | 79 | FEMALE | unknow | Stage IIB | T2 | MX | N1 |
| TCGA-EW-A1OZ | 1229 | 0 | 56 | FEMALE | unknow | Stage IIA | T2 | M0 | N0 (i-) |
| TCGA-AC-A62X | 417 | 0 | 72 | FEMALE | unknow | Stage IIA | T2 | MX | N0 |
| TCGA-E2-A156 | 726 | 0 | 61 | FEMALE | unknow | Stage I | T1c | M0 | N0 |
| TCGA-E9-A1R6 | 339 | 0 | 63 | FEMALE | unknow | Stage IIA | T2 | M0 | N0 |
| TCGA-EW-A1J6 | 875 | 0 | 70 | FEMALE | unknow | Stage I | T1c | M0 | N0 (i-) |
| TCGA-E9-A1RA | 1369 | 0 | 48 | FEMALE | unknow | Stage IA | T1c | M0 | N0 |
| TCGA-AR-A24S | 2976 | 0 | 61 | FEMALE | unknow | Stage I | T1 | M0 | N0 |
| TCGA-AN-A0FD | 196 | 0 | 71 | FEMALE | unknow | Stage IIA | T2 | M0 | N0 |
| TCGA-EW-A1PA | 575 | 0 | 59 | FEMALE | unknow | Stage IIB | T2 | M0 | N1a |
| TCGA-PE-A5DE | 2645 | 0 | 41 | FEMALE | unknow | Stage IIA | T2 | M0 | N0 |
| TCGA-C8-A135 | 393 | 0 | 64 | FEMALE | unknow | Stage IIB | T2 | M0 | N1 |
| TCGA-BH-A1F0 | 785 | 1 | 80 | FEMALE | unknow | Stage IIA | T1a | M0 | N1c |
| TCGA-BH-A1EX | 1508 | 1 | 67 | FEMALE | unknow | Stage IIB | T2 | M0 | N1b |
| TCGA-UU-A93S | 116 | 1 | 63 | FEMALE | unknow | Stage IV | T4d | M1 | N3b |
| TCGA-BH-A202 | 795 | 0 | 60 | FEMALE | unknow | Stage II | T2 | M0 | N0 |
| TCGA-AO-A126 | 3307 | 0 | 39 | FEMALE | unknow | Stage IIA | T2 | M0 | N0 (i-) |
| TCGA-AR-A0TU | 709 | 0 | 35 | FEMALE | unknow | Stage IIA | T2 | M0 | N0 |
| TCGA-AO-A0J4 | 1587 | 0 | 41 | FEMALE | unknow | Stage IA | T1c | M0 | N0 (i-) |
| TCGA-A2-A0SX | 1534 | 0 | 48 | FEMALE | unknow | Stage IA | T1c | M0 | N0 (i-) |
| TCGA-BH-A0AU | 1914 | 0 | 45 | FEMALE | unknow | Stage IIA | T2 | M0 | N0 |
| TCGA-A7-A13F | 765 | 0 | 44 | FEMALE | unknow | Stage IIIA | T3 | M0 | N1a |
| TCGA-A2-A04R | 3709 | 0 | 36 | FEMALE | unknow | Stage IA | T1 | M0 | N0 (i-) |
| TCGA-BH-A1FR | 1642 | 1 | 73 | FEMALE | unknow | Stage IIIB | T4b | M0 | N1a |
| TCGA-B6-A0RL | 2469 | 1 | 60 | FEMALE | unknow | Stage IIA | T2 | M0 | N0 (i-) |
| TCGA-EW-A1PE | 320 | 0 | 56 | FEMALE | unknow | Stage IIA | T1c | M0 | N1a |
| TCGA-AO-A0J2 | 997 | 0 | 41 | FEMALE | unknow | Stage IA | T1c | M0 | N0 (i-) |
| TCGA-AO-A0JE | 2335 | 0 | 53 | FEMALE | unknow | Stage IIIA | T2 | M0 | N2a |
| TCGA-BH-A0E1 | 477 | 0 | 52 | FEMALE | unknow | Stage IIB | T2 | M0 | N1 |
| TCGA-C8-A12U | 385 | 0 | 46 | FEMALE | unknow | Stage IIB | T2 | M0 | N1 |
| TCGA-A8-A06U | 883 | 1 | 80 | FEMALE | unknow | Stage IIB | T2 | M0 | N1a |
| TCGA-AR-A1AT | 1272 | 1 | 62 | FEMALE | unknow | Stage IIA | T2 | M0 | N0 |
| TCGA-B6-A0RP | 3126 | 1 | 73 | FEMALE | unknow | unknow | T2 | M0 | N1b |
| TCGA-E2-A15D | 526 | 0 | 47 | FEMALE | unknow | Stage IIA | T2 | M0 | N0 |
| TCGA-E2-A1B4 | 1004 | 1 | 74 | FEMALE | unknow | Stage IIIA | T1c | M0 | N2a |
| TCGA-AC-A2QI | 588 | 0 | 76 | FEMALE | unknow | Stage IIIA | T3 | MX | N1a |
| TCGA-BH-A18G | 149 | 0 | 81 | FEMALE | unknow | Stage IA | T1c | M0 | N0 |
| TCGA-D8-A1JS | 371 | 0 | 77 | FEMALE | unknow | Stage IA | T1c | M0 | N0 |
| TCGA-AO-A124 | 3506 | 0 | 38 | FEMALE | unknow | Stage IIA | T2 | M0 | N0 (i-) |
| TCGA-A2-A0T0 | 533 | 0 | 59 | FEMALE | unknow | Stage IIB | T2 | M0 | N1 |
| TCGA-EW-A2FV | 788 | 0 | 39 | FEMALE | unknow | Stage IIIC | T3 | MX | N3a |
| TCGA-AR-A0TR | 160 | 1 | 68 | FEMALE | unknow | Stage IIB | T2 | M0 | N1 |
| TCGA-AO-A12F | 1842 | 0 | 36 | FEMALE | unknow | Stage IIA | T2 | M0 | N0 (i-) |
| TCGA-GM-A2DN | 3091 | 0 | 58 | FEMALE | unknow | Stage IIA | T2 | M0 | N0 (i-) |
| TCGA-BH-A0E7 | 1363 | 0 | 79 | FEMALE | unknow | Stage IIB | T2 | M0 | N1a |
| TCGA-E9-A1RG | 647 | 0 | 62 | FEMALE | unknow | Stage IIIA | T1c | M0 | N2 |
| TCGA-AR-A24T | 3202 | 0 | 46 | FEMALE | unknow | Stage IIIC | T3 | M0 | N3 |
| TCGA-A7-A26E | 954 | 0 | 71 | FEMALE | unknow | Stage IIIA | T3 | MX | N1a |
| TCGA-D8-A1XQ | 499 | 0 | 69 | FEMALE | unknow | Stage IIA | T2 | M0 | N0 |
| TCGA-D8-A27E | 530 | 0 | 66 | FEMALE | unknow | Stage IA | T1c | M0 | N0 |
| TCGA-E9-A249 | 217 | 0 | 45 | FEMALE | unknow | Stage IIA | T2 | M0 | N0 (i-) |
| TCGA-D8-A146 | 643 | 0 | 57 | FEMALE | unknow | Stage IIA | T2 | MX | N0 |
| TCGA-D8-A1XL | 606 | 0 | 34 | FEMALE | unknow | Stage IIB | T2 | MX | N1a |
| TCGA-A2-A3XY | 1093 | 1 | 49 | FEMALE | unknow | Stage IIB | T2 | M0 | N1a |
| TCGA-E2-A10E | 865 | 0 | 64 | FEMALE | unknow | Stage IIA | T1c | M0 | N1a |
| TCGA-B6-A400 | 215 | 0 | 43 | FEMALE | unknow | Stage IIIA | T2 | M0 | N2a |
| TCGA-AN-A04A | 90 | 0 | 36 | FEMALE | unknow | Stage IIIA | T2 | M0 | N2 |
| TCGA-A7-A0D9 | 1139 | 0 | 37 | FEMALE | unknow | Stage IIA | T2 | M0 | N0 (i-) |
| TCGA-BH-A0BP | 2296 | 1 | 76 | FEMALE | unknow | Stage I | T1c | M0 | N0 |
| TCGA-EW-A1P3 | 1611 | 0 | 48 | FEMALE | unknow | Stage IIA | T2 | MX | N0 |
| TCGA-AO-A0JJ | 1887 | 0 | 54 | FEMALE | unknow | Stage IIB | T2 | M0 | N1a |
| TCGA-BH-A0HU | 392 | 0 | 52 | FEMALE | unknow | Stage IA | T1c | M0 | N0 (i-) |
| TCGA-E2-A14S | 1009 | 0 | 65 | FEMALE | unknow | Stage I | T1c | M0 | N0 |
| TCGA-BH-A0RX | 170 | 0 | 59 | FEMALE | unknow | Stage IIA | T2 | M0 | N0 (i-) |
| TCGA-BH-A1FN | 2192 | 1 | 34 | FEMALE | unknow | Stage IIA | T2 | M0 | N0 |
| TCGA-A2-A3XZ | 1532 | 0 | 46 | FEMALE | unknow | Stage I | T1c | M0 | N0 |
| TCGA-A1-A0SD | 437 | 0 | 59 | FEMALE | unknow | Stage IIA | T2 | M0 | N0 |
| TCGA-A7-A6VX | 317 | 0 | 68 | FEMALE | unknow | Stage IIA | T2 | M0 | N0 |
| TCGA-OL-A66L | 1301 | 0 | 71 | FEMALE | unknow | Stage IA | T1c | MX | N0 |
| TCGA-B6-A0X7 | 1781 | 1 | 62 | FEMALE | unknow | Stage X | T1c | MX | NX |
| TCGA-PL-A8LY | 8 | 0 | 30 | FEMALE | unknow | Stage IIB | T3 | M0 | N0 |
| TCGA-A2-A0EQ | 2426 | 0 | 64 | FEMALE | unknow | Stage IIA | T2 | M0 | N0 (i-) |
| TCGA-AR-A2LK | 1649 | 1 | 62 | FEMALE | unknow | Stage III | T3 | M0 | N2 |
| TCGA-A2-A0EW | 1884 | 1 | 53 | FEMALE | unknow | Stage IIIC | T1b | M0 | N3a |
| TCGA-AO-A0J8 | 680 | 0 | 61 | FEMALE | unknow | Stage IIA | T2 | M0 | N0 (i+) |
| TCGA-E2-A1IH | 1026 | 0 | 80 | FEMALE | unknow | Stage I | T1c | M0 | N0 |
| TCGA-BH-A0BC | 974 | 0 | 60 | FEMALE | unknow | Stage IIIC | T2 | M0 | N3 |
| TCGA-E9-A2JT | 288 | 0 | 63 | FEMALE | unknow | Stage IIA | T2 | M0 | N0 (i-) |
| TCGA-BH-A0B3 | 1203 | 0 | 53 | FEMALE | unknow | Stage IIB | T2 | M0 | N1a |
| TCGA-AC-A2B8 | 677 | 0 | 84 | FEMALE | unknow | Stage IIB | T3 | M0 | N0 (i-) |
| TCGA-A1-A0SE | 1321 | 0 | 56 | FEMALE | unknow | Stage I | T1c | M0 | N0 (i-) |
| TCGA-OL-A66J | 1996 | 0 | 80 | FEMALE | unknow | Stage I | T1c | MX | N0 |
| TCGA-BH-A0B7 | 2559 | 0 | 42 | FEMALE | unknow | Stage IIB | T2 | M0 | N1a |
| TCGA-E9-A3Q9 | 1001 | 0 | 78 | FEMALE | unknow | Stage IIIC | T3 | M0 | N3 |
| TCGA-BH-A0H5 | 1620 | 0 | 45 | FEMALE | unknow | Stage I | T1c | M0 | N0 (i-) |
| TCGA-E2-A1IU | 337 | 0 | 60 | FEMALE | unknow | Stage IA | T1c | M0 | N0 (mol+) |
| TCGA-BH-A1FG | 577 | 1 | 88 | FEMALE | unknow | Stage I | T1c | M0 | NX |
| TCGA-E2-A1B0 | 1631 | 0 | 50 | FEMALE | unknow | Stage IIIA | T2 | M0 | N2 |
| TCGA-C8-A278 | 297 | 0 | 61 | FEMALE | unknow | Stage IIIA | T2 | M0 | N2 |
| TCGA-E2-A107 | 1047 | 0 | 54 | FEMALE | unknow | Stage IIIA | T3 | M0 | N2a |
| TCGA-A1-A0SI | 635 | 0 | 52 | FEMALE | unknow | Stage IIB | T2 | M0 | N1a |
| TCGA-D8-A1JP | 639 | 0 | 73 | FEMALE | unknow | Stage IA | T1c | M0 | N0 |
| TCGA-B6-A0IK | 571 | 1 | 63 | FEMALE | unknow | Stage IIIB | T4 | M0 | N1 |
| TCGA-A7-A0DA | 1085 | 0 | 62 | FEMALE | unknow | Stage IIA | T2 | M0 | N0 (i-) |
| TCGA-A2-A0SV | 825 | 1 | 63 | FEMALE | unknow | Stage IV | T2 | M1 | N2a |
| TCGA-A8-A09D | 1522 | 0 | 47 | FEMALE | unknow | Stage IIB | T2 | M0 | N1 |
| TCGA-BH-A209 | 3959 | 1 | 77 | FEMALE | unknow | Stage I | T1c | M0 | N0 |
| TCGA-BH-A1FM | 1388 | 1 | 44 | FEMALE | unknow | Stage IIIA | T2 | M0 | N2 |
| TCGA-LD-A66U | 646 | 0 | 44 | FEMALE | unknow | Stage IIB | T2 | MX | N1mi |
| TCGA-AO-A0J7 | 618 | 0 | 71 | FEMALE | unknow | Stage IIB | T2 | M0 | N1 |
| TCGA-BH-A0C3 | 2709 | 0 | 47 | FEMALE | unknow | Stage I | T1c | M0 | N0 |
| TCGA-D8-A1JB | 1688 | 0 | 54 | FEMALE | unknow | Stage IIB | T2 | M0 | N1a |
| TCGA-5L-AAT0 | 1477 | 0 | 42 | FEMALE | unknow | Stage IIA | T2 | M0 | N0 |
| TCGA-AO-A1KO | 622 | 0 | 46 | FEMALE | unknow | Stage IIB | T3 | M0 | N0 (i+) |
| TCGA-D8-A1XK | 441 | 0 | 55 | FEMALE | unknow | Stage IIB | T2 | MX | N1a |
| TCGA-A8-A090 | 0 | 0 | 74 | FEMALE | unknow | Stage IIA | T2 | M0 | N0 |
| TCGA-AO-A03V | 1351 | 0 | 41 | FEMALE | unknow | Stage I | T1c | M0 | N0 (i-) |
| TCGA-E2-A159 | 762 | 0 | 50 | FEMALE | unknow | Stage IIA | T2 | M0 | N0 |
| TCGA-A8-A075 | 518 | 0 | 42 | FEMALE | unknow | Stage IIB | T2 | M0 | N1a |
| TCGA-BH-A0DE | 2372 | 0 | 62 | FEMALE | unknow | Stage IIA | T2 | M0 | N0 |
| TCGA-BH-A0C0 | 1270 | 0 | 62 | FEMALE | unknow | Stage IIA | T1c | M0 | N1a |
| TCGA-A7-A0CJ | 931 | 0 | 57 | FEMALE | unknow | Stage IIA | T2 | M0 | N0 (i-) |
| TCGA-BH-A0DK | 423 | 0 | 49 | FEMALE | unknow | Stage IIA | T2 | M0 | N0 (i-) |
| TCGA-E2-A105 | 1308 | 0 | 79 | FEMALE | unknow | Stage IIA | T2 | M0 | N0 (i-) |
| TCGA-B6-A0RO | 4929 | 0 | 71 | FEMALE | unknow | Stage IIIB | T4 | M0 | N1a |
| TCGA-AN-A0FV | 10 | 0 | 58 | FEMALE | unknow | Stage IIA | T2 | M0 | N0 |
| TCGA-AN-A0AM | 5 | 0 | 56 | FEMALE | unknow | Stage IIA | T2 | M0 | N0 |
| TCGA-BH-A8G0 | 662 | 0 | 54 | FEMALE | unknow | Stage IIB | T2 | M0 | N1a |
| TCGA-E2-A154 | 591 | 0 | 68 | FEMALE | unknow | Stage I | T1c | M0 | N0 |
| TCGA-BH-A0C7 | 2767 | 0 | 48 | FEMALE | unknow | Stage IIB | T2 | M0 | N1mi |
| TCGA-EW-A1P1 | 1210 | 0 | 68 | FEMALE | unknow | Stage IIIC | T2 | MX | N3b |
| TCGA-AR-A24W | 1550 | 0 | 55 | FEMALE | unknow | Stage IIB | T2 | M0 | N1 |
| TCGA-BH-A0BG | 1871 | 0 | 73 | FEMALE | unknow | Stage I | T1 | M0 | N0 |
| TCGA-AO-A0JF | 1980 | 0 | 68 | FEMALE | unknow | Stage IIA | T1c | M0 | N1a |
| TCGA-EW-A1PG | 1051 | 0 | 53 | FEMALE | unknow | Stage IIB | T2 | MX | N1a |
| TCGA-AC-A2BM | 3022 | 0 | 41 | FEMALE | unknow | Stage IIB | T2 | MX | N1a |
| TCGA-BH-A18T | 224 | 1 | 70 | FEMALE | unknow | Stage IIA | T2 | M0 | N0 |
| TCGA-BH-A5IZ | 567 | 0 | 51 | FEMALE | unknow | Stage IIB | T2 | cM0 (i+) | N1a |
| TCGA-E2-A15J | 1640 | 0 | 51 | FEMALE | unknow | Stage I | T1c | M0 | N0 |
| TCGA-E2-A1IK | 1800 | 0 | 71 | FEMALE | unknow | Stage IIA | T1c | M0 | N1mi |
| TCGA-A8-A09Z | 0 | 0 | 83 | FEMALE | unknow | Stage IIB | T3 | M0 | N0 |
| TCGA-A8-A0A1 | 365 | 0 | 84 | FEMALE | unknow | Stage IIA | T2 | M0 | N0 |
| TCGA-BH-A0BZ | 2255 | 0 | 59 | FEMALE | unknow | Stage IIIA | T3 | M0 | N1a |
| TCGA-E2-A14N | 1434 | 0 | 37 | FEMALE | unknow | Stage IIB | T2 | M0 | N1 |
| TCGA-A8-A092 | 942 | 0 | 48 | FEMALE | unknow | Stage IIIA | T2 | M0 | N2a |
| TCGA-LL-A440 | 759 | 0 | 61 | FEMALE | unknow | Stage IA | T1c | MX | N0 (i-) |
| TCGA-A2-A3KC | 1102 | 0 | 55 | FEMALE | unknow | Stage IIB | T2 | M0 | N1a |
| TCGA-B6-A0WW | 558 | 1 | 58 | FEMALE | unknow | Stage X | T2 | MX | N1b |
| TCGA-B6-A0WX | 639 | 1 | 40 | FEMALE | unknow | Stage IIIA | T3 | M0 | N1b |
| TCGA-D8-A27F | 488 | 0 | 40 | FEMALE | unknow | Stage IIA | T2 | M0 | N0 |
| TCGA-AN-A04D | 52 | 0 | 58 | FEMALE | unknow | Stage IIB | T2 | M0 | N1 |
| TCGA-AR-A24M | 3660 | 0 | 38 | FEMALE | unknow | Stage IIIA | T2 | M0 | N2 |
| TCGA-A8-A07R | 273 | 0 | 80 | FEMALE | unknow | Stage IIIC | T2 | M0 | N3a |
| TCGA-AR-A5QP | 1185 | 0 | 54 | FEMALE | unknow | Stage IIB | T2 | M0 | N1 |
| TCGA-A8-A06R | 547 | 0 | 69 | FEMALE | unknow | Stage IIB | T2 | M0 | N1a |
| TCGA-A2-A04V | 1920 | 1 | 39 | FEMALE | unknow | Stage IIA | T2 | M0 | N0 (i-) |
| TCGA-OL-A66P | 428 | 0 | 75 | FEMALE | unknow | Stage IIA | T2 | MX | N0 |
| TCGA-A1-A0SP | 584 | 0 | 40 | FEMALE | unknow | Stage IIA | T2 | M0 | N0 (i-) |
| TCGA-BH-AB28 | 287 | 0 | 53 | FEMALE | unknow | Stage IIIA | T3 | M0 | N1a |
| TCGA-AC-A2BK | 2222 | 0 | 78 | FEMALE | unknow | Stage IIIA | T2 | MX | N2a |
| TCGA-AC-A2FK | 2650 | 0 | 45 | FEMALE | unknow | Stage IIIC | T2 | M0 | N3a |
| TCGA-AO-A128 | 3248 | 0 | 61 | FEMALE | unknow | Stage IIA | T2 | M0 | N0 (i-) |
| TCGA-BH-A18S | 2009 | 1 | 79 | FEMALE | unknow | Stage I | T1c | M0 | N0 |
| TCGA-OL-A66N | 792 | 0 | 59 | FEMALE | unknow | Stage IIIC | T3 | MX | N3 |
| TCGA-BH-A0E9 | 2489 | 0 | 53 | FEMALE | unknow | Stage IIB | T2 | M0 | N1a |
| TCGA-AR-A24Z | 3001 | 0 | 57 | FEMALE | unknow | Stage IIA | T2 | M0 | N0 |
| TCGA-D8-A1XY | 503 | 0 | 74 | FEMALE | unknow | Stage IIA | T2 | MX | N0 |
| TCGA-C8-A12M | 358 | 0 | 70 | FEMALE | unknow | Stage IIA | T2 | M0 | N0 (i-) |
| TCGA-BH-A18M | 2207 | 1 | 39 | FEMALE | unknow | Stage IIIA | T3 | M0 | N1 |
| TCGA-B6-A0RM | 2373 | 1 | 57 | FEMALE | unknow | Stage X | TX | MX | NX |
| TCGA-E2-A15F | 658 | 0 | 64 | FEMALE | unknow | Stage I | T1c | M0 | N0 |
| TCGA-A2-A0YH | 659 | 0 | 53 | FEMALE | unknow | Stage IIIA | T2 | M0 | N2a |
| TCGA-B6-A0RG | 2082 | 0 | 26 | FEMALE | unknow | Stage IIB | T3 | M0 | N0 (i-) |
| TCGA-E9-A1NG | 786 | 1 | 62 | FEMALE | unknow | Stage IIA | T2 | M0 | N0 |
| TCGA-B6-A0RV | 5156 | 0 | 42 | FEMALE | unknow | Stage IIIA | T3 | M0 | N2 |
| TCGA-AO-A0J3 | 651 | 0 | 67 | FEMALE | unknow | Stage IIB | T2 | M0 | N1b |
| TCGA-WT-AB41 | 1611 | 0 | 55 | FEMALE | unknow | Stage IIB | T2 | MX | N1mi |
| TCGA-E9-A1NF | 1072 | 1 | 60 | FEMALE | unknow | Stage IIA | T2 | M0 | N0 |
| TCGA-AQ-A04J | 819 | 0 | 45 | FEMALE | unknow | Stage IIA | T2 | M0 | N0 (i+) |
| TCGA-AR-A0TX | 1972 | 0 | 64 | FEMALE | unknow | Stage IIA | T1 | M0 | N1 |
| TCGA-A7-A13G | 718 | 0 | 79 | FEMALE | unknow | Stage IIA | T2 | MX | N0 |
| TCGA-GM-A3XL | 2108 | 0 | 49 | FEMALE | unknow | Stage IIA | T2 | M0 | N0 (i-) |
| TCGA-A7-A6VY | 266 | 0 | 48 | FEMALE | unknow | Stage IIB | T2 | M0 | N1 |
| TCGA-GM-A2DL | 3519 | 0 | 50 | FEMALE | unknow | Stage I | T1c | M0 | N0 (i-) |
| TCGA-E2-A9RU | 0 | 0 | 90 | FEMALE | unknow | Stage IIIC | T2 | MX | N3a |
| TCGA-LL-A73Y | 477 | 0 | 67 | FEMALE | unknow | Stage IA | T1c | MX | N0 |
| TCGA-D8-A1Y3 | 430 | 0 | 61 | FEMALE | unknow | Stage IIIA | T2 | MX | N2a |
| TCGA-B6-A0I6 | 991 | 1 | 49 | FEMALE | unknow | Stage IIA | T1c | M0 | N1 |
| TCGA-AO-A1KR | 2513 | 0 | 51 | FEMALE | unknow | Stage IIA | T2 | M0 | N0 |
| TCGA-EW-A1J5 | 477 | 0 | 59 | FEMALE | unknow | Stage IIB | T3 | M0 | N0 (i-) |
| TCGA-E2-A56Z | 252 | 0 | 69 | FEMALE | unknow | Stage IIB | T2 | M0 | N1a |
| TCGA-AR-A1AH | 3807 | 0 | 51 | FEMALE | unknow | Stage IIB | T2 | M0 | N1 |
| TCGA-BH-A18H | 652 | 0 | 63 | FEMALE | unknow | Stage IA | T1b | M0 | N0 |
| TCGA-AN-A0XP | 9 | 0 | 69 | FEMALE | unknow | Stage IIIA | T2 | M0 | N2 |
| TCGA-C8-A3M7 | 1034 | 1 | 60 | FEMALE | unknow | Stage IIIB | T4b | M0 | N0 |
| TCGA-OL-A66K | 1275 | 1 | 72 | FEMALE | unknow | Stage IIA | T2 | MX | N0 |
| TCGA-AC-A23E | 698 | 0 | 50 | FEMALE | unknow | Stage IIB | T2 | MX | N1 |
| TCGA-A2-A04U | 2654 | 0 | 47 | FEMALE | unknow | Stage IIA | T2 | M0 | N0 (i+) |
| TCGA-A2-A25C | 523 | 0 | 50 | FEMALE | unknow | Stage IIB | T2 | M0 | N1a |
| TCGA-A2-A0YI | 1505 | 0 | 62 | FEMALE | unknow | Stage I | T1c | M0 | N0 (i+) |
| TCGA-A2-A0CO | 1468 | 0 | 85 | FEMALE | unknow | Stage IIB | T3 | M0 | N0 |
| TCGA-GM-A3NY | 1162 | 0 | 72 | FEMALE | unknow | Stage IIB | T2 | M0 | N1a |
| TCGA-BH-A18R | 1142 | 1 | 50 | FEMALE | unknow | Stage IIA | T2b | M0 | N1 |
| TCGA-AN-A0XN | 10 | 0 | 68 | FEMALE | unknow | Stage IIIA | T2 | M0 | N2 |
| TCGA-D8-A27K | 1461 | 0 | 47 | FEMALE | unknow | Stage IIB | T2 | M0 | N1a |
| TCGA-LL-A5YO | 440 | 0 | 50 | FEMALE | unknow | Stage IA | T1b | MX | N0 |
| TCGA-S3-AA12 | 574 | 0 | 82 | FEMALE | unknow | Stage IIIA | T3 | MX | N1a |
| TCGA-E2-A15I | 1692 | 0 | 44 | FEMALE | unknow | Stage IIA | T2 | M0 | N0 |
| TCGA-BH-A1EO | 2798 | 1 | 68 | FEMALE | unknow | Stage IIA | T1c | M0 | N1 |
| TCGA-BH-A0C1 | 1411 | 1 | 61 | FEMALE | unknow | Stage IIIA | T3 | M0 | N2a |
| TCGA-C8-A12Y | 1476 | 0 | 44 | FEMALE | unknow | Stage IIB | T2 | M0 | N1 |
| TCGA-AO-A03R | 2091 | 0 | 57 | FEMALE | unknow | Stage IIB | T2 | M0 | N1a |
| TCGA-D8-A27R | 307 | 0 | 41 | FEMALE | unknow | Stage IIIC | T2 | M0 | N3a |
| TCGA-D8-A142 | 425 | 0 | 74 | FEMALE | unknow | Stage IIB | T3 | M0 | N0 |
| TCGA-AQ-A04L | 3957 | 0 | 48 | FEMALE | unknow | Stage IIA | T2 | MX | N0 (i-) |
| TCGA-E2-A14V | 1042 | 0 | 53 | FEMALE | unknow | Stage IIB | T2 | M0 | N1 |
| TCGA-A7-A13D | 965 | 0 | 46 | FEMALE | unknow | Stage IIA | T2 | M0 | N0 (i-) |
| TCGA-A2-A0CK | 4159 | 0 | 60 | FEMALE | unknow | Stage IIIA | T3 | M0 | N2a |
| TCGA-E9-A1R5 | 92 | 0 | 63 | FEMALE | unknow | Stage IA | T1c | M0 | N0 |
| TCGA-AC-A8OP | 614 | 0 | 72 | FEMALE | unknow | Stage IA | T1c | MX | N0 (i-) |
| TCGA-OL-A6VR | 1220 | 0 | 48 | FEMALE | unknow | Stage IA | T1b | MX | N0 |
| TCGA-AC-A3OD | 451 | 0 | 68 | FEMALE | unknow | Stage IIB | T2 | MX | N1mi |
| TCGA-B6-A0RN | 8008 | 0 | 60 | FEMALE | unknow | Stage IA | T1c | M0 | N0 (i-) |
| TCGA-AC-A3TM | 762 | 0 | 50 | FEMALE | unknow | Stage IIIA | T3 | M0 | N1mi |
| TCGA-B6-A0RU | 8605 | 0 | 40 | FEMALE | unknow | Stage IA | T1c | M0 | N0 (i-) |
| TCGA-AR-A2LN | 1161 | 0 | 65 | FEMALE | unknow | Stage IIA | T2 | M0 | N0 |
| TCGA-BH-A0HA | 1611 | 0 | 31 | FEMALE | unknow | Stage I | T1c | M0 | N0 (i-) |
| TCGA-A2-A25F | 322 | 0 | 66 | FEMALE | unknow | Stage IIA | T2 | M0 | N0 (i-) |
| TCGA-BH-A1FH | 1034 | 1 | 47 | FEMALE | unknow | Stage IV | T2 | M1 | N1b |
| TCGA-C8-A134 | 383 | 0 | 52 | FEMALE | unknow | Stage IIA | T2 | M0 | N0 |
| TCGA-A1-A0SO | 852 | 0 | 67 | FEMALE | unknow | Stage IIB | T2 | M0 | N1 |
| TCGA-AR-A24K | 1548 | 0 | 46 | FEMALE | unknow | Stage IIA | T2 | M0 | N0 |
| TCGA-AR-A1AP | 2856 | 0 | 80 | FEMALE | unknow | Stage I | T1 | M0 | N0 |
| TCGA-AN-A0AR | 10 | 0 | 55 | FEMALE | unknow | Stage IIA | T2 | M0 | N0 |
| TCGA-E2-A1LS | 1604 | 0 | 46 | FEMALE | unknow | Stage IA | T1c | M0 | N0 |
| TCGA-B6-A0IB | 3941 | 1 | 64 | FEMALE | unknow | Stage IV | T3 | M1 | N3 |
| TCGA-A2-A1G1 | 584 | 0 | 85 | FEMALE | unknow | Stage IIB | T2 | M0 | N1 |
| TCGA-E9-A1NA | 1112 | 0 | 58 | FEMALE | unknow | Stage IIA | T2 | M0 | N0 |
| TCGA-A2-A0D0 | 2048 | 0 | 60 | FEMALE | unknow | Stage IIA | T2 | M0 | N0 (i-) |
| TCGA-AC-A2FG | 1853 | 0 | 79 | FEMALE | unknow | Stage IIB | T2 | MX | N1a |
| TCGA-E2-A1IJ | 865 | 0 | 57 | FEMALE | unknow | Stage I | T1c | M0 | N0 |
| TCGA-AC-A5EH | 511 | 0 | 76 | FEMALE | unknow | Stage IIB | T2 | MX | N1mi |
| TCGA-BH-A1F8 | 1 | 1 | 90 | FEMALE | unknow | Stage IIIB | T4b | M0 | N2 |
| TCGA-D8-A1JD | 552 | 0 | 41 | FEMALE | unknow | Stage IIB | T2 | M0 | N1a |
| TCGA-A2-A3XX | 1439 | 1 | 49 | FEMALE | unknow | Stage IIA | T2 | M0 | N0 |
| TCGA-E2-A15M | 336 | 1 | 66 | FEMALE | unknow | Stage IIA | T2 | M0 | N0 |
| TCGA-A2-A4RW | 222 | 0 | 49 | FEMALE | unknow | Stage IIIC | T2 | M0 | N3a |
| TCGA-AN-A0AK | 224 | 0 | 76 | FEMALE | unknow | Stage IIA | T2 | M0 | N0 |
| TCGA-AR-A5QN | 1013 | 0 | 68 | FEMALE | unknow | Stage IIIC | T2 | M0 | N3 |
| TCGA-A8-A0A6 | 640 | 0 | 64 | FEMALE | unknow | Stage IIIC | T2 | M0 | N3a |
| TCGA-BH-A0B5 | 2136 | 0 | 40 | FEMALE | unknow | Stage IIIA | T2 | M0 | N2a |
| TCGA-E2-A14T | 2311 | 0 | 52 | FEMALE | unknow | Stage IIA | T2 | M0 | N0 |
| TCGA-B6-A1KF | 3088 | 0 | 68 | FEMALE | unknow | Stage IIB | T2 | M0 | N1 |
| TCGA-E9-A3QA | 918 | 0 | 33 | FEMALE | unknow | Stage IIA | T2 | M0 | N0 (i-) |
| TCGA-Z7-A8R5 | 3287 | 0 | 61 | FEMALE | unknow | Stage IIIA | T3 | MX | N1a |
| TCGA-B6-A1KC | 1326 | 0 | 67 | FEMALE | unknow | Stage IIB | T2 | M0 | N1 |
| TCGA-AN-A0AS | 10 | 0 | 70 | FEMALE | unknow | Stage IIIA | T2 | M0 | N2 |
| TCGA-A2-A25D | 0 | 0 | 90 | FEMALE | unknow | Stage IIIC | T2 | M0 | N3a |
| TCGA-A8-A07S | 243 | 0 | 73 | FEMALE | unknow | Stage IIA | T2 | M0 | N0 |
| TCGA-C8-A138 | 380 | 0 | 54 | FEMALE | unknow | Stage IIIA | T2 | M0 | N2 |
| TCGA-AR-A24X | 3004 | 0 | 52 | FEMALE | unknow | Stage IIA | T2 | M0 | N0 |
| TCGA-EW-A1P7 | 915 | 0 | 59 | FEMALE | unknow | Stage IIA | T2 | M0 | N0 (i-) |
| TCGA-A2-A0CP | 2813 | 0 | 60 | FEMALE | unknow | Stage IA | T1c | M0 | N0 (i-) |
| TCGA-C8-A1HI | 343 | 0 | 40 | FEMALE | unknow | Stage IIIA | T2 | M0 | N2 |
| TCGA-A8-A091 | 1004 | 0 | 61 | FEMALE | unknow | Stage IIA | T2 | M0 | N0 |
| TCGA-E2-A14Q | 1163 | 0 | 50 | FEMALE | unknow | Stage IIB | T2 | M0 | N1mi |
| TCGA-BH-A8FZ | 574 | 0 | 58 | FEMALE | unknow | Stage IA | T1b | M0 | N0 |
| TCGA-AO-A1KP | 2953 | 0 | 77 | FEMALE | unknow | Stage IIA | T1 | M0 | N1 |
| TCGA-E2-A1L9 | 598 | 0 | 40 | FEMALE | unknow | Stage IIA | T1c | M0 | N1mi |
| TCGA-AN-A0FL | 231 | 0 | 62 | FEMALE | unknow | Stage IIA | T2 | M0 | N0 |
| TCGA-AN-A0AT | 10 | 0 | 62 | FEMALE | unknow | Stage IIA | T2 | M0 | N0 |
| TCGA-GM-A2DC | 2535 | 0 | 57 | FEMALE | unknow | Stage IIA | T1c | M0 | N1a |
| TCGA-BH-A1EN | 2127 | 1 | 78 | FEMALE | unknow | Stage IIA | T2 | M0 | NX |
| TCGA-BH-A0BF | 1324 | 1 | 56 | FEMALE | unknow | Stage IIB | T2 | M0 | N1mi |
| TCGA-A7-A0CH | 1079 | 0 | 79 | FEMALE | unknow | Stage IIA | T2 | M0 | N0 (i-) |
| TCGA-D8-A1Y1 | 302 | 1 | 80 | FEMALE | unknow | Stage IIIA | T3 | MX | N1a |
| TCGA-AO-A0J5 | 792 | 1 | 48 | FEMALE | unknow | Stage IV | T4 | M1 | N1a |
| TCGA-A2-A0EU | 1043 | 0 | 79 | FEMALE | unknow | Stage IA | T1c | M0 | N0 (i-) |
| TCGA-E2-A106 | 2541 | 0 | 34 | FEMALE | unknow | Stage IB | T1c | M0 | N1mi |
| TCGA-E2-A108 | 837 | 0 | 64 | FEMALE | unknow | Stage IIIA | T2 | M0 | N2a |
| TCGA-E2-A1II | 1025 | 0 | 51 | FEMALE | unknow | Stage I | T1c | M0 | N0 |
| TCGA-C8-A8HR | 408 | 0 | 49 | FEMALE | unknow | Stage IIIA | T3 | M0 | N2 |
| TCGA-E9-A247 | 1186 | 0 | 59 | FEMALE | unknow | Stage IA | T1c | M0 | N0 (i-) |
| TCGA-BH-A0HF | 727 | 0 | 77 | FEMALE | unknow | Stage IA | T1c | M0 | N0 (i-) |
| TCGA-D8-A1XZ | 466 | 0 | 81 | FEMALE | unknow | Stage IIIA | T1c | MX | N2a |
| TCGA-A2-A3Y0 | 1546 | 0 | 57 | FEMALE | unknow | Stage IIB | T2 | M0 | N1a |
| TCGA-BH-A204 | 2534 | 1 | 80 | FEMALE | unknow | Stage IIB | T2 | M0 | N1b |
| TCGA-D8-A1XT | 506 | 0 | 61 | FEMALE | unknow | Stage IIA | T1c | M0 | N1a |
| TCGA-BH-A0BS | 2612 | 0 | 55 | FEMALE | unknow | Stage IIIA | T3 | M0 | N1c |
| TCGA-C8-A26W | 381 | 0 | 58 | FEMALE | unknow | Stage IIB | T2 | M0 | N1 |
| TCGA-A8-A08T | 3409 | 1 | 64 | FEMALE | unknow | Stage IV | T2 | M1 | N1a |
| TCGA-AO-A0JL | 1683 | 0 | 59 | FEMALE | unknow | Stage IIIA | T2 | M0 | N2a |
| TCGA-A8-A0A9 | 822 | 0 | 80 | FEMALE | unknow | Stage IIA | T2 | M0 | N0 |
| TCGA-AR-A24R | 3430 | 0 | 45 | FEMALE | unknow | Stage IIIA | T1 | M0 | N2 |
| TCGA-AR-A24V | 3203 | 0 | 52 | FEMALE | unknow | Stage IIB | T2 | M0 | N1 |
| TCGA-E2-A1LE | 879 | 1 | 71 | FEMALE | unknow | Stage IIIC | T2 | M0 | N3a |
| TCGA-A2-A3XT | 2770 | 0 | 45 | FEMALE | unknow | Stage IIB | T2 | M0 | N1a |
| TCGA-A2-A0EM | 3094 | 0 | 73 | FEMALE | unknow | Stage IA | T1 | M0 | N0 (i-) |
| TCGA-BH-A1ES | 3462 | 1 | 35 | FEMALE | unknow | Stage IIB | T2 | M0 | N1a |
| TCGA-A8-A07Z | 1371 | 0 | 85 | FEMALE | unknow | Stage IIA | T2 | M0 | N0 |
| TCGA-AO-A0JG | 798 | 0 | 49 | FEMALE | unknow | Stage IIIA | T2 | M0 | N2a |
| TCGA-D8-A1JC | 480 | 0 | 59 | FEMALE | unknow | Stage IIIA | T2 | M0 | N2a |
| TCGA-D8-A1XB | 552 | 0 | 62 | FEMALE | unknow | Stage IIB | T2 | M0 | N1a |
| TCGA-BH-A0E6 | 293 | 0 | 69 | FEMALE | unknow | Stage IA | T1c | M0 | N0 (i-) |
| TCGA-E2-A2P5 | 821 | 1 | 78 | FEMALE | unknow | Stage IIIC | T2 | M0 | N3 |
| TCGA-EW-A1P4 | 907 | 0 | 43 | FEMALE | unknow | Stage IIA | T2 | M0 | N0 (i-) |
| TCGA-BH-A208 | 1759 | 1 | 48 | FEMALE | unknow | Stage IIB | T2 | M0 | N1b |
| TCGA-EW-A2FS | 1604 | 0 | 41 | FEMALE | unknow | Stage IIB | T2 | MX | N1mi |
| TCGA-A7-A2KD | 679 | 0 | 53 | FEMALE | unknow | Stage IIIA | T2 | MX | N2a |
| TCGA-BH-A0DG | 2041 | 0 | 30 | FEMALE | unknow | Stage IIA | T2 | M0 | N0 |
| TCGA-E9-A3HO | 1158 | 0 | 49 | FEMALE | unknow | Stage IIA | T2 | M0 | N0 (i-) |
| TCGA-AR-A0TS | 2558 | 0 | 46 | FEMALE | unknow | Stage IIB | T2 | M0 | N1 |
| TCGA-E9-A1NI | 300 | 0 | 51 | FEMALE | unknow | Stage IIA | T2 | M0 | N0 |
| TCGA-A2-A25B | 1291 | 0 | 39 | FEMALE | unknow | Stage IIB | T2 | M0 | N1 |
| TCGA-BH-A0HW | 1561 | 0 | 62 | FEMALE | unknow | Stage IA | T1c | M0 | N0 (i-) |
| TCGA-BH-A0H3 | 1928 | 0 | 46 | FEMALE | unknow | Stage I | T1c | M0 | N0 |
| TCGA-OL-A6VQ | 600 | 0 | 49 | FEMALE | unknow | Stage IIA | T2 | MX | N0 |
| TCGA-AC-A3QQ | 734 | 0 | 54 | FEMALE | unknow | Stage IA | T1c | MX | N0 (i-) |
| TCGA-BH-A5J0 | 715 | 0 | 63 | FEMALE | unknow | Stage IA | T1c | M0 | N0 |
| TCGA-D8-A27L | 499 | 0 | 49 | FEMALE | unknow | Stage IIIA | T1c | M0 | N2a |
| TCGA-A7-A4SF | 545 | 0 | 54 | FEMALE | unknow | Stage IIA | T2 | M0 | N0 |
| TCGA-A2-A0YJ | 566 | 0 | 39 | FEMALE | unknow | Stage IIIA | T3 | M0 | N2a |
| TCGA-D8-A141 | 626 | 0 | 40 | FEMALE | unknow | unknow | T1c | M0 | N1 |
| TCGA-AC-A6IV | 568 | 0 | 47 | FEMALE | unknow | Stage IIB | T2 | MX | N1 |
| TCGA-EW-A1IX | 1208 | 0 | 48 | FEMALE | unknow | Stage IIA | T1b | MX | N1a |
| TCGA-A2-A0EV | 968 | 0 | 80 | FEMALE | unknow | Stage IA | T1c | M0 | N0 (i-) |
| TCGA-B6-A0WZ | 6292 | 0 | 50 | FEMALE | unknow | Stage II | T2 | M0 | N1b |
| TCGA-PL-A8LX | 5 | 0 | 35 | FEMALE | unknow | Stage IV | T4 | M1 | N1a |
| TCGA-C8-A1HF | 332 | 0 | 48 | FEMALE | unknow | Stage IIA | T2 | M0 | N0 |
| TCGA-AQ-A7U7 | 584 | 1 | 55 | FEMALE | unknow | Stage IIIA | T2 | M0 | N2a |
| TCGA-A8-A07J | 365 | 0 | 35 | FEMALE | unknow | Stage IIB | T2 | M0 | N1a |
| TCGA-PL-A8LV | -7 | 0 | 54 | FEMALE | unknow | Stage IIIB | T4 | M0 | N0 |
| TCGA-A2-A0CV | 3011 | 0 | 41 | FEMALE | unknow | Stage IIB | T2 | M0 | N1a |
| TCGA-AO-A12D | 2515 | 0 | 43 | FEMALE | unknow | Stage IIA | T1c | M0 | N1a |
| TCGA-B6-A40C | 2164 | 0 | 51 | FEMALE | unknow | Stage IIB | T3 | M0 | N0 (i-) |
| TCGA-E9-A1N9 | 1101 | 0 | 58 | FEMALE | unknow | Stage IIA | T2 | M0 | N0 |
| TCGA-A8-A06N | 0 | 0 | 66 | FEMALE | unknow | Stage IIIB | T4b | M0 | N0 |
| TCGA-S3-AA15 | 525 | 0 | 51 | FEMALE | unknow | Stage IIB | T2 | cM0 (i+) | N1a |
| TCGA-A2-A0D2 | 1027 | 0 | 45 | FEMALE | unknow | Stage IIA | T2 | M0 | N0 (i+) |
| TCGA-LL-A9Q3 | 532 | 0 | 69 | FEMALE | unknow | Stage IIIC | T3 | MX | N3a |
| TCGA-A8-A06O | 396 | 0 | 60 | FEMALE | unknow | Stage I | T1c | M0 | N0 |
| TCGA-S3-AA11 | 421 | 0 | 67 | FEMALE | unknow | Stage IIA | T2 | M0 | N0 |
| TCGA-AQ-A1H2 | 475 | 0 | 84 | FEMALE | unknow | Stage IIIA | T2 | MX | N2a |
| TCGA-E9-A2JS | 904 | 1 | 72 | FEMALE | unknow | Stage IIB | T2 | M0 | N1 |
| TCGA-E2-A1L6 | 1648 | 0 | 44 | FEMALE | unknow | Stage IIA | T1c | M0 | N1mi |
| TCGA-B6-A0IA | 8391 | 0 | 51 | FEMALE | unknow | Stage IIA | T2 | M0 | N0 (i-) |
| TCGA-A2-A0YG | 666 | 0 | 63 | FEMALE | unknow | Stage IIIC | T2 | M0 | N3a |
| TCGA-B6-A0IQ | 4285 | 0 | 40 | FEMALE | unknow | Stage IIIA | T3 | M0 | N1b |
| TCGA-AC-A4ZE | 890 | 0 | 63 | FEMALE | unknow | Stage IIB | T3 | M0 | N0 |
| TCGA-D8-A13Z | 635 | 0 | 51 | FEMALE | unknow | unknow | T2 | M0 | N2a |
| TCGA-B6-A0IE | 1993 | 1 | 38 | FEMALE | unknow | Stage IIIA | T3 | M0 | N1b |
| TCGA-A2-A04Q | 2385 | 0 | 48 | FEMALE | unknow | Stage IA | T1 | M0 | N0 (i-) |
| TCGA-OL-A5RX | 878 | 0 | 51 | FEMALE | unknow | Stage IA | T1c | MX | N0 |
| TCGA-C8-A26Z | 470 | 0 | 59 | FEMALE | unknow | Stage IIA | T2 | M0 | N0 |
| TCGA-A8-A07I | 426 | 0 | 69 | FEMALE | unknow | Stage IIIA | T2 | M0 | N2a |
| TCGA-E9-A3X8 | 926 | 0 | 48 | FEMALE | unknow | Stage IIB | T2 | M0 | N1 |
| TCGA-AR-A1AW | 2632 | 0 | 65 | FEMALE | unknow | Stage IIA | T2 | M0 | N0 |
| TCGA-C8-A1HK | 366 | 0 | 53 | FEMALE | unknow | Stage IIB | T2 | M0 | N1 |
| TCGA-LL-A5YL | 519 | 0 | 64 | FEMALE | unknow | Stage IIB | T2 | MX | N1a |
| TCGA-EW-A1PB | 608 | 0 | 70 | FEMALE | unknow | Stage IIIA | T3 | MX | N1a |
| TCGA-HN-A2NL | 79 | 0 | 56 | FEMALE | unknow | Stage IIA | T2 | M0 | N0 |
| TCGA-A2-A0T2 | 255 | 1 | 66 | FEMALE | unknow | Stage IV | T3 | M1 | N3 |
| TCGA-LD-A7W5 | 216 | 0 | 52 | FEMALE | unknow | Stage IIIC | T2 | M0 | N3a |
| TCGA-AO-A12H | 1234 | 0 | 69 | FEMALE | unknow | Stage IIA | T2 | M0 | N0 (i-) |
| TCGA-B6-A0RI | 7126 | 0 | 44 | FEMALE | unknow | Stage IIIB | T4b | M0 | N1b |
| TCGA-B6-A0RT | 2721 | 0 | 39 | FEMALE | unknow | Stage IIIA | T3 | M0 | N1 |
| TCGA-A2-A3XS | 1032 | 1 | 62 | FEMALE | unknow | Stage IIIA | T1 | M0 | N2a |
| TCGA-AN-A0FN | 218 | 0 | 61 | FEMALE | unknow | Stage IA | T1c | M0 | N0 |
| TCGA-E2-A15T | 1563 | 0 | 65 | FEMALE | unknow | Stage IIA | T2 | M0 | N0 |
| TCGA-BH-A0HY | 1545 | 0 | 60 | FEMALE | unknow | Stage I | T1c | M0 | N0 |
| TCGA-BH-A0DX | 2156 | 0 | 62 | FEMALE | unknow | Stage I | T1b | M0 | N0 |
| TCGA-BH-A1EV | 365 | 1 | 45 | FEMALE | unknow | Stage IIIA | T3 | M0 | N1b |
| TCGA-AO-A03P | 2911 | 1 | 54 | FEMALE | unknow | Stage IIB | T2 | M0 | N1 |
| TCGA-S3-AA0Z | 629 | 0 | 63 | FEMALE | unknow | Stage IIB | T2 | M0 | N1a |
| TCGA-LL-A6FP | 0 | 0 | 90 | FEMALE | unknow | Stage IIA | T2 | MX | NX |
| TCGA-BH-A0W3 | 728 | 0 | 58 | FEMALE | unknow | Stage IIA | T1c | M0 | N1a |
| TCGA-AN-A0FX | 10 | 0 | 52 | FEMALE | unknow | Stage IIA | T2 | M0 | N0 |
| TCGA-A7-A56D | 448 | 0 | 84 | FEMALE | unknow | Stage IIA | T2 | M0 | N0 |
| TCGA-BH-A0DI | 912 | 0 | 63 | FEMALE | unknow | Stage IIB | T2 | M0 | N1a |
| TCGA-A8-A08X | 1308 | 0 | 43 | FEMALE | unknow | Stage IIIC | T4d | M0 | N3a |
| TCGA-D8-A73U | 492 | 0 | 88 | FEMALE | unknow | Stage IIA | T2 | M0 | N0 |
| TCGA-A1-A0SJ | 416 | 0 | 39 | FEMALE | unknow | Stage IIIA | T3 | M0 | N1a |
| TCGA-A2-A1FV | 714 | 0 | 74 | FEMALE | unknow | Stage IIB | T3 | M0 | N0 (i+) |
| TCGA-C8-A1HJ | 5 | 0 | 53 | FEMALE | unknow | Stage IIA | T2 | M0 | N0 |
| TCGA-E9-A228 | 1285 | 0 | 58 | FEMALE | unknow | Stage IIB | T2 | M0 | N1 |
| TCGA-JL-A3YX | 352 | 0 | 46 | FEMALE | unknow | Stage IIA | T2 | M0 | N0 |
| TCGA-D8-A145 | 410 | 0 | 80 | FEMALE | unknow | Stage IIA | T1c | M0 | N1a |
| TCGA-BH-A18I | 1093 | 0 | 53 | FEMALE | unknow | Stage IIA | T1c | cM0 (i+) | N1a |
| TCGA-E9-A1RE | 1419 | 0 | 74 | FEMALE | unknow | Stage IIIA | T2 | M0 | N2 |
| TCGA-A7-A426 | 364 | 0 | 50 | FEMALE | unknow | Stage IIIC | T3 | MX | N3a |
| TCGA-AO-A0JC | 1547 | 0 | 64 | FEMALE | unknow | Stage IIA | T2 | M0 | N0 (i-) |
| TCGA-AR-A1AQ | 3021 | 0 | 49 | FEMALE | unknow | Stage IIA | T2 | M0 | N0 |
| TCGA-BH-A203 | 1174 | 1 | 78 | FEMALE | unknow | Stage IIB | T2 | M0 | N1b |
| TCGA-D8-A1XW | 1309 | 0 | 53 | FEMALE | unknow | Stage IIA | T2 | M0 | N0 |
| TCGA-AC-A3W5 | 504 | 0 | 65 | FEMALE | unknow | Stage IIA | T2 | MX | N0 |
| TCGA-E2-A1LA | 748 | 0 | 59 | FEMALE | unknow | Stage IIA | T1c | M0 | N1a |
| TCGA-E2-A15G | 554 | 0 | 76 | FEMALE | unknow | Stage IIA | T2 | M0 | N0 |
| TCGA-B6-A1KN | 4233 | 0 | 57 | FEMALE | unknow | Stage IIIB | T4b | M0 | N1 |
| TCGA-E2-A1IE | 2362 | 0 | 61 | FEMALE | unknow | Stage IIB | T2 | M0 | N1 |
| TCGA-A2-A0CQ | 2695 | 0 | 62 | FEMALE | unknow | Stage IA | T1 | M0 | N0 (i-) |
| TCGA-AN-A0XV | 162 | 0 | 67 | FEMALE | unknow | Stage IIIA | T2 | M0 | N2 |
| TCGA-BH-A0BT | 2365 | 0 | 56 | FEMALE | unknow | Stage IIA | T1c | M0 | N1mi |
| TCGA-OL-A5RV | 1062 | 0 | 43 | FEMALE | unknow | Stage IIIC | T2 | MX | N3 |
| TCGA-B6-A0I1 | 2361 | 1 | 73 | FEMALE | unknow | Stage IIA | T2 | M0 | N0 |
| TCGA-BH-A1F6 | 2965 | 1 | 51 | FEMALE | unknow | unknow | T4d | MX | N2a |
| TCGA-A2-A0YC | 990 | 0 | 59 | FEMALE | unknow | Stage IIB | T2 | M0 | N1mi |
| TCGA-E2-A155 | 640 | 0 | 58 | FEMALE | unknow | Stage IIB | T2 | M0 | N1a |
| TCGA-AO-A1KS | 350 | 0 | 69 | FEMALE | unknow | Stage IIA | T2 | M0 | N0 (i-) |
| TCGA-EW-A423 | 533 | 0 | 75 | FEMALE | unknow | Stage IIA | T2 | MX | N0 |
| TCGA-A2-A0CS | 2348 | 1 | 73 | FEMALE | unknow | Stage IV | T4b | M1 | N3a |
| TCGA-D8-A1X9 | 727 | 0 | 66 | FEMALE | unknow | Stage IIB | T2 | M0 | N1a |
| TCGA-AR-A0TP | 4275 | 0 | 43 | FEMALE | unknow | Stage IIA | T2 | M0 | N0 |
| TCGA-3C-AALI | 4005 | 0 | 50 | FEMALE | unknow | Stage IIB | T2 | M0 | N1a |
| TCGA-E2-A2P6 | 1051 | 0 | 77 | FEMALE | unknow | Stage IIB | T2 | M0 | N1 |
| TCGA-E2-A158 | 450 | 0 | 43 | FEMALE | unknow | Stage IIA | T1c | M0 | N1mi |
| TCGA-S3-AA14 | 529 | 0 | 47 | FEMALE | unknow | Stage I | T1 | cM0 (i+) | N0 (i+) |
| TCGA-AR-A1AI | 3296 | 0 | 47 | FEMALE | unknow | Stage IIA | T2 | M0 | N0 |
| TCGA-GM-A3NW | 3361 | 0 | 63 | FEMALE | unknow | Stage IIA | T2 | M0 | N0 (i-) |
| TCGA-A8-A07L | 975 | 0 | 58 | FEMALE | unknow | Stage IIIA | T3 | M0 | N1a |
| TCGA-LL-A5YM | 394 | 0 | 88 | FEMALE | unknow | Stage IIIB | T4b | M0 | NX |
| TCGA-LQ-A4E4 | 849 | 0 | 73 | FEMALE | unknow | Stage IIIA | T3 | MX | N1a |
| TCGA-A7-A4SA | 454 | 0 | 40 | FEMALE | unknow | Stage IIB | T2 | M0 | N1a |
| TCGA-BH-A0BV | 1519 | 0 | 78 | FEMALE | unknow | Stage IIB | T2 | M0 | N1a |
| TCGA-B6-A0WY | 3461 | 1 | 40 | FEMALE | unknow | Stage IIIA | T3 | M0 | N1b |
| TCGA-BH-A201 | 856 | 0 | 64 | FEMALE | unknow | Stage IA | T1 | M0 | N0 |
| TCGA-E2-A572 | 1208 | 0 | 72 | FEMALE | unknow | Stage IIIA | T2 | M0 | N2 |
| TCGA-A8-A09M | 1006 | 0 | 75 | FEMALE | unknow | Stage IIIC | T2 | M0 | N3a |
| TCGA-BH-A0DP | 476 | 0 | 60 | FEMALE | unknow | Stage IIB | T3 | M0 | N0 (i-) |
| TCGA-AR-A2LE | 5062 | 0 | 69 | FEMALE | unknow | Stage I | T1 | M0 | N0 |
| TCGA-OL-A5D8 | 973 | 0 | 40 | FEMALE | unknow | Stage IIB | T3 | MX | N0 |
| TCGA-E9-A1NH | 576 | 0 | 71 | FEMALE | unknow | Stage IIB | T2 | M0 | N1 |
| TCGA-BH-A0W7 | 1363 | 0 | 49 | FEMALE | unknow | Stage I | T1c | M0 | N0 (i-) |
| TCGA-A1-A0SF | 1463 | 0 | 54 | FEMALE | unknow | Stage IIA | T2 | M0 | N0 |
| TCGA-A8-A09R | 273 | 0 | 82 | FEMALE | unknow | Stage IIB | T2 | M0 | N1 |
| TCGA-A8-A06Z | 31 | 0 | 84 | FEMALE | unknow | Stage IIB | T3 | M0 | N0 |
| TCGA-A8-A09G | 0 | 0 | 79 | FEMALE | unknow | Stage IIIC | T3 | M0 | N3a |
| TCGA-A2-A04T | 2246 | 0 | 62 | FEMALE | unknow | Stage IIA | T2 | M0 | N0 (i-) |
| TCGA-D8-A27I | 439 | 0 | 58 | FEMALE | unknow | Stage IIIA | T1c | M0 | N2a |
| TCGA-A2-A0SW | 1365 | 1 | 82 | FEMALE | unknow | Stage IV | T2 | M1 | N2 |
| TCGA-BH-A18U | 1563 | 1 | 72 | FEMALE | unknow | Stage IIIA | T2 | M0 | N2a |
| TCGA-EW-A1PF | 439 | 0 | 50 | FEMALE | unknow | Stage IA | T1c | M0 | N0 |
| TCGA-LD-A7W6 | 404 | 0 | 54 | FEMALE | unknow | Stage IIB | T2 | M0 | N1a |
| TCGA-BH-A0E0 | 134 | 0 | 38 | FEMALE | unknow | Stage IIIC | T3 | M0 | N3a |
| TCGA-D8-A1XJ | 664 | 0 | 76 | FEMALE | unknow | Stage IIIA | T3 | MX | N1a |
| TCGA-A7-A26I | 661 | 0 | 65 | FEMALE | unknow | Stage IIA | T2 | M0 | N0 |
| TCGA-A2-A04X | 1686 | 0 | 34 | FEMALE | unknow | Stage IIA | T2 | M0 | N0 (i-) |
| TCGA-AN-A0XU | 10 | 0 | 54 | FEMALE | unknow | Stage IIA | T2 | M0 | N0 |
| TCGA-A8-A07O | 304 | 0 | 51 | FEMALE | unknow | Stage IIA | T2 | M0 | N0 |
| TCGA-D8-A147 | 584 | 0 | 45 | FEMALE | unknow | unknow | T2 | M0 | N0 |
| TCGA-E2-A10C | 1220 | 0 | 54 | FEMALE | unknow | Stage IIB | T2 | M0 | N1a |
| TCGA-EW-A1P6 | 562 | 0 | 64 | FEMALE | unknow | Stage IIB | T2 | M0 | N1a |
| TCGA-C8-A275 | 1 | 0 | 56 | FEMALE | unknow | Stage IIA | T1 | M0 | N1 |
| TCGA-A8-A0AD | 1157 | 0 | 83 | FEMALE | unknow | Stage I | T1c | M0 | N0 |
| TCGA-A8-A07F | 577 | 0 | 65 | FEMALE | unknow | Stage IIB | T2 | M0 | N1a |
| TCGA-AO-A12E | 2142 | 0 | 51 | FEMALE | unknow | Stage IIB | T3 | M0 | N0 (i+) |
| TCGA-A2-A1G0 | 616 | 0 | 49 | FEMALE | unknow | Stage IIB | T2 | M0 | N1a |
| TCGA-LD-A9QF | 323 | 0 | 73 | FEMALE | unknow | Stage IA | T1c | M0 | N0 |
| TCGA-AC-A6IX | 373 | 0 | 49 | FEMALE | unknow | Stage IIIC | T2 | MX | N3a |
| TCGA-AO-A12A | 3112 | 0 | 47 | FEMALE | unknow | Stage IIA | T2 | M0 | N0 (i+) |
| TCGA-C8-A131 | 411 | 0 | 82 | FEMALE | unknow | Stage IIIA | T2 | M0 | N2 |
| TCGA-AR-A0U3 | 4080 | 0 | 59 | FEMALE | unknow | Stage IIB | T2 | M0 | N1 |
| TCGA-D8-A27H | 397 | 0 | 72 | FEMALE | unknow | Stage IIA | T2 | M0 | N0 |
| TCGA-A8-A079 | 274 | 0 | 69 | FEMALE | unknow | Stage IIIB | T4b | M0 | N3 |
| TCGA-A8-A07U | 760 | 0 | 66 | FEMALE | unknow | Stage IIIA | T2 | M0 | N2a |
| TCGA-C8-A12O | 385 | 0 | 50 | FEMALE | unknow | Stage IIA | T2 | M0 | N0 (i-) |
| TCGA-B6-A402 | 2281 | 0 | 47 | FEMALE | unknow | Stage I | T1c | M0 | N0 (i-) |
| TCGA-E9-A22E | 1269 | 0 | 56 | FEMALE | unknow | Stage IIIA | T2 | M0 | N2 |
| TCGA-A8-A07P | 334 | 0 | 68 | FEMALE | unknow | Stage IIB | T2 | M0 | N1 |
| TCGA-E2-A14Y | 2109 | 0 | 35 | FEMALE | unknow | Stage IIA | T2 | M0 | N0 |
| TCGA-BH-A18P | 921 | 1 | 60 | FEMALE | unknow | Stage I | T1 | M0 | N0 |
| TCGA-E9-A1R0 | 860 | 0 | 58 | FEMALE | unknow | Stage IIA | T1c | M0 | N1 |
| TCGA-A8-A07B | 1308 | 0 | 69 | FEMALE | unknow | Stage IIA | T2 | M0 | N0 |
| TCGA-AN-A0FY | 10 | 0 | 55 | FEMALE | unknow | Stage IA | T1c | M0 | N0 |
| TCGA-E2-A153 | 707 | 0 | 51 | FEMALE | unknow | Stage IIB | T2 | M0 | N1a |
| TCGA-B6-A0IN | 2573 | 1 | 45 | FEMALE | unknow | Stage X | T1c | MX | NX |
| TCGA-A8-A09X | 426 | 1 | 62 | FEMALE | unknow | Stage IIIC | T2 | M0 | N3a |
| TCGA-D8-A1JJ | 611 | 0 | 54 | FEMALE | unknow | Stage IIA | T2 | M0 | N0 |
| TCGA-A2-A0CY | 1673 | 0 | 63 | FEMALE | unknow | Stage IIB | T2 | M0 | N1 |
| TCGA-A2-A0T6 | 575 | 0 | 50 | FEMALE | unknow | Stage IIB | T3 | M0 | N0 (i-) |
| TCGA-D8-A1XD | 522 | 0 | 36 | FEMALE | unknow | Stage IIIA | T1c | M0 | N2a |
| TCGA-AN-A046 | 10 | 0 | 68 | FEMALE | unknow | Stage IIA | T2 | M0 | N0 |
| TCGA-E2-A15H | 393 | 0 | 38 | FEMALE | unknow | Stage IIA | T1c | M0 | N1mi |
| TCGA-A8-A08I | 365 | 0 | 53 | FEMALE | unknow | Stage IIA | T2 | M0 | N0 |
| TCGA-D8-A1XU | 395 | 0 | 56 | FEMALE | unknow | Stage IA | T1c | M0 | N0 |
| TCGA-BH-A1FB | 3669 | 1 | 60 | FEMALE | unknow | Stage IIB | T2 | M0 | N1b |
| TCGA-GM-A2DO | 2596 | 0 | 54 | FEMALE | unknow | Stage I | T1c | M0 | N0 (i-) |
| TCGA-A2-A0EX | 752 | 0 | 46 | FEMALE | unknow | Stage IIB | T3 | M0 | N0 (i+) |
| TCGA-A8-A09K | 912 | 0 | 68 | FEMALE | unknow | Stage IIA | T1c | M0 | N1 |
| TCGA-OL-A66O | 528 | 0 | 39 | FEMALE | unknow | Stage IIB | T2 | MX | N1a |
| TCGA-A7-A13H | 899 | 0 | 61 | FEMALE | unknow | Stage IIB | T2 | MX | N1a |
| TCGA-AQ-A54N | 78 | 0 | 51 | FEMALE | unknow | Stage IIA | T2 | M0 | N0 |
| TCGA-E9-A22A | 1189 | 0 | 74 | FEMALE | unknow | Stage IIA | T2 | M0 | N0 |
| TCGA-BH-A0H7 | 702 | 0 | 65 | FEMALE | unknow | Stage IIIA | T1c | M0 | N2a |
| TCGA-AR-A254 | 2605 | 0 | 50 | FEMALE | unknow | Stage IIIA | T2 | M0 | N2 |
| TCGA-E9-A1RC | 1224 | 0 | 56 | FEMALE | unknow | Stage IIIC | T4b | M0 | N3 |
| TCGA-AR-A250 | 2707 | 0 | 58 | FEMALE | unknow | Stage IIA | T2 | M0 | N0 |
| TCGA-AC-A6NO | 51 | 0 | 43 | FEMALE | unknow | Stage IIB | T2 | MX | N1a |
| TCGA-AR-A1AN | 2920 | 0 | 46 | FEMALE | unknow | Stage IIA | T2 | M0 | N0 |
| TCGA-E2-A14X | 972 | 0 | 55 | FEMALE | unknow | Stage IIIA | T2 | M0 | N2 |
| TCGA-AC-A5XU | 455 | 0 | 74 | FEMALE | unknow | Stage IIB | T3 | MX | N0 (i-) |
| TCGA-E9-A1N3 | 1059 | 0 | 70 | FEMALE | unknow | Stage IIIA | T1c | M0 | N2 |
| TCGA-BH-A0W5 | 1288 | 0 | 77 | FEMALE | unknow | Stage IIA | T1c | M0 | N1a |
| TCGA-B6-A3ZX | 1152 | 1 | 50 | FEMALE | unknow | Stage IV | T3 | M1 | N1 |
| TCGA-AC-A2FM | 792 | 1 | 87 | FEMALE | unknow | Stage IIB | T2 | M0 | N1mi |
| TCGA-D8-A1J8 | 431 | 0 | 77 | FEMALE | unknow | Stage IIB | T2 | M0 | N1a |
| TCGA-E2-A1BC | 501 | 0 | 63 | FEMALE | unknow | Stage IA | T1c | M0 | N0 |
| TCGA-D8-A1JF | 366 | 0 | 79 | FEMALE | unknow | Stage IIIA | T1c | M0 | N2a |
| TCGA-OL-A66I | 714 | 0 | 36 | FEMALE | unknow | Stage IIA | T1c | MX | N1mi |
| TCGA-AC-A6IW | 413 | 0 | 73 | FEMALE | unknow | Stage IIA | T2 | MX | N0 (i-) |
| TCGA-BH-A0AW | 622 | 0 | 56 | FEMALE | unknow | Stage IIA | T1c | M0 | N1a |
| TCGA-BH-A0B8 | 1569 | 0 | 64 | FEMALE | unknow | Stage I | T1b | M0 | N0 (i-) |
| TCGA-A2-A3XU | 912 | 1 | 35 | FEMALE | unknow | Stage IIB | T2 | M0 | N1mi |
| TCGA-B6-A408 | 2072 | 0 | 55 | FEMALE | unknow | Stage IIIC | T2 | M0 | N3a |
| TCGA-E2-A1B6 | 867 | 0 | 44 | FEMALE | unknow | Stage IIA | T2 | M0 | N0 |
| TCGA-C8-A12W | 385 | 0 | 49 | FEMALE | unknow | Stage IIIB | T4 | M0 | N1 |
| TCGA-A8-A095 | 1277 | 0 | 45 | FEMALE | unknow | Stage I | T1c | M0 | N0 |
| TCGA-BH-A1FE | 2273 | 1 | 31 | FEMALE | unknow | Stage IIB | T2 | M0 | N1 |
| TCGA-A2-A04Y | 1099 | 0 | 53 | FEMALE | unknow | Stage IIB | T2 | M0 | N1mi |
| TCGA-A8-A07E | 608 | 0 | 81 | FEMALE | unknow | Stage X | T4b | MX | N3a |
| TCGA-A2-A0T5 | 531 | 0 | 39 | FEMALE | unknow | Stage IIA | T2 | M0 | N0 (i-) |
| TCGA-LL-A6FR | 489 | 0 | 50 | FEMALE | unknow | Stage IIA | T2 | MX | N0 (i-) |
| TCGA-AO-A03U | 1793 | 1 | 31 | FEMALE | unknow | Stage I | T1c | M0 | N0 (i-) |
| TCGA-AR-A24O | 3607 | 0 | 43 | FEMALE | unknow | Stage IIIA | T3 | M0 | N2 |
| TCGA-A2-A1FX | 1847 | 0 | 61 | FEMALE | unknow | Stage IIIA | T3 | M0 | N2a |
| TCGA-C8-A1HM | 375 | 0 | 74 | FEMALE | unknow | Stage IIA | T2 | M0 | N0 |
| TCGA-A2-A0CU | 158 | 1 | 73 | FEMALE | unknow | Stage IIA | T2 | M0 | N0 (i+) |
| TCGA-BH-A0HX | 829 | 0 | 54 | FEMALE | unknow | Stage IIB | T2 | M0 | N1a |
| TCGA-A2-A0ER | 2263 | 0 | 63 | FEMALE | unknow | Stage IB | T1c | M0 | N1mi |
| TCGA-AR-A1AU | 2868 | 0 | 39 | FEMALE | unknow | Stage IIIA | T2 | M0 | N2 |
| TCGA-C8-A12K | 0 | 0 | 80 | FEMALE | unknow | Stage IIB | T2 | M0 | N1 |
| TCGA-AC-A2FE | 2636 | 1 | 62 | FEMALE | unknow | Stage IIIC | T3 | MX | N3a |
| TCGA-A2-A0YM | 965 | 0 | 67 | FEMALE | unknow | Stage IIA | T2 | M0 | N0 (i-) |
| TCGA-D8-A1XA | 839 | 0 | 64 | FEMALE | unknow | Stage IA | T1c | M0 | N0 |
| TCGA-AN-A03X | 10 | 0 | 74 | FEMALE | unknow | Stage IIA | T2 | M0 | N0 |
| TCGA-BH-A42V | 635 | 0 | 41 | FEMALE | unknow | Stage IB | T1c | M0 | N1mi |
| TCGA-AR-A2LL | 2012 | 0 | 70 | FEMALE | unknow | Stage IIB | T3 | M0 | N0 |
| TCGA-A2-A4RY | 648 | 0 | 46 | FEMALE | unknow | Stage IIIC | T3 | M0 | N3a |
| TCGA-A8-A09T | 579 | 0 | 68 | FEMALE | unknow | unknow | T1c | MX | N0 |
| TCGA-AC-A3HN | 496 | 0 | 87 | FEMALE | unknow | Stage IIB | T2 | MX | N1a |
| TCGA-EW-A6SD | 1010 | 0 | 32 | FEMALE | unknow | Stage IIB | T2 | M0 | N1a |
| TCGA-A8-A093 | 546 | 0 | 61 | FEMALE | unknow | Stage IIA | T2 | M0 | N0 |
| TCGA-B6-A0IH | 2965 | 1 | 81 | FEMALE | unknow | Stage IIIA | T3 | M0 | N1b |
| TCGA-A2-A0CL | 3015 | 0 | 37 | FEMALE | unknow | Stage IIIA | T3 | M0 | N2a |
| TCGA-AO-A129 | 3286 | 0 | 29 | FEMALE | unknow | Stage IIB | T2 | M0 | N1a |
| TCGA-D8-A1Y0 | 472 | 0 | 65 | FEMALE | unknow | Stage IIIA | T1c | MX | N2a |
| TCGA-AR-A1AX | 2629 | 0 | 64 | FEMALE | unknow | Stage I | T1 | M0 | N0 |
| TCGA-E9-A24A | 747 | 0 | 69 | FEMALE | unknow | Stage IIA | T1c | M0 | N1 |
| TCGA-BH-A0HN | 516 | 0 | 67 | FEMALE | unknow | Stage IA | T1c | M0 | N0 (i-) |
| TCGA-D8-A143 | 431 | 0 | 51 | FEMALE | unknow | Stage IIA | T2 | M0 | N0 |
| TCGA-BH-A0DQ | 98 | 0 | 42 | FEMALE | unknow | Stage IIB | T2 | M0 | N1 |
| TCGA-A8-A09C | 31 | 0 | 69 | FEMALE | unknow | Stage X | T2 | MX | N0 |
| TCGA-AN-A049 | 19 | 0 | 62 | FEMALE | unknow | Stage IIA | T2 | M0 | N0 |
| TCGA-E9-A226 | 1048 | 1 | 45 | FEMALE | unknow | Stage IIIA | T2 | M0 | N2 |
| TCGA-AR-A2LH | 616 | 1 | 55 | FEMALE | unknow | Stage III | T3 | M0 | N3 |
| TCGA-AR-A24Q | 3172 | 0 | 49 | FEMALE | unknow | Stage IIB | T3 | M0 | N0 |
| TCGA-E2-A15R | 1732 | 0 | 64 | FEMALE | unknow | Stage IIA | T1c | M0 | N1a |
| TCGA-E2-A15C | 694 | 0 | 61 | FEMALE | unknow | Stage I | T1c | M0 | N0 |
| TCGA-PE-A5DC | 1430 | 1 | 72 | FEMALE | unknow | Stage IIIA | T2 | M0 | N2a |
| TCGA-B6-A0I8 | 749 | 1 | 46 | FEMALE | unknow | Stage X | T1 | M0 | NX |
| TCGA-BH-A0BD | 554 | 0 | 47 | FEMALE | unknow | Stage IIA | T1c | M0 | N1a |
| TCGA-A8-A09V | 457 | 0 | 51 | FEMALE | unknow | Stage IIA | T2 | M0 | N0 |
| TCGA-A8-A08Z | 1217 | 0 | 76 | FEMALE | unknow | unknow | T4b | M0 | N3a |
| TCGA-A2-A0YL | 1474 | 0 | 48 | FEMALE | unknow | Stage IIIA | T3 | M0 | N2a |
| TCGA-A2-A0D1 | 1051 | 0 | 76 | FEMALE | unknow | Stage IIA | T2 | M0 | N0 (i-) |
| TCGA-B6-A0I2 | 4361 | 0 | 45 | FEMALE | unknow | Stage IA | T1c | M0 | N0 (i-) |
| TCGA-A2-A0SU | 1662 | 0 | 66 | FEMALE | unknow | Stage IIA | T2 | M0 | N0 (i+) |
| TCGA-A8-A06P | 396 | 0 | 63 | FEMALE | unknow | Stage IIIA | T1c | M0 | N2a |
| TCGA-AO-A0JB | 1542 | 0 | 50 | FEMALE | unknow | Stage IIIA | T3 | M0 | N1a |
| TCGA-B6-A2IU | 5176 | 0 | 62 | FEMALE | unknow | Stage IIA | T2 | M0 | N0 |
| TCGA-C8-A273 | 513 | 0 | 29 | FEMALE | unknow | Stage IIB | T2 | M0 | N1 |
| TCGA-XX-A89A | 488 | 0 | 68 | FEMALE | unknow | Stage IIB | T3 | MX | N0 |
| TCGA-A8-A09I | 1371 | 0 | 84 | FEMALE | unknow | Stage IIA | T2 | M0 | N0 |
| TCGA-C8-A27A | 747 | 0 | 48 | FEMALE | unknow | Stage IIB | T2 | M0 | N1 |
| TCGA-AO-A12C | 2372 | 0 | 42 | FEMALE | unknow | Stage IIB | T2 | M0 | N1a |
| TCGA-A8-A08B | 1156 | 0 | 52 | FEMALE | unknow | Stage IIA | T2 | M0 | N0 |
| TCGA-C8-A1HO | 375 | 0 | 34 | FEMALE | unknow | Stage IIIC | T3 | M0 | N3 |
| TCGA-C8-A1HE | 375 | 0 | 59 | FEMALE | unknow | Stage IIA | T2 | M0 | N0 |
| TCGA-BH-A6R8 | 293 | 0 | 46 | FEMALE | unknow | Stage IIA | T2 | M0 | N0 |
| TCGA-EW-A2FW | 672 | 0 | 52 | FEMALE | unknow | Stage IIA | T2 | MX | N0 (i-) |
| TCGA-E2-A10F | 878 | 0 | 47 | FEMALE | unknow | Stage IIA | T2 | M0 | N0 (i-) |
| TCGA-GM-A3XN | 2019 | 0 | 44 | FEMALE | unknow | Stage IIIA | T1c | M0 | N2a |
| TCGA-C8-A12T | 0 | 0 | 43 | FEMALE | unknow | Stage IIA | T2 | M0 | N0 |
| TCGA-OL-A5D7 | 1780 | 0 | 70 | FEMALE | unknow | Stage IIA | T2 | MX | N0 |
| TCGA-AQ-A0Y5 | 172 | 1 | 70 | FEMALE | unknow | Stage IIIA | T2 | MX | N2a |
| TCGA-A2-A1FZ | 683 | 0 | 63 | FEMALE | unknow | Stage IIA | T2 | M0 | N0 (i-) |
| TCGA-3C-AALJ | 1474 | 0 | 62 | FEMALE | unknow | Stage IIB | T2 | M0 | N1a |
| TCGA-V7-A7HQ | 2033 | 0 | 75 | FEMALE | unknow | Stage IIIA | T1c | M0 | N2a |
| TCGA-AR-A255 | 2161 | 0 | 62 | FEMALE | unknow | Stage I | T1 | M0 | N0 |
| TCGA-A7-A6VW | 285 | 0 | 48 | FEMALE | unknow | Stage IIA | T2 | M0 | N0 |
| TCGA-BH-A0W4 | 759 | 0 | 46 | FEMALE | unknow | Stage IIA | T2 | M0 | N0 (i-) |
| TCGA-GM-A2DF | 2155 | 0 | 53 | FEMALE | unknow | Stage IIA | T1c | M0 | N1a |
| TCGA-E2-A1LI | 3121 | 0 | 57 | FEMALE | unknow | Stage IIB | T2 | M0 | N1 |
| TCGA-A2-A0ET | 1066 | 0 | 58 | FEMALE | unknow | Stage IIIA | T2 | M0 | N2a |
| TCGA-B6-A0RE | 7777 | 0 | 61 | FEMALE | unknow | Stage X | TX | M0 | N0 (i-) |
| TCGA-A8-A06Q | 31 | 0 | 63 | FEMALE | unknow | Stage IIIA | T3 | M0 | N2 |
| TCGA-LL-A441 | 996 | 0 | 62 | FEMALE | unknow | Stage IA | T1c | MX | N0 |
| TCGA-BH-A0DT | 2403 | 0 | 41 | FEMALE | unknow | Stage IIA | T1c | M0 | N1a |
| TCGA-C8-A274 | 508 | 0 | 63 | FEMALE | unknow | Stage IIA | T2 | M0 | N0 |
| TCGA-LL-A442 | 889 | 0 | 56 | FEMALE | unknow | Stage IIA | T2 | MX | N0 (i-) |
| TCGA-E2-A573 | 1062 | 0 | 48 | FEMALE | unknow | Stage IA | T1c | M0 | N0 |
| TCGA-C8-A26Y | 0 | 0 | 90 | FEMALE | unknow | Stage IIA | T2 | M0 | N0 |
| TCGA-GM-A2D9 | 1812 | 1 | 69 | FEMALE | unknow | Stage I | T1c | M0 | N0 (i-) |
| TCGA-A2-A0EO | 2442 | 0 | 54 | FEMALE | unknow | Stage IA | T1 | M0 | N0 (i-) |
| TCGA-B6-A0WS | 2965 | 1 | 58 | FEMALE | unknow | Stage X | T1c | MX | NX |
| TCGA-BH-A0HP | 414 | 0 | 65 | FEMALE | unknow | Stage IIIA | T3 | M0 | N2a |
| TCGA-D8-A1JU | 447 | 0 | 51 | FEMALE | unknow | Stage IA | T1c | M0 | N0 |
| TCGA-A2-A1G4 | 595 | 0 | 71 | FEMALE | unknow | Stage IIIA | T3 | M0 | N1a |
| TCGA-A8-A094 | 0 | 0 | 75 | FEMALE | unknow | Stage IIA | T2 | M0 | N0 |
| TCGA-BH-A0DV | 2064 | 0 | 54 | FEMALE | unknow | Stage IIIA | T2 | M0 | N2a |
| TCGA-A2-A0EY | 1925 | 0 | 62 | FEMALE | unknow | Stage IIB | T2 | M0 | N1a |
| TCGA-AR-A24N | 3035 | 0 | 54 | FEMALE | unknow | Stage I | T1 | M0 | N0 |
| TCGA-AR-A1AS | 1150 | 0 | 54 | FEMALE | unknow | Stage IIB | T2 | M0 | N1 |
| TCGA-BH-A0HI | 620 | 0 | 78 | FEMALE | unknow | Stage IA | T1c | M0 | N0 (i-) |
| TCGA-A7-A13E | 614 | 1 | 62 | FEMALE | unknow | Stage IIB | T2 | MX | N1 |
| TCGA-AN-A0XL | 163 | 0 | 61 | FEMALE | unknow | Stage IIA | T2 | M0 | N0 |
| TCGA-A8-A084 | 458 | 0 | 81 | FEMALE | unknow | Stage IIB | T2 | M0 | N1a |
| TCGA-AN-A0G0 | 16 | 0 | 56 | FEMALE | unknow | Stage IIA | T2 | M0 | N0 |
| TCGA-D8-A13Y | 1728 | 0 | 52 | FEMALE | unknow | Stage IA | T1c | M0 | N0 |
| TCGA-AN-A0XR | 10 | 0 | 55 | FEMALE | unknow | Stage IIIA | T2 | M0 | N2 |
| TCGA-D8-A1JI | 577 | 0 | 54 | FEMALE | unknow | Stage IIA | T1c | MX | N1a |
| TCGA-E9-A227 | 975 | 0 | 42 | FEMALE | unknow | Stage IIB | T2 | M0 | N1 |
| TCGA-E2-A574 | 1179 | 0 | 44 | FEMALE | unknow | Stage IIA | T2 | M0 | N0 |
| TCGA-A8-A086 | 396 | 0 | 59 | FEMALE | unknow | Stage IIA | T1c | M0 | N1a |
| TCGA-A2-A0ES | 2190 | 0 | 52 | FEMALE | unknow | Stage IIA | T2 | M0 | N0 (i-) |
| TCGA-A7-A0CE | 1074 | 0 | 57 | FEMALE | unknow | Stage IIA | T2 | M0 | N0 (i-) |
| TCGA-BH-A0DH | 1156 | 0 | 63 | FEMALE | unknow | Stage IIB | T2 | M0 | N1a |
| TCGA-A2-A0T4 | 624 | 0 | 62 | FEMALE | unknow | Stage IIA | T2 | M0 | N0 (i-) |
| TCGA-B6-A401 | 2596 | 0 | 47 | FEMALE | unknow | Stage IIA | T1c | M0 | N1a |
| TCGA-AR-A2LM | 1935 | 0 | 49 | FEMALE | unknow | Stage II | T1 | M0 | N1 |
| TCGA-GM-A2DB | 2406 | 0 | 62 | FEMALE | unknow | Stage IIA | T2 | M0 | N0 (i-) |
| TCGA-A2-A0ST | 3017 | 0 | 62 | FEMALE | unknow | Stage IIA | T1c | M0 | N1a |
| TCGA-B6-A0WT | 5739 | 0 | 61 | FEMALE | unknow | Stage IIB | T2 | M0 | N1 |
| TCGA-E2-A1LG | 1523 | 0 | 50 | FEMALE | unknow | Stage IIA | T2 | M0 | N0 |
| TCGA-E9-A6HE | 847 | 0 | 45 | FEMALE | unknow | Stage IIIC | T3 | MX | N3 |
| TCGA-GM-A3XG | 1330 | 0 | 46 | FEMALE | unknow | Stage IIIA | T3 | M0 | N1a |
| TCGA-JL-A3YW | 360 | 0 | 49 | FEMALE | unknow | Stage IIB | T2 | M0 | N1 |
| TCGA-D8-A1JN | 620 | 0 | 80 | FEMALE | unknow | Stage IIIC | T3 | MX | N3a |
| TCGA-D8-A1Y2 | 433 | 0 | 71 | FEMALE | unknow | Stage IIA | T2 | MX | N0 |
| TCGA-D8-A1XM | 538 | 0 | 57 | FEMALE | unknow | Stage IA | T1c | M0 | N0 |
| TCGA-BH-A0BW | 2371 | 0 | 71 | FEMALE | unknow | Stage I | T1c | M0 | N0 |
| TCGA-D8-A27V | 381 | 0 | 62 | FEMALE | unknow | Stage IIA | T2 | M0 | N0 |
| TCGA-A2-A4S2 | 643 | 0 | 62 | FEMALE | unknow | Stage IIIA | T3 | M0 | N2 |
| TCGA-EW-A424 | 715 | 0 | 51 | FEMALE | unknow | Stage IIIA | T3 | MX | N1 |
| TCGA-A1-A0SG | 434 | 0 | 61 | FEMALE | unknow | Stage IIB | T2 | M0 | N1a |
| TCGA-LL-A8F5 | 596 | 0 | 61 | FEMALE | unknow | Stage IIA | T2 | MX | N0 (i-) |
| TCGA-E9-A1RD | 34 | 0 | 67 | FEMALE | unknow | Stage IIA | T2 | M0 | N0 |
| TCGA-OL-A6VO | 858 | 0 | 43 | FEMALE | unknow | Stage IA | T1c | MX | N0 |
| TCGA-B6-A0I5 | 8556 | 0 | 49 | FEMALE | unknow | Stage IIB | T2 | M0 | N1b |
| TCGA-BH-A1FD | 1009 | 1 | 68 | FEMALE | unknow | Stage I | T1c | M0 | N0 |
| TCGA-D8-A1XR | 482 | 0 | 56 | FEMALE | unknow | Stage IIB | T2 | M0 | N1a |
| TCGA-BH-A0B9 | 1572 | 0 | 44 | FEMALE | unknow | Stage IA | T1c | M0 | N0 (i-) |
| TCGA-BH-A0AV | 1820 | 0 | 52 | FEMALE | unknow | Stage I | T1c | M0 | N0 |
| TCGA-E2-A3DX | 1325 | 0 | 43 | FEMALE | unknow | Stage IIIC | T2 | M0 | N3 |
| TCGA-BH-A0HQ | 1121 | 0 | 56 | FEMALE | unknow | Stage IIA | T2 | M0 | N0 (i+) |
| TCGA-A7-A4SC | 446 | 0 | 62 | FEMALE | unknow | Stage IIB | T3 | MX | N0 |
| TCGA-C8-A137 | 379 | 0 | 34 | FEMALE | unknow | Stage IIB | T2 | M0 | N1 |
| TCGA-S3-AA17 | 424 | 0 | 64 | FEMALE | unknow | Stage IIB | T2 | cM0 (i+) | N1mi |
| TCGA-AC-A3W7 | 471 | 0 | 66 | FEMALE | unknow | Stage IIB | T2 | MX | N1a |
| TCGA-AN-A0FK | 213 | 0 | 88 | FEMALE | unknow | Stage IIIB | T4 | M0 | N0 |
| TCGA-A2-A0YD | 769 | 0 | 63 | FEMALE | unknow | Stage IIB | T3 | M0 | N0 (i+) |
| TCGA-D8-A1JH | 426 | 0 | 56 | FEMALE | unknow | Stage IA | T1c | M0 | N0 |
| TCGA-BH-A0H0 | 461 | 0 | 69 | FEMALE | unknow | Stage IA | T1c | M0 | N0 (i-) |
| TCGA-AN-A0XW | 170 | 0 | 36 | FEMALE | unknow | Stage IIIA | T2 | M0 | N2 |
| TCGA-E9-A1NC | 1203 | 0 | 61 | FEMALE | unknow | Stage IIB | T2 | M0 | N1 |
| TCGA-B6-A1KI | 2236 | 0 | 63 | FEMALE | unknow | Stage I | T1c | M0 | N0 |
| TCGA-E2-A14U | 1318 | 0 | 74 | FEMALE | unknow | Stage I | T1c | M0 | N0 |
| TCGA-OL-A5DA | 1783 | 0 | 61 | FEMALE | unknow | Stage IIA | T2 | MX | N0 |
| TCGA-A8-A07C | 1034 | 0 | 57 | FEMALE | unknow | Stage IIA | T2 | M0 | N0 |
| TCGA-EW-A1IY | 258 | 0 | 38 | FEMALE | unknow | Stage I | T1c | MX | N0 |
| TCGA-AN-A0AL | 227 | 0 | 41 | FEMALE | unknow | Stage IIIB | T4 | M0 | N0 |
| TCGA-A2-A0CM | 754 | 1 | 40 | FEMALE | unknow | Stage IIA | T2 | M0 | N0 (i-) |
| TCGA-AR-A24H | 4894 | 0 | 65 | FEMALE | unknow | Stage IIA | T2 | M0 | N0 |
| TCGA-LL-A7SZ | 594 | 0 | 49 | FEMALE | unknow | Stage IIB | T2 | MX | N1a |
| TCGA-D8-A1JE | 575 | 0 | 62 | FEMALE | unknow | Stage IIA | T1c | M0 | N1a |
| TCGA-E9-A1N5 | 1120 | 0 | 45 | FEMALE | unknow | Stage IIB | T2 | M0 | N1 |
| TCGA-BH-A0BO | 2197 | 0 | 54 | FEMALE | unknow | Stage I | T1b | M0 | N0 |
| TCGA-A8-A08G | 607 | 0 | 41 | FEMALE | unknow | Stage IIA | T2 | M0 | N0 |
| TCGA-AR-A1AR | 524 | 1 | 50 | FEMALE | unknow | Stage IIIA | T1 | M0 | N2 |
| TCGA-A2-A0YT | 723 | 1 | 56 | FEMALE | unknow | Stage IIIB | T4b | M0 | N2a |
| TCGA-AN-A0XO | 375 | 0 | 59 | FEMALE | unknow | Stage IIIA | T2 | M0 | N2 |
| TCGA-A2-A0CZ | 1616 | 0 | 46 | FEMALE | unknow | Stage IIA | T2 | M0 | N0 (i-) |
| TCGA-E9-A1NE | 1088 | 0 | 28 | FEMALE | unknow | Stage IIB | T2 | M0 | N1 |
| TCGA-E9-A1R2 | 1063 | 0 | 51 | FEMALE | unknow | Stage IIIC | T2 | MX | N3 |
| TCGA-B6-A0RQ | 4267 | 1 | 68 | FEMALE | unknow | Stage IIB | T3 | M0 | N0 (i-) |
| TCGA-AC-A23C | 585 | 0 | 62 | FEMALE | unknow | Stage IIB | T2 | MX | N1 |
| TCGA-BH-A0BL | 2278 | 0 | 35 | FEMALE | unknow | Stage I | T1c | M0 | N0 |
| TCGA-A8-A076 | 1642 | 0 | 66 | FEMALE | unknow | Stage IIA | T2 | M0 | N0 |
| TCGA-A1-A0SN | 1196 | 0 | 50 | FEMALE | unknow | Stage IIA | T1c | MX | N1 |
| TCGA-A8-A08O | 943 | 0 | 45 | FEMALE | unknow | Stage IV | T2 | M1 | N3a |
| TCGA-AC-A8OQ | 34 | 0 | 72 | FEMALE | unknow | Stage IIB | T2 | MX | N1a |
| TCGA-E9-A22D | 1248 | 0 | 38 | FEMALE | unknow | Stage IIA | T2 | M0 | N0 |
| TCGA-AR-A24L | 2866 | 1 | 26 | FEMALE | unknow | Stage IIB | T2 | M0 | N1 |
| TCGA-E2-A1LK | 266 | 1 | 84 | FEMALE | unknow | Stage IIIC | T4b | M0 | N3a |
