# Supplementary material for: Identification and validation of DNA methylation-driven gene OSR1 as a novel tumor suppressor for the diagnosis and prognosis of breast cancer
Source: Front Genet. 2025 Jul 7;16:1583620. doi: 10.3389/fgene.2025.1583620 (PMC12277919; doi:10.3389/fgene.2025.1583620)
Supplement: Supplementary file 2 [file Table2.docx]

TableS2

| gene_name | log2FC | padj |
| --- | --- | --- |
| ECRG4 | 3.659736749 | 2.0053E-229 |
| CMTM5 | 3.845365771 | 6.6834E-159 |
| PDE1C | 2.143461669 | 1.8614E-154 |
| IL17B | 2.83457739 | 2.8115E-153 |
| LEFTY2 | 3.383033142 | 7.15E-149 |
| ADGRD2 | 4.025433884 | 6.671E-139 |
| TAFA3 | 3.029803651 | 6.5485E-133 |
| LOXL4 | 2.310267705 | 7.0795E-128 |
| NCAN | 4.554355284 | 1.9983E-125 |
| SOX8 | 3.212733199 | 1.1132E-124 |
| NGFR | 2.091827767 | 9.2366E-124 |
| WIF1 | 4.320309805 | 9.5267E-123 |
| PTX3 | 2.90400072 | 1.8247E-120 |
| SYNM | 2.12680241 | 2.7659E-114 |
| EDN3 | 3.733556685 | 9.5895E-114 |
| CAPN6 | 3.242835436 | 1.3365E-113 |
| CLDN11 | 1.759598641 | 1.5738E-113 |
| LINC02487 | 3.521629242 | 2.0245E-112 |
| ATOH8 | 1.800708745 | 1.0137E-109 |
| SMYD1 | 3.370460872 | 1.1774E-109 |
| LINC02613 | 2.455689383 | 1.2171E-109 |
| CAVIN2 | 1.853955236 | 2.9451E-108 |
| SLC7A3 | 2.902140672 | 8.7024E-105 |
| SNORC | 2.28704076 | 7.4316E-104 |
| TNXB | 1.810096287 | 1.4168E-103 |
| ACTG2 | 2.672119022 | 2.7379E-103 |
| COL17A1 | 2.476709538 | 2.9861E-103 |
| BOC | 1.532516144 | 6.2571E-103 |
| NPY2R | 4.721893526 | 6.6049E-101 |
| KCNMB1 | 1.580421024 | 1.32871E-100 |
| CLDN19 | 2.822262478 | 3.86647E-99 |
| TCEAL5 | 2.457895628 | 1.03616E-98 |
| IRX1 | 2.515730482 | 3.78503E-98 |
| TSLP | 2.020600343 | 7.54931E-98 |
| ADAMTS9-AS1 | 1.958076261 | 1.03521E-97 |
| CHGA | -4.428405609 | 2.13839E-97 |
| RELN | 2.224440427 | 1.61049E-96 |
| CPLX2 | -5.363285063 | 4.42061E-96 |
| COL9A1 | 3.543831178 | 8.22196E-96 |
| PAK5 | 2.585475324 | 4.3232E-93 |
| SCRG1 | 2.959327052 | 6.43685E-92 |
| AC015909.3 | 3.44762062 | 3.91054E-91 |
| RHEX | 2.55390367 | 1.35573E-90 |
| GDF10 | 2.046742509 | 4.22876E-90 |
| CA6 | 4.144915794 | 4.6807E-90 |
| MPZ | 1.921930784 | 7.77432E-90 |
| VEGFD | 2.089525225 | 8.3071E-90 |
| C6orf15 | 3.278617871 | 1.10956E-89 |
| SPRY2 | 1.182850202 | 1.45069E-89 |
| PAMR1 | 1.445423582 | 3.51946E-89 |
| AC099541.2 | 2.373945632 | 7.60899E-89 |
| LINC00092 | 2.474630494 | 7.85001E-89 |
| OVCH2 | 2.494513939 | 1.07302E-88 |
| ABCA10 | 1.686729555 | 6.19314E-88 |
| AQP1 | 1.163585642 | 6.66191E-87 |
| AC104407.1 | 4.712653252 | 1.75408E-86 |
| SPAAR | 1.773213085 | 1.88727E-86 |
| MYH11 | 1.76395932 | 1.28028E-85 |
| PTCH2 | 1.219286066 | 1.77491E-85 |
| ADAM33 | 1.668110067 | 2.14008E-85 |
| ADAMTS9-AS2 | 1.709864691 | 4.0527E-85 |
| KLHL29 | 1.338518913 | 6.08156E-85 |
| AL031668.2 | 2.527324289 | 9.46943E-85 |
| ETV3L | 3.067078953 | 1.40981E-84 |
| ELN | 1.762140188 | 1.45518E-84 |
| HTR2A | 1.978231172 | 1.76519E-84 |
| ALX4 | 2.220725478 | 2.28371E-83 |
| PROS1 | 1.077048594 | 3.36808E-83 |
| PHF24 | 2.252244371 | 7.19057E-83 |
| COL11A2 | 2.187523653 | 4.42852E-82 |
| MIR145 | 2.094272986 | 2.87932E-81 |
| CCL14 | 2.126743628 | 1.05429E-80 |
| MEOX1 | 1.814906068 | 1.71894E-80 |
| C2orf88 | 1.616542343 | 2.43979E-80 |
| ITGA7 | 1.329739186 | 6.08286E-80 |
| ABCA8 | 1.847169834 | 7.07168E-80 |
| PCSK1 | -3.014928853 | 1.39907E-79 |
| TMEM100 | 1.904919663 | 2.16094E-79 |
| LINC01152 | 2.631229022 | 3.71504E-79 |
| PTN | 1.962850621 | 2.82253E-78 |
| H1-9P | 2.675540365 | 2.83184E-78 |
| GPR17 | 1.813933687 | 2.87563E-78 |
| ITGA9 | 1.078721625 | 2.87563E-78 |
| EMILIN3 | 2.464567888 | 5.07044E-78 |
| LINC02884 | 1.727184094 | 6.04919E-78 |
| CTD-2297D10.2 | 2.540242071 | 6.04919E-78 |
| KIAA1755 | 1.330894194 | 4.4161E-77 |
| AC036108.2 | 2.277406958 | 5.20805E-77 |
| AC008459.1 | 4.18447007 | 9.44396E-77 |
| SLC22A11 | 1.959678994 | 2.44621E-76 |
| B3GALT1 | 1.935197754 | 2.06659E-75 |
| RYR3 | 1.35587606 | 2.2988E-75 |
| AC036108.3 | 1.797985136 | 2.39745E-75 |
| GRIA4 | 2.403725643 | 3.72144E-75 |
| SLC7A14-AS1 | 2.247310436 | 6.79477E-75 |
| AL109809.4 | 1.819576031 | 6.973E-75 |
| PNMA8C | 2.108374613 | 7.49548E-75 |
| MFAP4 | 1.539998548 | 1.32867E-74 |
| PTGFR | 1.869944598 | 1.38853E-74 |
| EDAR | 2.512531942 | 2.61649E-74 |
| RASL12 | 1.078865484 | 1.44067E-73 |
| USP44 | 1.40335243 | 1.86291E-73 |
| ITIH5 | 1.393511213 | 3.22162E-73 |
| MMRN1 | 1.812830792 | 5.15338E-73 |
| STAB2 | 1.614388963 | 5.61459E-73 |
| FREM1 | 1.586363691 | 8.8017E-73 |
| CHGB | -3.825624821 | 1.16316E-72 |
| CADM3-AS1 | 2.057482443 | 1.52424E-72 |
| AC022509.2 | 2.07730438 | 1.69795E-72 |
| AL603840.1 | 2.362395811 | 2.0554E-72 |
| NSG1 | 1.854249349 | 2.45454E-72 |
| CMA1 | 1.993026939 | 2.88895E-72 |
| ACKR1 | 2.041835039 | 5.73695E-72 |
| ITGA10 | 1.363034476 | 8.54399E-72 |
| ADCYAP1R1 | 1.51883478 | 9.56443E-72 |
| SCN5A | 1.558234621 | 1.03021E-71 |
| ANGPTL7 | 2.780237203 | 1.68284E-71 |
| IGF1 | 1.452740511 | 1.68695E-71 |
| MT1M | 1.640418969 | 1.73667E-71 |
| COL14A1 | 1.503318655 | 2.48326E-71 |
| ID4 | 1.75530027 | 2.95999E-71 |
| ALKAL2 | 2.463332146 | 8.65448E-71 |
| TRABD2B | 1.528864801 | 8.68941E-71 |
| BMX | 1.376961194 | 5.64238E-70 |
| FAM107A | 1.82494068 | 8.73332E-70 |
| NRXN2 | 1.271408425 | 3.10654E-69 |
| FAM110D | 1.20521571 | 3.80506E-69 |
| CHRDL1 | 1.899280729 | 1.49045E-68 |
| ROBO3 | 1.140055028 | 2.035E-68 |
| KCNA1 | 1.994409655 | 2.70966E-68 |
| AL583785.1 | 1.875200553 | 3.90302E-68 |
| TEPP | 1.968222549 | 6.32889E-68 |
| SPHKAP | 3.340543385 | 2.06406E-67 |
| AC068473.3 | 1.680355334 | 4.19689E-67 |
| LINC01819 | 2.527149849 | 8.85792E-67 |
| KIT | 1.629094879 | 1.71356E-66 |
| GPIHBP1 | 1.531871842 | 1.72119E-66 |
| RGMA | 1.607998467 | 2.09676E-66 |
| WDR86 | 1.351785178 | 4.69991E-66 |
| S100B | 2.157158275 | 7.72997E-66 |
| GFAP | 1.923395068 | 1.18134E-65 |
| GLI1 | 1.338815658 | 2.05634E-65 |
| DSG1 | 3.015304426 | 2.65854E-65 |
| AVPR2 | 1.451895785 | 3.03188E-65 |
| TACR1 | 1.726058117 | 3.05221E-65 |
| SCN4B | 1.197518622 | 4.88483E-65 |
| MAP1LC3C | 1.548630891 | 5.32617E-65 |
| LINC01140 | 1.171358533 | 5.36867E-65 |
| C1QTNF4 | 1.733111589 | 6.01575E-65 |
| TRPM6 | 1.793987785 | 8.97214E-65 |
| ANKRD53 | 1.025134582 | 1.94063E-64 |
| AC022509.1 | 2.039921417 | 2.12892E-64 |
| CNTNAP3P2 | 1.800139552 | 2.61073E-64 |
| AC008055.2 | 3.277955319 | 4.38765E-64 |
| PPP1R14A | 1.409285146 | 4.86557E-64 |
| CHRM3 | 2.16155531 | 5.15809E-64 |
| AC097713.1 | 3.135142371 | 7.8693E-64 |
| ROPN1B | 2.639304381 | 9.26915E-64 |
| SCARA5 | 2.162602217 | 1.21321E-63 |
| CAPN11 | 1.189970265 | 1.71884E-63 |
| LINC00987 | 1.29460583 | 1.96658E-63 |
| CYTL1 | 1.240574303 | 2.74772E-63 |
| LINC02580 | 1.361104245 | 4.91241E-63 |
| LYVE1 | 1.419493036 | 8.73012E-63 |
| CD300LG | 2.17222737 | 1.31663E-62 |
| AL449403.2 | 3.574149771 | 1.78396E-62 |
| NFE4 | 3.691715773 | 1.88375E-62 |
| ACTA2-AS1 | 1.446575998 | 1.96833E-62 |
| RPL26P30 | 1.692983133 | 5.2367E-62 |
| COL9A3 | 2.17782777 | 6.52291E-62 |
| CHST3 | 1.132235578 | 8.22368E-62 |
| CYP2A6 | -3.257100095 | 8.28376E-62 |
| LRRN4CL | 1.371494649 | 8.86408E-62 |
| CCKBR | 3.57896096 | 9.97468E-62 |
| CARMN | 1.482617224 | 1.17827E-61 |
| AC093496.1 | 3.152405299 | 1.5569E-61 |
| IL34 | 1.551124993 | 2.97366E-61 |
| CADM3 | 1.91355048 | 3.33502E-61 |
| AC243562.2 | 1.674967376 | 3.59688E-61 |
| OCA2 | 2.919485401 | 3.71574E-61 |
| LINC00842 | 2.824444088 | 4.83891E-61 |
| CLDN5 | 1.368187216 | 1.30955E-60 |
| AC005906.2 | 2.304168457 | 5.32463E-60 |
| CCL16 | 1.677359503 | 8.50575E-60 |
| SAMD5 | 1.478504943 | 1.06263E-59 |
| MAML2 | 1.042022476 | 2.47695E-59 |
| NPR1 | 1.019895684 | 2.57705E-59 |
| TSPAN7 | 1.189173159 | 9.03272E-59 |
| MFGE8 | 1.323875844 | 9.34787E-59 |
| AC008514.1 | 2.131496979 | 9.34787E-59 |
| MRGPRX3 | 2.381007242 | 1.02101E-58 |
| CCDC8 | 1.226147403 | 1.13471E-58 |
| SOX17 | 1.016727837 | 1.22466E-58 |
| OR2S1P | 2.191709411 | 1.24699E-58 |
| INMT | 1.294109007 | 2.1372E-58 |
| AP003555.2 | 1.636793968 | 2.31367E-58 |
| LINC02538 | 2.330827129 | 3.42678E-58 |
| RBMS3-AS3 | 1.441948726 | 3.96863E-58 |
| EGR2 | 1.203319144 | 4.74743E-58 |
| IGSF10 | 1.51759167 | 4.8664E-58 |
| HRCT1 | 2.099312633 | 5.11094E-58 |
| PLAC9 | 1.314403463 | 6.32594E-58 |
| PDE9A-AS1 | 1.588873229 | 7.53169E-58 |
| MMP20 | 2.90585301 | 1.19725E-57 |
| ANGPT4 | 1.319709769 | 1.46149E-57 |
| FNDC4 | 1.030208085 | 1.5242E-57 |
| AL450332.1 | 2.139350222 | 1.5309E-57 |
| ITM2A | 1.201475287 | 1.6921E-57 |
| RNF175 | 1.098663259 | 1.74032E-57 |
| CEROX1 | 1.843620782 | 1.74505E-57 |
| CNTNAP3B | 1.606277013 | 1.77659E-57 |
| CTSG | 1.763777767 | 2.08368E-57 |
| FXYD6 | 1.00832966 | 2.60153E-57 |
| POU3F3 | 2.507257702 | 3.15493E-57 |
| CNN1 | 1.341282398 | 3.4273E-57 |
| DEPP1 | 1.066048724 | 3.57851E-57 |
| APCDD1L-DT | 1.916679968 | 7.67185E-57 |
| ZNF521 | 1.085385186 | 1.39112E-56 |
| C7 | 2.080960231 | 1.41983E-56 |
| MYO7B | 1.198789724 | 1.78205E-56 |
| TUNAR | 3.229910622 | 2.4111E-56 |
| DMD | 1.253700966 | 3.0959E-56 |
| PENK | 2.065259734 | 3.25006E-56 |
| AF001548.3 | 2.283077018 | 3.85188E-56 |
| BPI | 2.422747393 | 3.89784E-56 |
| ABCB1 | 1.116508275 | 4.9005E-56 |
| TSHZ2 | 1.188923502 | 7.6596E-56 |
| LINC02660 | 1.954351621 | 8.80178E-56 |
| IL17RD | 1.040761476 | 9.52931E-56 |
| ABCA9 | 1.334420201 | 1.93302E-55 |
| SFRP1 | 2.092560259 | 1.98022E-55 |
| AC096921.2 | 1.011858379 | 2.50023E-55 |
| AL109741.1 | 1.131028265 | 4.74046E-55 |
| RASGEF1C | 2.269431666 | 4.83565E-55 |
| AC055874.1 | 3.205090841 | 4.96307E-55 |
| AC124852.1 | 1.485568491 | 6.16296E-55 |
| PAK3 | 1.405374145 | 2.11212E-54 |
| C8orf88 | 1.125215315 | 3.12129E-54 |
| GEM | 1.025986376 | 3.70428E-54 |
| LYPLAL1-AS1 | 1.455749661 | 3.92544E-54 |
| AC016735.1 | 2.434175303 | 4.31148E-54 |
| IL33 | 1.531822436 | 4.53113E-54 |
| FGFBP2 | 1.923136934 | 8.14993E-54 |
| MAMDC2 | 1.294498873 | 1.05199E-53 |
| AC007368.1 | 2.904391314 | 1.14596E-53 |
| RHOXF1-AS1 | 1.676485057 | 1.54956E-53 |
| FMO2 | 1.52295001 | 1.70526E-53 |
| LINC00460 | 1.684583886 | 3.12284E-53 |
| DNM3 | 1.013954987 | 3.27176E-53 |
| AC015656.1 | 2.061622569 | 6.78161E-53 |
| CH25H | 1.18220472 | 7.21712E-53 |
| JPH4 | 1.170368222 | 7.51101E-53 |
| AC068733.3 | 1.686133941 | 8.55458E-53 |
| MEF2C-AS1 | 1.131650094 | 1.09736E-52 |
| MEOX2 | 1.349221351 | 1.23485E-52 |
| TRPM3 | 1.339239823 | 1.58318E-52 |
| TMEM132C | 2.031921498 | 3.39851E-52 |
| EPHB1 | 1.428021338 | 3.43545E-52 |
| SEZ6 | -2.535512416 | 3.96586E-52 |
| SELP | 1.459265284 | 4.92769E-52 |
| LRRC4B | 1.301172871 | 6.42008E-52 |
| TTYH1 | 2.165825714 | 7.35171E-52 |
| PI16 | 2.093238139 | 9.17382E-52 |
| SRL | 1.044450269 | 1.0595E-51 |
| FAM180B | 1.759023634 | 1.07018E-51 |
| RNF186 | 2.628227478 | 1.11781E-51 |
| LPL | 1.449337767 | 1.22855E-51 |
| TSBP1-AS1 | 1.318738836 | 1.78484E-51 |
| ORMDL3 | -1.161093169 | 2.10676E-51 |
| LCN10 | 2.505609595 | 2.77661E-51 |
| NFIA-AS2 | 1.354306903 | 3.56558E-51 |
| ALDH1A2 | 1.276736917 | 4.04951E-51 |
| PDK4 | 1.36757634 | 4.76956E-51 |
| KRT14 | 2.292560818 | 5.0465E-51 |
| AC021683.2 | 1.941347065 | 5.05273E-51 |
| AP000662.1 | 1.325997251 | 8.49518E-51 |
| MAB21L1 | 1.254905244 | 9.27348E-51 |
| AC021683.1 | 1.955264756 | 1.22667E-50 |
| TAC1 | 2.157359534 | 1.25967E-50 |
| SNHG26 | 1.420000086 | 1.43864E-50 |
| SYN2 | 1.4702797 | 1.53393E-50 |
| PGM5P4 | 1.728049008 | 1.60809E-50 |
| AP003071.4 | 1.20492529 | 1.70381E-50 |
| FAM171A1 | 1.228227368 | 2.09539E-50 |
| CNKSR2 | 1.413748338 | 4.19607E-50 |
| C10orf90 | 2.099311965 | 4.62785E-50 |
| FCER1A | 1.432730294 | 4.95569E-50 |
| LEPR | 1.00796464 | 7.52808E-50 |
| DACT2 | 1.887891933 | 1.01702E-49 |
| AL160412.1 | 2.506080444 | 1.14253E-49 |
| OXTR | 1.494306323 | 1.22463E-49 |
| CX3CL1 | 1.260649038 | 1.406E-49 |
| KRT79 | 2.324528646 | 1.41833E-49 |
| UNC13A | -1.769513417 | 1.48219E-49 |
| EGR1 | 1.249245629 | 2.09204E-49 |
| AL031651.2 | 1.353620711 | 2.09542E-49 |
| HSPB6 | 1.475540522 | 2.39028E-49 |
| LINC01305 | 1.921731033 | 2.40719E-49 |
| PLAGL1 | 1.036023306 | 2.57346E-49 |
| CHRDL2 | 1.811989031 | 2.59286E-49 |
| ADAMTS3 | 1.15247381 | 2.71749E-49 |
| SRSF12 | 1.588325081 | 2.76121E-49 |
| OPRPN | 3.493931398 | 3.60603E-49 |
| FGG | -3.824812128 | 4.44217E-49 |
| BMP6 | 1.212483842 | 4.45254E-49 |
| GIPC2 | 1.117336427 | 4.79994E-49 |
| CDC20P1 | 1.112625524 | 4.87009E-49 |
| KCNIP1 | 1.262449163 | 4.94332E-49 |
| PSMD3 | -1.068337142 | 7.75558E-49 |
| SYT8 | 2.144387913 | 9.31863E-49 |
| DCDC2C | 2.197748003 | 1.42277E-48 |
| XPNPEP2 | 1.368639053 | 1.98484E-48 |
| RLBP1 | 2.946447834 | 2.34021E-48 |
| CCN1 | 1.02877113 | 2.4442E-48 |
| CSN1S1 | 3.782004792 | 2.58884E-48 |
| LRRTM2 | 1.285404426 | 3.27757E-48 |
| NDRG2 | 1.030846565 | 3.96451E-48 |
| CXCL2 | 1.600741684 | 5.36579E-48 |
| MRGPRF | 1.03701849 | 6.49452E-48 |
| AGTR2 | 3.522155874 | 9.82713E-48 |
| CNTNAP3 | 1.542238195 | 1.19653E-47 |
| GFRA3 | 2.107639207 | 1.46674E-47 |
| KCNIP2 | 1.327600636 | 2.25699E-47 |
| AL391807.1 | 1.794505866 | 2.28192E-47 |
| TSPAN11 | 1.053288457 | 2.67064E-47 |
| AC027601.2 | 1.055157836 | 3.08841E-47 |
| RUNDC3A | -1.842962244 | 3.69037E-47 |
| LCN6 | 2.422629769 | 3.71954E-47 |
| PGM5P4-AS1 | 1.830698731 | 3.9822E-47 |
| STAC | 1.601909188 | 4.04977E-47 |
| MAS1L | 1.875866121 | 4.42121E-47 |
| NBAT1 | 1.241031957 | 5.15911E-47 |
| SCHIP1 | 1.09841364 | 5.70953E-47 |
| SHC4 | 1.96810992 | 5.71466E-47 |
| LINC00707 | 2.405271633 | 6.57959E-47 |
| PDE9A | 1.18051369 | 8.19083E-47 |
| MASP1 | 1.613025144 | 8.57731E-47 |
| TDRD10 | 1.134725612 | 1.07384E-46 |
| MT1JP | 2.475485901 | 1.20208E-46 |
| CPXM1 | 1.164648073 | 1.34676E-46 |
| LEP | 2.298851978 | 1.57506E-46 |
| NPAS4 | 1.578594065 | 1.79921E-46 |
| ZFP36 | 1.023612602 | 2.31246E-46 |
| KRT1 | 2.295244079 | 2.31407E-46 |
| ITPRIPL1 | 1.043405223 | 2.77794E-46 |
| NSG2 | -2.279109703 | 4.11209E-46 |
| LRFN5 | 1.331798816 | 4.43742E-46 |
| HPSE2 | 1.743210102 | 9.13961E-46 |
| LINC01013 | 1.180654543 | 1.12885E-45 |
| C1QL2 | 2.674273817 | 1.16849E-45 |
| SOX10 | 2.389200284 | 1.27285E-45 |
| AC044810.2 | 2.055496734 | 1.93459E-45 |
| TESC | 1.171174309 | 2.03699E-45 |
| POMC | 1.525341138 | 2.6316E-45 |
| CCDC178 | 1.385276139 | 2.86048E-45 |
| AL158055.1 | 1.440347801 | 2.95738E-45 |
| NIPAL4 | 1.684232977 | 2.9619E-45 |
| DEFB124 | 1.954251975 | 4.02885E-45 |
| ROCR | 2.571133496 | 4.78116E-45 |
| LORICRIN | 2.551839089 | 5.13274E-45 |
| CRB2 | 1.157938887 | 5.14112E-45 |
| PRELP | 1.233898829 | 5.30985E-45 |
| RASGRP2 | 1.120517921 | 5.75873E-45 |
| IGFN1 | 1.812379341 | 6.40323E-45 |
| ADH4 | 2.0390024 | 6.47431E-45 |
| SLIT1 | -1.860523934 | 7.03406E-45 |
| CD1C | 1.3790284 | 7.5614E-45 |
| DCT | 1.705002862 | 7.78028E-45 |
| IL6-AS1 | 1.589419985 | 7.78028E-45 |
| PGM5P3-AS1 | 1.75686816 | 9.64142E-45 |
| HS3ST4 | 2.600715205 | 9.83704E-45 |
| XKR7 | -2.543351336 | 9.91876E-45 |
| AC005682.1 | 1.321939994 | 1.15965E-44 |
| CLEC2L | 1.844801851 | 1.39201E-44 |
| KBTBD12 | 2.288221796 | 2.1793E-44 |
| CCN3 | 1.074757706 | 2.38216E-44 |
| AC007461.1 | 2.207821461 | 2.40699E-44 |
| AC025470.2 | 2.063048619 | 3.16712E-44 |
| PTGS2 | 1.564473463 | 3.24242E-44 |
| LGR6 | 1.777704682 | 3.50011E-44 |
| ADAMTS1 | 1.043114858 | 5.94758E-44 |
| CA4 | 2.168012013 | 6.39324E-44 |
| LINC01985 | 2.109811694 | 8.70921E-44 |
| LINC02599 | 1.336382322 | 1.19616E-43 |
| AL365434.2 | 2.551400641 | 1.38774E-43 |
| RN7SL3 | -2.330030091 | 1.5002E-43 |
| HIF3A | 1.668887388 | 1.65467E-43 |
| TP63 | 1.506759333 | 1.75954E-43 |
| GPM6B | 1.069182934 | 1.77679E-43 |
| SNORD15B | -1.934488317 | 2.3679E-43 |
| AL121821.2 | 1.236389395 | 2.38255E-43 |
| AC010976.2 | 1.386422056 | 2.97995E-43 |
| TNMD | 1.748874448 | 3.06106E-43 |
| NR4A3 | 1.304589705 | 3.35127E-43 |
| AP001021.1 | 1.766060309 | 3.55019E-43 |
| MIEN1 | -1.227985543 | 3.9877E-43 |
| AC026329.1 | 1.465262617 | 4.13084E-43 |
| MMP27 | 1.540808211 | 4.19488E-43 |
| KCNH8 | 1.69529017 | 5.45658E-43 |
| AC016924.1 | 1.632489808 | 7.44969E-43 |
| CCL23 | 1.259937106 | 7.99027E-43 |
| AC024337.2 | 1.206468064 | 9.43342E-43 |
| AC013553.3 | 1.293801094 | 9.7043E-43 |
| ABCB5 | 1.685790907 | 1.12865E-42 |
| CLCA4 | 2.018473227 | 1.3635E-42 |
| OR7C1 | 1.318602413 | 1.39861E-42 |
| AP001107.5 | 1.248027513 | 1.42242E-42 |
| LINC01028 | 2.619765842 | 1.62558E-42 |
| ADRA1A | 1.567508553 | 1.63315E-42 |
| SLC17A7 | 1.026098321 | 1.7011E-42 |
| TNNI2 | 1.435482534 | 2.0711E-42 |
| DBX2 | 1.403626664 | 2.07475E-42 |
| GABRA5 | 2.773081139 | 2.4174E-42 |
| KLK5 | 2.376446823 | 3.5008E-42 |
| TNNT3 | 1.586222778 | 4.7314E-42 |
| ZNF560 | 2.61896907 | 5.56023E-42 |
| HLF | 1.256214489 | 5.70702E-42 |
| TCF23 | 1.434188099 | 6.63636E-42 |
| LIN28A | -3.065489862 | 6.847E-42 |
| FAT2 | 1.601915983 | 7.09125E-42 |
| AC090004.2 | 1.28485898 | 8.44536E-42 |
| GDAP1 | -1.243274018 | 8.85832E-42 |
| AL139241.1 | 2.216212735 | 9.41095E-42 |
| IGHA2 | 1.73838539 | 1.77268E-41 |
| LINC00924 | 1.103971688 | 1.77268E-41 |
| ASTN1 | 1.506470204 | 1.9211E-41 |
| HOTAIRM1 | 1.041825621 | 2.39644E-41 |
| CALN1 | 1.682813904 | 2.42059E-41 |
| RPE65 | 2.028401822 | 2.58841E-41 |
| HFM1 | 1.341308398 | 3.24889E-41 |
| CPA1 | 1.720635041 | 3.28285E-41 |
| FOSB | 1.577748404 | 3.29063E-41 |
| AC025614.2 | 1.93779734 | 3.35294E-41 |
| AL135841.1 | 2.105978484 | 3.52098E-41 |
| HEPACAM | 1.95698733 | 4.36373E-41 |
| DSC3 | 1.890811078 | 4.67132E-41 |
| FOSL1 | 1.363111842 | 4.78515E-41 |
| GALNT8 | 1.275632559 | 5.30656E-41 |
| ADM5 | 1.079189967 | 5.5688E-41 |
| DNASE1L3 | 1.296974635 | 7.30648E-41 |
| AL445250.1 | 1.144777031 | 8.10212E-41 |
| SLC7A10 | 1.715594141 | 1.03491E-40 |
| PCOLCE2 | 1.613988316 | 1.13864E-40 |
| AFF2 | 1.205361126 | 1.36065E-40 |
| LINC00165 | 1.871126038 | 1.69338E-40 |
| KLF15 | 1.149540699 | 3.34603E-40 |
| KCNA6 | 1.386873311 | 4.03745E-40 |
| AP000851.1 | 2.302645618 | 4.99419E-40 |
| LINC02587 | 1.451160233 | 5.80332E-40 |
| KLHL33 | 1.017516626 | 5.8288E-40 |
| MGAM2 | 2.010171619 | 5.96653E-40 |
| AP005264.6 | 1.713360164 | 7.37504E-40 |
| AL136985.3 | 1.065558093 | 7.85415E-40 |
| KLHL34 | 2.279188668 | 9.17635E-40 |
| VIT | 1.350630711 | 1.00082E-39 |
| PXDNL | -1.553460117 | 1.03674E-39 |
| CCNYL2 | 2.593605595 | 1.18278E-39 |
| SERPINE2 | 1.009138982 | 1.21559E-39 |
| FXYD2 | 1.243219632 | 1.37649E-39 |
| AC080188.2 | 1.345349801 | 1.40846E-39 |
| SCN2B | 1.463554333 | 1.71827E-39 |
| TMPRSS5 | 1.42821615 | 1.75248E-39 |
| AC104117.3 | 1.09080366 | 1.82566E-39 |
| SNAP25 | -1.662939244 | 1.90216E-39 |
| HAPLN3 | 1.227498184 | 1.93412E-39 |
| FOXC1 | 1.522127109 | 2.15002E-39 |
| RGR | 2.864394789 | 2.39548E-39 |
| CACNA1G | 1.317676524 | 2.77567E-39 |
| LIF | 1.166254908 | 3.25146E-39 |
| LINC00163 | 2.296284003 | 3.85298E-39 |
| PLIN4 | 1.673589894 | 6.17059E-39 |
| AL049775.1 | 1.090987773 | 6.29698E-39 |
| MMP23B | 1.389683373 | 6.39311E-39 |
| B4GALNT2 | -2.087115793 | 7.51368E-39 |
| FOXG1 | 3.1534749 | 7.7433E-39 |
| FAM234B | -1.193963687 | 7.78349E-39 |
| AC004637.1 | 2.161996506 | 9.14988E-39 |
| AL109809.1 | 1.442208968 | 9.32662E-39 |
| DCHS2 | 1.570762491 | 1.02401E-38 |
| FGF9 | 1.691881471 | 1.08403E-38 |
| FXYD7 | 1.291057364 | 1.75296E-38 |
| AC110772.2 | 1.346220068 | 1.8811E-38 |
| AL356123.2 | 2.302749357 | 2.46279E-38 |
| KCNIP1-OT1 | 2.036272888 | 2.47113E-38 |
| NRG2 | 1.403597631 | 3.23082E-38 |
| CRYAB | 1.483674968 | 3.50725E-38 |
| KANK4 | 1.314207659 | 4.00066E-38 |
| RDH5 | 1.042514792 | 4.05623E-38 |
| LRRC3B | 1.651727687 | 4.80749E-38 |
| LAMC3 | 1.128944555 | 5.7423E-38 |
| COL26A1 | 1.581183172 | 5.92512E-38 |
| NFIB | 1.01444289 | 5.99416E-38 |
| HSPB7 | 1.17228651 | 6.03533E-38 |
| ANGPTL1 | 1.169891718 | 6.19691E-38 |
| PSAT1P3 | 1.295825687 | 6.47689E-38 |
| CLEC10A | 1.317815426 | 7.2266E-38 |
| ROPN1 | 2.677910548 | 8.61322E-38 |
| SMIM10L2A | 1.111380374 | 8.95333E-38 |
| DLX5 | 1.503473364 | 9.37495E-38 |
| HOXA2 | 1.221795391 | 1.0036E-37 |
| TMEM151B | 1.619996318 | 1.03674E-37 |
| COL27A1 | 1.001241515 | 1.06739E-37 |
| REM1 | 1.00568477 | 1.16488E-37 |
| PHYHIP | 1.039013291 | 1.52911E-37 |
| AC025280.2 | 1.271008883 | 1.57275E-37 |
| INSYN2B | 1.485786576 | 1.73707E-37 |
| OLAH | 2.029193923 | 2.20521E-37 |
| OR10A3 | 2.265229235 | 2.59089E-37 |
| TP53AIP1 | 1.284288108 | 3.46904E-37 |
| CA3 | 1.447850147 | 3.81739E-37 |
| LINC02511 | 1.760721651 | 4.35364E-37 |
| LINC02461 | 1.557488451 | 4.53229E-37 |
| CPA6 | -2.136948256 | 5.50645E-37 |
| AL354793.1 | 1.725945179 | 5.93221E-37 |
| IGF2BP3 | 1.902811607 | 7.27143E-37 |
| LINC00377 | 2.00786822 | 7.65553E-37 |
| AC018445.5 | 1.115856704 | 7.99113E-37 |
| NR4A1 | 1.141636685 | 9.68541E-37 |
| TMEM200C | 1.131203392 | 9.68541E-37 |
| ALPL | 1.128554383 | 1.1235E-36 |
| VIM-AS1 | 1.225122193 | 1.18599E-36 |
| IL6 | 1.481927327 | 1.42525E-36 |
| ENPP6 | 1.088724484 | 1.44735E-36 |
| GPD1 | 1.645255782 | 2.5136E-36 |
| SYP | -1.421112209 | 3.41199E-36 |
| HAS1 | 1.544469524 | 3.59855E-36 |
| SCN3A | 1.278579927 | 3.62499E-36 |
| KRT17P1 | 1.957087317 | 4.20897E-36 |
| AL390755.2 | 1.93549774 | 4.93311E-36 |
| CELF3 | -1.57488476 | 6.50548E-36 |
| AC004554.1 | 1.129500662 | 6.50548E-36 |
| SEZ6L | -1.988073025 | 6.75283E-36 |
| NPFFR1 | 1.447368352 | 6.82336E-36 |
| HACD1 | 1.123628117 | 8.35617E-36 |
| MYOCD | 1.191582158 | 1.04396E-35 |
| CSRNP3 | 1.026875223 | 1.05508E-35 |
| BMPER | 1.366472557 | 1.16015E-35 |
| AC073365.1 | 2.044103925 | 1.2829E-35 |
| TMEM72-AS1 | 1.105117686 | 1.53783E-35 |
| DES | 1.654761888 | 1.57797E-35 |
| AL021408.1 | 1.302554953 | 1.868E-35 |
| KLF14 | 1.38869241 | 1.868E-35 |
| EXTL1 | 1.48009356 | 2.17874E-35 |
| BCL11A | 1.586232875 | 2.39719E-35 |
| AC107294.3 | 1.023527774 | 2.46113E-35 |
| PRSS33 | 3.129681097 | 3.44735E-35 |
| NTF4 | 1.469584621 | 4.03426E-35 |
| EN1 | 1.767961298 | 4.14404E-35 |
| EDN1 | 1.029214967 | 4.28433E-35 |
| ABCA13 | 2.159135886 | 4.62189E-35 |
| HOXA3 | 1.04233941 | 5.12844E-35 |
| KRT17 | 1.745661053 | 5.41119E-35 |
| DNM1P46 | 1.09053139 | 5.41119E-35 |
| TEX19 | -1.802149991 | 5.68117E-35 |
| BMP2 | 1.112887934 | 6.62946E-35 |
| MIR99AHG | 1.052928438 | 7.16555E-35 |
| AC006963.1 | 1.705616063 | 7.31752E-35 |
| LINC01883 | 2.906494925 | 1.06029E-34 |
| AL031587.1 | 1.984824236 | 1.06707E-34 |
| BTNL9 | 1.069683412 | 1.10246E-34 |
| KLHL13 | 1.15711965 | 1.16079E-34 |
| AL591368.1 | 1.347429277 | 1.20459E-34 |
| HSD17B13 | 1.328008702 | 1.2413E-34 |
| VWC2 | 1.664161409 | 1.39721E-34 |
| COL19A1 | 1.990314338 | 1.50462E-34 |
| TRIM36 | -1.260976571 | 1.51484E-34 |
| ST3GAL6-AS1 | 1.018288395 | 1.6757E-34 |
| AC145098.2 | 1.031399443 | 1.69314E-34 |
| CSN2 | 6.153907084 | 1.70375E-34 |
| COLCA2 | 1.16198841 | 2.00135E-34 |
| STAC2 | 1.993939889 | 2.26482E-34 |
| PLIN1 | 1.600431694 | 2.45406E-34 |
| FRMD3 | 1.03091579 | 2.81601E-34 |
| CDK5R2 | -2.460697331 | 3.17744E-34 |
| PNMA3 | 1.632987777 | 3.53814E-34 |
| AP001360.1 | 1.999527723 | 6.35856E-34 |
| PLCXD3 | 1.499945113 | 6.39803E-34 |
| SERTM2 | 1.634838753 | 7.06459E-34 |
| TNN | 1.217248794 | 7.37279E-34 |
| GABRP | 2.031149518 | 7.52548E-34 |
| EDDM13 | 1.030298208 | 8.26589E-34 |
| SLC35F3 | 1.490707929 | 8.69183E-34 |
| HYMAI | 1.75381121 | 8.87698E-34 |
| EPO | -1.786986044 | 1.3121E-33 |
| GREM2 | 1.28103888 | 1.43107E-33 |
| HNRNPA1P33 | 1.740805275 | 1.8115E-33 |
| PLA2G4A | 1.066094397 | 2.01925E-33 |
| AC084759.3 | 1.825530264 | 2.24312E-33 |
| ALDH1L1 | 1.349165731 | 2.41463E-33 |
| CLK3P2 | 2.05244087 | 2.45106E-33 |
| NIBAN3 | 1.385080177 | 2.55295E-33 |
| AL591222.1 | 1.571276462 | 2.63639E-33 |
| SFRP5 | 2.08291165 | 2.83422E-33 |
| DNM1P51 | 1.10618167 | 3.02124E-33 |
| CDH17 | 1.043943795 | 3.04903E-33 |
| AC104237.3 | 1.79343788 | 3.0593E-33 |
| MEG9 | 1.247998421 | 3.08714E-33 |
| VSTM5 | 1.355444543 | 3.40181E-33 |
| ARMT1 | -1.013035688 | 3.40564E-33 |
| BNC1 | 1.572584245 | 3.51373E-33 |
| AC009951.6 | 1.084435285 | 3.65405E-33 |
| MT1X | 1.020779983 | 3.78276E-33 |
| RGS2 | 1.040037436 | 3.83194E-33 |
| TRGC1 | -1.578780811 | 4.22067E-33 |
| AC005165.1 | 1.431975633 | 5.12108E-33 |
| AL445437.1 | 1.333203716 | 5.3552E-33 |
| IL1R2 | 1.223050811 | 5.63176E-33 |
| FZD9 | 1.733740317 | 5.87101E-33 |
| RTBDN | -2.011072858 | 5.88932E-33 |
| AC104088.3 | 1.50204512 | 5.89772E-33 |
| MMP7 | 1.591220266 | 6.93069E-33 |
| APCDD1L | 1.361104302 | 8.82548E-33 |
| NOS1 | 1.347067556 | 9.24097E-33 |
| TRIM29 | 1.46464892 | 9.27666E-33 |
| KCNQ4 | 1.278859611 | 1.01846E-32 |
| CDH12 | 2.006513833 | 1.14599E-32 |
| FOXC2 | 1.236458302 | 1.18924E-32 |
| RANBP20P | 2.504205204 | 1.23548E-32 |
| CYP4F24P | 1.814074945 | 1.4151E-32 |
| AC053545.1 | 1.423983032 | 1.88043E-32 |
| CCL21 | 1.553356086 | 1.88219E-32 |
| AC100810.7 | 1.919922939 | 2.06776E-32 |
| AC009242.1 | 1.160129465 | 2.36797E-32 |
| THSD7B | 1.072780194 | 2.45155E-32 |
| KCNE1B | 1.651565679 | 2.64431E-32 |
| AC091078.1 | 1.780341286 | 2.65398E-32 |
| QRFPR | 1.787733427 | 3.12606E-32 |
| AC105398.1 | 2.349496995 | 3.57382E-32 |
| AC018445.1 | 2.53187682 | 3.66072E-32 |
| DLX6-AS1 | 1.975574281 | 3.93285E-32 |
| AC092535.5 | 1.018407687 | 4.09684E-32 |
| CDH22 | 1.558536544 | 4.28798E-32 |
| PRSS3 | 1.775187734 | 4.51069E-32 |
| AC068057.1 | 2.305012275 | 5.40233E-32 |
| KCNS2 | 1.19071057 | 5.55293E-32 |
| AC018697.1 | 1.968587099 | 5.83104E-32 |
| PLCZ1 | 1.864212054 | 5.83561E-32 |
| ALK | 1.312162256 | 5.86661E-32 |
| AC090136.3 | 1.145391902 | 6.30998E-32 |
| RNASE7 | 1.523158161 | 6.84726E-32 |
| RFLNA | 1.217710999 | 7.05115E-32 |
| AC027329.1 | 2.024939748 | 7.68358E-32 |
| CHI3L1 | 1.488742374 | 7.71123E-32 |
| AL133346.1 | 1.214444638 | 8.3405E-32 |
| AC011239.2 | 1.160934912 | 9.9175E-32 |
| FFAR2 | -1.271213518 | 1.01523E-31 |
| ZNF488 | 1.306417689 | 1.30208E-31 |
| ST8SIA1 | 1.056437052 | 1.38715E-31 |
| TEX26-AS1 | 1.391459249 | 1.39447E-31 |
| AC007671.1 | 1.087739042 | 1.46301E-31 |
| LIPE | 1.078040281 | 1.48873E-31 |
| LALBA | 5.008502348 | 1.59466E-31 |
| RFPL4B | 1.632493089 | 1.59466E-31 |
| NUDT11 | 1.023686551 | 1.62383E-31 |
| SLC19A3 | 1.45812134 | 1.62585E-31 |
| UGT8 | 1.685932075 | 1.62837E-31 |
| LINC00968 | 1.126003342 | 1.82926E-31 |
| TUBB2B | 1.355950372 | 1.95107E-31 |
| GOLGA8VP | 1.067284821 | 2.33858E-31 |
| LINC02594 | 1.564987632 | 2.41027E-31 |
| GATA6-AS1 | 1.300182927 | 2.45976E-31 |
| CD8B2 | 1.449882056 | 3.06217E-31 |
| AL606500.1 | -2.033644355 | 3.37508E-31 |
| CALB2 | 1.430908444 | 3.77542E-31 |
| NGF-AS1 | 1.831794194 | 3.89119E-31 |
| ADGRG2 | 1.206023746 | 3.91607E-31 |
| OTC | 2.204029797 | 4.39153E-31 |
| LINC00445 | 1.770571725 | 4.76516E-31 |
| PROX1 | 1.158658041 | 5.27938E-31 |
| PTCHD1 | 1.714117531 | 6.98228E-31 |
| SLC7A14 | 1.536587966 | 8.75527E-31 |
| KLK6 | 2.121322691 | 1.06737E-30 |
| VGLL1 | 2.29042578 | 1.07252E-30 |
| CIDEC | 1.766692729 | 1.34194E-30 |
| AL137026.1 | 1.138878448 | 1.62029E-30 |
| LINC01356 | 1.235260438 | 1.7283E-30 |
| OR10A6 | 2.094829647 | 1.78305E-30 |
| LINC01668 | 1.615118353 | 1.78305E-30 |
| NTRK3 | 1.387308954 | 1.9077E-30 |
| SBK2 | -1.979186844 | 2.31286E-30 |
| AC113167.1 | 1.698987942 | 2.33091E-30 |
| LGR5 | 1.496714717 | 2.8313E-30 |
| OR2L13 | 1.683920828 | 2.87353E-30 |
| AP001476.1 | 1.240295087 | 3.24878E-30 |
| RYR1 | 1.140358151 | 3.32819E-30 |
| AL035425.3 | 2.087165908 | 3.45193E-30 |
| H4C14 | -1.036784378 | 3.62065E-30 |
| AC005550.2 | 1.978390275 | 3.6296E-30 |
| FXYD1 | 1.629334502 | 3.78863E-30 |
| AC006058.1 | 1.605732085 | 3.95204E-30 |
| AC013565.1 | 1.289270447 | 3.95239E-30 |
| MRGPRF-AS1 | 1.407855842 | 3.97572E-30 |
| ATP4A | 1.654915924 | 4.10986E-30 |
| PTGDS | 1.246234383 | 4.4562E-30 |
| GRIK1-AS1 | 1.531452443 | 4.51979E-30 |
| AP003355.2 | 1.538996421 | 5.55972E-30 |
| AC024940.1 | 1.668574488 | 6.10426E-30 |
| NCAM1 | 1.26536186 | 6.45797E-30 |
| AC079768.4 | 2.475485675 | 7.10517E-30 |
| CEACAM5 | -1.948819275 | 8.28037E-30 |
| FDCSP | 2.317015966 | 8.75138E-30 |
| AC005833.2 | 1.199434437 | 8.96673E-30 |
| SLC8A3 | 1.042330129 | 1.00096E-29 |
| CDHR1 | 1.2238182 | 1.10674E-29 |
| AC104237.2 | 1.816841492 | 1.19382E-29 |
| PRSS12 | 1.518905188 | 1.3626E-29 |
| ANKRD35 | 1.025393937 | 1.54359E-29 |
| NCKAP5-AS2 | 1.139721594 | 1.54932E-29 |
| AC011008.2 | 1.101759244 | 1.67213E-29 |
| HOXA4 | 1.055592491 | 1.90075E-29 |
| PACERR | 1.542182877 | 1.90075E-29 |
| AL390755.1 | 1.206675643 | 1.90078E-29 |
| AL359732.1 | 1.830440801 | 1.9725E-29 |
| TCEAL2 | 1.496234068 | 2.1519E-29 |
| L3MBTL4 | 1.07447567 | 2.52084E-29 |
| AC245884.9 | 2.044337124 | 3.00106E-29 |
| NEFM | 1.320982507 | 3.20106E-29 |
| TMEM252 | 1.307342445 | 4.63245E-29 |
| LINC01785 | 3.251726644 | 4.67839E-29 |
| SMR3B | 3.297109986 | 4.69442E-29 |
| CFHR1 | 1.872361545 | 4.96089E-29 |
| SOSTDC1 | 1.640493618 | 5.02924E-29 |
| SCRT1 | -1.314607996 | 5.42233E-29 |
| GRB7 | -1.082325118 | 6.34543E-29 |
| PANTR1 | 2.003143735 | 7.31958E-29 |
| NPM2 | 1.150374665 | 7.58132E-29 |
| SCN7A | 1.34780773 | 8.86543E-29 |
| AC016026.1 | 1.090549247 | 9.71082E-29 |
| AC092155.1 | 1.574748728 | 9.96441E-29 |
| AC188617.2 | 1.864748259 | 1.00898E-28 |
| HSD11B1-AS1 | 1.297169085 | 1.15276E-28 |
| LINC00839 | 1.323585764 | 1.4147E-28 |
| AC015660.1 | 1.233476924 | 1.63737E-28 |
| KRT17P8 | 1.746498213 | 1.74496E-28 |
| AC012101.2 | 1.026169518 | 1.75458E-28 |
| AL117372.1 | 3.33271209 | 1.8676E-28 |
| TMCC2 | 1.095105607 | 1.97827E-28 |
| GLP1R | 1.405549682 | 2.03955E-28 |
| ADGRB3 | 1.113767752 | 3.1723E-28 |
| ANTXRL | 1.82380457 | 3.3634E-28 |
| CAVIN2-AS1 | 1.965873054 | 3.62536E-28 |
| AL158828.1 | 1.891502502 | 3.68612E-28 |
| FGF2 | 1.093397637 | 3.77993E-28 |
| LIPG | 1.076483831 | 3.93237E-28 |
| AC119424.1 | 1.826404616 | 5.45183E-28 |
| C1QTNF9 | 1.118768981 | 6.07726E-28 |
| PICSAR | 1.697545988 | 6.37693E-28 |
| ACTL6B | -2.216733752 | 6.94093E-28 |
| MYOZ1 | 1.094763651 | 7.30597E-28 |
| DSG3 | 1.851510391 | 7.47696E-28 |
| ROR1-AS1 | 1.190231962 | 8.44955E-28 |
| AQP7 | 1.410764749 | 8.65183E-28 |
| SOX6 | 1.238308358 | 9.03604E-28 |
| OPRK1 | 2.210066292 | 9.83322E-28 |
| AC243562.1 | 1.084550235 | 9.97199E-28 |
| ACAN | 1.186835968 | 1.05751E-27 |
| SLC16A12 | 1.321345769 | 1.13418E-27 |
| UTS2R | 1.257088117 | 1.14667E-27 |
| AC021188.1 | 1.070357102 | 1.21015E-27 |
| CACNA1C-AS2 | 1.226723212 | 1.23006E-27 |
| U82695.1 | 1.214714288 | 1.27074E-27 |
| GPR182 | 1.001328912 | 1.33833E-27 |
| ZP4 | 2.601066625 | 1.42833E-27 |
| LINC01697 | 1.476769053 | 1.59065E-27 |
| OR2B11 | 1.099406124 | 1.59617E-27 |
| AC110048.2 | 1.115118468 | 1.69248E-27 |
| FCER2 | 1.748839507 | 1.8239E-27 |
| FABP4 | 1.531124952 | 2.05139E-27 |
| GDF7 | 1.039996998 | 2.17627E-27 |
| RN7SL417P | 1.445006003 | 2.22416E-27 |
| CCL24 | 1.530742216 | 2.52884E-27 |
| LINC02732 | 2.164133615 | 2.58319E-27 |
| LINC01354 | 1.11074961 | 2.64303E-27 |
| GDPD3 | -1.130809916 | 2.65681E-27 |
| AC106799.1 | 2.117885254 | 2.7129E-27 |
| SLC27A6 | 1.380361813 | 2.97776E-27 |
| ASB12 | 1.07482144 | 3.17374E-27 |
| AC012409.3 | 1.128016932 | 3.3255E-27 |
| AC107294.1 | 1.135357515 | 3.36564E-27 |
| SIX3 | 1.824038771 | 3.36868E-27 |
| KRT17P3 | 1.858656407 | 3.74826E-27 |
| ERBB2 | -1.091650828 | 3.84031E-27 |
| CENPVL3 | 2.066514668 | 3.99559E-27 |
| CLEC9A | 1.011307136 | 4.05652E-27 |
| AC108477.1 | 1.057684307 | 4.2809E-27 |
| PCARE | 1.871553925 | 4.3739E-27 |
| FOXCUT | 2.035604633 | 5.15116E-27 |
| AL122008.3 | 1.706996041 | 5.61931E-27 |
| CSF3 | 1.850681348 | 5.9009E-27 |
| AL161668.4 | 1.639302234 | 5.95388E-27 |
| DLX6 | 1.941932098 | 6.54733E-27 |
| GPRIN2 | 1.359558686 | 6.81598E-27 |
| AC004687.3 | 1.708262731 | 7.36172E-27 |
| AKR1B10 | -1.782716327 | 7.62749E-27 |
| BVES-AS1 | 1.152585117 | 8.37497E-27 |
| CIDEA | 1.729640874 | 9.03895E-27 |
| PEX5L | -1.527616656 | 9.7194E-27 |
| SLITRK2 | 1.11417757 | 9.80384E-27 |
| C4orf51 | 1.719564781 | 1.1589E-26 |
| MIA | 1.592201527 | 1.18096E-26 |
| GLYAT | 1.629607722 | 1.18225E-26 |
| MLC1 | 1.43693269 | 1.19515E-26 |
| LINCR-0003 | 2.166141384 | 1.22171E-26 |
| SHISA3 | 1.278792688 | 1.30114E-26 |
| NPC1L1 | -1.242417594 | 1.30427E-26 |
| SPIB | 1.460994069 | 1.38514E-26 |
| AC078842.1 | 1.662321877 | 1.55811E-26 |
| HMCN2 | 1.005966823 | 1.56942E-26 |
| SNORA73B | -1.235013406 | 1.7239E-26 |
| AC015712.6 | 1.094843923 | 1.80899E-26 |
| RBP4 | 1.369685547 | 1.84504E-26 |
| LINC02668 | 1.672243115 | 1.86723E-26 |
| LINC00906 | 1.607799999 | 2.51088E-26 |
| BANCR | 1.719664057 | 2.69575E-26 |
| AQP7P1 | 1.490801544 | 2.70784E-26 |
| FGF12 | -1.061163032 | 2.86517E-26 |
| TPPP2 | 1.618389219 | 2.97306E-26 |
| AL121749.1 | 1.137445763 | 3.26146E-26 |
| PACRG-AS1 | 1.311565413 | 3.83432E-26 |
| SYT5 | -1.615779045 | 3.97127E-26 |
| DNM1P47 | 1.137778852 | 4.01964E-26 |
| KCNJ16 | 1.663932194 | 4.02514E-26 |
| LINC00032 | 1.478435499 | 4.03319E-26 |
| LINC00589 | -1.436887192 | 4.43963E-26 |
| AC027031.2 | 1.044262962 | 4.60039E-26 |
| RPS17P1 | 1.510528789 | 4.77128E-26 |
| OSTN | 2.167467102 | 4.8307E-26 |
| LINC01589 | 1.026091071 | 5.06999E-26 |
| AC069148.1 | 1.194668803 | 5.63756E-26 |
| KRT16P6 | 1.851811626 | 5.68747E-26 |
| FOS | 1.006198004 | 7.78879E-26 |
| CYP7A1 | 1.04611699 | 7.92806E-26 |
| AP000851.2 | 1.670525004 | 8.79429E-26 |
| LRTM2 | -1.795259945 | 9.71504E-26 |
| KCNE1 | 1.050106904 | 1.32313E-25 |
| SH3PXD2A-AS1 | 1.192536458 | 1.58144E-25 |
| AC023480.1 | 1.283521766 | 1.83709E-25 |
| MRGPRX7P | 2.021361172 | 1.97486E-25 |
| ABCA12 | -1.372105042 | 2.03473E-25 |
| COL6A6 | 1.071550686 | 2.03482E-25 |
| MARCHF4 | -1.205405676 | 2.10517E-25 |
| EGFEM1P | 1.28590974 | 2.23417E-25 |
| KRT6B | 1.755597671 | 2.24852E-25 |
| AL591686.1 | 1.741721479 | 2.83578E-25 |
| AC015522.1 | 2.424026925 | 2.91635E-25 |
| AL118505.1 | 1.424986083 | 2.99134E-25 |
| TRARG1 | 1.728370412 | 3.00706E-25 |
| FCRLB | -1.35707755 | 3.56527E-25 |
| AC018445.6 | 1.442783203 | 3.87744E-25 |
| AL136366.1 | 1.507510759 | 4.52877E-25 |
| GPR142 | 1.415659028 | 4.79401E-25 |
| G0S2 | 1.111498233 | 5.07243E-25 |
| SGCZ | 1.944273818 | 5.07775E-25 |
| MRGPRX2 | 1.877872267 | 5.43641E-25 |
| EGR3 | 1.029162448 | 5.92963E-25 |
| ST7-OT4 | 2.02401296 | 6.26788E-25 |
| CYP19A1 | 1.020032224 | 6.3387E-25 |
| AC103982.1 | 1.954764331 | 6.5912E-25 |
| C5orf34-AS1 | 1.011932288 | 6.94214E-25 |
| AC020907.1 | 1.505938337 | 7.06015E-25 |
| SLC47A1P2 | 1.417957143 | 7.49886E-25 |
| AJ011932.1 | 1.122863083 | 7.49886E-25 |
| KDM4E | 1.493021503 | 7.50765E-25 |
| LINC02169 | -2.644532356 | 9.70416E-25 |
| AC090826.1 | 1.329548118 | 9.70416E-25 |
| LMO7DN | 1.337771223 | 9.73094E-25 |
| SATB1-AS1 | 1.100002079 | 1.06876E-24 |
| PRKCQ-AS1 | 1.069590946 | 1.11091E-24 |
| IGF2-AS | 1.190695819 | 1.17418E-24 |
| CYP27C1 | 1.33444497 | 1.19896E-24 |
| ESRG | 2.17532973 | 1.26419E-24 |
| GRIA1 | -1.831504208 | 1.4851E-24 |
| CRLF1 | 1.163040395 | 1.56425E-24 |
| LINC02147 | 1.61346277 | 1.73349E-24 |
| FBN3 | 1.580606222 | 1.82294E-24 |
| DPYS | 1.104746528 | 2.00543E-24 |
| LINC02607 | 1.549034222 | 2.09869E-24 |
| AL132996.1 | 1.239341077 | 2.11679E-24 |
| CLEC4F | 1.152397431 | 2.34726E-24 |
| AL356489.2 | 1.1176546 | 2.62908E-24 |
| PLD5 | 1.376493501 | 2.77068E-24 |
| EFCAB1 | 1.212136976 | 2.8337E-24 |
| AL356417.2 | 1.037981236 | 2.9686E-24 |
| PCYT1B | 1.052298362 | 3.0212E-24 |
| KIF19 | 1.115982142 | 3.04972E-24 |
| WNT6 | 1.480522979 | 3.28024E-24 |
| EEF1A2 | -1.328821995 | 3.51933E-24 |
| SLC22A16 | 1.329576601 | 3.67804E-24 |
| GFRA2 | 1.018802906 | 3.75205E-24 |
| AC244157.2 | 1.668811173 | 4.07001E-24 |
| BLK | 1.393620373 | 4.46707E-24 |
| AC108457.1 | 2.001585953 | 4.77448E-24 |
| AC021134.1 | -2.42905014 | 5.55317E-24 |
| CHRNA9 | -1.763559928 | 5.87966E-24 |
| AL035670.1 | 1.335890864 | 5.92873E-24 |
| COL4A2-AS1 | 1.013370618 | 7.01019E-24 |
| CCNA1 | 1.124775267 | 7.05209E-24 |
| CHRM3-AS2 | 1.261578907 | 8.08362E-24 |
| SACS-AS1 | 1.438330407 | 8.33802E-24 |
| FGF17 | 1.208386876 | 8.41029E-24 |
| FMR1NB | -2.046784425 | 9.35046E-24 |
| AC092598.1 | -2.069682814 | 9.54321E-24 |
| GDF5 | 1.393673147 | 1.00418E-23 |
| C14orf180 | 1.719990701 | 1.04114E-23 |
| MRGPRX4 | 2.052647888 | 1.05294E-23 |
| AC000082.1 | 1.445615008 | 1.07697E-23 |
| ANGPTL5 | 1.19392611 | 1.14182E-23 |
| AC027449.1 | 1.209478998 | 1.15323E-23 |
| LINC02189 | 2.525944164 | 1.2909E-23 |
| HAP1 | 1.006997869 | 1.54143E-23 |
| XKR4 | 1.346596707 | 1.72095E-23 |
| AC009970.1 | 2.140778477 | 1.81515E-23 |
| AC080038.2 | 1.378417738 | 1.90743E-23 |
| CYP39A1 | 1.030419584 | 1.98506E-23 |
| MIR202HG | 1.602672944 | 2.10036E-23 |
| VTN | -1.147373698 | 2.60961E-23 |
| KY | 1.170112505 | 2.65198E-23 |
| CD1E | 1.057878286 | 2.70331E-23 |
| CAPZA3 | 1.776237188 | 2.76839E-23 |
| BEND4 | 1.424543767 | 2.89284E-23 |
| BX119927.1 | 1.44148352 | 3.22979E-23 |
| GNG13 | -1.392699393 | 3.2495E-23 |
| SNORA23 | -1.809808592 | 3.45427E-23 |
| SOX9-AS1 | 1.025730283 | 3.48251E-23 |
| SERPINA6 | -2.387033448 | 3.75157E-23 |
| THEGL | 1.542751252 | 4.55767E-23 |
| ADH1B | 1.570718355 | 4.84696E-23 |
| AC246817.2 | 1.076982275 | 5.81984E-23 |
| AL118556.1 | 1.256324543 | 5.82076E-23 |
| KRT17P6 | 1.555056996 | 6.34404E-23 |
| LINC00578 | -1.325077589 | 6.34548E-23 |
| PROX1-AS1 | 1.414280007 | 6.35119E-23 |
| OGN | 1.147506252 | 6.52274E-23 |
| WNT16 | 1.057095864 | 6.78417E-23 |
| TCAP | -1.190592771 | 6.86265E-23 |
| KCNK5 | 1.128750379 | 8.98727E-23 |
| LINC02752 | 1.10519058 | 9.35981E-23 |
| AL133476.1 | 1.613959462 | 9.7079E-23 |
| PRRG3 | 1.004256954 | 1.03097E-22 |
| THBS4 | 1.085269305 | 1.05462E-22 |
| AC062004.1 | 1.150946768 | 1.10099E-22 |
| AC021733.4 | 1.166410416 | 1.14439E-22 |
| AC011247.2 | 2.177972501 | 1.19651E-22 |
| FRG1FP | -1.598126884 | 1.26519E-22 |
| AC015712.7 | 1.116123616 | 1.28153E-22 |
| LINC02551 | -1.261469914 | 1.40211E-22 |
| AC244230.2 | 1.887215742 | 1.46682E-22 |
| PNMA6A | 1.03276389 | 1.50602E-22 |
| ORM1 | -1.889558587 | 1.56927E-22 |
| AC022784.1 | 1.501426399 | 1.58907E-22 |
| PNLDC1 | 1.251515991 | 1.59645E-22 |
| AIRE | 1.152100723 | 1.74889E-22 |
| ART3 | 1.884306139 | 1.82494E-22 |
| AP000821.2 | 1.489871718 | 2.04974E-22 |
| AC026124.1 | 1.885933003 | 2.21255E-22 |
| KRT8P22 | -1.371908226 | 2.25565E-22 |
| H2AC19 | -1.14075789 | 2.43661E-22 |
| RNY1 | 2.26568627 | 2.56499E-22 |
| AL138899.2 | 1.14482091 | 2.62141E-22 |
| AL353135.2 | 1.111639924 | 2.62776E-22 |
| AC018450.1 | 1.183277186 | 2.66421E-22 |
| LINC02207 | 1.002954237 | 2.73195E-22 |
| C10orf105 | 1.103878391 | 3.04221E-22 |
| LCNL1 | 1.327616318 | 3.05856E-22 |
| ZBTB8B | 1.38033525 | 3.12968E-22 |
| AC106873.1 | 2.877107121 | 3.35323E-22 |
| SLCO1A2 | 1.727832531 | 3.43244E-22 |
| ALX1 | 1.730761097 | 3.9753E-22 |
| CD200R1L-AS1 | 1.835645706 | 5.23445E-22 |
| MEG8 | 1.01273312 | 5.5212E-22 |
| RARRES2P1 | 1.497593982 | 5.56554E-22 |
| SMAD9-IT1 | 1.229789602 | 6.48275E-22 |
| COL4A2-AS2 | 1.554022206 | 6.58672E-22 |
| FSD1 | 1.262065054 | 6.79699E-22 |
| AL590434.1 | 1.206844412 | 6.84366E-22 |
| KLK1 | 1.077603297 | 6.90193E-22 |
| ABCC12 | -1.759126456 | 7.39791E-22 |
| Y_RNA | 1.046695474 | 7.49482E-22 |
| KRT81 | 1.756026584 | 7.78603E-22 |
| SLC1A2 | -1.068656806 | 8.05973E-22 |
| AMER3 | -2.332959007 | 8.11574E-22 |
| AP001626.1 | 1.370915461 | 8.42263E-22 |
| SLC30A8 | -2.020661957 | 8.47752E-22 |
| AC026316.5 | 1.717942064 | 8.55554E-22 |
| OR7A5 | 1.564866075 | 8.73159E-22 |
| NRG1 | 1.054462403 | 9.03764E-22 |
| AP002001.2 | -3.472973784 | 9.35339E-22 |
| AADACL2-AS1 | 1.173408819 | 9.4296E-22 |
| AL035045.1 | 1.551039803 | 9.63565E-22 |
| LDLRAD1 | -1.247303384 | 9.9782E-22 |
| OR2S2 | 2.369629792 | 1.11824E-21 |
| EPHX3 | 1.017553134 | 1.19391E-21 |
| Z84486.1 | 1.464959992 | 1.19703E-21 |
| LINC02801 | 1.317968811 | 1.20869E-21 |
| SLC15A1 | 1.686094993 | 1.27478E-21 |
| KIF26B-AS1 | 1.960034872 | 1.28212E-21 |
| AC004816.2 | 1.12317711 | 1.34629E-21 |
| LINC01777 | 2.641629549 | 1.37907E-21 |
| AP002383.3 | 1.81151723 | 1.385E-21 |
| U3 | -2.094714462 | 1.42203E-21 |
| SYT4 | -3.181293305 | 1.53104E-21 |
| ALLC | 1.67507543 | 1.54189E-21 |
| IGHE | -1.428554309 | 1.67802E-21 |
| ELFN2 | -1.354698731 | 1.73814E-21 |
| CCKAR | 1.407542287 | 1.89604E-21 |
| AL445259.1 | 1.695639508 | 1.94454E-21 |
| LRRTM4 | 1.662417316 | 1.97557E-21 |
| AC110792.4 | 1.029316965 | 2.34686E-21 |
| KRT8P43 | -1.068105503 | 2.39519E-21 |
| Z98745.2 | 1.582157257 | 2.48368E-21 |
| FGD5P1 | 1.03997903 | 2.5541E-21 |
| SPTSSB | -1.238410836 | 2.59486E-21 |
| LINC01910 | 1.835181036 | 2.9478E-21 |
| LINC01929 | -1.060865804 | 3.19069E-21 |
| SAA1 | 1.2281925 | 3.19569E-21 |
| FAM135B | -1.418351213 | 3.44627E-21 |
| NTS | -2.283748315 | 3.51744E-21 |
| AL356218.2 | 1.38455972 | 3.52091E-21 |
| AC126768.1 | 1.579796104 | 3.63072E-21 |
| CXCL3 | 1.049644659 | 3.64894E-21 |
| AP001021.3 | 1.38161403 | 3.71174E-21 |
| TMEM145 | -1.14663761 | 3.86126E-21 |
| PGM5-AS1 | 1.413377607 | 4.75693E-21 |
| RNU5B-1 | 2.792932554 | 4.89016E-21 |
| SLC27A2 | -1.134343267 | 5.63756E-21 |
| ELF5 | 1.509941016 | 5.7332E-21 |
| CEACAM16 | -1.631308213 | 5.78914E-21 |
| FAM83A | -1.430905444 | 5.79922E-21 |
| AL031681.1 | -1.454428997 | 5.8229E-21 |
| NOL4 | 1.565818635 | 6.1056E-21 |
| ITIH2 | 1.056559892 | 6.21976E-21 |
| CHL1-AS2 | 1.345881426 | 6.51687E-21 |
| CD177 | -1.49605082 | 6.62907E-21 |
| PHEX-AS1 | 2.086708507 | 7.03648E-21 |
| TMEM211 | 1.488034125 | 7.06526E-21 |
| AC103957.1 | 1.66753106 | 7.38124E-21 |
| RNU6-242P | 1.494871791 | 7.5293E-21 |
| GRIN2B | 1.265170218 | 7.60985E-21 |
| KCNK3 | -1.308240267 | 8.0091E-21 |
| CTXN3 | 1.652993184 | 8.51937E-21 |
| LINP1 | 2.063076908 | 8.54514E-21 |
| MAGEE2 | 1.029579531 | 9.27654E-21 |
| MIR7-3HG | -1.726224447 | 9.99643E-21 |
| AL390236.1 | 1.172682167 | 1.01315E-20 |
| LINC01208 | -1.428278334 | 1.03843E-20 |
| KLK10 | 1.613351248 | 1.11873E-20 |
| DCD | -2.77519226 | 1.12296E-20 |
| RN7SKP9 | 2.742744213 | 1.22948E-20 |
| AL589693.1 | 1.301620296 | 1.36232E-20 |
| CLDN6 | 1.53633064 | 1.36992E-20 |
| SIRLNT | -2.501739591 | 1.40738E-20 |
| JCHAIN | 1.119009002 | 1.60659E-20 |
| AC090796.1 | -1.635576715 | 1.74778E-20 |
| LINC01460 | 1.532891982 | 1.79716E-20 |
| C1QL4 | 1.432369941 | 1.83682E-20 |
| AC025470.1 | 2.165881332 | 1.8786E-20 |
| EQTN | 1.404703551 | 1.89654E-20 |
| TMPRSS12 | 1.344966436 | 1.95919E-20 |
| AC108925.1 | 1.280714173 | 1.9859E-20 |
| CCL19 | 1.222285062 | 2.05868E-20 |
| SEC24AP1 | 1.352324516 | 2.05868E-20 |
| AL034346.1 | 1.328248659 | 2.09266E-20 |
| AL139379.1 | 2.225175517 | 2.10931E-20 |
| CRISP3 | -1.806212889 | 2.40398E-20 |
| AP001783.1 | 1.954991577 | 2.42373E-20 |
| AC063919.1 | 1.500681955 | 2.57132E-20 |
| TFDP1P2 | 1.154852044 | 2.59829E-20 |
| AC095032.1 | 1.060171727 | 2.96715E-20 |
| RGS7BP | 1.006999405 | 3.66383E-20 |
| DPP6 | 1.173314191 | 4.00494E-20 |
| IGHA1 | 1.126786946 | 4.4473E-20 |
| LINC02097 | 1.296544253 | 4.51348E-20 |
| AC092666.1 | 1.371768759 | 4.54772E-20 |
| ADGRG4 | 1.632756195 | 4.77095E-20 |
| YBX2P2 | 1.869519917 | 4.77549E-20 |
| AL450344.3 | 1.623551907 | 5.13968E-20 |
| STK32A-AS1 | 1.459967217 | 5.20431E-20 |
| SLC39A12 | 1.885968945 | 5.41227E-20 |
| AL357833.1 | 2.4296804 | 5.86608E-20 |
| AP005264.1 | 1.078697275 | 6.29844E-20 |
| TRPA1 | -1.43664771 | 6.33763E-20 |
| CNR2 | 1.173429103 | 6.64068E-20 |
| RN7SL170P | 2.172541665 | 7.65578E-20 |
| TRH | -1.808062386 | 7.88189E-20 |
| IGHD | 1.3700146 | 8.43618E-20 |
| CXCL17 | -1.45277944 | 8.83084E-20 |
| RPS26P21 | 1.622173379 | 8.97147E-20 |
| AC245187.2 | 1.606925369 | 9.54132E-20 |
| AC073325.1 | -2.268400191 | 9.71641E-20 |
| KRT5 | 1.404085001 | 9.8286E-20 |
| BTN1A1 | 1.023344889 | 1.01547E-19 |
| PPARGC1A | 1.054713821 | 1.09014E-19 |
| LINC01267 | 1.12155904 | 1.12752E-19 |
| ZG16 | -1.786951353 | 1.13591E-19 |
| AC009951.4 | 1.35460067 | 1.2489E-19 |
| LINC00603 | 1.636442232 | 1.47617E-19 |
| AL139317.4 | 1.134683159 | 1.49531E-19 |
| SPOCK3 | 1.377013701 | 1.64434E-19 |
| AL590807.1 | 1.682733929 | 1.66008E-19 |
| AL359538.3 | 1.919461798 | 1.66191E-19 |
| AC006059.4 | 1.301491082 | 1.74927E-19 |
| CAMKV | 1.613921909 | 1.91134E-19 |
| AC044893.1 | 1.907503585 | 1.92489E-19 |
| ADIPOQ | 1.523132176 | 1.97395E-19 |
| CARTPT | -3.590589963 | 2.05665E-19 |
| AKR7A3 | -1.118409645 | 2.09873E-19 |
| AC015909.2 | 1.181839048 | 2.13659E-19 |
| APOB | 1.237898065 | 2.23538E-19 |
| LGALS7B | 1.377446126 | 2.23541E-19 |
| NRTN | 1.037902415 | 2.25064E-19 |
| RPL21P133 | 1.413273881 | 2.3575E-19 |
| MROH2B | 1.41966536 | 2.43161E-19 |
| CDH19 | 2.035857817 | 2.65531E-19 |
| SLC8A2 | -1.244284622 | 2.67308E-19 |
| FCRL1 | 1.400500618 | 2.96805E-19 |
| SFTPA2 | -1.308956811 | 3.02576E-19 |
| SPINK4 | -1.488177569 | 3.10107E-19 |
| AC079193.2 | 1.157147113 | 3.13835E-19 |
| CNGA1 | 1.070108604 | 3.18494E-19 |
| SIM1 | 1.181637252 | 3.24839E-19 |
| TMEM132E | 1.013349402 | 3.25249E-19 |
| PLP1 | 1.196236975 | 3.48873E-19 |
| KCNG1 | 1.358752344 | 3.5234E-19 |
| FEZF2 | 2.309271564 | 3.53386E-19 |
| AC092118.1 | 1.276246593 | 3.58519E-19 |
| C8orf86 | -1.297593387 | 3.59371E-19 |
| LINC01563 | 1.057288714 | 3.60846E-19 |
| AC104370.1 | 2.600674986 | 3.72354E-19 |
| ORM2 | -1.562909234 | 3.91015E-19 |
| BLACAT1 | 1.369461366 | 4.79687E-19 |
| AC087242.1 | 1.808033112 | 4.88557E-19 |
| LINC01412 | 1.281952997 | 5.21507E-19 |
| AC002066.1 | 1.058647066 | 5.50488E-19 |
| AC006946.3 | 1.062850006 | 5.53389E-19 |
| NXPE4 | 1.142827154 | 5.79134E-19 |
| BOK-AS1 | 1.103215233 | 7.1547E-19 |
| GPR15 | 1.191459731 | 7.73826E-19 |
| AC062028.1 | -1.111399909 | 7.80262E-19 |
| MAB21L4 | -1.361262652 | 7.88329E-19 |
| TRHDE-AS1 | 1.35513764 | 8.06838E-19 |
| LINC02506 | -2.142300083 | 8.31622E-19 |
| XAGE2 | 3.292244773 | 8.69466E-19 |
| LINC01956 | 1.710835032 | 9.1234E-19 |
| SYT13 | -1.429514716 | 1.01257E-18 |
| PRDM13 | 2.802766553 | 1.04151E-18 |
| OR5E1P | 1.867725485 | 1.06813E-18 |
| AC092910.1 | 2.128455368 | 1.12857E-18 |
| NLRP7 | 1.187620329 | 1.19437E-18 |
| AC097462.3 | 1.465231062 | 1.1982E-18 |
| TBC1D27P | 1.187993674 | 1.19955E-18 |
| ASCL1 | -2.17730376 | 1.2016E-18 |
| COL22A1 | 1.079262424 | 1.22512E-18 |
| GABBR2 | 1.75081262 | 1.2535E-18 |
| AL445223.1 | 1.83823151 | 1.28724E-18 |
| AC138965.2 | -1.190214841 | 1.31932E-18 |
| TSPAN1 | -1.009817539 | 1.36526E-18 |
| CD1B | 1.211334498 | 1.3913E-18 |
| BBOX1 | 1.255516065 | 1.42133E-18 |
| AC006566.2 | 2.063055601 | 1.42474E-18 |
| KRTDAP | 1.602070965 | 1.44201E-18 |
| TMEM95 | 1.594125681 | 1.45165E-18 |
| AC008945.2 | 1.173922176 | 1.48446E-18 |
| WDR72 | -1.581883422 | 1.49427E-18 |
| IL19 | -1.097956551 | 1.52246E-18 |
| LINC02183 | 1.253563774 | 1.5832E-18 |
| AL731553.1 | 1.144723749 | 1.69472E-18 |
| ITPRID1 | 1.521817017 | 1.71827E-18 |
| MOG | 1.851361297 | 1.76248E-18 |
| TCL1A | 1.422751222 | 1.78692E-18 |
| C2CD4A | -1.392273579 | 1.78987E-18 |
| AC019171.1 | 1.317541853 | 1.79276E-18 |
| SLC13A5 | 1.088112629 | 1.79296E-18 |
| A3GALT2 | 1.088559075 | 1.79509E-18 |
| LINC01983 | -1.655138812 | 1.83481E-18 |
| FGF13-AS1 | 1.024803627 | 1.86343E-18 |
| LINC00518 | 2.131721732 | 1.90605E-18 |
| AC120498.4 | -1.119201387 | 1.96464E-18 |
| AL138689.1 | 1.020280941 | 2.03492E-18 |
| TSHB | 1.266538955 | 2.06883E-18 |
| AL035447.1 | 1.547620119 | 2.25174E-18 |
| LRIT2 | 1.872925866 | 2.28109E-18 |
| AL356433.1 | 1.554326116 | 2.30939E-18 |
| PGAM1P2 | 1.847019109 | 2.40725E-18 |
| AC000065.1 | 1.353901166 | 2.43165E-18 |
| AC103591.2 | 1.075628732 | 2.45603E-18 |
| ZPLD1 | -1.288210101 | 2.47361E-18 |
| SLC6A2 | 1.568032995 | 2.52758E-18 |
| H4C15 | -1.093212645 | 2.6871E-18 |
| LINC02872 | 1.10657586 | 2.74544E-18 |
| IQCJ-SCHIP1-AS1 | 2.452618121 | 2.90231E-18 |
| LINC00402 | 1.279183453 | 2.96477E-18 |
| AL358394.1 | 1.372768448 | 2.99293E-18 |
| LINC02817 | 1.087098402 | 3.13802E-18 |
| AC104393.1 | 2.114467972 | 3.21878E-18 |
| AP001021.2 | 1.452546781 | 3.2283E-18 |
| FOXL2NB | 1.481625004 | 3.54615E-18 |
| SCDP1 | -1.118660938 | 3.58351E-18 |
| AL139106.1 | 1.31357065 | 3.68848E-18 |
| DNAJB6P7 | 1.90376237 | 3.69081E-18 |
| AL450267.2 | 1.863045932 | 3.71174E-18 |
| LINC01215 | 1.143445553 | 3.74255E-18 |
| SAA2 | 1.138602961 | 3.74786E-18 |
| AL133232.1 | 1.820739942 | 3.75494E-18 |
| LINC01276 | 1.374047327 | 3.79233E-18 |
| CGA | -2.061126548 | 4.01378E-18 |
| ALDH1L1-AS2 | 1.388350312 | 4.07848E-18 |
| NR4A1AS | 1.046207324 | 4.25651E-18 |
| AL354861.3 | 1.308830055 | 4.32387E-18 |
| AC022001.2 | 1.623341797 | 4.3741E-18 |
| AC003077.1 | 1.267638381 | 4.54496E-18 |
| PMP2 | 2.153353713 | 5.50186E-18 |
| CHIA | 2.07346555 | 5.72707E-18 |
| CRTAC1 | 1.088884904 | 5.86734E-18 |
| LINC01436 | 1.163553197 | 6.59369E-18 |
| CHRNB2 | -1.14804463 | 7.04793E-18 |
| KCNE5 | 1.162785869 | 7.21075E-18 |
| AL356292.1 | 1.393567331 | 7.99811E-18 |
| ANKRD30BP1 | -1.482663543 | 8.01912E-18 |
| NCMAP | 1.130367105 | 8.02508E-18 |
| FP325317.1 | 1.655405695 | 8.11205E-18 |
| P3H2-AS1 | 1.472105967 | 8.1716E-18 |
| AC026408.1 | 1.368764267 | 8.189E-18 |
| LINC02347 | 1.860360827 | 8.21122E-18 |
| AC136621.1 | 1.74150692 | 8.58253E-18 |
| LINC02721 | 1.299488246 | 8.61429E-18 |
| AC009731.1 | 1.942061814 | 8.69272E-18 |
| PPP1R1C | -1.326404531 | 8.77256E-18 |
| IGHV3-72 | 1.163072464 | 8.9107E-18 |
| EPYC | -1.189891888 | 9.06338E-18 |
| AC011840.2 | 1.362849209 | 9.28762E-18 |
| LINC02182 | 1.432472313 | 9.70581E-18 |
| CCN6 | 1.25572604 | 1.01616E-17 |
| SLC4A10 | -1.347569899 | 1.02203E-17 |
| C4orf45 | 1.412299424 | 1.03449E-17 |
| PHOX2A | 1.763631776 | 1.05951E-17 |
| PRG2 | 1.155519985 | 1.17614E-17 |
| ALDH3A1 | 1.063180878 | 1.41812E-17 |
| AL122014.1 | 1.755381729 | 1.44245E-17 |
| AC018445.3 | 1.416988533 | 1.45952E-17 |
| MYT1 | -1.26523298 | 1.48642E-17 |
| AC007920.2 | 1.1369139 | 1.73114E-17 |
| AC023296.1 | 1.232727243 | 1.77007E-17 |
| RERGL | 1.162270706 | 1.77953E-17 |
| CDK6-AS1 | 1.028565506 | 1.80078E-17 |
| SVOP | -1.860426834 | 1.94477E-17 |
| AL358976.1 | 1.357840268 | 1.9705E-17 |
| AC126365.1 | -1.13681115 | 2.05505E-17 |
| KCTD4 | 1.653923405 | 2.06159E-17 |
| AL592429.1 | 1.299170692 | 2.14428E-17 |
| TBX10 | -1.948559805 | 2.15161E-17 |
| SLC6A15 | 1.760163775 | 2.28163E-17 |
| AL356489.3 | 1.07735841 | 2.29959E-17 |
| F11-AS1 | 2.675717225 | 2.48388E-17 |
| ETS1-AS1 | 2.214857906 | 2.51024E-17 |
| RIC3-DT | 3.088440765 | 2.56591E-17 |
| AC147651.1 | 2.094375223 | 2.63297E-17 |
| AC026355.2 | -1.426628495 | 2.69032E-17 |
| LINC02268 | 1.16477018 | 2.73837E-17 |
| PTPRZ1 | 1.387379569 | 2.80533E-17 |
| AC006159.1 | 1.907662614 | 2.82935E-17 |
| LINC00636 | 1.258492138 | 3.03698E-17 |
| MS4A1 | 1.247417311 | 3.07388E-17 |
| RHOXF1P3 | -1.589758437 | 3.17568E-17 |
| TMEFF2 | 1.37660544 | 3.43871E-17 |
| KRT24 | -1.824254923 | 3.45578E-17 |
| IGF2BP2-AS1 | 2.428999314 | 3.48887E-17 |
| AC105114.1 | 2.236604733 | 3.48929E-17 |
| AC135178.2 | 1.280051692 | 3.49462E-17 |
| GSTA3 | -1.623982634 | 3.59832E-17 |
| LHX1 | -2.162010045 | 3.96723E-17 |
| CR769775.2 | 1.530834445 | 3.97374E-17 |
| VPREB3 | 1.001164009 | 4.04135E-17 |
| ADH1A | 1.220172825 | 4.09649E-17 |
| CLDN10 | 1.227325521 | 4.56577E-17 |
| AL353746.1 | 1.257139673 | 4.58971E-17 |
| AMN | 1.004635247 | 4.61542E-17 |
| CASC8 | 1.252618948 | 4.63337E-17 |
| GABRG1 | 1.280887808 | 4.66017E-17 |
| AC104260.3 | 1.164972415 | 4.75182E-17 |
| AC002546.1 | 1.521567561 | 4.79203E-17 |
| LINC01070 | 1.677029526 | 5.1314E-17 |
| AP001628.2 | 1.637350127 | 5.26572E-17 |
| NT5C1A | 1.243220066 | 5.38802E-17 |
| TF | 1.144698115 | 5.54287E-17 |
| C11orf91 | 1.065768648 | 5.82982E-17 |
| GPM6A | -1.254458878 | 6.09715E-17 |
| ITPKB-IT1 | 1.296283801 | 6.27354E-17 |
| IL12RB2 | 1.165575092 | 6.56192E-17 |
| MAGEB4 | 2.316769411 | 7.04127E-17 |
| AC011374.1 | 1.053255057 | 7.46051E-17 |
| AP005060.1 | 2.034372945 | 7.7549E-17 |
| ALX3 | 1.624150219 | 7.87558E-17 |
| CEACAM6 | -1.337310186 | 7.87797E-17 |
| AC097499.2 | -1.7264518 | 7.97523E-17 |
| ROS1 | 1.343683316 | 8.04776E-17 |
| SYNPO2L | -1.360727818 | 8.18539E-17 |
| AC034159.2 | 1.891481548 | 8.27954E-17 |
| AC006487.1 | 1.099798499 | 8.30906E-17 |
| DDX11L10 | -1.115380658 | 9.04814E-17 |
| LINC00392 | 4.060093629 | 9.5753E-17 |
| NAMA | 1.211230006 | 9.80693E-17 |
| AC068774.1 | 1.024335035 | 1.083E-16 |
| AL138899.1 | 1.043009172 | 1.10059E-16 |
| AL359502.2 | 1.922002782 | 1.10786E-16 |
| TIMP4 | 1.025605208 | 1.16522E-16 |
| PLET1 | 1.548035214 | 1.18173E-16 |
| SLIT3-AS1 | 1.568875317 | 1.25781E-16 |
| ACTG1P22 | -1.715183704 | 1.30884E-16 |
| AC005863.1 | 1.794132218 | 1.34325E-16 |
| HAR1B | -1.292671534 | 1.36133E-16 |
| CHIAP1 | 2.107612447 | 1.36133E-16 |
| LINC02847 | 1.144287969 | 1.40619E-16 |
| FGB | -2.168829944 | 1.45027E-16 |
| AC021683.6 | 1.534485642 | 1.45548E-16 |
| AC118555.1 | 1.716979054 | 1.547E-16 |
| LINC01522 | -1.193981674 | 1.57179E-16 |
| SLC22A12 | 1.896306714 | 1.60114E-16 |
| AC012363.2 | 1.246651306 | 1.60114E-16 |
| AC113370.1 | 1.52929623 | 1.63989E-16 |
| OR2L3 | 1.683697017 | 1.66752E-16 |
| AL357874.1 | 1.002221172 | 1.78187E-16 |
| ELANE | 1.088207619 | 1.82621E-16 |
| MTOR-AS1 | 1.71748023 | 1.90645E-16 |
| CPA2 | 1.134120479 | 1.94453E-16 |
| KRT17P2 | 1.230766101 | 1.95702E-16 |
| AL356275.1 | 1.073127021 | 1.96163E-16 |
| LINC02794 | 1.327568634 | 1.96198E-16 |
| C4orf54 | 1.586343999 | 1.96308E-16 |
| HNRNPA3P7 | 1.202278599 | 2.06104E-16 |
| LINC01198 | 1.873719501 | 2.29638E-16 |
| LINC01230 | 1.22906014 | 2.34492E-16 |
| CDH7 | -1.483412951 | 2.44786E-16 |
| C6 | 1.188601686 | 2.45684E-16 |
| ICAM5 | 1.008927123 | 2.54636E-16 |
| AC016995.1 | 1.714954704 | 2.5716E-16 |
| IGHV1-45 | 1.274235231 | 2.57656E-16 |
| ANXA8L1 | 1.243413677 | 2.60754E-16 |
| DCX | 1.438123004 | 2.64489E-16 |
| SNORA47 | 1.179989911 | 2.68722E-16 |
| AL161717.1 | 1.520915621 | 2.82229E-16 |
| LINC02066 | 2.333344039 | 2.87454E-16 |
| AP001970.1 | 2.180487639 | 3.10537E-16 |
| SH3GL3 | 1.469355996 | 3.23764E-16 |
| ADIPOQ-AS1 | 1.781620774 | 3.31808E-16 |
| FTMT | 2.590457986 | 3.3207E-16 |
| TUFMP1 | -1.403706657 | 3.55943E-16 |
| GPR12 | 1.587549839 | 3.77264E-16 |
| KRT16 | 1.427789639 | 3.83374E-16 |
| LINC01781 | 1.25025659 | 4.1389E-16 |
| TAFA4 | 1.388873251 | 4.23803E-16 |
| FABP1 | 1.903961064 | 4.40623E-16 |
| LINC02237 | 1.547815989 | 4.56812E-16 |
| NRAD1 | 1.263258866 | 4.69947E-16 |
| KRT83 | 1.380107205 | 4.70458E-16 |
| KLHL30-AS1 | 1.406577121 | 4.90434E-16 |
| C11orf86 | 2.073160113 | 4.92524E-16 |
| AC025423.3 | -1.920379216 | 5.35136E-16 |
| AL445985.2 | -1.297368721 | 5.7957E-16 |
| AL035425.1 | 1.457670007 | 5.83691E-16 |
| CCND2P1 | -2.293688817 | 5.87442E-16 |
| DIO1 | -1.22752841 | 6.08789E-16 |
| LINC01808 | 1.740953462 | 6.52459E-16 |
| BARX1 | 1.207826887 | 6.94368E-16 |
| AC006518.2 | 1.200091043 | 7.23375E-16 |
| LINC01671 | 1.118763299 | 7.35215E-16 |
| AC006058.3 | 1.880925554 | 7.60118E-16 |
| AC006058.4 | 1.363266766 | 8.09877E-16 |
| AC026316.3 | 1.800638927 | 8.12572E-16 |
| LINC02188 | 1.48595888 | 8.18164E-16 |
| LINCADL | 1.499721625 | 8.41569E-16 |
| AQP7P2 | 1.721772594 | 8.41944E-16 |
| SNORD93 | 1.315236854 | 8.73392E-16 |
| RNU1-88P | 3.064394529 | 8.97706E-16 |
| PTENP1-AS | 1.129457704 | 9.0663E-16 |
| GAL3ST3 | 1.521157778 | 9.08493E-16 |
| LEMD1 | 1.552473284 | 9.18336E-16 |
| SERTM1 | 1.37453356 | 9.45901E-16 |
| DISC1FP1 | 1.34564181 | 9.58261E-16 |
| AC112250.2 | 1.238918544 | 9.73646E-16 |
| CNTNAP3C | 1.66766115 | 9.7502E-16 |
| TNFRSF13B | 1.070555209 | 1.01641E-15 |
| SLC5A5 | -1.041627911 | 1.07718E-15 |
| PSORS1C3 | 1.251228713 | 1.10348E-15 |
| ADGRF4 | -1.199287214 | 1.1109E-15 |
| AC010395.1 | 1.714394271 | 1.11936E-15 |
| FAM169B | 1.418429546 | 1.12558E-15 |
| AC022001.1 | 1.855714738 | 1.15892E-15 |
| AL353660.1 | 1.126898158 | 1.16642E-15 |
| AP001977.1 | 1.432460443 | 1.21601E-15 |
| AC120349.3 | 1.435485555 | 1.42919E-15 |
| FLG2 | 1.690802658 | 1.43935E-15 |
| MTFR2P1 | 1.396510362 | 1.44005E-15 |
| AL133268.4 | 1.391333926 | 1.51814E-15 |
| AC006504.2 | 2.026392177 | 1.65998E-15 |
| AL122034.1 | 2.267870164 | 1.71466E-15 |
| AL445183.2 | 1.080669012 | 1.88883E-15 |
| ARHGAP40 | 1.3196656 | 1.90208E-15 |
| RN7SKP240 | 1.845744872 | 1.91082E-15 |
| MUC7 | 1.991230311 | 1.91546E-15 |
| CNTNAP2 | -1.087411965 | 2.15404E-15 |
| TKTL1 | 1.027013486 | 2.24218E-15 |
| AL136114.1 | -1.246826664 | 2.30483E-15 |
| FRG1GP | -1.031150377 | 2.40261E-15 |
| AC107050.1 | 2.04814718 | 2.40352E-15 |
| LINC01902 | 1.115512019 | 2.40736E-15 |
| FERMT1 | 1.056132936 | 2.4217E-15 |
| FNTAP2 | 1.365388406 | 2.42313E-15 |
| LINC02065 | 1.384654056 | 2.42481E-15 |
| AVPR1B | 1.139169252 | 2.4901E-15 |
| KCNIP1-AS1 | 1.754078837 | 2.58632E-15 |
| TMPRSS6 | -1.046855894 | 2.63761E-15 |
| AL672032.1 | 1.083768471 | 2.90368E-15 |
| AC015983.2 | 1.087341435 | 3.15077E-15 |
| CEACAM7 | -1.131216994 | 3.23094E-15 |
| MIR205 | 1.191256889 | 3.37557E-15 |
| AC025431.1 | 1.401217679 | 3.3999E-15 |
| AC234772.1 | 1.675379167 | 3.4708E-15 |
| AC131182.1 | 1.781275502 | 3.53167E-15 |
| AL512631.1 | 1.232315265 | 3.62844E-15 |
| C1orf94 | 2.095940019 | 3.68468E-15 |
| AL731559.1 | 1.367367641 | 3.7297E-15 |
| H2AC12 | 1.137226183 | 3.78545E-15 |
| LINC02157 | 1.056259521 | 4.24012E-15 |
| AC010641.2 | 1.037565263 | 4.32068E-15 |
| LEMD1-AS1 | 1.06188308 | 4.45319E-15 |
| AL031121.2 | 1.043407145 | 4.45522E-15 |
| HNRNPRP1 | 1.186734519 | 4.45885E-15 |
| CLEC4GP1 | 1.393312368 | 4.51784E-15 |
| MSMB | -1.234841903 | 4.53244E-15 |
| CSMD3 | -1.85756427 | 4.71776E-15 |
| LINC02515 | 1.142672572 | 4.87316E-15 |
| AL121957.1 | 1.479771613 | 4.96816E-15 |
| AC104260.1 | 1.091092682 | 5.36054E-15 |
| AC007099.1 | 1.905899666 | 5.42798E-15 |
| NETO1 | -1.010757872 | 5.44797E-15 |
| LINC00844 | 1.169536714 | 5.59012E-15 |
| LINC00670 | 1.345272311 | 5.60544E-15 |
| PGPEP1L | 1.306143683 | 5.6103E-15 |
| AC117503.5 | 1.67114494 | 5.98871E-15 |
| AL035587.3 | 1.318449318 | 6.15752E-15 |
| ILF2P1 | 1.292969128 | 6.20075E-15 |
| AC188616.1 | 1.164732529 | 6.37494E-15 |
| AL512598.2 | 1.007291616 | 6.4967E-15 |
| MYL7 | 1.391974108 | 6.84043E-15 |
| SCARNA5 | -1.51351156 | 6.989E-15 |
| MAGEB2 | -2.315698805 | 7.22707E-15 |
| CACNG2 | -1.433761135 | 7.2767E-15 |
| UBE2U | 2.515276744 | 7.8806E-15 |
| NR5A1 | 1.524068332 | 8.05883E-15 |
| ABCC11 | -1.121230441 | 8.17758E-15 |
| TMEM72 | 1.250496886 | 8.35895E-15 |
| AL034351.3 | 1.537873965 | 8.83613E-15 |
| AC090543.1 | 1.328508029 | 9.44772E-15 |
| AC133106.1 | 1.851224421 | 9.49888E-15 |
| LINC01423 | 1.138172267 | 9.83394E-15 |
| CECR2 | -1.010398033 | 1.03302E-14 |
| AC092135.1 | 1.612767298 | 1.04827E-14 |
| AC017104.5 | 1.092330808 | 1.05118E-14 |
| KAZN-AS1 | -1.141448313 | 1.05481E-14 |
| LHX1-DT | -2.112775107 | 1.10198E-14 |
| NFYCP2 | 2.446511283 | 1.11276E-14 |
| LACRT | -2.695548861 | 1.12792E-14 |
| TAGLN3 | 1.334850669 | 1.1494E-14 |
| TARID | 1.310084431 | 1.15785E-14 |
| AL355870.2 | 1.433153048 | 1.23018E-14 |
| AC022148.1 | 1.130738266 | 1.29843E-14 |
| AL356234.2 | -1.266855267 | 1.33178E-14 |
| KCNH6 | -1.27499993 | 1.36029E-14 |
| SLC30A10 | 1.340070596 | 1.38019E-14 |
| GABRG3 | 1.45749178 | 1.40552E-14 |
| AC110772.1 | 1.3119793 | 1.40743E-14 |
| AC084866.1 | 1.352192492 | 1.4155E-14 |
| AL138716.1 | 1.104553429 | 1.42589E-14 |
| AC097375.1 | 1.427002066 | 1.43755E-14 |
| AC011246.1 | 1.200739774 | 1.47343E-14 |
| KRT9 | 1.412851747 | 1.57101E-14 |
| H2BE1 | 1.061424488 | 1.59527E-14 |
| CCDC83 | -1.042024854 | 1.6305E-14 |
| AC093675.2 | 1.333369016 | 1.71939E-14 |
| TSEN15P2 | 1.354673416 | 1.90919E-14 |
| NLRP10 | 1.405432233 | 1.9188E-14 |
| LINC02149 | -2.231012897 | 1.93914E-14 |
| LINC01761 | 1.706132375 | 1.9566E-14 |
| AC005753.2 | 1.115944582 | 1.96998E-14 |
| AC021739.3 | 1.353266423 | 1.97305E-14 |
| GAPDHS | 1.254935178 | 1.9789E-14 |
| NLRP13 | 1.761818441 | 2.0349E-14 |
| AL138686.2 | 2.098901008 | 2.35534E-14 |
| AC010307.4 | -1.054260686 | 2.38308E-14 |
| AL357143.1 | 1.062091101 | 2.39496E-14 |
| HTR1A | 1.533093083 | 2.40029E-14 |
| AL121956.1 | 1.600449905 | 2.40029E-14 |
| AC092903.1 | 1.887618033 | 2.43796E-14 |
| KIRREL3-AS1 | 1.997418773 | 2.46579E-14 |
| HOXD13 | 1.101869907 | 2.53537E-14 |
| AL161773.1 | 1.004841662 | 2.57115E-14 |
| AC009268.3 | 1.684353396 | 2.70411E-14 |
| AC025434.1 | 1.889202001 | 2.73402E-14 |
| RNF222 | 1.020815983 | 2.73785E-14 |
| CCDC92B | -2.370625168 | 2.74414E-14 |
| AL022238.1 | 1.161232119 | 2.89118E-14 |
| SAA4 | 1.218726119 | 3.01319E-14 |
| AC084064.1 | 1.583624107 | 3.08651E-14 |
| AC105129.1 | 1.284380455 | 3.10208E-14 |
| CTCFL | 1.344112582 | 3.13502E-14 |
| TPSP2 | -1.138442555 | 3.1426E-14 |
| AP001324.3 | 1.056555367 | 3.27993E-14 |
| EEF1A1P32 | 1.528157014 | 3.30314E-14 |
| CST5 | -1.879355719 | 3.55871E-14 |
| LRRC38 | 1.134044167 | 3.56879E-14 |
| DHRS2 | -1.390193349 | 3.57206E-14 |
| AC008013.2 | 1.23505568 | 3.60624E-14 |
| AC015574.1 | 1.820853086 | 3.66974E-14 |
| AL162574.1 | 1.12456875 | 3.67974E-14 |
| AC021979.3 | 1.837099253 | 3.73858E-14 |
| AC080188.1 | 1.132285491 | 3.83679E-14 |
| GAPDHP42 | 1.133387058 | 3.91403E-14 |
| AL390242.1 | 1.41919936 | 3.92972E-14 |
| AC079600.3 | 1.650169541 | 3.95495E-14 |
| LIVAR | -1.097329724 | 3.958E-14 |
| AL353768.2 | 2.011129141 | 3.958E-14 |
| AC016251.1 | 1.858051027 | 4.16485E-14 |
| GAPDHP15 | 1.23015513 | 4.30227E-14 |
| AL513412.1 | 2.08643826 | 4.42855E-14 |
| GPR52 | 1.146783908 | 4.596E-14 |
| AC110023.1 | 1.935248027 | 4.94945E-14 |
| AL354861.2 | 1.177257911 | 5.00437E-14 |
| AGGF1P3 | 1.251249024 | 5.15186E-14 |
| MTCO1P15 | 1.402865861 | 5.20178E-14 |
| RNU4ATAC12P | 1.528190178 | 5.2169E-14 |
| LINC01724 | 1.41327786 | 5.45153E-14 |
| CELF2-AS2 | 2.432463701 | 5.69701E-14 |
| ADARB2-AS1 | -2.44190522 | 5.71988E-14 |
| LINC02351 | 1.16458853 | 5.91397E-14 |
| KCNC1 | -1.003932412 | 6.06528E-14 |
| AC091076.1 | 1.123104746 | 6.06528E-14 |
| AC092957.1 | 1.47556681 | 6.0888E-14 |
| RPL6P24 | 1.696366936 | 6.68839E-14 |
| RPL9P30 | 1.864710678 | 6.75665E-14 |
| AC111149.2 | -2.674732893 | 6.98693E-14 |
| TCF7L1-IT1 | 1.368517196 | 7.00258E-14 |
| IGLV3-13 | 1.295757886 | 7.28903E-14 |
| RPS7P12 | 1.174274033 | 7.35345E-14 |
| AC008764.10 | 1.308303992 | 7.42222E-14 |
| SP9 | 1.565061695 | 7.52614E-14 |
| MIR126 | 1.106428939 | 7.53681E-14 |
| AFAP1-AS1 | 1.276603303 | 7.54923E-14 |
| STARD13-IT1 | 1.540608094 | 7.60325E-14 |
| COL5A1-AS1 | 1.441182337 | 7.76908E-14 |
| AC010969.1 | 1.227198458 | 7.91264E-14 |
| AL450345.1 | -1.645815629 | 8.01346E-14 |
| SLC35D3 | -1.085405913 | 8.23523E-14 |
| RN7SKP255 | 3.447516848 | 8.43857E-14 |
| NDST4 | -1.899548242 | 8.48596E-14 |
| COL18A1-AS1 | 1.351278874 | 8.4908E-14 |
| AC093390.1 | 1.379902411 | 8.61675E-14 |
| TMED11P | 1.461614385 | 8.63635E-14 |
| AC098650.1 | 1.271098193 | 8.75694E-14 |
| AP001011.1 | 2.206700629 | 9.08195E-14 |
| AL355870.1 | 1.241255464 | 9.6078E-14 |
| MKX | -1.161124021 | 9.61558E-14 |
| CASC16 | -1.289735369 | 9.67748E-14 |
| TRDN | 1.154776189 | 9.73216E-14 |
| AC099689.3 | 1.549947121 | 1.0034E-13 |
| FIBCD1 | -1.093522466 | 1.0349E-13 |
| EPB42 | 1.245910258 | 1.04892E-13 |
| CHODL | 1.02844647 | 1.06605E-13 |
| IFNL4 | 1.426573196 | 1.08319E-13 |
| MALRD1 | -1.294244412 | 1.08446E-13 |
| AL360181.1 | 1.041043102 | 1.09663E-13 |
| AL109946.1 | 1.036691023 | 1.10612E-13 |
| AC131009.2 | -1.040263018 | 1.12346E-13 |
| FSCN1P1 | 1.04832082 | 1.17434E-13 |
| RNA5SP515 | 1.352687507 | 1.22249E-13 |
| PRKCA-AS1 | 2.576967175 | 1.24606E-13 |
| SEC14L4 | 1.393842089 | 1.25993E-13 |
| LEMD1-DT | 1.504970927 | 1.26712E-13 |
| AP000897.2 | 1.894242162 | 1.29306E-13 |
| AC044781.1 | 1.33670235 | 1.31342E-13 |
| AC020661.4 | 1.770127413 | 1.43399E-13 |
| AC130467.1 | 1.954619016 | 1.46283E-13 |
| SERPINB5 | 1.093835607 | 1.48146E-13 |
| AC025280.1 | 1.348250413 | 1.57408E-13 |
| GMCL1P2 | -2.082788091 | 1.70231E-13 |
| AP005233.2 | 1.33853233 | 1.70309E-13 |
| NMRK2 | 1.362263898 | 1.73815E-13 |
| AC012409.4 | 1.279444292 | 1.74767E-13 |
| AC000124.1 | -1.234889656 | 1.76713E-13 |
| KLK7 | 1.336519172 | 1.8715E-13 |
| RABGAP1L-AS1 | 1.333956913 | 1.8715E-13 |
| AC090220.1 | 2.0820353 | 1.88906E-13 |
| SPATA21 | 1.063170689 | 1.94498E-13 |
| KIRREL1-IT1 | 1.849131372 | 2.00436E-13 |
| B3GALT1-AS1 | 1.340944015 | 2.00915E-13 |
| MGAT3-AS1 | 1.076524635 | 2.01475E-13 |
| BTNL2 | 1.013333875 | 2.0645E-13 |
| AC092839.1 | 1.37003207 | 2.18048E-13 |
| AP001972.3 | 1.076134776 | 2.31754E-13 |
| SNORA70G | 2.135598896 | 2.32398E-13 |
| FAM25A | -2.151343297 | 2.39915E-13 |
| RAD1P2 | 1.058273099 | 2.48651E-13 |
| APOA5 | -1.304247807 | 2.52227E-13 |
| UBE2CP3 | 1.946650298 | 2.55859E-13 |
| AC022001.3 | 1.138208215 | 2.56702E-13 |
| AL033381.3 | 2.10728752 | 2.66266E-13 |
| PROK2 | 1.060120311 | 2.70217E-13 |
| R3HDML | 1.402103741 | 2.79172E-13 |
| AC115485.1 | 1.494615973 | 2.81365E-13 |
| XAGE3 | 1.777599152 | 2.85401E-13 |
| RNU4-22P | 1.273691643 | 2.87148E-13 |
| MPPED2-AS1 | 1.346423686 | 2.94868E-13 |
| AC093821.1 | 1.05763354 | 2.99758E-13 |
| BARHL2 | 1.949275133 | 3.07803E-13 |
| AC016549.1 | 1.706319758 | 3.13969E-13 |
| AL157392.1 | 1.89427923 | 3.46379E-13 |
| AC008992.1 | 1.068732715 | 3.47886E-13 |
| AL022337.1 | -1.913966095 | 3.48112E-13 |
| AL356747.1 | 2.932050943 | 3.5282E-13 |
| LINC02595 | 1.152987335 | 3.53983E-13 |
| AL136140.1 | 1.009008337 | 3.56265E-13 |
| AC087392.2 | 1.329379457 | 4.10584E-13 |
| AC004158.1 | 1.583178338 | 4.12518E-13 |
| KRT74 | 1.539013086 | 4.16765E-13 |
| AC002543.1 | 1.519277125 | 4.16765E-13 |
| LINC01811 | -1.29089443 | 4.20386E-13 |
| AC007953.2 | 2.037940688 | 4.24613E-13 |
| RASA2-IT1 | 2.100525324 | 4.29944E-13 |
| AC129492.2 | -1.112685959 | 4.31797E-13 |
| BTBD17 | 1.351105779 | 4.44804E-13 |
| AL391097.1 | 2.577794375 | 4.51234E-13 |
| AL159996.1 | 1.22770501 | 4.59638E-13 |
| AL353611.1 | 1.345220095 | 4.73546E-13 |
| AC012409.5 | 1.264907172 | 5.02913E-13 |
| RNU6-342P | 1.394940659 | 5.33206E-13 |
| ANK2-AS1 | 2.647506499 | 5.48521E-13 |
| Y_RNA | 1.778439379 | 5.54931E-13 |
| LINC01193 | -2.043193984 | 5.71855E-13 |
| IYD | -1.138180983 | 5.73652E-13 |
| VGLL2 | 1.451978832 | 5.81228E-13 |
| TEX15 | 1.768728675 | 5.83373E-13 |
| EBLN1 | -1.526066517 | 5.8653E-13 |
| DMRTA2 | 1.428464858 | 6.10702E-13 |
| SNORD13 | 1.952903965 | 6.16826E-13 |
| KCNH5 | 1.672287521 | 6.24869E-13 |
| AC009716.2 | 1.330139783 | 6.30626E-13 |
| IFNE | 1.024424346 | 6.38302E-13 |
| AC084114.1 | -2.470854295 | 6.43643E-13 |
| AL645768.1 | 1.042661154 | 6.47668E-13 |
| FGF4 | -3.033712481 | 6.48543E-13 |
| PRR13P1 | 2.191506749 | 6.68838E-13 |
| AF117829.2 | 1.90635872 | 6.90743E-13 |
| MIR2052HG | -1.298789946 | 7.1634E-13 |
| RSPO4 | 1.014970921 | 7.26859E-13 |
| LINC02181 | 1.461574215 | 7.42882E-13 |
| DIAPH1-AS1 | 1.371251048 | 7.58374E-13 |
| RPRML | 1.727294938 | 7.68183E-13 |
| ELAVL3 | -1.193076294 | 7.779E-13 |
| CES5AP1 | 1.012021549 | 7.96257E-13 |
| AC092168.2 | 1.090266513 | 7.96257E-13 |
| AC015722.2 | 1.14612464 | 7.97233E-13 |
| AC068075.2 | -1.278778561 | 8.24896E-13 |
| BPIFA1 | -2.54270321 | 8.28843E-13 |
| AC009505.1 | 1.963604545 | 8.72801E-13 |
| AL359555.2 | 1.789394084 | 9.05822E-13 |
| IGSF23 | 1.186131424 | 9.1239E-13 |
| AC093772.2 | 1.602548035 | 9.53372E-13 |
| AL139280.1 | 1.263761589 | 9.60943E-13 |
| CERS3-AS1 | 1.138900503 | 9.87048E-13 |
| AL035410.1 | 1.60742611 | 9.92324E-13 |
| RPL36AP41 | 1.702377413 | 1.03041E-12 |
| AL449214.1 | 1.89906207 | 1.05672E-12 |
| LIN28B | 2.581052322 | 1.1253E-12 |
| AL356273.1 | 1.532391249 | 1.15061E-12 |
| LIN28B-AS1 | 1.851333071 | 1.16737E-12 |
| AC114550.2 | 1.350618754 | 1.1869E-12 |
| SNORA74B | -1.440563564 | 1.21502E-12 |
| PTPRJ-AS1 | 1.519238009 | 1.2174E-12 |
| NEUROD1 | -3.109140088 | 1.23664E-12 |
| PROKR1 | -1.155679171 | 1.25029E-12 |
| RPL6P4 | -1.326222763 | 1.27687E-12 |
| AC105393.1 | 2.239711413 | 1.28622E-12 |
| AC025040.1 | 1.041265122 | 1.28758E-12 |
| AC093909.3 | 1.439941595 | 1.30302E-12 |
| NIP7P3 | 1.3684626 | 1.31719E-12 |
| DUSP13 | -1.106809408 | 1.39255E-12 |
| INSYN1-AS1 | -1.41445835 | 1.39255E-12 |
| HORMAD1 | 1.468763783 | 1.40864E-12 |
| AL392089.1 | 1.1257454 | 1.43317E-12 |
| AC114982.1 | 2.014219178 | 1.43364E-12 |
| FAM3D | 1.045505986 | 1.4357E-12 |
| ZNF840P | 1.197046767 | 1.43821E-12 |
| AC023509.5 | 1.317486763 | 1.4396E-12 |
| AL137781.1 | 1.362110745 | 1.55213E-12 |
| ANKUB1 | 1.105226091 | 1.62938E-12 |
| LINC02284 | 1.029209113 | 1.65307E-12 |
| GAPDHP51 | 1.762967271 | 1.66108E-12 |
| PPP1R9A-AS1 | 1.415752125 | 1.68369E-12 |
| AC004696.2 | 1.073671368 | 1.69696E-12 |
| AC103702.2 | 1.1899869 | 1.70241E-12 |
| PLS3-AS1 | 1.082247123 | 1.73762E-12 |
| IZUMO2 | 1.449886868 | 1.77641E-12 |
| BLZF2P | 1.214156159 | 1.81876E-12 |
| LINC00028 | 1.100682282 | 1.88434E-12 |
| IGLV10-54 | 1.077373946 | 1.91961E-12 |
| AC009152.6 | 1.23396102 | 1.93861E-12 |
| IGFBP1 | 1.171774262 | 1.96545E-12 |
| LINC02788 | 1.367155553 | 1.97331E-12 |
| MIR6772 | 1.09867506 | 2.10082E-12 |
| AC104656.1 | 2.217210156 | 2.11146E-12 |
| GPR26 | -1.410918008 | 2.13599E-12 |
| AL807761.4 | 1.721729558 | 2.17092E-12 |
| LINC01879 | -1.001420861 | 2.341E-12 |
| AC025181.1 | 1.137847037 | 2.34891E-12 |
| H3C15 | -1.140181478 | 2.35151E-12 |
| ZDHHC22 | -1.323007448 | 2.49713E-12 |
| KCNJ3 | -1.446812599 | 2.53483E-12 |
| SCGB3A1 | 1.156975676 | 2.58406E-12 |
| AC009139.1 | 1.078328682 | 2.59576E-12 |
| MIR4728 | -1.058627328 | 2.63021E-12 |
| FBXO40 | 1.118673346 | 2.68268E-12 |
| AL449983.1 | 1.946028495 | 2.69481E-12 |
| AL035603.1 | 1.051353002 | 2.70707E-12 |
| OVAAL | 1.141092248 | 2.74811E-12 |
| AL607028.1 | 1.064702604 | 2.77124E-12 |
| OTOP3 | -1.797240539 | 2.77625E-12 |
| H2AC9P | -1.080678003 | 2.80157E-12 |
| ANKRD34B | -1.160336277 | 2.88599E-12 |
| PRL | 1.099342489 | 2.91883E-12 |
| AC023403.1 | 1.607447447 | 2.93465E-12 |
| CUX2 | -1.071767938 | 3.00539E-12 |
| DNAH17-AS1 | 1.207982368 | 3.05439E-12 |
| KLK8 | 1.349552504 | 3.11764E-12 |
| AL160408.4 | -1.312084701 | 3.12254E-12 |
| SPDYE4 | 1.093881618 | 3.15003E-12 |
| AC091564.6 | 1.272079355 | 3.15551E-12 |
| B3GALNT1P1 | 1.514427551 | 3.28096E-12 |
| AC108156.1 | 1.192657497 | 3.3596E-12 |
| AP001994.1 | 1.05168456 | 3.44596E-12 |
| RFX4 | 1.256871596 | 3.49051E-12 |
| AL158207.2 | 1.787622037 | 3.51318E-12 |
| AC010280.1 | -1.620494482 | 3.52644E-12 |
| AL031658.2 | -2.14427433 | 3.5329E-12 |
| PNMT | -1.143928096 | 3.58807E-12 |
| AL022334.1 | 1.055005577 | 3.65817E-12 |
| AC008687.3 | 1.219781646 | 3.78276E-12 |
| NPY4R | 1.481555871 | 3.84263E-12 |
| NPY | 1.784245835 | 3.93903E-12 |
| OXGR1 | 1.04782466 | 3.94106E-12 |
| AC009044.1 | 2.109984289 | 4.15634E-12 |
| Y_RNA | 1.151250351 | 4.19346E-12 |
| AC104304.3 | -1.082184954 | 4.25922E-12 |
| AC026355.3 | -1.000560488 | 4.30002E-12 |
| PPP1R14C | 1.058597147 | 4.34044E-12 |
| SOHLH1 | -1.430050859 | 4.43459E-12 |
| AC092868.2 | 1.502109225 | 4.49336E-12 |
| AC007423.1 | 1.46293404 | 4.5625E-12 |
| SHCBP1L | 1.25640577 | 4.59304E-12 |
| AL512643.1 | 1.711539963 | 4.89012E-12 |
| MIR4534 | 1.400436106 | 5.04198E-12 |
| AP003032.1 | 1.320420419 | 5.07828E-12 |
| AC100788.1 | 1.19353215 | 5.17269E-12 |
| AC055714.1 | 1.901651263 | 5.24393E-12 |
| CLEC4C | 1.06840827 | 5.54164E-12 |
| MPRIP-AS1 | 1.038579436 | 5.83429E-12 |
| AL163192.1 | 1.798868532 | 5.88844E-12 |
| AL138847.2 | 1.126492337 | 6.02008E-12 |
| LINC01865 | 1.466246871 | 6.26955E-12 |
| RBMS3-AS2 | 1.09299881 | 6.38274E-12 |
| AC245123.1 | 1.149294953 | 6.48745E-12 |
| ATP5PBP1 | 1.743141568 | 6.54963E-12 |
| CSAG1 | -1.631271612 | 6.75035E-12 |
| LINC01364 | 1.497031576 | 6.89318E-12 |
| AC093001.1 | -1.332707424 | 7.03516E-12 |
| AP002762.3 | 1.479018471 | 7.11426E-12 |
| AL162578.1 | 1.21510134 | 7.57865E-12 |
| RNU6-107P | 1.773351414 | 7.92875E-12 |
| AC005696.4 | -1.06227042 | 8.35696E-12 |
| RAX2 | 1.300820527 | 8.91291E-12 |
| OTOR | -1.103955625 | 9.19448E-12 |
| LINC01889 | -1.302842418 | 9.20009E-12 |
| AC092145.1 | 1.036236699 | 9.21347E-12 |
| ALOXE3P1 | -1.194613796 | 9.33323E-12 |
| NPM1P20 | 1.725855175 | 9.66481E-12 |
| AC010480.1 | 1.923933052 | 9.68082E-12 |
| ONECUT2 | -1.077405726 | 9.79924E-12 |
| MIR4432HG | 1.022023995 | 9.83337E-12 |
| NUTM1 | 1.00588286 | 9.83665E-12 |
| AC013457.1 | 2.341818046 | 9.83665E-12 |
| HSD3B2 | 1.232256369 | 9.96092E-12 |
| AP005210.2 | 2.079879243 | 1.01175E-11 |
| BPIFA2 | -1.707862961 | 1.03388E-11 |
| AC105114.2 | 1.430108245 | 1.05677E-11 |
| LINC02251 | 1.391029447 | 1.0959E-11 |
| MIR27B | 1.011746777 | 1.10945E-11 |
| ANKRD26P3 | 1.636650803 | 1.11546E-11 |
| HSPE1P27 | 1.609137689 | 1.14817E-11 |
| LRRC53 | -1.877598604 | 1.17938E-11 |
| AC105094.1 | 1.640960379 | 1.18597E-11 |
| AC012349.1 | 1.08645651 | 1.23187E-11 |
| LINC01797 | 1.544471279 | 1.23529E-11 |
| GLYATL3 | -2.578313518 | 1.24347E-11 |
| LINC02753 | 1.375046679 | 1.27308E-11 |
| AC008957.2 | 1.795512507 | 1.29668E-11 |
| AL606923.2 | 1.902452693 | 1.32104E-11 |
| RPL35AP23 | 2.127691649 | 1.34043E-11 |
| RPS12P16 | 1.705649839 | 1.34321E-11 |
| AC009054.1 | 1.580331919 | 1.37172E-11 |
| AC016542.2 | 2.004605277 | 1.39053E-11 |
| GABRA4 | 1.521517658 | 1.39372E-11 |
| AC103725.1 | 1.653769513 | 1.39918E-11 |
| ZP2 | -1.638554411 | 1.42419E-11 |
| AC026341.3 | 1.240662906 | 1.43184E-11 |
| AP000802.1 | 1.181622597 | 1.44108E-11 |
| AC008619.1 | 1.017141279 | 1.46872E-11 |
| ANKRD18B | -1.225843074 | 1.4923E-11 |
| AL133480.1 | 2.105550001 | 1.55935E-11 |
| AL158157.1 | 1.831770249 | 1.57869E-11 |
| ELOCP33 | 1.750672587 | 1.61785E-11 |
| TAS2R13 | 1.220127316 | 1.69084E-11 |
| AL450344.2 | 1.810135214 | 1.72376E-11 |
| AC010978.1 | 1.031578497 | 1.74748E-11 |
| CHODL-AS1 | 1.744202347 | 1.75321E-11 |
| AC011944.2 | 1.071122483 | 1.75591E-11 |
| PSMC1P7 | -1.143675143 | 1.85003E-11 |
| POU5F1P5 | 1.764597821 | 1.885E-11 |
| CLCA2 | -1.235156151 | 1.91253E-11 |
| AC012354.1 | 1.441226227 | 1.92977E-11 |
| AC027013.1 | 1.342136413 | 1.94089E-11 |
| AC099792.1 | 1.012168207 | 2.06521E-11 |
| AC233976.1 | 1.220958074 | 2.18232E-11 |
| AC084740.1 | -1.175870235 | 2.18589E-11 |
| AL049649.1 | 1.099557622 | 2.19075E-11 |
| GIMAP3P | 1.05125497 | 2.23116E-11 |
| METTL8P1 | -1.49630302 | 2.25367E-11 |
| AC012409.1 | 1.538856618 | 2.43791E-11 |
| LMO7DN-IT1 | 1.107384276 | 2.53757E-11 |
| AL391839.1 | 2.153151824 | 2.62217E-11 |
| ARNTL2-AS1 | 1.4587132 | 2.70414E-11 |
| PCK1 | 1.079236421 | 2.73507E-11 |
| BX255923.1 | 1.451121886 | 2.78732E-11 |
| AL022151.1 | 1.438980466 | 2.81291E-11 |
| AF015262.1 | -1.032999312 | 2.88254E-11 |
| AL160281.1 | 2.318484675 | 2.97997E-11 |
| AC002458.1 | 1.733728266 | 3.059E-11 |
| LINC02323 | 1.14438573 | 3.10063E-11 |
| AC018558.7 | -2.024560784 | 3.2049E-11 |
| RNU1-36P | 1.313888426 | 3.21768E-11 |
| Z97192.1 | 1.349436954 | 3.27378E-11 |
| LCE1C | 1.586174663 | 3.39543E-11 |
| AL160408.1 | -1.148909874 | 3.40856E-11 |
| AL138828.1 | 1.020855586 | 3.44273E-11 |
| Y_RNA | 1.000493364 | 3.49304E-11 |
| ATP5MC1P6 | 1.46135232 | 3.5645E-11 |
| RPS12P17 | 1.264729599 | 3.59566E-11 |
| ENO1P3 | 1.562953034 | 3.67043E-11 |
| SIRPB3P | 1.024154996 | 3.72131E-11 |
| IQCA1L | 1.330335812 | 3.75086E-11 |
| AL845321.1 | 1.253791119 | 3.80786E-11 |
| AL355300.1 | 1.776276755 | 3.81806E-11 |
| MTDHP3 | 1.690926864 | 3.86562E-11 |
| RNU6-1189P | 1.144039283 | 3.86586E-11 |
| LINC02224 | -1.470223797 | 4.02203E-11 |
| AC099754.1 | 1.712098604 | 4.07941E-11 |
| AL096712.1 | 1.20158458 | 4.13304E-11 |
| AC084026.3 | -1.785822443 | 4.2609E-11 |
| SULT6B2P | 1.164281455 | 4.30864E-11 |
| AC055733.3 | 2.001985283 | 4.31846E-11 |
| AP001981.2 | 1.006318519 | 4.46559E-11 |
| GHRHR | 1.105271823 | 4.57335E-11 |
| RN7SL698P | 1.725564129 | 4.58433E-11 |
| AL356753.1 | 1.306218871 | 4.75101E-11 |
| RPL31P50 | 1.247250017 | 4.78919E-11 |
| LINC02303 | -1.634766895 | 4.81838E-11 |
| AC093843.1 | 1.680841774 | 5.09508E-11 |
| CALY | -1.005280778 | 5.20957E-11 |
| KCNAB1-AS1 | 1.85960895 | 5.34352E-11 |
| AC004672.1 | 1.183688828 | 5.34649E-11 |
| AC025186.1 | 1.233630075 | 5.36992E-11 |
| FGF3 | 1.303751287 | 5.37327E-11 |
| AC245100.3 | 1.09733787 | 5.51315E-11 |
| AL031587.4 | 2.041991222 | 5.74542E-11 |
| AC111182.1 | 1.088435237 | 5.82198E-11 |
| AL360178.2 | 2.422784828 | 5.84076E-11 |
| RPL12P20 | 2.050382173 | 6.01007E-11 |
| DPYD-AS1 | 1.192037817 | 6.24011E-11 |
| AC073592.6 | 1.5529006 | 6.24971E-11 |
| AC090192.1 | 1.042311524 | 6.5517E-11 |
| AC084277.1 | 1.520142877 | 6.66432E-11 |
| PCBP3-AS1 | 1.814715046 | 6.70089E-11 |
| AC133681.1 | 1.260615479 | 6.73969E-11 |
| BPIFB1 | -1.045760773 | 6.76893E-11 |
| ESX1 | 2.550327545 | 6.79427E-11 |
| ACP7 | 1.102514336 | 6.89341E-11 |
| AP003071.2 | 1.385947924 | 6.97536E-11 |
| KRT41P | -1.353106888 | 7.00336E-11 |
| AC012066.1 | 1.614133888 | 7.03667E-11 |
| DDX18P5 | 1.23596714 | 7.11036E-11 |
| AL139184.1 | 1.726451006 | 7.18338E-11 |
| AC092723.5 | 1.490303661 | 7.40591E-11 |
| RPS20P31 | 1.95626576 | 7.711E-11 |
| AC005230.1 | 2.063448178 | 7.71727E-11 |
| PADI3 | -1.137419398 | 7.74679E-11 |
| AC073592.5 | 1.308086226 | 7.74781E-11 |
| NXPE1 | 1.014087478 | 7.78539E-11 |
| ITPKB-AS1 | 1.432613268 | 7.80193E-11 |
| ATP5PBP3 | 1.531214334 | 7.88407E-11 |
| AL662873.1 | 1.45785917 | 8.24996E-11 |
| AC136431.2 | 1.028276452 | 8.50669E-11 |
| AL359555.4 | 1.42277294 | 8.52291E-11 |
| FGL1 | -1.118432978 | 8.66839E-11 |
| RNU6-199P | 1.349916028 | 9.4771E-11 |
| MSLN | 1.225888257 | 9.78289E-11 |
| CTSE | -1.108740774 | 9.829E-11 |
| AC073575.1 | 1.257535112 | 9.83038E-11 |
| AL583827.1 | 1.457113592 | 1.00164E-10 |
| AL512593.1 | 2.49055222 | 1.00819E-10 |
| U8 | 1.064677834 | 1.02327E-10 |
| AL033539.2 | 1.098134964 | 1.06457E-10 |
| AL157385.2 | 1.038266009 | 1.0648E-10 |
| PPP1R17 | 1.576119261 | 1.06916E-10 |
| KCNU1 | -1.300451195 | 1.0847E-10 |
| EREG | 1.103036858 | 1.08777E-10 |
| FER1L6-AS2 | 1.610187867 | 1.10092E-10 |
| HIKESHIP1 | 1.315544634 | 1.19265E-10 |
| AL357075.2 | 1.641056867 | 1.21051E-10 |
| LINC01231 | 1.273745739 | 1.22053E-10 |
| ANKRD30BP3 | 1.130536712 | 1.22547E-10 |
| CTSLP2 | 1.100021472 | 1.22547E-10 |
| RN7SKP163 | 1.34799172 | 1.28648E-10 |
| AC026355.4 | -1.25270431 | 1.30556E-10 |
| ZFP42 | 1.618571208 | 1.38252E-10 |
| LINC02275 | 1.198577859 | 1.40447E-10 |
| SPTBN1-AS1 | 1.737481029 | 1.40604E-10 |
| AC009154.2 | 1.464957047 | 1.41612E-10 |
| PCDH11X | 1.141465775 | 1.42055E-10 |
| MYBPC1 | 1.209432971 | 1.43059E-10 |
| AL590365.1 | 1.02065031 | 1.45376E-10 |
| AL512324.3 | 1.470288273 | 1.48377E-10 |
| RN7SL629P | 2.021155747 | 1.52028E-10 |
| AL161781.2 | 1.48377949 | 1.52888E-10 |
| AC090617.7 | 1.497228799 | 1.54101E-10 |
| AC100800.1 | 1.767676559 | 1.55231E-10 |
| AC105328.1 | -1.148395978 | 1.57596E-10 |
| AL137017.1 | 1.436671397 | 1.59434E-10 |
| THUMPD3P1 | 2.38538453 | 1.60009E-10 |
| AC087481.2 | 1.238581817 | 1.64265E-10 |
| GXYLT1P6 | 1.540367195 | 1.67956E-10 |
| GRIA2 | -1.341703787 | 1.71645E-10 |
| AC027018.1 | 1.153450957 | 1.72019E-10 |
| AP000779.1 | 2.187817868 | 1.74551E-10 |
| LINC02529 | 1.651458529 | 1.80028E-10 |
| ZIC4 | 1.088701073 | 1.80678E-10 |
| AC011483.1 | 1.464431495 | 1.8077E-10 |
| PRAP1 | 1.097335799 | 1.82005E-10 |
| SNX19P3 | -1.013226196 | 1.82916E-10 |
| AC004817.4 | 1.614826559 | 1.85741E-10 |
| LINC01630 | 1.189552375 | 1.886E-10 |
| CR391992.1 | 1.30392881 | 1.90459E-10 |
| AP003119.1 | -1.035714978 | 1.91931E-10 |
| ROBO2 | -1.004093161 | 2.01161E-10 |
| SLC5A8 | -1.471595947 | 2.03871E-10 |
| CST11 | -1.59204972 | 2.07671E-10 |
| AL646090.2 | 1.593240584 | 2.09929E-10 |
| AP004289.2 | 1.801022706 | 2.12488E-10 |
| BRINP2 | -1.08476917 | 2.13551E-10 |
| SELENOV | 1.338999046 | 2.14891E-10 |
| DNAAF6 | -1.07261918 | 2.15335E-10 |
| A2ML1 | 1.128950433 | 2.24853E-10 |
| KIF3AP1 | -1.596716364 | 2.26969E-10 |
| RNU6ATAC39P | 1.387964871 | 2.29008E-10 |
| Y_RNA | 1.279104514 | 2.29274E-10 |
| AL450322.2 | 1.173853606 | 2.30413E-10 |
| AL359852.1 | 1.131855258 | 2.43775E-10 |
| AP002001.1 | -2.012032415 | 2.4771E-10 |
| PRSS2 | -1.121009034 | 2.5131E-10 |
| COX5BP3 | 1.896418293 | 2.51667E-10 |
| AL772337.3 | -1.605357872 | 2.52274E-10 |
| AIRN | 1.555548371 | 2.53635E-10 |
| AC022915.2 | 1.021779451 | 2.57585E-10 |
| IGHEP1 | -1.246431081 | 2.64849E-10 |
| TRBJ2-6 | 1.195301516 | 2.66842E-10 |
| ARHGAP36 | 1.460757773 | 2.67829E-10 |
| OSTCP8 | 1.112585138 | 2.74254E-10 |
| ARHGEF3-AS1 | 2.114128737 | 2.7597E-10 |
| AL390783.1 | 1.091403513 | 2.81866E-10 |
| RPS15AP9 | 1.118550431 | 2.84653E-10 |
| LATS2-AS1 | 2.483948935 | 2.88619E-10 |
| AL117340.1 | 1.116855424 | 2.93712E-10 |
| PTPRK-AS1 | 1.0450008 | 2.96065E-10 |
| PLSCR5 | 1.758895989 | 3.0267E-10 |
| AC010531.4 | 1.495996189 | 3.15462E-10 |
| RHAG | -1.098275111 | 3.16497E-10 |
| LINC01532 | 1.279300214 | 3.16497E-10 |
| AP001172.1 | 1.189265753 | 3.19122E-10 |
| RBBP8P1 | 1.595294854 | 3.19394E-10 |
| AL121929.1 | -1.248268866 | 3.21576E-10 |
| LINC01567 | 1.556981429 | 3.23328E-10 |
| AC073127.1 | 1.832164925 | 3.26363E-10 |
| AC087286.1 | 1.838391498 | 3.27674E-10 |
| AC127496.3 | 1.002235841 | 3.27674E-10 |
| AC004491.1 | 1.45562512 | 3.28828E-10 |
| AC008667.2 | 1.515461351 | 3.29811E-10 |
| AL603824.1 | 1.581427759 | 3.30416E-10 |
| OLFM4 | 1.191062269 | 3.38028E-10 |
| DNER | 1.049222171 | 3.46909E-10 |
| AL034349.1 | 1.663765899 | 3.51625E-10 |
| RPS15AP6 | 1.000010429 | 3.52584E-10 |
| LINC00052 | -1.556514262 | 3.54615E-10 |
| ARHGEF9-IT1 | 1.39332583 | 3.62295E-10 |
| AL021408.2 | 1.346442664 | 3.69415E-10 |
| DAZL | 1.043888657 | 3.75592E-10 |
| RNU7-124P | 1.942048762 | 3.7941E-10 |
| AL049695.1 | 1.048933056 | 3.8225E-10 |
| RPL21P23 | 1.430753903 | 3.92772E-10 |
| CA1 | 1.104948615 | 3.99015E-10 |
| AC126773.5 | 1.283222295 | 4.02862E-10 |
| LINC00710 | 1.528684927 | 4.05357E-10 |
| HOMER2P1 | -1.811506746 | 4.06209E-10 |
| SLC26A9 | 1.028125414 | 4.12086E-10 |
| SSTR5 | -1.398137614 | 4.21482E-10 |
| KRT20 | -1.485002019 | 4.24096E-10 |
| AL358394.2 | 1.06809953 | 4.24096E-10 |
| ZIC1 | 1.144212402 | 4.3757E-10 |
| AC116345.1 | 1.178143448 | 4.43461E-10 |
| AC126182.3 | 1.068544062 | 4.48637E-10 |
| SUMO2P8 | 1.75935734 | 4.56075E-10 |
| AL158801.3 | 1.619601362 | 4.5883E-10 |
| AP006259.1 | 1.783757092 | 4.69803E-10 |
| LINC01779 | 1.082231652 | 4.81448E-10 |
| AC013451.1 | 1.159740802 | 4.82029E-10 |
| AL451123.2 | 1.145403105 | 5.02465E-10 |
| AL355613.1 | 1.213995209 | 5.13972E-10 |
| LINC02063 | 1.715712635 | 5.20527E-10 |
| NATP | -1.736263049 | 5.23137E-10 |
| AC136424.1 | 3.029135028 | 5.33742E-10 |
| AL133444.1 | 1.173924119 | 5.3918E-10 |
| LINC00393 | 2.008361976 | 5.49771E-10 |
| RPL23AP17 | 1.57009927 | 5.63915E-10 |
| AC091939.1 | 1.360061554 | 5.9288E-10 |
| LINC02433 | -1.265482604 | 5.98313E-10 |
| SSX1 | -2.688725813 | 5.99342E-10 |
| AC106800.3 | 1.390434555 | 6.12521E-10 |
| NKX1-2 | 1.409890066 | 6.17651E-10 |
| AC106872.4 | 1.421627579 | 6.39757E-10 |
| LINC02840 | 1.326772393 | 6.47353E-10 |
| AL035693.1 | 2.141829888 | 6.51284E-10 |
| AC083864.5 | 1.025481287 | 6.56918E-10 |
| AC093627.1 | 1.396285745 | 6.58472E-10 |
| MAGEC1 | -1.393104224 | 6.60529E-10 |
| POM121L2 | 1.268720909 | 6.61307E-10 |
| INGX | 1.05955982 | 6.6996E-10 |
| DMRT1 | 1.402504837 | 6.80233E-10 |
| AL161457.1 | 1.313267119 | 6.80368E-10 |
| RPS27AP8 | 1.695658067 | 7.11265E-10 |
| SPINK8 | -1.037775854 | 7.19891E-10 |
| E2F3-IT1 | 1.77690431 | 7.26344E-10 |
| Metazoa_SRP | -2.187963526 | 7.32419E-10 |
| AC004470.2 | 1.324959194 | 7.52683E-10 |
| AC055840.1 | 2.525502141 | 7.66709E-10 |
| AC018445.4 | 1.134409916 | 7.69477E-10 |
| AC106712.1 | -1.328186552 | 7.90102E-10 |
| AL353771.1 | 1.437706931 | 7.94786E-10 |
| MTA3P1 | 1.889529308 | 8.05509E-10 |
| AP005205.3 | 1.370163767 | 8.10962E-10 |
| FGFBP1 | 1.312529964 | 8.2576E-10 |
| SYNE1-AS1 | 1.24800805 | 8.52621E-10 |
| AC007040.3 | 1.045126989 | 8.63292E-10 |
| Y_RNA | 1.067110404 | 9.36705E-10 |
| AF131215.2 | 1.440214401 | 9.42916E-10 |
| LINC02648 | 1.040174105 | 9.4396E-10 |
| PHACTR2-AS1 | 1.031705897 | 9.53601E-10 |
| AC104791.1 | 1.074488948 | 9.60156E-10 |
| BANF1P1 | 1.083146235 | 9.84139E-10 |
| AC009154.1 | 1.309913621 | 1.00652E-09 |
| AF131215.1 | 2.214061685 | 1.02619E-09 |
| ELOA2 | 1.059814821 | 1.03708E-09 |
| AL589863.2 | 1.349483983 | 1.04637E-09 |
| MIR125B1 | 1.241164847 | 1.06529E-09 |
| RPL7P49 | 1.20174979 | 1.07883E-09 |
| LGALS14 | 1.219594468 | 1.13334E-09 |
| RPL7AP3 | 1.135796756 | 1.13639E-09 |
| CNMD | 1.022185636 | 1.21765E-09 |
| SEMA5A-AS1 | 1.595286491 | 1.22011E-09 |
| LINC01908 | 1.025020304 | 1.22102E-09 |
| AC135586.1 | 1.045373997 | 1.23891E-09 |
| GMNC | 1.483914107 | 1.27305E-09 |
| AC012213.4 | -1.461116206 | 1.28153E-09 |
| LHFPL4 | -1.012171604 | 1.32851E-09 |
| AC096996.2 | -1.016358867 | 1.3484E-09 |
| AC016042.1 | 1.343844209 | 1.35436E-09 |
| GSTM3P2 | 1.690445855 | 1.37387E-09 |
| AP002008.3 | 1.628498095 | 1.39134E-09 |
| AC108865.1 | 1.121243303 | 1.41887E-09 |
| TEX13C | -2.027454226 | 1.42895E-09 |
| LINC01971 | 1.041832664 | 1.43055E-09 |
| AL450003.1 | 2.103916331 | 1.43257E-09 |
| AC053503.5 | 1.548550489 | 1.4462E-09 |
| AC136285.3 | 1.607022071 | 1.46897E-09 |
| ADAM18 | -1.608805948 | 1.48E-09 |
| AC138965.1 | -1.086883141 | 1.51027E-09 |
| AP003467.2 | 1.580329555 | 1.54942E-09 |
| AC011239.1 | 1.334795505 | 1.57294E-09 |
| CRHR1 | 1.175033175 | 1.58824E-09 |
| AC104129.1 | 1.153442469 | 1.5956E-09 |
| AL139095.3 | 1.469810358 | 1.63506E-09 |
| DYNAP | -1.696125682 | 1.63931E-09 |
| AC100826.1 | -1.381193157 | 1.69735E-09 |
| AL445205.1 | 1.686383231 | 1.71776E-09 |
| MC5R | -1.01745754 | 1.74915E-09 |
| AC068580.2 | 1.285924032 | 1.8323E-09 |
| CSTL1 | -1.094215805 | 1.88817E-09 |
| AL606970.4 | 1.634491011 | 1.89423E-09 |
| KRT72 | 1.11106754 | 1.90249E-09 |
| LINC01488 | -1.052482492 | 1.94885E-09 |
| AL035696.4 | 1.188352654 | 1.97817E-09 |
| FIGLA | 1.09671575 | 2.02645E-09 |
| KRT13 | -1.305038462 | 2.17444E-09 |
| AL049629.2 | 1.115035325 | 2.23281E-09 |
| AC112693.1 | 1.614650211 | 2.2575E-09 |
| Y_RNA | 2.111856007 | 2.26184E-09 |
| COX6CP14 | 1.814140488 | 2.27486E-09 |
| AC000065.2 | 1.3128698 | 2.30383E-09 |
| PCDH8 | 1.082338663 | 2.33187E-09 |
| OR2A14 | 1.15876078 | 2.34951E-09 |
| NBPF13P | -1.072071672 | 2.35563E-09 |
| RNU5D-1 | 1.369207617 | 2.37973E-09 |
| AC008277.1 | 1.036828942 | 2.39839E-09 |
| URAD | 1.184344903 | 2.45483E-09 |
| TLX1NB | 1.194819759 | 2.46657E-09 |
| AC005096.1 | 1.219554099 | 2.52116E-09 |
| MUC2 | -1.417332731 | 2.53649E-09 |
| PSMA1P1 | 1.171424592 | 2.5488E-09 |
| AC108102.1 | 1.425871575 | 2.556E-09 |
| AC092120.1 | 1.468893239 | 2.57594E-09 |
| VDAC2P2 | 1.034438461 | 2.65837E-09 |
| AC040174.2 | 1.241011625 | 2.68263E-09 |
| SERPINB12 | 1.839958539 | 2.70295E-09 |
| AL512324.6 | 1.295417015 | 2.76971E-09 |
| AC022467.2 | 2.534067284 | 2.77539E-09 |
| TRBJ2-4 | 1.165302894 | 2.87375E-09 |
| SLIT2-IT1 | 1.82193296 | 2.8969E-09 |
| AC069243.1 | 2.334207225 | 2.89821E-09 |
| RNU6-446P | 1.241870345 | 2.9731E-09 |
| CDH10 | -1.275847366 | 2.9914E-09 |
| KRT18P49 | -1.031957522 | 3.00122E-09 |
| AL132982.1 | 1.525486515 | 3.02441E-09 |
| SETP10 | 1.699642595 | 3.03083E-09 |
| AL359555.1 | 1.221712847 | 3.05604E-09 |
| AC005076.2 | 1.038270188 | 3.09264E-09 |
| Y_RNA | 2.05684075 | 3.11928E-09 |
| TMA16P2 | 1.319582581 | 3.13928E-09 |
| LINC01254 | 1.395874245 | 3.14637E-09 |
| AC036176.3 | 1.615294218 | 3.17598E-09 |
| AL356776.1 | 1.255938573 | 3.17742E-09 |
| AC073508.1 | -1.288333473 | 3.24514E-09 |
| RPL35AP14 | 1.225677734 | 3.27941E-09 |
| AC021766.1 | 1.389738443 | 3.31243E-09 |
| AC018978.1 | 1.360398151 | 3.33076E-09 |
| AC005070.2 | 1.321906018 | 3.33963E-09 |
| ATP11A-AS1 | 1.540550064 | 3.41188E-09 |
| AC110801.1 | 1.254617477 | 3.41881E-09 |
| OR2L2 | 1.115542603 | 3.50509E-09 |
| GRK5-IT1 | 1.15489437 | 3.5091E-09 |
| AC009533.4 | 1.294467276 | 3.5201E-09 |
| NCOA7-AS1 | 1.749823405 | 3.52044E-09 |
| AL023495.1 | 1.239244206 | 3.53529E-09 |
| AC022730.4 | 1.208240523 | 3.53838E-09 |
| EIF2S2P5 | 2.043863087 | 3.59752E-09 |
| PROKR2 | 1.246563352 | 3.63381E-09 |
| NECAP1P2 | 1.277304221 | 3.65517E-09 |
| AL360182.1 | 1.358142571 | 3.66607E-09 |
| KRT16P2 | 1.220538913 | 3.66607E-09 |
| OR7E36P | 1.310801521 | 3.69932E-09 |
| FRMD6-AS2 | 1.161381701 | 3.71307E-09 |
| AL035252.2 | 1.193852596 | 3.71901E-09 |
| AL359694.1 | 1.101598857 | 3.76521E-09 |
| LINC01606 | 1.477233173 | 3.76554E-09 |
| LBP | -1.012530302 | 4.23559E-09 |
| LIPK | 1.034567493 | 4.33049E-09 |
| AL157402.2 | 1.766134429 | 4.38443E-09 |
| LINC02469 | 1.085193041 | 4.39986E-09 |
| AC005304.2 | 1.435368321 | 4.49747E-09 |
| AL161672.1 | 1.338270151 | 4.51063E-09 |
| RTP3 | 1.85920387 | 4.54517E-09 |
| NEUROG2 | 1.167541401 | 4.69012E-09 |
| AL136961.1 | 1.141569791 | 4.83268E-09 |
| HMGB1P51 | 1.358878162 | 4.83517E-09 |
| KRT85 | 1.135503017 | 4.84267E-09 |
| AC009533.3 | 1.175394416 | 4.87087E-09 |
| EEF1B2P8 | 1.930155412 | 4.90138E-09 |
| TAS2R50 | 1.008959611 | 5.09058E-09 |
| SLITRK1 | -1.303919667 | 5.29444E-09 |
| AC093277.1 | -1.714996478 | 5.49937E-09 |
| AC016866.2 | 1.791765883 | 5.561E-09 |
| AL050343.3 | 1.058053877 | 5.5994E-09 |
| AC108673.1 | 1.416427769 | 5.76211E-09 |
| AP000962.1 | 1.463912132 | 6.01441E-09 |
| ANKRD30BL | 1.18608428 | 6.22718E-09 |
| AL136320.1 | 1.507390715 | 6.34593E-09 |
| AC104462.2 | 1.636034436 | 6.56674E-09 |
| AC011676.3 | 1.809152487 | 6.71085E-09 |
| WNT8A | 1.102635274 | 6.7514E-09 |
| OR7E115P | 1.200858008 | 6.7514E-09 |
| PTP4A1P3 | 2.430838169 | 6.76865E-09 |
| LINC02249 | 1.364168559 | 6.95903E-09 |
| LINC02860 | 1.183390472 | 7.00764E-09 |
| AP001120.5 | 1.448300245 | 7.08205E-09 |
| OR7E110P | 1.722931194 | 7.15548E-09 |
| AC126323.1 | -1.095380494 | 7.18294E-09 |
| AL049820.1 | 1.566302677 | 7.2177E-09 |
| AC046158.1 | 1.357135487 | 7.27187E-09 |
| RN7SKP78 | 1.065504946 | 7.40326E-09 |
| AL592078.2 | -1.047677886 | 7.47548E-09 |
| AC009268.1 | 1.325673839 | 7.47956E-09 |
| AC009227.1 | -1.230731964 | 7.55063E-09 |
| AC103996.2 | 1.028587927 | 7.56995E-09 |
| TMEM213 | 1.0036928 | 7.61547E-09 |
| AC090953.1 | 1.133954265 | 7.65679E-09 |
| AC009509.3 | 1.746364456 | 7.67689E-09 |
| AC022493.2 | 1.568391953 | 8.09506E-09 |
| AC022872.1 | 1.094284013 | 8.17102E-09 |
| AC087749.2 | 1.218992803 | 8.19294E-09 |
| AC016642.1 | 1.403523601 | 8.23256E-09 |
| AC104164.2 | 2.025088616 | 8.2637E-09 |
| AC002383.1 | -1.504731693 | 8.31879E-09 |
| AL024474.2 | 1.637350475 | 8.32815E-09 |
| AC087203.2 | 1.00618375 | 8.49927E-09 |
| AC123023.1 | -1.125958662 | 8.50971E-09 |
| AC090519.1 | 1.023413994 | 8.69142E-09 |
| AP000962.3 | 2.442766684 | 8.73265E-09 |
| AC007731.3 | 1.488577995 | 8.76969E-09 |
| TLX1 | 1.206407461 | 8.93258E-09 |
| AL627311.1 | 1.968189273 | 9.14571E-09 |
| RNF113B | 1.078255014 | 9.15418E-09 |
| AC103853.2 | 1.332126468 | 9.15418E-09 |
| SCGB1D4 | -2.201913648 | 9.29804E-09 |
| AC005153.1 | 1.733396329 | 9.36931E-09 |
| AL355531.1 | 1.213687289 | 9.62879E-09 |
| AL451062.1 | 1.821661065 | 9.62977E-09 |
| KRT84 | 1.156319328 | 9.70448E-09 |
| RN7SL141P | 2.454498126 | 9.83715E-09 |
| AC104958.1 | 1.127750303 | 9.88273E-09 |
| AL442071.1 | 1.640617599 | 9.88389E-09 |
| AL355836.2 | 1.11639058 | 1.00452E-08 |
| AL109923.1 | 1.066245252 | 1.01816E-08 |
| AL023754.1 | -1.159745645 | 1.03137E-08 |
| LY6D | 1.216763306 | 1.03841E-08 |
| HNRNPA1P76 | 1.200866919 | 1.04306E-08 |
| ITM2BP1 | 1.156479359 | 1.04919E-08 |
| CYP4F26P | -1.307909326 | 1.06474E-08 |
| KRTAP1-1 | 1.420831415 | 1.07813E-08 |
| AC108025.1 | 1.656994548 | 1.09454E-08 |
| ATP8A2P2 | 1.108005442 | 1.10307E-08 |
| OR5P2 | 1.565152833 | 1.10415E-08 |
| LAMTOR3P2 | 1.001105444 | 1.12209E-08 |
| AL122001.1 | 1.028600555 | 1.13393E-08 |
| AC015920.1 | 1.284863235 | 1.14445E-08 |
| CYP2A7P2 | -1.24519455 | 1.14458E-08 |
| AP003100.1 | 1.687221619 | 1.14701E-08 |
| AC007639.1 | 1.112744452 | 1.15318E-08 |
| AP002448.1 | 1.574956764 | 1.15901E-08 |
| AL590128.1 | 1.54924135 | 1.19032E-08 |
| LINC01796 | 1.387166724 | 1.20058E-08 |
| AC091043.1 | 2.063008641 | 1.2125E-08 |
| CACNA1C-AS4 | 1.676876134 | 1.21715E-08 |
| MAGEB6 | -1.740766022 | 1.25025E-08 |
| LINC02346 | 1.229561084 | 1.274E-08 |
| AL162253.1 | 1.141389817 | 1.32855E-08 |
| AC079336.3 | 1.553337153 | 1.33939E-08 |
| RNU6-250P | -1.349886143 | 1.35067E-08 |
| TUBA3C | -1.225331397 | 1.39872E-08 |
| AC136424.2 | 2.098566249 | 1.41973E-08 |
| COX6B1P6 | 2.014985489 | 1.4461E-08 |
| NR2E1 | 1.14973085 | 1.48237E-08 |
| AL159158.1 | -1.192820435 | 1.51844E-08 |
| CUBNP3 | 1.276630726 | 1.53766E-08 |
| AC004775.1 | 1.07586266 | 1.54076E-08 |
| CER1 | 1.233365632 | 1.54866E-08 |
| OR6A2 | 1.457423606 | 1.59051E-08 |
| LZTS1-AS1 | 1.801023508 | 1.59391E-08 |
| INSM1 | -1.052565911 | 1.5952E-08 |
| AC016954.1 | 1.470379172 | 1.62536E-08 |
| LINC00992 | -1.042443068 | 1.64145E-08 |
| GSTA1 | 1.048270375 | 1.65748E-08 |
| OR11Q1P | 1.811144398 | 1.69531E-08 |
| PVALB | -1.108982102 | 1.76795E-08 |
| AC092329.2 | 1.014545306 | 1.7919E-08 |
| PPFIA1P1 | 1.011473785 | 1.85147E-08 |
| PRSS1 | -1.226978461 | 1.87438E-08 |
| PRO1804 | 1.418787649 | 1.90352E-08 |
| RNU6-813P | -1.082585402 | 1.90482E-08 |
| Metazoa_SRP | 1.223425388 | 2.00104E-08 |
| AC004672.2 | 1.5272098 | 2.0096E-08 |
| RPL7L1P12 | 1.430635948 | 2.1116E-08 |
| LINC01551 | 1.560668671 | 2.12863E-08 |
| AP000781.2 | 1.048332997 | 2.23735E-08 |
| SLC47A1P1 | 1.066929678 | 2.25652E-08 |
| AC018742.1 | 1.032626173 | 2.26152E-08 |
| MIR140 | 1.504628039 | 2.27002E-08 |
| LPP-AS1 | 1.934784326 | 2.27154E-08 |
| AP002762.2 | 1.39925816 | 2.30218E-08 |
| MAGEA12 | -1.960714671 | 2.36853E-08 |
| NPY4R2 | 1.378995046 | 2.37839E-08 |
| AL356423.1 | 1.457811946 | 2.38519E-08 |
| AL590617.1 | 1.097687352 | 2.39096E-08 |
| AL023755.1 | 1.128460467 | 2.39563E-08 |
| KIF5C-AS1 | 1.095544225 | 2.45283E-08 |
| MIR137HG | 1.299979621 | 2.46143E-08 |
| AC009951.5 | 1.750229387 | 2.46301E-08 |
| AC117465.1 | 1.139456446 | 2.49442E-08 |
| RC3H1-IT1 | 1.326549235 | 2.52479E-08 |
| AC093627.3 | 1.111840769 | 2.52688E-08 |
| PAGE2B | -1.149550957 | 2.54186E-08 |
| AC007100.1 | 1.762443287 | 2.56053E-08 |
| AC013564.1 | 1.937739984 | 2.60749E-08 |
| MTND1P36 | 1.853756186 | 2.61155E-08 |
| AL512326.3 | 1.274461069 | 2.66556E-08 |
| OR10A2 | 1.097896654 | 2.68258E-08 |
| AC125437.2 | 1.305003968 | 2.73966E-08 |
| AC009152.3 | 1.089661753 | 2.75112E-08 |
| AC011504.1 | 1.567262464 | 2.85374E-08 |
| LCAL1 | -1.127911506 | 2.85608E-08 |
| Z97205.2 | 1.217197269 | 2.86632E-08 |
| LINC02834 | 1.315826575 | 2.9052E-08 |
| LINC00221 | -1.58778016 | 2.97178E-08 |
| RAD21L1 | -1.147014347 | 3.01692E-08 |
| AC117945.2 | -1.993638017 | 3.03264E-08 |
| RPS15AP13 | 2.010386191 | 3.08933E-08 |
| ATP6V0CP1 | 1.12595986 | 3.11543E-08 |
| AC090018.1 | 2.054945176 | 3.1827E-08 |
| AC007920.1 | 1.011319963 | 3.20827E-08 |
| LINC01324 | 2.039903831 | 3.21509E-08 |
| WWOX-AS1 | 1.140654867 | 3.42346E-08 |
| SPRR3 | -1.606022026 | 3.45198E-08 |
| AP003168.2 | 1.417692706 | 3.45723E-08 |
| AC091185.2 | 1.539699305 | 3.51115E-08 |
| TMEM30CP | 1.243426771 | 3.55303E-08 |
| BCAR4 | -1.0903422 | 3.60575E-08 |
| AP001318.3 | 1.885584707 | 3.64286E-08 |
| Metazoa_SRP | 1.507942516 | 3.65236E-08 |
| AC091305.1 | -1.15601836 | 3.65599E-08 |
| DDI1 | 2.030289525 | 3.67484E-08 |
| GFY | -1.082492796 | 3.67521E-08 |
| HIGD1C | 1.059279315 | 3.67653E-08 |
| AC074024.1 | 2.290906823 | 3.76111E-08 |
| LINC00348 | 2.002544286 | 3.76728E-08 |
| U2 | -1.079735362 | 3.80792E-08 |
| AC007326.1 | 1.163747094 | 3.85653E-08 |
| SMG6-IT1 | 2.264190477 | 3.86329E-08 |
| AC020741.1 | 1.193819103 | 3.90857E-08 |
| AL391097.2 | 1.210536406 | 3.9103E-08 |
| NEUROG3 | -1.026135233 | 4.00924E-08 |
| ANKRD20A19P | -1.13165337 | 4.02559E-08 |
| EDDM3A | -1.081137023 | 4.04983E-08 |
| AL627308.3 | 1.317920329 | 4.08725E-08 |
| LHX5 | 1.207759499 | 4.10131E-08 |
| AP003327.2 | -1.193076854 | 4.10808E-08 |
| AL356320.1 | 1.369913258 | 4.11486E-08 |
| RN7SL748P | 1.254079965 | 4.16062E-08 |
| JAKMIP2-AS1 | 1.084934014 | 4.17742E-08 |
| JTBP1 | 1.070747276 | 4.18498E-08 |
| CACNA1C-IT3 | 1.870993403 | 4.20072E-08 |
| AC011242.1 | 2.171807198 | 4.25163E-08 |
| SMIM32 | -1.122869666 | 4.28254E-08 |
| AC108073.3 | 1.069185406 | 4.32244E-08 |
| Z98749.1 | 1.125891958 | 4.38294E-08 |
| MTND5P2 | 1.490506145 | 4.43274E-08 |
| RBM46 | 1.005406063 | 4.4453E-08 |
| AC034105.3 | 1.262698887 | 4.50917E-08 |
| KCTD9P5 | 1.951069603 | 4.5095E-08 |
| AC092436.3 | 1.273178316 | 4.54191E-08 |
| AC245041.1 | 1.006468733 | 4.60121E-08 |
| HSPB2 | 1.005499227 | 4.62877E-08 |
| AC022973.1 | 1.710340342 | 4.71472E-08 |
| CKAP2LP1 | 1.384764203 | 4.78307E-08 |
| AC100793.1 | 1.223792689 | 4.86453E-08 |
| FGA | -1.720849732 | 4.88178E-08 |
| AL355852.1 | 1.227405764 | 4.92179E-08 |
| POU4F1 | 1.393943421 | 4.95572E-08 |
| AC107029.2 | 1.601192959 | 5.11861E-08 |
| AC091046.1 | 1.374203719 | 5.25676E-08 |
| ONECUT1 | -1.086483193 | 5.3402E-08 |
| AC068647.1 | 1.359704742 | 5.34622E-08 |
| AL391097.3 | 1.48956714 | 5.47384E-08 |
| AC104232.2 | 2.332874944 | 5.53497E-08 |
| AC003989.1 | 1.68848536 | 5.61294E-08 |
| Z99127.2 | 1.347107039 | 5.61823E-08 |
| KRT16P4 | 1.154491268 | 5.7312E-08 |
| Y_RNA | 1.187329918 | 5.75351E-08 |
| NDUFAF4P4 | 1.986088935 | 5.88665E-08 |
| COX6CP10 | 1.244326565 | 6.27977E-08 |
| AC087501.2 | 1.052616778 | 6.29536E-08 |
| TVP23CP1 | 1.523459069 | 6.40202E-08 |
| PAGE5 | -1.244094119 | 6.41377E-08 |
| RHOXF1P1 | -1.159219867 | 6.5411E-08 |
| AC016542.3 | 2.220314876 | 6.95258E-08 |
| PTMAP6 | 1.521332525 | 6.95361E-08 |
| GAB4 | 1.127177197 | 7.00243E-08 |
| AC006023.1 | 1.071741973 | 7.03534E-08 |
| AC037450.1 | 1.297565442 | 7.06479E-08 |
| BPIFA4P | -1.480668638 | 7.0984E-08 |
| AL161909.2 | 1.245579946 | 7.21395E-08 |
| F11 | 1.1316296 | 7.39297E-08 |
| AL161644.1 | 1.513412945 | 7.50581E-08 |
| RNU6-414P | 1.708986607 | 7.60286E-08 |
| AC239600.1 | 1.131704059 | 7.61293E-08 |
| MTND4P32 | 1.883417864 | 7.8121E-08 |
| AC091805.1 | 1.427005879 | 7.8298E-08 |
| RPL36AP33 | 1.32612502 | 7.89653E-08 |
| AL845331.2 | 1.081976435 | 8.05307E-08 |
| AC096708.2 | 1.033602244 | 8.11353E-08 |
| AC010886.1 | 1.613078445 | 8.14985E-08 |
| LINC02744 | 1.047052817 | 8.18337E-08 |
| AC092894.1 | 2.051091031 | 8.29231E-08 |
| RPL6P12 | 1.522909954 | 8.34474E-08 |
| AC004485.1 | 2.079977542 | 8.41668E-08 |
| KHDC3L | 1.120309051 | 8.43721E-08 |
| CASP14 | -1.210767192 | 8.44792E-08 |
| OR2L1P | 1.019908809 | 8.46764E-08 |
| SSTR5-AS1 | -1.269489172 | 8.53685E-08 |
| COX6CP17 | 2.249576551 | 8.57131E-08 |
| AC114964.2 | -1.193080089 | 8.59492E-08 |
| DTD1-AS1 | 2.130795556 | 8.62138E-08 |
| LINC02434 | 2.225293713 | 8.9225E-08 |
| CNOT10-AS1 | 1.454120182 | 8.97595E-08 |
| AC048380.1 | 1.286945473 | 8.98946E-08 |
| LINC02630 | 1.67949176 | 9.0192E-08 |
| AC131391.1 | 1.084908347 | 9.15802E-08 |
| AC011840.1 | -1.754879309 | 9.18635E-08 |
| AC010287.1 | 1.378319413 | 9.20734E-08 |
| AC019127.1 | 1.060257042 | 9.37847E-08 |
| SYNPR | 1.069006578 | 9.38498E-08 |
| C10orf71 | -1.771728351 | 9.38894E-08 |
| AC093297.1 | -1.278594924 | 9.42171E-08 |
| OR6B3 | 1.018859458 | 9.45811E-08 |
| AC037479.1 | 1.063079378 | 9.5678E-08 |
| AC092120.2 | 1.445638758 | 9.58182E-08 |
| MED15P4 | -1.183448292 | 9.85309E-08 |
| LINC01502 | 1.204928181 | 9.89328E-08 |
| AC010997.6 | 1.623972771 | 1.00449E-07 |
| RNU6-1161P | 1.143109537 | 1.00838E-07 |
| AC017006.2 | 1.298586356 | 1.02211E-07 |
| AL353693.1 | 1.096387724 | 1.03309E-07 |
| MIR4269 | 1.043912561 | 1.03351E-07 |
| AL162584.1 | 1.285961206 | 1.03417E-07 |
| PAFAH1B1P1 | 1.209961248 | 1.0344E-07 |
| AC007621.3 | 1.211266364 | 1.0344E-07 |
| Y_RNA | 1.465463257 | 1.048E-07 |
| AC105760.1 | 1.117165585 | 1.04873E-07 |
| AP002439.1 | 1.571990538 | 1.05646E-07 |
| AC091163.1 | 1.01714224 | 1.05917E-07 |
| TSPAN9-IT1 | 1.923550562 | 1.06311E-07 |
| AC079384.1 | -1.237562157 | 1.06834E-07 |
| CREB3L2-AS1 | 1.283674949 | 1.07332E-07 |
| SUMO1P1 | -1.101174699 | 1.0749E-07 |
| AC022509.5 | 1.396076954 | 1.08289E-07 |
| AC010967.1 | 1.098082221 | 1.08506E-07 |
| AL589863.1 | 2.887192735 | 1.08796E-07 |
| SNX18P7 | -1.533210233 | 1.09369E-07 |
| COL18A1-AS2 | 1.450302511 | 1.09777E-07 |
| AL390237.1 | 1.17871678 | 1.13908E-07 |
| AC011595.1 | 1.043664676 | 1.13944E-07 |
| AC009303.3 | 1.869216613 | 1.15596E-07 |
| AL365255.1 | -1.034706106 | 1.1604E-07 |
| ATP5PFP1 | -1.022724977 | 1.1604E-07 |
| AC120349.2 | 1.77446676 | 1.16913E-07 |
| DOCK9-AS1 | 1.6989358 | 1.18155E-07 |
| CHL1-AS1 | 1.010119576 | 1.18422E-07 |
| Y_RNA | 2.121705801 | 1.19405E-07 |
| AC112243.1 | -1.843157151 | 1.20768E-07 |
| AKT3-IT1 | 1.128277945 | 1.21609E-07 |
| Y_RNA | 1.239131446 | 1.2584E-07 |
| CACYBPP1 | 1.093401561 | 1.26143E-07 |
| AL450313.1 | 1.033018127 | 1.26533E-07 |
| PSMA2P2 | 1.562727925 | 1.26746E-07 |
| AL137857.1 | 1.205985383 | 1.32648E-07 |
| AC060773.1 | 1.567564514 | 1.35021E-07 |
| AC005344.1 | 2.467652652 | 1.36056E-07 |
| CHP1P1 | 1.49248799 | 1.36198E-07 |
| AC096921.1 | 1.67617999 | 1.37713E-07 |
| AL591242.1 | 1.022889413 | 1.40374E-07 |
| AC011406.1 | 1.387712612 | 1.42044E-07 |
| AL138733.1 | 1.032272447 | 1.42473E-07 |
| AC006372.1 | -1.017787383 | 1.43428E-07 |
| AC118465.1 | 1.131262669 | 1.44594E-07 |
| AC105429.2 | 1.469674035 | 1.45241E-07 |
| AL121950.1 | -1.06034922 | 1.46306E-07 |
| RNU6-998P | 1.523936676 | 1.47496E-07 |
| AC113385.1 | 1.116139452 | 1.49243E-07 |
| GATA4 | -1.27140915 | 1.49567E-07 |
| SPDYC | -1.163987554 | 1.50993E-07 |
| AC026462.5 | -1.127288279 | 1.51048E-07 |
| AC091046.2 | 1.999717594 | 1.51536E-07 |
| CHRM3-AS1 | 1.937658786 | 1.51966E-07 |
| AP005205.2 | 1.017506795 | 1.52472E-07 |
| AC011298.1 | -1.454782742 | 1.55742E-07 |
| AC009262.1 | -1.055278285 | 1.56285E-07 |
| AC009060.1 | 1.053360703 | 1.60511E-07 |
| SAR1AP2 | 2.201134399 | 1.60591E-07 |
| ZBTB46-AS1 | 1.299818423 | 1.61459E-07 |
| AL445489.1 | 1.573952176 | 1.62881E-07 |
| KRT35 | -1.147916294 | 1.637E-07 |
| HSPA8P19 | 1.230767736 | 1.67652E-07 |
| AL110505.1 | 1.558842261 | 1.67652E-07 |
| AL117382.1 | 1.167241934 | 1.67877E-07 |
| AC073359.1 | 1.195555856 | 1.69793E-07 |
| PPIAP52 | 1.090249775 | 1.76569E-07 |
| AP000561.1 | 2.046678014 | 1.78684E-07 |
| AC012213.1 | -1.240307392 | 1.79679E-07 |
| AC024619.3 | 1.073204196 | 1.82143E-07 |
| AC017101.1 | 1.423370243 | 1.82987E-07 |
| KLK11 | 1.076250824 | 1.83536E-07 |
| AL596223.1 | 1.068068053 | 1.84615E-07 |
| AC005828.2 | 1.472510218 | 1.8561E-07 |
| AC016931.1 | 1.221805406 | 1.8816E-07 |
| AC007533.1 | 1.08524023 | 1.88296E-07 |
| AL360007.1 | 1.513389117 | 1.92232E-07 |
| NCR2 | 1.048879827 | 1.92739E-07 |
| GPR1-AS | 1.420816628 | 1.96754E-07 |
| CPB1 | -1.252110533 | 1.9837E-07 |
| CARM1P1 | 1.413013513 | 2.07389E-07 |
| AC092910.2 | 2.638329266 | 2.09612E-07 |
| YWHAQP5 | 1.033134704 | 2.09813E-07 |
| FAM205C | 1.009230038 | 2.10171E-07 |
| AL160274.1 | 1.432101029 | 2.18962E-07 |
| AC011700.1 | 1.192151818 | 2.19902E-07 |
| RFX6 | 1.070209628 | 2.25904E-07 |
| ZNF280A | 1.346339878 | 2.26048E-07 |
| RNU6-606P | 1.731329778 | 2.28151E-07 |
| AL360091.2 | 1.162433343 | 2.3705E-07 |
| AL391361.2 | 1.133115748 | 2.38659E-07 |
| AL121750.1 | 1.399972184 | 2.38866E-07 |
| AP000432.2 | 1.495333821 | 2.41005E-07 |
| AL357832.1 | 1.141812897 | 2.41475E-07 |
| LINC00237 | 1.077224721 | 2.4564E-07 |
| LINC01090 | 1.134831193 | 2.4583E-07 |
| AC091059.2 | 1.11164854 | 2.47703E-07 |
| AC090950.2 | 1.082865604 | 2.49146E-07 |
| AC005293.1 | 1.300490305 | 2.56094E-07 |
| AIG1P1 | 2.13391284 | 2.56096E-07 |
| RN7SKP237 | 1.379431115 | 2.5868E-07 |
| LINC00351 | -2.366921725 | 2.64747E-07 |
| AC002069.2 | -1.026805444 | 2.65753E-07 |
| PRSS52P | 1.234184549 | 2.75813E-07 |
| GLIS3-AS1 | 1.095730314 | 2.79531E-07 |
| PMCHL2 | -1.187791143 | 2.83571E-07 |
| LINC01647 | -1.25396909 | 2.84271E-07 |
| AC073578.5 | -1.254857863 | 2.8697E-07 |
| ELFN2 | -1.086350514 | 2.89624E-07 |
| AC069228.1 | -1.343835146 | 2.90681E-07 |
| SMAD1-AS2 | 1.041375 | 2.95239E-07 |
| AC115837.1 | -1.361068965 | 2.96118E-07 |
| AC126768.2 | 1.139627 | 2.96848E-07 |
| SOX14 | 2.159252236 | 2.9972E-07 |
| HTR3B | 1.0575891 | 3.06423E-07 |
| AC027129.1 | 1.308115861 | 3.39335E-07 |
| AL513188.1 | 1.439668141 | 3.42145E-07 |
| AC003989.2 | 1.906834871 | 3.46133E-07 |
| AL109809.2 | 1.189694584 | 3.46575E-07 |
| HOXB-AS4 | 1.047964567 | 3.471E-07 |
| EIF4A1P11 | 1.208247204 | 3.5435E-07 |
| MTNR1A | 1.033712012 | 3.58752E-07 |
| KRT16P5 | 1.418093991 | 3.59304E-07 |
| RN7SKP185 | 1.266557136 | 3.64999E-07 |
| ATXN8OS | -1.726102406 | 3.64999E-07 |
| AC004865.1 | 1.232150323 | 3.68555E-07 |
| FTHL17 | 3.199584279 | 3.72387E-07 |
| AL031291.1 | 1.266124909 | 3.77494E-07 |
| AL121759.2 | 1.926147593 | 3.84715E-07 |
| AC110753.1 | 1.713236741 | 3.91226E-07 |
| AL596330.1 | 1.40888348 | 4.02051E-07 |
| PRB4 | 1.656329587 | 4.03703E-07 |
| AP006222.2 | -1.11794974 | 4.08919E-07 |
| AC007336.3 | 1.104545375 | 4.1142E-07 |
| AC006058.2 | 1.64099565 | 4.11596E-07 |
| NTM-AS1 | 1.443809549 | 4.1444E-07 |
| HMGN1P12 | 1.230805654 | 4.15079E-07 |
| AC104663.1 | -1.703990743 | 4.26344E-07 |
| AC012146.3 | 1.159031058 | 4.3018E-07 |
| RPS27AP7 | 1.169858139 | 4.31438E-07 |
| AL078599.2 | 1.610564709 | 4.31854E-07 |
| RPL35AP2 | 1.307304313 | 4.3624E-07 |
| AL445686.1 | 1.324973675 | 4.37443E-07 |
| CYP2A7 | -1.1233534 | 4.42962E-07 |
| AL121974.1 | -1.038189238 | 4.48994E-07 |
| RNU6-942P | 1.263384228 | 4.5285E-07 |
| DPRXP1 | 1.369215947 | 4.54014E-07 |
| AL109824.1 | 1.0400967 | 4.54544E-07 |
| PNMA6B | 1.013325664 | 4.57391E-07 |
| AC106743.1 | 1.246480941 | 4.61582E-07 |
| AL138826.1 | -1.973433056 | 4.74828E-07 |
| AC113423.2 | -1.015465641 | 4.75252E-07 |
| ZNF75BP | 2.641454999 | 4.8298E-07 |
| AC078962.3 | 1.605412607 | 4.83735E-07 |
| AC010731.1 | 1.000783507 | 4.8706E-07 |
| AC011939.2 | 1.116269052 | 4.88129E-07 |
| PRSS48 | 1.244028165 | 4.92676E-07 |
| GPR151 | 1.207398721 | 4.94339E-07 |
| LINC01259 | 1.036323398 | 4.94647E-07 |
| AL450226.1 | 1.436204297 | 4.98562E-07 |
| AC093894.2 | 1.65625997 | 5.20013E-07 |
| AC104237.1 | 1.005883059 | 5.23036E-07 |
| AL136982.5 | -1.518647253 | 5.23305E-07 |
| AC016493.1 | 1.951724152 | 5.24385E-07 |
| AC055733.1 | 1.646251871 | 5.25806E-07 |
| AP002490.2 | 1.651203815 | 5.26207E-07 |
| AL161431.1 | 1.793293171 | 5.31847E-07 |
| HTR1DP1 | 1.12092015 | 5.38881E-07 |
| AP002884.1 | 1.300233348 | 5.45101E-07 |
| AC055764.1 | 1.331871035 | 5.48824E-07 |
| AC098826.2 | 1.442175319 | 5.6259E-07 |
| AL450327.1 | 1.155161457 | 5.74109E-07 |
| AL807757.1 | 1.33011912 | 5.77442E-07 |
| YY1P1 | 1.468734269 | 5.8015E-07 |
| SCRT2 | 1.708630239 | 5.89233E-07 |
| SIX3-AS1 | 1.193264761 | 5.94704E-07 |
| AL136979.1 | 1.077177694 | 6.06957E-07 |
| AL589986.2 | 1.194456565 | 6.13154E-07 |
| KLF4P1 | 1.688204895 | 6.23203E-07 |
| TMEFF1 | 1.049122495 | 6.28395E-07 |
| AC090241.1 | 1.182890748 | 6.30928E-07 |
| DISC1-IT1 | 1.141368665 | 6.47308E-07 |
| LHCGR | 1.01162193 | 6.59773E-07 |
| LINC00488 | 1.606167803 | 6.6357E-07 |
| RPS15AP39 | 1.397807272 | 6.68192E-07 |
| AC018946.1 | 1.257484119 | 6.69456E-07 |
| AC027251.1 | 1.903304833 | 6.72496E-07 |
| AC018714.1 | 1.336532158 | 6.72988E-07 |
| KCNK16 | -1.478926846 | 6.9063E-07 |
| MIR548V | 1.175787162 | 6.92646E-07 |
| RPEP4 | 1.064868044 | 6.94615E-07 |
| AL354754.1 | -2.035773518 | 7.02402E-07 |
| PALLD-AS1 | 1.649956145 | 7.02617E-07 |
| LINC02868 | -1.023549622 | 7.02884E-07 |
| AC097709.1 | 1.485736181 | 7.03528E-07 |
| NAALADL2-AS3 | 1.427656348 | 7.16804E-07 |
| OR7E136P | 1.058669961 | 7.22695E-07 |
| RNU4ATAC16P | 1.671129818 | 7.34095E-07 |
| LINC01924 | -1.321553146 | 7.39639E-07 |
| MIR4256 | 1.036646902 | 7.46947E-07 |
| AC080128.1 | 1.26507281 | 7.57655E-07 |
| TIMM9P1 | 1.743698965 | 7.5987E-07 |
| AC092580.2 | 1.14709981 | 7.83542E-07 |
| AC079866.2 | 1.192953369 | 7.89852E-07 |
| SLC25A6P3 | 1.889494607 | 7.89877E-07 |
| RNU6-123P | 1.298813577 | 7.9298E-07 |
| AL163193.1 | 1.208586745 | 7.94942E-07 |
| AC110603.1 | 2.71275806 | 7.95114E-07 |
| AL356805.1 | 1.622881355 | 8.09128E-07 |
| AC090971.4 | 1.034523154 | 8.30487E-07 |
| XXYLT1-AS1 | 1.048941591 | 8.31295E-07 |
| RPL7L1P2 | 1.529032004 | 8.41703E-07 |
| AL645568.1 | 1.084826149 | 8.50372E-07 |
| AC037487.2 | 1.033881908 | 8.50981E-07 |
| MIR581 | 1.377063097 | 8.56503E-07 |
| CYCSP4 | 1.579186925 | 8.6387E-07 |
| NDUFA8P1 | 2.238431944 | 8.84876E-07 |
| AC002381.1 | 2.250305275 | 8.88615E-07 |
| AC106827.1 | 1.654878243 | 8.94797E-07 |
| Y_RNA | 1.39186051 | 9.04437E-07 |
| GUCY2F | 1.064270901 | 9.06756E-07 |
| RNU6-674P | 1.771055738 | 9.09124E-07 |
| CCT4P2 | 1.269697291 | 9.19314E-07 |
| MS4A6E | 1.034733084 | 9.23479E-07 |
| EIF4E1B | -1.093081931 | 9.23653E-07 |
| AHSG | 1.239292065 | 9.29927E-07 |
| ACCSL | 1.061352846 | 9.50408E-07 |
| AL606517.1 | 2.006870764 | 9.58308E-07 |
| LINC02287 | -1.221624079 | 9.7586E-07 |
| AC104982.2 | 1.259403806 | 9.8742E-07 |
| FOXN3-AS2 | 1.999026663 | 1.01106E-06 |
| FO393419.2 | 1.432042361 | 1.0208E-06 |
| Y_RNA | 1.742509485 | 1.04733E-06 |
| AC004974.1 | 1.628465767 | 1.05101E-06 |
| SLC5A7 | -1.095588235 | 1.07458E-06 |
| AC005144.1 | 1.184829339 | 1.08557E-06 |
| NANOGP5 | 1.133756927 | 1.10943E-06 |
| AC243972.2 | -1.367581683 | 1.11402E-06 |
| AC074375.1 | 1.56281821 | 1.18306E-06 |
| AL357146.1 | 1.008100182 | 1.18519E-06 |
| AC008277.2 | 1.464481258 | 1.19882E-06 |
| NUP35P1 | 1.586262838 | 1.2218E-06 |
| CACNG6 | -1.085731091 | 1.23569E-06 |
| AL139280.3 | 1.67587653 | 1.25027E-06 |
| AC012555.2 | 1.578566125 | 1.2586E-06 |
| PCAT5 | 1.089996087 | 1.26467E-06 |
| MIR548O | 1.108370867 | 1.29432E-06 |
| AC016383.2 | 1.973549186 | 1.29437E-06 |
| AC129915.3 | 1.0382102 | 1.3077E-06 |
| RPL17P17 | 1.594740416 | 1.32821E-06 |
| CDC20B | -1.026549813 | 1.35513E-06 |
| SLC22A24 | -1.146664262 | 1.35955E-06 |
| LARS2-AS1 | 1.109427838 | 1.38396E-06 |
| OR7E121P | 1.581919311 | 1.39535E-06 |
| TPM3P1 | 1.051014559 | 1.43246E-06 |
| AL159159.1 | 1.289761142 | 1.44331E-06 |
| TRIM15 | 1.001008139 | 1.44466E-06 |
| LINC01549 | 1.040863914 | 1.44929E-06 |
| DPRXP3 | 1.000494392 | 1.46525E-06 |
| LINC01768 | -1.025527413 | 1.4755E-06 |
| AP003117.2 | 1.109731549 | 1.50213E-06 |
| AC022874.1 | -1.354592946 | 1.50861E-06 |
| AC096736.3 | 1.541903199 | 1.52444E-06 |
| RNGTTP1 | 1.137320791 | 1.52934E-06 |
| SLC2A2 | 1.774810842 | 1.53557E-06 |
| PDZRN3-AS1 | 1.155675205 | 1.55354E-06 |
| RN7SKP161 | 1.088388322 | 1.57182E-06 |
| SELENOOLP | 2.141226156 | 1.6064E-06 |
| AC092954.1 | 1.018144413 | 1.65761E-06 |
| OR7E26P | 1.10455049 | 1.68596E-06 |
| AC012568.1 | 1.397254728 | 1.68666E-06 |
| AC007601.2 | 1.293976669 | 1.69887E-06 |
| RPS3AP28 | 1.143612574 | 1.75919E-06 |
| PRELID3BP4 | 1.098915367 | 1.836E-06 |
| MRPS6P2 | 1.751697124 | 1.86575E-06 |
| CR545473.1 | 1.742211356 | 1.86833E-06 |
| LINC02269 | 1.159974756 | 1.88132E-06 |
| AL359916.1 | 1.12384239 | 1.93813E-06 |
| AC006529.1 | 1.045094632 | 1.93826E-06 |
| Y_RNA | 2.138253499 | 1.94007E-06 |
| CYP2A13 | -1.043374097 | 1.96404E-06 |
| MIR135B | 1.33654665 | 1.99452E-06 |
| AL359955.1 | 1.284894207 | 2.01774E-06 |
| PRKX-AS1 | 1.14181104 | 2.04364E-06 |
| AC244131.1 | 1.702546244 | 2.05174E-06 |
| AC115622.1 | -2.194476456 | 2.07642E-06 |
| TRIM10 | 1.018251637 | 2.12443E-06 |
| AL451047.1 | 1.148910983 | 2.12926E-06 |
| PDHA1P1 | 1.355746211 | 2.13042E-06 |
| LINC02672 | -1.183516649 | 2.13297E-06 |
| AC060834.2 | 2.131307729 | 2.17998E-06 |
| MIR1296 | 1.649808611 | 2.19813E-06 |
| AC124784.1 | 1.055975851 | 2.21467E-06 |
| AL359233.1 | 1.101777263 | 2.24124E-06 |
| C20orf85 | -1.337361464 | 2.30367E-06 |
| AC019185.1 | 1.016623864 | 2.32641E-06 |
| Metazoa_SRP | 1.400893721 | 2.36607E-06 |
| AP003467.1 | 1.067327046 | 2.36754E-06 |
| AL137224.1 | 1.757916091 | 2.38968E-06 |
| WFDC10A | -1.239772113 | 2.4277E-06 |
| AC079209.2 | 1.686696859 | 2.42966E-06 |
| AC037433.1 | -1.442949674 | 2.45464E-06 |
| RBISP2 | 1.11459062 | 2.50119E-06 |
| AC004130.1 | 1.286847181 | 2.50283E-06 |
| PNPLA5 | 1.255070216 | 2.54287E-06 |
| LINC00706 | 1.776614274 | 2.56702E-06 |
| LINC01310 | 1.193987171 | 2.63532E-06 |
| C15orf32 | 1.161644712 | 2.63587E-06 |
| AL590103.1 | 1.793352335 | 2.63721E-06 |
| DAB1-AS1 | 1.673975881 | 2.6445E-06 |
| LINC02843 | 1.079589129 | 2.65068E-06 |
| LINC02697 | 1.24411049 | 2.67503E-06 |
| SST | 1.536825238 | 2.71326E-06 |
| NPIPA7 | 1.508910288 | 2.76754E-06 |
| AL096869.1 | 1.154397883 | 2.7821E-06 |
| AL450226.2 | 1.070562216 | 2.79729E-06 |
| AC020661.2 | 1.305306517 | 2.81484E-06 |
| AC116096.1 | 1.212387145 | 2.81553E-06 |
| AC073140.1 | 1.350410516 | 2.84227E-06 |
| AC010595.1 | -1.597211623 | 2.91855E-06 |
| GJA6P | 2.361230651 | 2.98893E-06 |
| AC099552.4 | 1.033536862 | 3.02906E-06 |
| LINC02806 | 1.096306156 | 3.04325E-06 |
| AC016065.2 | 1.024610125 | 3.0457E-06 |
| AL450344.1 | 1.671793208 | 3.16058E-06 |
| OR7E99P | 2.029852812 | 3.16831E-06 |
| AC114878.2 | 1.177932549 | 3.19519E-06 |
| SNORD116 | 1.392451596 | 3.20927E-06 |
| AC106053.1 | 1.207113776 | 3.2158E-06 |
| RCC2P4 | 1.402055512 | 3.24787E-06 |
| AP002961.1 | 1.171909321 | 3.43043E-06 |
| AL391863.1 | 1.150538253 | 3.49526E-06 |
| VN2R10P | 1.018401418 | 3.54048E-06 |
| DAPK1-IT1 | 1.452796714 | 3.56223E-06 |
| AC018889.1 | 1.246389412 | 3.59828E-06 |
| RPL37P3 | 1.230018848 | 3.63207E-06 |
| RNA5SP101 | -1.036419004 | 3.64667E-06 |
| AC115621.1 | 1.598884242 | 3.6767E-06 |
| GPR139 | -1.145924446 | 3.68368E-06 |
| VPS26BP1 | 1.47644648 | 3.72529E-06 |
| CDX2 | 1.15321866 | 3.74051E-06 |
| Z99127.1 | 1.058056862 | 3.75319E-06 |
| RPL22P21 | 1.669520633 | 3.75966E-06 |
| Z82185.1 | -1.21777248 | 3.81427E-06 |
| AC091133.5 | -1.03016927 | 3.96643E-06 |
| RNY4 | 2.171256925 | 4.00911E-06 |
| AC016734.1 | 1.38679654 | 4.02931E-06 |
| RNA5SP491 | 1.084434894 | 4.04091E-06 |
| AP000897.1 | 2.220613967 | 4.04437E-06 |
| PDE4B-AS1 | 2.080319359 | 4.05272E-06 |
| ZBTB20-AS3 | 2.050258631 | 4.06541E-06 |
| F9 | -1.838930229 | 4.06971E-06 |
| AC092448.1 | 1.620926437 | 4.10286E-06 |
| AC106707.2 | 1.319471485 | 4.12691E-06 |
| AC061961.1 | -1.097397541 | 4.33283E-06 |
| AC022022.2 | 1.810520946 | 4.37273E-06 |
| AC025125.1 | 1.139706877 | 4.37719E-06 |
| AC129926.2 | -2.44677172 | 4.4093E-06 |
| GPR101 | 1.084101744 | 4.57257E-06 |
| DUTP7 | 1.031144002 | 4.61826E-06 |
| AC079316.1 | 1.790162094 | 4.68343E-06 |
| AP001922.3 | 1.046967542 | 4.71189E-06 |
| DDX3P2 | 1.528666324 | 4.92146E-06 |
| LINC01639 | -1.558739092 | 5.05272E-06 |
| LINC02645 | -1.168028283 | 5.08038E-06 |
| AC084783.1 | 1.161047124 | 5.08329E-06 |
| LINC02844 | -1.119051072 | 5.12349E-06 |
| AC018607.1 | 1.674571995 | 5.19939E-06 |
| AC010857.1 | -1.09821867 | 5.32924E-06 |
| AC005357.2 | 1.088564224 | 5.36631E-06 |
| AC068389.3 | 1.098046472 | 5.41224E-06 |
| FABP7P1 | 1.362185 | 5.5108E-06 |
| RNA5SP44 | 1.557770367 | 5.58603E-06 |
| FAM204CP | 2.062654238 | 5.60245E-06 |
| AC020915.4 | 1.472030034 | 5.61225E-06 |
| LCE3A | 1.177279586 | 5.66967E-06 |
| AC017037.2 | -1.183173654 | 5.78069E-06 |
| LINC02617 | 1.12641405 | 5.80229E-06 |
| DMBT1L1 | 1.071323076 | 5.87475E-06 |
| IFNK | 1.423958092 | 5.91709E-06 |
| LINC00332 | 1.128884503 | 5.92081E-06 |
| LINC02197 | -1.231262203 | 5.9288E-06 |
| CYP2A7P1 | -1.650424002 | 6.01665E-06 |
| MIR6510 | 1.037308314 | 6.11518E-06 |
| RN7SKP95 | 1.580139623 | 6.24047E-06 |
| STX18-IT1 | 1.179367826 | 6.28737E-06 |
| RNU6-1176P | 1.176146082 | 6.38663E-06 |
| RNU6-60P | 2.228468184 | 6.43757E-06 |
| LINC02837 | 1.559229528 | 6.55703E-06 |
| PAX7 | -1.152306921 | 6.61346E-06 |
| UGT2B10 | -1.147109901 | 6.61722E-06 |
| AL831737.1 | 1.140887729 | 6.87621E-06 |
| AC005998.1 | 1.10368347 | 6.8841E-06 |
| PRB2 | 1.650473589 | 7.06508E-06 |
| AL355974.1 | 1.241826699 | 7.22737E-06 |
| PHF5GP | 1.135161922 | 7.26131E-06 |
| AL353662.2 | 1.212171737 | 7.37261E-06 |
| LCN8 | 1.0945202 | 7.50564E-06 |
| AC120498.3 | -1.079182448 | 7.52665E-06 |
| AC006398.1 | 1.872139207 | 7.71247E-06 |
| PGK2 | -1.060499152 | 7.72983E-06 |
| AC011753.2 | 1.219674792 | 7.73938E-06 |
| RNY3P13 | 1.549003303 | 7.83236E-06 |
| S100A7A | -1.341826475 | 7.86086E-06 |
| AC012354.2 | 1.059012801 | 7.87267E-06 |
| AC079316.2 | 1.135330675 | 7.91024E-06 |
| AC108075.1 | 1.268112783 | 7.96773E-06 |
| RPS15AP29 | 1.18913282 | 8.3033E-06 |
| AC117464.1 | -1.344575701 | 8.31839E-06 |
| AC100791.1 | -1.015791588 | 8.7301E-06 |
| ARHGAP26-IT1 | 1.372220059 | 8.85478E-06 |
| AC012354.7 | 1.057003618 | 8.85773E-06 |
| RNU2-6P | 1.111660528 | 9.01328E-06 |
| AC087821.1 | 1.15142173 | 9.04449E-06 |
| RNU4ATAC11P | 1.776548939 | 9.12647E-06 |
| LINC02484 | -2.555125914 | 9.14648E-06 |
| AC007599.1 | 1.799393524 | 9.20311E-06 |
| BAGE2 | -1.610912252 | 9.21487E-06 |
| CSN1S2AP | 3.649726508 | 9.50256E-06 |
| AP000919.2 | 1.001600446 | 9.62013E-06 |
| PSG1 | 1.468866085 | 9.74953E-06 |
| AL157385.1 | 1.171577941 | 9.77341E-06 |
| AL365318.1 | 1.098146088 | 9.85508E-06 |
| KRT16P3 | 1.049232447 | 9.89569E-06 |
| Y_RNA | 1.037601947 | 9.92649E-06 |
| AC099506.1 | 1.06162923 | 9.98138E-06 |
| AC073522.1 | -1.077662496 | 1.01258E-05 |
| AC005695.2 | 1.707866136 | 1.03102E-05 |
| AC007151.1 | 1.07573793 | 1.03415E-05 |
| AC090617.1 | 1.725852473 | 1.04607E-05 |
| APOBEC1 | -1.230638678 | 1.05143E-05 |
| KRT16P1 | 1.076417417 | 1.05318E-05 |
| AL359551.1 | 1.071666896 | 1.0836E-05 |
| GTF2IP6 | -2.25991916 | 1.12699E-05 |
| AL158819.1 | -1.204222254 | 1.15931E-05 |
| TCF4-AS1 | 1.298309904 | 1.17438E-05 |
| AC243773.1 | -1.725204115 | 1.17549E-05 |
| C8orf87 | -1.193536552 | 1.20227E-05 |
| BNIP3P18 | -1.351996415 | 1.20322E-05 |
| RN7SKP273 | 1.1412351 | 1.21662E-05 |
| AL034555.1 | 1.286652143 | 1.22481E-05 |
| AC241520.1 | 1.313276085 | 1.2279E-05 |
| TCL1B | -1.235790815 | 1.2409E-05 |
| AL591519.1 | 1.326320345 | 1.27985E-05 |
| LINC01574 | -1.550593206 | 1.28899E-05 |
| AC006254.3 | 1.169226679 | 1.29218E-05 |
| RN7SKP8 | 2.041921536 | 1.34301E-05 |
| VSTM2A | -1.176716107 | 1.36865E-05 |
| RN7SL801P | 2.227326116 | 1.38809E-05 |
| H3P9 | 1.222016977 | 1.40087E-05 |
| AC090820.2 | 1.20606526 | 1.40203E-05 |
| LBX1 | -1.419400871 | 1.40999E-05 |
| GPR149 | 1.467120124 | 1.41496E-05 |
| SNORD115 | 2.218137216 | 1.43869E-05 |
| AC108515.1 | -1.158440658 | 1.44529E-05 |
| AC027279.4 | 1.004696122 | 1.46341E-05 |
| AL357153.4 | 1.039630964 | 1.48873E-05 |
| RNU6-786P | 1.051239737 | 1.51843E-05 |
| AC079414.1 | -1.448095891 | 1.51898E-05 |
| RPS26P18 | 2.046221326 | 1.52684E-05 |
| AC132825.4 | 1.040779084 | 1.52715E-05 |
| AL117341.1 | 1.985103637 | 1.52826E-05 |
| CST8 | -1.246395943 | 1.53684E-05 |
| LINC02570 | 1.325682278 | 1.5554E-05 |
| DCAF4L2 | -2.060594889 | 1.55815E-05 |
| AC092916.1 | 2.474092976 | 1.56658E-05 |
| AC064843.1 | 1.307153975 | 1.56725E-05 |
| AL589655.1 | 1.751642009 | 1.58124E-05 |
| AC007448.3 | 1.408942196 | 1.60988E-05 |
| AL590632.1 | 1.00173453 | 1.6651E-05 |
| AC093915.1 | 1.007208681 | 1.67248E-05 |
| AC008810.1 | 1.239336691 | 1.67812E-05 |
| AC104009.1 | 1.31767146 | 1.67919E-05 |
| MAGEB1 | 1.484883245 | 1.68925E-05 |
| SNORA80A | 1.419449136 | 1.70792E-05 |
| RN7SL220P | 1.297880093 | 1.72165E-05 |
| AL109767.1 | 1.103203285 | 1.72639E-05 |
| AC017007.2 | 1.062168584 | 1.72693E-05 |
| 7SK | 1.699383131 | 1.76621E-05 |
| RPL17P40 | 1.265866235 | 1.76692E-05 |
| AC096552.1 | 1.158753011 | 1.76791E-05 |
| AL139193.1 | 1.6545717 | 1.77564E-05 |
| MTNR1B | -1.317963063 | 1.79364E-05 |
| SNORA70 | 1.911287484 | 1.82388E-05 |
| PHF2P2 | -1.170581224 | 1.86456E-05 |
| LINC01608 | -1.145731004 | 1.87027E-05 |
| AC007598.1 | 1.195168272 | 1.88841E-05 |
| RN7SL683P | 2.03046584 | 1.90675E-05 |
| PLCE1-AS2 | 1.044240539 | 1.91456E-05 |
| AC091073.1 | 1.423015294 | 1.95982E-05 |
| Z99289.3 | 1.063203857 | 1.97896E-05 |
| AC079988.1 | 1.52357072 | 2.00245E-05 |
| AC068647.2 | 1.118149486 | 2.04051E-05 |
| OR1N2 | 1.413578919 | 2.04085E-05 |
| AC091173.1 | -1.264583388 | 2.04914E-05 |
| AL512430.2 | 1.450556195 | 2.09265E-05 |
| TMEM229A | -1.22855318 | 2.1107E-05 |
| AL138885.1 | 1.04115233 | 2.12203E-05 |
| AC093083.1 | 1.38428713 | 2.18304E-05 |
| LINC02398 | 1.010285818 | 2.20097E-05 |
| AC100839.1 | 1.154471869 | 2.22709E-05 |
| AL669841.1 | 1.519537105 | 2.23182E-05 |
| AL033519.2 | 1.097901536 | 2.2687E-05 |
| KRT34 | 1.185086707 | 2.28847E-05 |
| AC018442.2 | 1.101927223 | 2.30833E-05 |
| RN7SKP299 | 1.163798617 | 2.39713E-05 |
| AC087276.3 | 1.051593747 | 2.43376E-05 |
| H1-1 | 1.113959098 | 2.43702E-05 |
| RSU1P3 | 2.322884647 | 2.5021E-05 |
| DUX4L9 | 1.1084532 | 2.50283E-05 |
| AC007923.1 | -1.437468706 | 2.51324E-05 |
| HSPE1P13 | 1.33176287 | 2.53273E-05 |
| GSTM5P1 | 1.004087996 | 2.59195E-05 |
| AC007106.2 | 1.053564898 | 2.61224E-05 |
| PAGE2 | -1.233861513 | 2.65647E-05 |
| AL022100.1 | 1.198206235 | 2.67567E-05 |
| U3 | 1.857629956 | 2.76363E-05 |
| TBCAP2 | 1.241041204 | 2.77055E-05 |
| AC005100.1 | 1.093857805 | 2.7904E-05 |
| AADACL3 | 1.256632738 | 2.79236E-05 |
| C7orf33 | 1.135103923 | 2.80543E-05 |
| AC139792.3 | 1.32798797 | 2.80608E-05 |
| LINC00469 | -1.112282007 | 2.87082E-05 |
| HRG | 1.029163853 | 2.94839E-05 |
| AC073050.1 | 1.131223218 | 2.96682E-05 |
| CCNJP2 | 1.892818535 | 2.97171E-05 |
| OR5P3 | 1.238138695 | 3.00732E-05 |
| AC110994.1 | 1.265834024 | 3.0704E-05 |
| RNVU1-18 | -3.180827428 | 3.11061E-05 |
| LINC02109 | -1.371015021 | 3.28459E-05 |
| AL138701.2 | 1.10838317 | 3.3031E-05 |
| AC004549.1 | 2.336864603 | 3.32742E-05 |
| AC105910.1 | 1.089631097 | 3.41422E-05 |
| AL049825.1 | 1.086527089 | 3.42563E-05 |
| LHFPL3-AS1 | 1.053106344 | 3.44624E-05 |
| AC003080.1 | 1.076016804 | 3.46569E-05 |
| IL20RB-AS1 | 1.159652453 | 3.48194E-05 |
| AL662889.1 | 1.969903036 | 3.54317E-05 |
| TEX55 | 1.188912166 | 3.55375E-05 |
| AL590704.1 | 1.871322559 | 3.6172E-05 |
| AC112196.1 | 1.092551696 | 3.69043E-05 |
| CST9 | -1.023722789 | 3.72328E-05 |
| AC112673.1 | 1.312693579 | 3.74104E-05 |
| SPRR1A | -1.109224533 | 3.76698E-05 |
| AL158136.1 | 1.026465409 | 3.7826E-05 |
| RPL37P18 | 1.736768419 | 3.79384E-05 |
| AC016044.1 | -1.279787629 | 3.90576E-05 |
| CLEC3A | -1.165200965 | 3.90899E-05 |
| AC079414.2 | -1.277796023 | 3.9382E-05 |
| MYO16-AS1 | 1.308339339 | 4.00371E-05 |
| KRTAP3-3 | -1.240823037 | 4.02894E-05 |
| AC087379.1 | 1.087585109 | 4.10691E-05 |
| AC009141.1 | 1.256848099 | 4.14943E-05 |
| MIR603 | 1.525643217 | 4.20029E-05 |
| AL355483.2 | 1.040003682 | 4.3371E-05 |
| LINC00668 | -1.180038978 | 4.4102E-05 |
| ALPP | 1.09581115 | 4.41839E-05 |
| PTH2 | -1.264777875 | 4.43228E-05 |
| AL355994.4 | 1.01377856 | 4.44276E-05 |
| MIR124-1HG | 1.256642819 | 4.5681E-05 |
| S100G | -1.13196108 | 4.61562E-05 |
| RPS20P9 | 1.206711634 | 4.67443E-05 |
| AC097063.1 | 1.338082445 | 4.81872E-05 |
| RBMS3-AS1 | 1.429062039 | 4.86576E-05 |
| FOXG1-AS1 | 1.701038971 | 4.89942E-05 |
| KARS1P1 | 1.242823675 | 4.91557E-05 |
| AC078905.1 | -1.630980226 | 4.93942E-05 |
| RNA5SP159 | 1.205258038 | 5.00221E-05 |
| AC090985.2 | 1.167820331 | 5.06253E-05 |
| LINC02199 | 1.354974328 | 5.10647E-05 |
| AC055733.2 | 1.863310977 | 5.11021E-05 |
| MIR548XHG | -1.718836273 | 5.14604E-05 |
| AC008534.1 | 1.175640691 | 5.21262E-05 |
| AL589826.2 | 1.113063202 | 5.32692E-05 |
| MIR2355 | 1.226293062 | 5.47902E-05 |
| FP236383.5 | -1.654673265 | 5.49639E-05 |
| MTND1P3 | 1.041680265 | 5.49798E-05 |
| AL591212.2 | 1.63320225 | 5.50441E-05 |
| AC023090.1 | 1.10508921 | 5.62132E-05 |
| EIF1AXP2 | 1.569404555 | 5.83606E-05 |
| RAD51AP1P1 | 1.618957537 | 5.83706E-05 |
| BRD7P3 | 1.092309789 | 5.90745E-05 |
| MIR4713HG | -1.012595994 | 5.92043E-05 |
| AC060234.3 | 1.195158973 | 5.98759E-05 |
| AC073544.1 | 1.289720855 | 6.02409E-05 |
| RN7SL173P | 1.455969512 | 6.1267E-05 |
| RNU6-1146P | 1.377476729 | 6.15241E-05 |
| FGF21 | 1.355846716 | 6.24119E-05 |
| AC090099.2 | 1.297619809 | 6.25845E-05 |
| OR2AG1 | 1.079133194 | 6.29307E-05 |
| RN7SL760P | 1.225121696 | 6.41817E-05 |
| RN7SL16P | 1.224910068 | 6.42746E-05 |
| AP002967.1 | 1.679637661 | 6.50763E-05 |
| AL139002.1 | 1.878891344 | 6.54832E-05 |
| RNU11-2P | 1.436387846 | 6.56318E-05 |
| ARL4AP1 | 1.189377634 | 6.68245E-05 |
| AC093292.1 | -1.368481168 | 6.77167E-05 |
| SLC9A3P2 | -1.326997565 | 6.90849E-05 |
| AC007736.1 | 1.628351924 | 6.94285E-05 |
| MIR938 | 1.620163825 | 7.1211E-05 |
| KCNV1 | 1.07765958 | 7.21531E-05 |
| BPIFB3 | 2.097950801 | 7.24353E-05 |
| VSTM2A-OT1 | -1.293154852 | 7.31892E-05 |
| FAM32CP | -1.186167788 | 7.47595E-05 |
| LINC02582 | 1.159883765 | 7.71612E-05 |
| HMBOX1-IT1 | 1.145790391 | 7.73663E-05 |
| LINC02003 | 1.047747217 | 7.84794E-05 |
| PHBP4 | 1.012923786 | 7.89877E-05 |
| AC016885.2 | -1.198533205 | 7.9413E-05 |
| MZT1P1 | 1.518123521 | 7.94211E-05 |
| LINC02616 | -1.734600571 | 8.04981E-05 |
| AL450990.1 | -1.654570331 | 8.10686E-05 |
| SSXP10 | 1.441879567 | 8.23801E-05 |
| AL606517.2 | 2.04119862 | 8.27582E-05 |
| HMGB1P7 | 1.220002583 | 8.33663E-05 |
| AC104781.1 | 1.413241056 | 8.41845E-05 |
| AL365434.1 | 1.025701424 | 8.47422E-05 |
| RNU4-40P | 1.166528181 | 8.78774E-05 |
| LINC02355 | 1.037840736 | 8.85359E-05 |
| GABRG3-AS1 | 1.630173831 | 8.97433E-05 |
| RNU6-1177P | 1.07549764 | 9.23097E-05 |
| LINC02233 | -1.034541799 | 9.35296E-05 |
| DDX53 | -1.524421369 | 9.49253E-05 |
| AC083875.1 | 1.990633943 | 9.56068E-05 |
| OR52E6 | -1.349485506 | 9.58672E-05 |
| COMETT | 1.090081201 | 9.6373E-05 |
| PCDH11Y | 1.085757276 | 9.82843E-05 |
| MIR604 | 1.033880398 | 9.95741E-05 |
| SRGAP3-AS3 | 1.29470693 | 0.000101166 |
| AC022022.1 | 1.363219871 | 0.000102167 |
| SNRPEP9 | 1.271696684 | 0.000102251 |
| AC007599.2 | 1.242727037 | 0.000103108 |
| AC078860.3 | 1.021563449 | 0.000104318 |
| ARPC1BP1 | 1.435317374 | 0.000104448 |
| RN7SL30P | 1.14903905 | 0.000105043 |
| AC093912.1 | 1.289015542 | 0.000106837 |
| MIR5699 | 1.407703685 | 0.000107378 |
| LINC01854 | 1.72872738 | 0.000111676 |
| MKX-AS1 | -1.117927262 | 0.000112027 |
| SOX1 | 1.258476009 | 0.000113059 |
| OR7E129P | 1.256761491 | 0.000113101 |
| CYP4F34P | 1.23748433 | 0.000115168 |
| AC010359.1 | 2.275371978 | 0.000118848 |
| AC023813.2 | 1.389064185 | 0.000118994 |
| MTND2P2 | 1.591172206 | 0.000121131 |
| AC093423.2 | 1.09551333 | 0.000121616 |
| PDX1 | 1.37758079 | 0.000122003 |
| GABRG2 | -1.175536198 | 0.00012375 |
| AL049548.1 | 1.876382363 | 0.000124765 |
| UGT1A7 | -1.650448369 | 0.000132869 |
| RHOXF1P2 | -1.190749218 | 0.000134065 |
| AC005160.1 | 2.056948976 | 0.000135237 |
| AC019270.1 | 1.164284066 | 0.000136588 |
| ECE2 | 1.243415522 | 0.000139002 |
| RN7SL736P | 1.00062853 | 0.000140289 |
| RNA5SP247 | 1.026043968 | 0.000143577 |
| AC019330.1 | 1.336108356 | 0.000143853 |
| THCAT155 | -1.392385989 | 0.000146043 |
| AC027335.1 | 1.809607295 | 0.000146413 |
| BACH1-IT3 | 1.418179481 | 0.000147043 |
| RNA5SP282 | 1.062397462 | 0.000147623 |
| LINC02077 | -2.339114336 | 0.000149276 |
| AL162511.1 | 1.260464314 | 0.000149276 |
| RNU7-181P | 1.045257435 | 0.000150257 |
| RBM22P4 | 1.460749229 | 0.000150869 |
| AL356413.1 | -1.303269508 | 0.000155087 |
| AL049777.1 | 1.502676657 | 0.000157033 |
| LINC02335 | -2.35411905 | 0.000157047 |
| USP12-AS1 | 1.581555363 | 0.000157185 |
| TANK-AS1 | 1.58176168 | 0.000157252 |
| NDUFA3P2 | 1.064845724 | 0.000158077 |
| VRTN | 1.00249868 | 0.000158091 |
| AL353613.1 | 1.42028297 | 0.000158792 |
| AC004986.1 | 1.270473287 | 0.000159099 |
| HAND1 | -1.075148182 | 0.00015964 |
| AC109597.1 | 1.25962963 | 0.000160622 |
| AL359693.1 | 1.086030602 | 0.000164248 |
| RNU6-433P | 1.803364922 | 0.000165453 |
| AP003173.1 | -2.228434776 | 0.000174131 |
| AC018730.1 | 1.17202693 | 0.000174303 |
| AL033504.1 | 1.447997481 | 0.000175158 |
| AC007431.2 | 1.026200732 | 0.000175746 |
| TPMTP2 | 1.035750415 | 0.000178813 |
| ELMO1-AS1 | 1.650157148 | 0.000180842 |
| AC073651.1 | 1.000280461 | 0.000182188 |
| OR5G5P | 1.169833483 | 0.000183294 |
| NPAP1 | 1.149081615 | 0.000188816 |
| CSP1 | 1.349148449 | 0.000190209 |
| AC060765.1 | -1.515263416 | 0.000190406 |
| AC104985.1 | 1.599117522 | 0.000196541 |
| AL161733.1 | -1.097902247 | 0.000198161 |
| AL731575.1 | 1.347271731 | 0.000201813 |
| LINC02682 | 1.237016415 | 0.000201821 |
| LINC00678 | 1.12002672 | 0.000202357 |
| AP002478.2 | 1.113251585 | 0.000203179 |
| LINC00355 | -1.234937349 | 0.000219447 |
| RN7SL57P | 1.318973791 | 0.0002287 |
| ZNF859P | 1.311852053 | 0.000232821 |
| AL357552.1 | 1.010270695 | 0.000232887 |
| LINC02282 | 2.229689789 | 0.000237469 |
| AC107057.1 | 1.748695172 | 0.000238013 |
| TPD52L3 | 1.354090969 | 0.000241322 |
| RPS29P2 | 1.153508764 | 0.000246415 |
| AC026461.3 | 1.091747118 | 0.000252612 |
| IGKV2D-26 | 1.026096887 | 0.000257474 |
| RNU5A-8P | 1.561389652 | 0.000258047 |
| METTL21AP1 | 1.032254374 | 0.000260531 |
| MYG1P1 | 1.305492069 | 0.00026176 |
| RN7SKP235 | 1.010257104 | 0.000267552 |
| RNU6-1327P | 1.406365734 | 0.00027055 |
| AC008533.1 | 1.123732895 | 0.000271492 |
| LINC02327 | -1.10949246 | 0.000273009 |
| PIMREGP4 | 1.486630719 | 0.000284461 |
| RNU6-1042P | 1.261514982 | 0.000294977 |
| LINC00261 | -1.443075793 | 0.000299041 |
| AC090692.1 | 1.265502546 | 0.000300875 |
| SPINT3 | -1.44416324 | 0.000303298 |
| AL161729.2 | 1.004558754 | 0.000303329 |
| AC023385.1 | 1.704687904 | 0.000303337 |
| RN7SL430P | 1.447031622 | 0.000308585 |
| AC024132.3 | 1.296554485 | 0.000311375 |
| TRIM51 | 1.492841769 | 0.000311651 |
| GAST | 1.112825183 | 0.000313465 |
| AL136360.2 | 1.019408945 | 0.000313539 |
| AL512326.5 | 1.231898628 | 0.000314361 |
| RN7SL125P | -1.198562193 | 0.000315297 |
| COX7BP2 | 1.058374045 | 0.000316035 |
| UCMA | 1.065381051 | 0.000322842 |
| ADAM2 | -1.207177987 | 0.000329988 |
| RPS12P5 | 1.413061325 | 0.000338179 |
| EIF2AP4 | 1.032880829 | 0.000342021 |
| ATP6V0E1P1 | 1.042186636 | 0.000346181 |
| IGKV2OR2-7D | 1.004803044 | 0.000352064 |
| AC092720.2 | -1.433331392 | 0.000373598 |
| ASB18 | 1.022222642 | 0.000377093 |
| AC093802.2 | 1.741259347 | 0.00037765 |
| AL357315.1 | 1.307347685 | 0.000381771 |
| AC138832.1 | 1.174960889 | 0.00038374 |
| U6 | 1.226743701 | 0.000385028 |
| Metazoa_SRP | 1.341286566 | 0.000386397 |
| AL136313.1 | 1.853061895 | 0.000388225 |
| Y_RNA | 1.602947813 | 0.000395112 |
| AC087821.2 | 2.134738892 | 0.000397811 |
| AP001102.1 | 1.24799685 | 0.000400853 |
| AC093909.4 | 1.136557404 | 0.000407419 |
| AP000462.1 | 1.25636579 | 0.000418949 |
| AL137818.1 | 1.297039442 | 0.000422581 |
| LCE1B | 1.560356251 | 0.000432631 |
| TRIM43 | 1.285978648 | 0.000433016 |
| ATG10-AS1 | 1.917860115 | 0.000434902 |
| AL513475.2 | 1.113904339 | 0.000439864 |
| VAX1 | 1.089081169 | 0.000440955 |
| TMEM108-AS1 | 1.065027195 | 0.000441012 |
| TMEM271 | 1.109376946 | 0.000466043 |
| AC144521.1 | 1.546722009 | 0.000469314 |
| RN7SKP245 | 1.150924839 | 0.00047088 |
| AC018558.6 | -1.678467622 | 0.000472419 |
| AL359894.1 | -1.514342569 | 0.000477421 |
| RN7SL8P | 1.242371215 | 0.000483829 |
| MTND1P32 | 1.219433895 | 0.000487118 |
| AC136603.1 | 1.456919452 | 0.000512121 |
| AC067960.1 | 1.774578256 | 0.000512271 |
| AC132807.2 | -1.074581272 | 0.000512488 |
| RN7SL330P | 1.273829244 | 0.000515046 |
| AC022239.2 | 1.194027487 | 0.000515473 |
| RNU6-617P | -1.008878498 | 0.000518688 |
| AL031428.1 | 1.110533843 | 0.00052 |
| AC024022.1 | 1.318902329 | 0.000525007 |
| OR51I2 | 1.299279127 | 0.00052518 |
| LINC02136 | 1.10879667 | 0.000535035 |
| MIR5690 | 1.011311341 | 0.00053842 |
| USP12P2 | 1.105505295 | 0.000538532 |
| AL139023.1 | -1.230133884 | 0.000546923 |
| AC012354.9 | 1.000986151 | 0.00055377 |
| AC069061.2 | -1.863042036 | 0.000559774 |
| LINC02122 | -1.159073911 | 0.00057066 |
| UCN3 | -1.192067637 | 0.000575534 |
| AC106818.1 | -1.295903295 | 0.000591694 |
| AC106745.1 | 1.093930873 | 0.000604515 |
| AL355538.1 | 1.071296093 | 0.000614593 |
| AC079779.1 | 1.288673598 | 0.000644417 |
| AC009142.1 | 1.373005628 | 0.000645834 |
| AC021242.2 | 1.459885538 | 0.000645943 |
| AC005972.2 | 1.391389994 | 0.000650293 |
| AGBL4-IT1 | 1.0383379 | 0.000665433 |
| AC013549.3 | 1.205275681 | 0.000666113 |
| RNA5SP53 | 1.090024626 | 0.000668769 |
| AC008825.1 | -1.735457664 | 0.000670503 |
| AC036111.1 | 1.098575822 | 0.000672767 |
| AL008720.1 | -1.16606027 | 0.000675145 |
| SLC17A6 | 1.588305411 | 0.000680124 |
| YBX1P3 | 1.241660284 | 0.000680702 |
| LINC02726 | -1.292890956 | 0.000692263 |
| AC018558.5 | -1.312245985 | 0.000693296 |
| AP006219.3 | 1.103115771 | 0.000693374 |
| AC106785.2 | 1.478429588 | 0.000704112 |
| MROH4P | 1.180493129 | 0.000714603 |
| PRB1 | 1.592500831 | 0.000722913 |
| AC079200.1 | 1.230218096 | 0.000731331 |
| AL159987.1 | 1.868002149 | 0.000735349 |
| NKX2-1-AS1 | 1.841636393 | 0.000751911 |
| AC006262.4 | 1.093422769 | 0.000796749 |
| OR56A3 | -1.054970789 | 0.000798895 |
| SNORA74C-2 | 1.612179166 | 0.000801715 |
| RNU6-946P | 1.807549362 | 0.000820706 |
| EDDM3CP | 2.38873441 | 0.000836416 |
| TEX37 | 1.006629262 | 0.000842907 |
| AC011676.2 | 1.064606811 | 0.000850665 |
| AL158817.1 | 1.137321093 | 0.000854424 |
| AC026427.2 | 1.338056385 | 0.000857144 |
| RNU6-268P | 1.445203678 | 0.000904781 |
| AC016902.1 | 1.137075689 | 0.000907944 |
| AL359095.1 | 1.789254263 | 0.000920606 |
| AL390754.1 | 1.214509746 | 0.000925667 |
| AC058823.1 | 1.012848634 | 0.000938097 |
| CHAT | 1.156561394 | 0.001004985 |
| BX284613.1 | 1.426182051 | 0.001043045 |
| OTX2 | 1.162558259 | 0.00104863 |
| AC026474.1 | 1.015863056 | 0.00105107 |
| RN7SKP56 | 1.724351246 | 0.001061265 |
| RPS13P4 | 1.185980929 | 0.001070649 |
| AL137001.2 | -1.650858264 | 0.001095166 |
| RPL13AP17 | -1.060317804 | 0.001104722 |
| AL138880.1 | 1.483170713 | 0.001126065 |
| NKX2-1 | 1.352241438 | 0.001129892 |
| SNRPCP4 | 1.246027216 | 0.001129975 |
| AC078860.1 | 1.246282121 | 0.001135239 |
| AC025211.1 | 1.035687648 | 0.001137556 |
| TGIF2LX | -1.753127965 | 0.00116143 |
| HSPA8P13 | 1.791229848 | 0.001170943 |
| OR13D1 | 1.166499486 | 0.00118733 |
| LINC01980 | -1.031869014 | 0.001196904 |
| AL512430.1 | 1.00128108 | 0.001202797 |
| AC100778.4 | 1.565377513 | 0.00120766 |
| AC122714.1 | 1.385396689 | 0.001276165 |
| LINC01079 | -1.156346989 | 0.001300876 |
| BCAS2P1 | 1.41189381 | 0.001333546 |
| SLURP2 | 1.00620445 | 0.001371704 |
| AC107890.1 | -1.255523666 | 0.001384067 |
| RNU2-59P | 1.739858776 | 0.001397588 |
| AC012574.2 | 1.074868655 | 0.001404806 |
| AL355112.1 | 1.220538604 | 0.001433571 |
| AC099654.5 | 1.654836136 | 0.001436816 |
| AC025839.1 | 1.161264498 | 0.001446103 |
| HNRNPA1P29 | 1.496766651 | 0.001452633 |
| AC010677.2 | 1.391147145 | 0.001484433 |
| VSTM2B | 1.147559646 | 0.001509034 |
| RNA5SP107 | -1.805147922 | 0.00153351 |
| RN7SKP158 | 1.231480469 | 0.001604534 |
| C4BPAP1 | 1.027763932 | 0.001624902 |
| AC022778.1 | 1.385519328 | 0.001668034 |
| RN7SL660P | -1.630139572 | 0.001724318 |
| MTCO3P15 | 1.305508759 | 0.001728455 |
| LINC00400 | -1.263560051 | 0.001740301 |
| AF130417.1 | 1.277698312 | 0.001759698 |
| AC004936.1 | 1.771097748 | 0.001779821 |
| AC096861.1 | 1.120059282 | 0.001785977 |
| OR13Z2P | -1.029031704 | 0.001826194 |
| GNAT3 | -1.357538135 | 0.001831268 |
| RNU4-80P | 1.079305959 | 0.001836629 |
| AP003062.2 | 1.147697175 | 0.001865755 |
| AP000289.1 | 1.025195034 | 0.001874171 |
| RNY4P7 | 1.197287476 | 0.001932973 |
| AL035090.1 | 1.126716466 | 0.001934286 |
| AC009108.4 | 1.019927734 | 0.001985576 |
| BLID | 1.442971518 | 0.001989176 |
| LINC00709 | -2.118600219 | 0.002011256 |
| PPP1R2P2 | 1.140305372 | 0.002068166 |
| AC012500.1 | 1.774696008 | 0.002110812 |
| RNU6-848P | -1.546559968 | 0.002145795 |
| AC023271.1 | 1.015168348 | 0.002147823 |
| AL118558.2 | 1.038246287 | 0.002305381 |
| AC106895.1 | 1.25200277 | 0.002311026 |
| LINC00383 | -1.073223224 | 0.002324903 |
| AL050403.1 | 1.084250669 | 0.002328115 |
| AC100763.1 | 1.223219686 | 0.002332729 |
| AC016994.1 | 1.288896537 | 0.002389091 |
| SSX3 | -1.531663378 | 0.002401655 |
| KRTAP10-4 | -1.116967333 | 0.002432029 |
| AC105393.2 | 1.087249052 | 0.002472357 |
| AC073968.2 | -1.003987049 | 0.00249894 |
| POU3F4 | 1.310958824 | 0.00250424 |
| AC025575.2 | -1.044733268 | 0.002555104 |
| AC076966.1 | 1.110488401 | 0.002936869 |
| AC021134.2 | -1.062696622 | 0.002939575 |
| AC090116.1 | 1.055854597 | 0.002940922 |
| DCAF8L2 | -1.118076309 | 0.00300595 |
| ZNF679 | 1.750229625 | 0.0030366 |
| Y_RNA | 1.821626153 | 0.003108118 |
| AL139327.1 | -1.443871849 | 0.003128344 |
| RNU6-1045P | 1.172803998 | 0.003198393 |
| H3P33 | 1.023266701 | 0.0032653 |
| LINC01370 | -1.473942711 | 0.003332507 |
| AC016587.1 | 1.128148882 | 0.00338898 |
| RNU1-83P | 1.120225119 | 0.003412368 |
| AC007846.1 | 1.111176089 | 0.003428093 |
| GNPATP | -1.052650914 | 0.003439558 |
| TRGVB | -1.076068279 | 0.003567787 |
| RNY3 | 2.553057435 | 0.003603697 |
| AP005057.1 | -1.093332491 | 0.003620528 |
| LY6L | -1.481478678 | 0.003628415 |
| AC006504.4 | 1.199282965 | 0.003669135 |
| RPL31P20 | 1.307006022 | 0.003826014 |
| AP000844.1 | 1.063244566 | 0.003828049 |
| MIR2052 | -1.137819134 | 0.003875114 |
| AC073591.1 | 1.451624635 | 0.003901319 |
| DIAPH3-AS2 | 1.356023752 | 0.003909395 |
| SERPINB11 | 1.423280808 | 0.003959058 |
| MAGEB18 | -1.221573389 | 0.004049826 |
| AC023158.1 | -1.067045654 | 0.004260538 |
| LINC01953 | -1.402263495 | 0.004335789 |
| OR7A19P | 1.184852613 | 0.00437408 |
| OTOP1 | 1.278015761 | 0.004423527 |
| MMP26 | -1.236729986 | 0.004471891 |
| TLX3 | 1.177851763 | 0.004481278 |
| UGT2B24P | -1.01419323 | 0.004591321 |
| LCE2A | 2.015398245 | 0.00463896 |
| AC097639.2 | 1.341399068 | 0.004710599 |
| AL356094.2 | 1.009538229 | 0.004831495 |
| AC016027.3 | 1.120322491 | 0.004937897 |
| LINC01014 | 1.019038262 | 0.004969577 |
| OR4K8P | -1.219141452 | 0.005055607 |
| AC092608.2 | 2.291366324 | 0.005065795 |
| FRG2DP | -1.281585358 | 0.005161745 |
| AC006270.2 | 1.095579946 | 0.005210972 |
| LINC01456 | -1.140647674 | 0.005232983 |
| AC113398.2 | 1.72073717 | 0.005273421 |
| CT83 | 1.359438776 | 0.00531247 |
| CRCT1 | 1.120105879 | 0.005447864 |
| AP003115.1 | 1.008384 | 0.005494717 |
| AC023158.2 | -1.087644821 | 0.005593771 |
| RNA5SP20 | 1.346603736 | 0.005598183 |
| LINC02167 | 1.128914657 | 0.005845875 |
| ZNF722P | -1.383951507 | 0.006015419 |
| AC022537.1 | 1.942593622 | 0.006109106 |
| LINC02643 | 1.067536035 | 0.006357352 |
| GJD2 | 1.316590551 | 0.006608776 |
| AC013562.1 | 1.252001965 | 0.006610959 |
| AL513487.1 | 1.136398855 | 0.006677907 |
| MAGEB16 | -1.229639566 | 0.006712738 |
| HSPE1P28 | 1.45963035 | 0.006924581 |
| AC095030.1 | -1.026813661 | 0.007154487 |
| GTF3AP6 | -1.173621566 | 0.00740284 |
| RN7SL555P | -1.301513834 | 0.00768517 |
| DBX1 | 1.04457434 | 0.007765508 |
| DEFB126 | -1.20354041 | 0.007817285 |
| AL360169.1 | 1.084604999 | 0.00794005 |
| DANT1 | 1.257619029 | 0.007952651 |
| RN7SL517P | 1.056244461 | 0.008142583 |
| PGAM4P2 | -1.665295476 | 0.008322129 |
| Z83818.2 | 1.194759703 | 0.00842636 |
| AC100850.1 | 1.029147178 | 0.008550101 |
| AC107023.1 | -1.15744292 | 0.008576285 |
| AC016292.1 | 1.18110118 | 0.008643707 |
| AC140481.1 | -1.092494887 | 0.008841463 |
| SLCO1B3 | 1.012140864 | 0.008964162 |
| AC009153.1 | 1.026526901 | 0.009114491 |
| AL137161.1 | 1.069753579 | 0.009157274 |
| RNU1-11P | -1.358022656 | 0.009169653 |
| MAGEA3 | -1.172501563 | 0.009953753 |
| LINC02835 | -1.233131326 | 0.010098445 |
| RPL37P4 | -1.293062108 | 0.010228883 |
| MAGEA4 | 1.03440019 | 0.010352633 |
| MIR181A2 | 1.00149823 | 0.010418225 |
| MRPS10P2 | 1.003298313 | 0.010768014 |
| AF241725.1 | -2.720394398 | 0.011072272 |
| AL122126.1 | -1.482950691 | 0.011139953 |
| RNA5SP174 | 1.023973758 | 0.011232274 |
| AC103853.1 | 1.170019222 | 0.011380748 |
| IL36A | -1.059569741 | 0.011815468 |
| LINC01153 | 1.370283094 | 0.011915657 |
| AC010789.1 | 1.002484729 | 0.011940418 |
| AC093791.1 | -1.055871606 | 0.012292474 |
| H3Y2 | 1.073000076 | 0.012308877 |
| LINC01419 | -1.307773157 | 0.012599961 |
| RNU6-681P | 1.178277302 | 0.012950779 |
| UGT1A6 | -1.009068274 | 0.013022674 |
| AL590681.1 | 1.086131344 | 0.013084724 |
| LINC02294 | -1.098983391 | 0.013125562 |
| SPRR2A | -1.17709756 | 0.013256939 |
| RNU6-1188P | 1.153496395 | 0.013672994 |
| LINC02377 | -1.265469699 | 0.013926286 |
| Z97205.3 | 1.027174683 | 0.014065479 |
| AC022960.3 | 1.109095519 | 0.014071514 |
| VENTXP1 | 1.398916376 | 0.014471506 |
| AL592043.1 | -1.278320548 | 0.014862307 |
| AC108676.2 | 1.151974595 | 0.015293416 |
| AC109635.5 | 1.095518368 | 0.015520923 |
| AC093414.1 | 1.119363331 | 0.015613012 |
| ARHGAP22-IT1 | 1.029104246 | 0.015966933 |
| Z82202.2 | -1.568308957 | 0.016161131 |
| PPDPFL | 1.107084256 | 0.017290786 |
| MAGEA4-AS1 | 1.375266687 | 0.017406238 |
| PIGFP2 | 1.438134235 | 0.01857325 |
| RARRES2P10 | -1.550239721 | 0.018692672 |
| AL356313.1 | -1.459208559 | 0.018713992 |
| AL160159.1 | 2.029700499 | 0.019109255 |
| GCG | 1.018224626 | 0.019373629 |
| LINC01194 | -1.242833671 | 0.020061836 |
| LCE1A | 2.696038039 | 0.020555302 |
| LINC02378 | -1.068869622 | 0.020573649 |
| MAGEA9B | 1.355149636 | 0.020668182 |
| CU104787.1 | -1.382932532 | 0.020726751 |
| KLHL1 | -1.062941982 | 0.020764042 |
| RN7SL308P | 1.249839895 | 0.021472997 |
| AC105224.1 | 1.384008281 | 0.021870725 |
| AL731574.1 | 1.411834649 | 0.021880794 |
| RN7SL398P | 1.287507872 | 0.022067297 |
| MIR1299 | 1.011089069 | 0.022789191 |
| NEUROD4 | -1.312992484 | 0.022871769 |
| NFYAP1 | -1.010344407 | 0.023012776 |
| RN7SL197P | -1.442457736 | 0.02318182 |
| SPRR4 | 1.40990645 | 0.024457667 |
| AL389889.2 | 1.074850033 | 0.025097 |
| ADAD1P2 | 1.482468357 | 0.026294836 |
| AC097521.1 | 1.045434182 | 0.026434755 |
| AC069079.1 | 1.08104905 | 0.026635404 |
| MAGEA6 | -1.042925776 | 0.027045979 |
| ZNF317P1 | 1.191448314 | 0.02736482 |
| AC093787.1 | 1.033375815 | 0.027918231 |
| AC008592.2 | 1.500545027 | 0.027978056 |
| MARK2P12 | 1.213492093 | 0.028108894 |
| AC022081.1 | -1.107648883 | 0.028950636 |
| UGT1A8 | -1.041380244 | 0.029265095 |
| AC104793.1 | -1.491486176 | 0.029394464 |
| HTN1 | 1.09481598 | 0.029673603 |
| RN7SKP79 | 1.592903747 | 0.029733803 |
| LINC01202 | -1.140563596 | 0.03033941 |
| AL590138.1 | 1.009751469 | 0.031003969 |
| AL512326.2 | 1.091154526 | 0.031539114 |
| AC090506.1 | 1.30876476 | 0.031665029 |
| AC008833.1 | 1.365800354 | 0.03176547 |
| AL359987.1 | -1.183730321 | 0.032003454 |
| AC110790.1 | -1.323568291 | 0.033445803 |
| AL358333.2 | 1.115455988 | 0.034380916 |
| LINC02031 | -1.2526885 | 0.03440214 |
| AC011287.2 | 1.220684593 | 0.034915728 |
| AC079772.1 | 1.159379806 | 0.037593173 |
| AC107385.2 | -1.924380989 | 0.038143068 |
| AL035250.2 | 1.09486447 | 0.038555584 |
| USP24P1 | -1.378484239 | 0.040060054 |
| PLCB1-IT1 | 1.243938682 | 0.040915614 |
| AL929601.1 | -1.15675974 | 0.044605227 |
| EIF4E2P1 | 1.009784263 | 0.04529148 |
| GAGE1 | 1.294740289 | 0.045440813 |
| BX276092.5 | -1.582391818 | 0.046740027 |
| AC018558.2 | -1.418260692 | 0.047278092 |
| AL354685.1 | -1.571525611 | 0.049433972 |
