# Supplementary material for: Identification and validation of DNA methylation-driven gene OSR1 as a novel tumor suppressor for the diagnosis and prognosis of breast cancer
Source: Front Genet. 2025 Jul 7;16:1583620. doi: 10.3389/fgene.2025.1583620 (PMC12277919; doi:10.3389/fgene.2025.1583620)
Supplement: Supplementary file 3 [file Table5.docx]

**Supplementary Table 3**. Gene Ontology pathway functional enrichment for *0SR1*-related DEGs

| ONTOLOGY | ID | Description | GeneRatio | BgRatio | pvalue | p.adjust | qvalue | geneID | Count | zscore |
| --- | --- | --- | --- | --- | --- | --- | --- | --- | --- | --- |
| BP | GO:0010817 | regulation of hormone levels | 16/169 | 496/18800 | 1.08018E-05 | 0.017109047 | 0.014489875 | SOX8/OPRK1/CHGA/SFRP1/RPE65/EDN3/CGA/NEUROD1/CARTPT/SLC30A8/KLK6/FGG/FGB/LEP/PCSK1/ADH4 | 16 | 0 |
| BP | GO:0021700 | developmental maturation | 12/169 | 296/18800 | 1.56662E-05 | 0.017109047 | 0.014489875 | SOX8/SEZ6/ROPN1/ACTL6B/SOX10/ROPN1B/ASCL1/CDK5R2/FGG/LEP/RELN/RHEX | 12 | 0.577350269 |
| BP | GO:0072210 | metanephric nephron development | 5/169 | 40/18800 | 2.82204E-05 | 0.017109047 | 0.014489875 | SOX8/OSR1/IRX1/AGTR2/LHX1 | 5 | 1.341640786 |
| BP | GO:0046883 | regulation of hormone secretion | 10/169 | 231/18800 | 4.69536E-05 | 0.017109047 | 0.014489875 | OPRK1/CHGA/SFRP1/EDN3/NEUROD1/CARTPT/SLC30A8/FGG/FGB/LEP | 10 | -0.632455532 |
| BP | GO:0046879 | hormone secretion | 11/169 | 281/18800 | 4.90345E-05 | 0.017109047 | 0.014489875 | OPRK1/CHGA/SFRP1/EDN3/CGA/NEUROD1/CARTPT/SLC30A8/FGG/FGB/LEP | 11 | -0.904534034 |
| BP | GO:0023061 | signal release | 14/169 | 451/18800 | 5.93391E-05 | 0.017109047 | 0.014489875 | OPRK1/CHGA/SFRP1/EDN3/SYT4/CGA/CPLX2/SYT8/NEUROD1/CARTPT/SLC30A8/FGG/FGB/LEP | 14 | -1.069044968 |
| BP | GO:0009914 | hormone transport | 11/169 | 290/18800 | 6.51174E-05 | 0.017109047 | 0.014489875 | OPRK1/CHGA/SFRP1/EDN3/CGA/NEUROD1/CARTPT/SLC30A8/FGG/FGB/LEP | 11 | -0.904534034 |
| BP | GO:0071695 | anatomical structure maturation | 10/169 | 246/18800 | 7.94689E-05 | 0.017109047 | 0.014489875 | SOX8/ROPN1/ACTL6B/SOX10/ROPN1B/ASCL1/CDK5R2/FGG/LEP/RHEX | 10 | 0.632455532 |
| BP | GO:0032275 | luteinizing hormone secretion | 3/169 | 10/18800 | 8.17545E-05 | 0.017109047 | 0.014489875 | OPRK1/CGA/LEP | 3 | 0.577350269 |
| BP | GO:0051047 | positive regulation of secretion | 11/169 | 300/18800 | 8.81069E-05 | 0.017109047 | 0.014489875 | OPRK1/EDN3/SYT4/LACRT/CARTPT/SLC30A8/CDK5R2/FGG/FGB/LEP/PCSK1 | 11 | -1.507556723 |
| BP | GO:0050708 | regulation of protein secretion | 10/169 | 252/18800 | 9.70061E-05 | 0.017109047 | 0.014489875 | CHGA/SFRP1/SYT4/NEUROD1/CARTPT/SLC30A8/FGG/FGB/LEP/PCSK1 | 10 | -1.897366596 |
| BP | GO:0031424 | keratinization | 6/169 | 85/18800 | 0.000117419 | 0.017109047 | 0.014489875 | KLK5/KRT1/KRT79/LCE1A/LCE2A/LORICRIN | 6 | 2.449489743 |
| BP | GO:0001764 | neuron migration | 8/169 | 164/18800 | 0.000120136 | 0.017109047 | 0.014489875 | ASCL1/GFRA3/FEZF2/SOX14/CDK5R2/FOXG1/RELN/LHX1 | 8 | 0.707106781 |
| BP | GO:0050433 | regulation of catecholamine secretion | 5/169 | 56/18800 | 0.000145892 | 0.017109047 | 0.014489875 | OPRK1/CHGA/SYT4/SYT8/CARTPT | 5 | -0.447213595 |
| BP | GO:0001656 | metanephros development | 6/169 | 89/18800 | 0.000151469 | 0.017109047 | 0.014489875 | SOX8/OSR1/IRX1/AGTR2/POU3F3/LHX1 | 6 | 1.632993162 |
| BP | GO:0050432 | catecholamine secretion | 5/169 | 57/18800 | 0.000158768 | 0.017109047 | 0.014489875 | OPRK1/CHGA/SYT4/SYT8/CARTPT | 5 | -0.447213595 |
| BP | GO:0072080 | nephron tubule development | 6/169 | 91/18800 | 0.000171196 | 0.017109047 | 0.014489875 | SOX8/OSR1/IRX1/AGTR2/POU3F3/LHX1 | 6 | 1.632993162 |
| BP | GO:0022029 | telencephalon cell migration | 5/169 | 58/18800 | 0.000172494 | 0.017109047 | 0.014489875 | FEZF2/CDK5R2/FOXG1/RELN/POU3F3 | 5 | 1.341640786 |
| BP | GO:0030072 | peptide hormone secretion | 9/169 | 220/18800 | 0.000173058 | 0.017109047 | 0.014489875 | CHGA/SFRP1/EDN3/NEUROD1/CARTPT/SLC30A8/FGG/FGB/LEP | 9 | -1 |
| BP | GO:0048565 | digestive tract development | 7/169 | 130/18800 | 0.000175105 | 0.017109047 | 0.014489875 | OTC/ALX4/SOX10/SFRP1/CCKBR/SFRP5/ASCL1 | 7 | 1.889822365 |
| BP | GO:0051952 | regulation of amine transport | 6/169 | 92/18800 | 0.000181792 | 0.017109047 | 0.014489875 | OPRK1/CHGA/SYT4/SYT8/CARTPT/LEP | 6 | 0 |
| BP | GO:0090183 | regulation of kidney development | 4/169 | 32/18800 | 0.000186184 | 0.017109047 | 0.014489875 | SOX8/OSR1/AGTR2/LHX1 | 4 | 1 |
| BP | GO:1903532 | positive regulation of secretion by cell | 10/169 | 274/18800 | 0.00019205 | 0.017109047 | 0.014489875 | OPRK1/EDN3/SYT4/CARTPT/SLC30A8/CDK5R2/FGG/FGB/LEP/PCSK1 | 10 | -1.264911064 |
| BP | GO:0046888 | negative regulation of hormone secretion | 5/169 | 60/18800 | 0.000202647 | 0.017109047 | 0.014489875 | OPRK1/CHGA/SFRP1/CARTPT/LEP | 5 | 0.447213595 |
| BP | GO:0090276 | regulation of peptide hormone secretion | 8/169 | 177/18800 | 0.00020304 | 0.017109047 | 0.014489875 | CHGA/SFRP1/NEUROD1/CARTPT/SLC30A8/FGG/FGB/LEP | 8 | -1.414213562 |
| BP | GO:0061326 | renal tubule development | 6/169 | 94/18800 | 0.000204537 | 0.017109047 | 0.014489875 | SOX8/OSR1/IRX1/AGTR2/POU3F3/LHX1 | 6 | 1.632993162 |
| BP | GO:0002790 | peptide secretion | 9/169 | 225/18800 | 0.00020469 | 0.017109047 | 0.014489875 | CHGA/SFRP1/EDN3/NEUROD1/CARTPT/SLC30A8/FGG/FGB/LEP | 9 | -1 |
| BP | GO:0021799 | cerebral cortex radially oriented cell migration | 4/169 | 33/18800 | 0.000210388 | 0.017109047 | 0.014489875 | CDK5R2/FOXG1/RELN/POU3F3 | 4 | 1 |
| BP | GO:0021885 | forebrain cell migration | 5/169 | 61/18800 | 0.000219151 | 0.017207142 | 0.014572953 | FEZF2/CDK5R2/FOXG1/RELN/POU3F3 | 5 | 1.341640786 |
| BP | GO:0002791 | regulation of peptide secretion | 8/169 | 180/18800 | 0.000227666 | 0.017279823 | 0.014634508 | CHGA/SFRP1/NEUROD1/CARTPT/SLC30A8/FGG/FGB/LEP | 8 | -1.414213562 |
| BP | GO:0048469 | cell maturation | 8/169 | 183/18800 | 0.000254689 | 0.017612951 | 0.014916638 | SOX8/ROPN1/ACTL6B/SOX10/ROPN1B/ASCL1/FGG/RHEX | 8 | 0.707106781 |
| BP | GO:0090087 | regulation of peptide transport | 8/169 | 183/18800 | 0.000254689 | 0.017612951 | 0.014916638 | CHGA/SFRP1/NEUROD1/CARTPT/SLC30A8/FGG/FGB/LEP | 8 | -1.414213562 |
| BP | GO:0019233 | sensory perception of pain | 6/169 | 99/18800 | 0.000271287 | 0.017612951 | 0.014916638 | TAC1/OPRK1/PHF24/OPRPN/SMR3B/PENK | 6 | 2.449489743 |
| BP | GO:0019229 | regulation of vasoconstriction | 5/169 | 64/18800 | 0.000274845 | 0.017612951 | 0.014916638 | EDN3/CHRM3/FGG/FGB/LEP | 5 | 0.447213595 |
| BP | GO:0072073 | kidney epithelium development | 7/169 | 140/18800 | 0.000276217 | 0.017612951 | 0.014916638 | SOX8/SFRP1/OSR1/IRX1/AGTR2/POU3F3/LHX1 | 7 | 1.889822365 |
| BP | GO:0042886 | amide transport | 10/169 | 287/18800 | 0.000278466 | 0.017612951 | 0.014916638 | CHGA/SFRP1/EDN3/NEUROD1/CARTPT/SLC30A8/FGG/FGB/LEP/ABCA13 | 10 | -0.632455532 |
| BP | GO:0015837 | amine transport | 6/169 | 100/18800 | 0.000286482 | 0.017630259 | 0.014931297 | OPRK1/CHGA/SYT4/SYT8/CARTPT/LEP | 6 | 0 |
| BP | GO:0055123 | digestive system development | 7/169 | 142/18800 | 0.000301175 | 0.0180467 | 0.015283986 | OTC/ALX4/SOX10/SFRP1/CCKBR/SFRP5/ASCL1 | 7 | 1.889822365 |
| BP | GO:0032274 | gonadotropin secretion | 3/169 | 16/18800 | 0.000366664 | 0.020363287 | 0.017245934 | OPRK1/CGA/LEP | 3 | 0.577350269 |
| BP | GO:0051238 | sequestering of metal ion | 3/169 | 16/18800 | 0.000366664 | 0.020363287 | 0.017245934 | FTHL17/SLC30A8/FTMT | 3 | 0.577350269 |
| BP | GO:0072224 | metanephric glomerulus development | 3/169 | 16/18800 | 0.000366664 | 0.020363287 | 0.017245934 | OSR1/AGTR2/LHX1 | 3 | 0.577350269 |
| BP | GO:0008544 | epidermis development | 11/169 | 355/18800 | 0.00037842 | 0.020515773 | 0.017375076 | ALX4/NGFR/COL17A1/EDAR/KLK5/KRT1/KRT79/LCE1A/KRT14/LCE2A/LORICRIN | 11 | 3.31662479 |
| BP | GO:0090278 | negative regulation of peptide hormone secretion | 4/169 | 39/18800 | 0.00040552 | 0.021345937 | 0.018078153 | CHGA/SFRP1/CARTPT/LEP | 4 | 0 |
| BP | GO:0022037 | metencephalon development | 6/169 | 107/18800 | 0.000412482 | 0.021345937 | 0.018078153 | SEZ6/ASCL1/NEUROD1/CDK5R2/GDF10/LHX1 | 6 | -1.632993162 |
| BP | GO:0015833 | peptide transport | 9/169 | 249/18800 | 0.00043124 | 0.021439048 | 0.01815701 | CHGA/SFRP1/EDN3/NEUROD1/CARTPT/SLC30A8/FGG/FGB/LEP | 9 | -1 |
| BP | GO:0007218 | neuropeptide signaling pathway | 6/169 | 108/18800 | 0.000433539 | 0.021439048 | 0.01815701 | TAC1/OPRK1/NTS/CARTPT/PENK/NPY2R | 6 | 0.816496581 |
| BP | GO:0002792 | negative regulation of peptide secretion | 4/169 | 40/18800 | 0.000447442 | 0.021439048 | 0.01815701 | CHGA/SFRP1/CARTPT/LEP | 4 | 0 |
| BP | GO:0030198 | extracellular matrix organization | 10/169 | 307/18800 | 0.000473546 | 0.021439048 | 0.01815701 | COL17A1/COL9A3/COL9A1/MMP20/LOXL4/PTX3/KLK5/ANGPTL7/COL11A2/C6orf15 | 10 | 3.16227766 |
| BP | GO:0051937 | catecholamine transport | 5/169 | 72/18800 | 0.000476062 | 0.021439048 | 0.01815701 | OPRK1/CHGA/SYT4/SYT8/CARTPT | 5 | -0.447213595 |
| BP | GO:0072078 | nephron tubule morphogenesis | 5/169 | 72/18800 | 0.000476062 | 0.021439048 | 0.01815701 | SOX8/OSR1/IRX1/AGTR2/LHX1 | 5 | 1.341640786 |
| BP | GO:0043062 | extracellular structure organization | 10/169 | 308/18800 | 0.000485706 | 0.021439048 | 0.01815701 | COL17A1/COL9A3/COL9A1/MMP20/LOXL4/PTX3/KLK5/ANGPTL7/COL11A2/C6orf15 | 10 | 3.16227766 |
| BP | GO:0072009 | nephron epithelium development | 6/169 | 111/18800 | 0.000501779 | 0.021439048 | 0.01815701 | SOX8/OSR1/IRX1/AGTR2/POU3F3/LHX1 | 6 | 1.632993162 |
| BP | GO:0045229 | external encapsulating structure organization | 10/169 | 310/18800 | 0.000510804 | 0.021439048 | 0.01815701 | COL17A1/COL9A3/COL9A1/MMP20/LOXL4/PTX3/KLK5/ANGPTL7/COL11A2/C6orf15 | 10 | 3.16227766 |
| BP | GO:0072189 | ureter development | 3/169 | 18/18800 | 0.000527267 | 0.021439048 | 0.01815701 | SOX8/OSR1/LHX1 | 3 | 0.577350269 |
| BP | GO:0072234 | metanephric nephron tubule development | 3/169 | 18/18800 | 0.000527267 | 0.021439048 | 0.01815701 | SOX8/OSR1/POU3F3 | 3 | 1.732050808 |
| BP | GO:0090190 | positive regulation of branching involved in ureteric bud morphogenesis | 3/169 | 18/18800 | 0.000527267 | 0.021439048 | 0.01815701 | SOX8/AGTR2/LHX1 | 3 | 0.577350269 |
| BP | GO:0072088 | nephron epithelium morphogenesis | 5/169 | 74/18800 | 0.000540252 | 0.021581636 | 0.018277769 | SOX8/OSR1/IRX1/AGTR2/LHX1 | 5 | 1.341640786 |
| BP | GO:0061333 | renal tubule morphogenesis | 5/169 | 76/18800 | 0.000610736 | 0.023605191 | 0.019991544 | SOX8/OSR1/IRX1/AGTR2/LHX1 | 5 | 1.341640786 |
| BP | GO:0072079 | nephron tubule formation | 3/169 | 19/18800 | 0.000622008 | 0.023605191 | 0.019991544 | SOX8/OSR1/IRX1 | 3 | 1.732050808 |
| BP | GO:0072087 | renal vesicle development | 3/169 | 19/18800 | 0.000622008 | 0.023605191 | 0.019991544 | SOX8/OSR1/LHX1 | 3 | 0.577350269 |
| BP | GO:0021795 | cerebral cortex cell migration | 4/169 | 44/18800 | 0.000646335 | 0.023815162 | 0.020169372 | CDK5R2/FOXG1/RELN/POU3F3 | 4 | 1 |
| BP | GO:0072028 | nephron morphogenesis | 5/169 | 77/18800 | 0.000648459 | 0.023815162 | 0.020169372 | SOX8/OSR1/IRX1/AGTR2/LHX1 | 5 | 1.341640786 |
| BP | GO:0072170 | metanephric tubule development | 3/169 | 20/18800 | 0.000726958 | 0.026274357 | 0.022252096 | SOX8/OSR1/POU3F3 | 3 | 1.732050808 |
| BP | GO:0072243 | metanephric nephron epithelium development | 3/169 | 21/18800 | 0.00084254 | 0.029357683 | 0.024863405 | SOX8/OSR1/POU3F3 | 3 | 1.732050808 |
| BP | GO:0090189 | regulation of branching involved in ureteric bud morphogenesis | 3/169 | 21/18800 | 0.00084254 | 0.029357683 | 0.024863405 | SOX8/AGTR2/LHX1 | 3 | 0.577350269 |
| BP | GO:0021543 | pallium development | 7/169 | 169/18800 | 0.000852993 | 0.029357683 | 0.024863405 | ASCL1/FEZF2/NEUROD1/CDK5R2/FOXG1/RELN/POU3F3 | 7 | 0.377964473 |
| BP | GO:0042310 | vasoconstriction | 5/169 | 82/18800 | 0.00086384 | 0.029357683 | 0.024863405 | EDN3/CHRM3/FGG/FGB/LEP | 5 | 0.447213595 |
| BP | GO:0048483 | autonomic nervous system development | 4/169 | 48/18800 | 0.000900955 | 0.029686298 | 0.025141714 | SOX8/SOX10/ASCL1/GFRA3 | 4 | 1 |
| BP | GO:0015844 | monoamine transport | 5/169 | 83/18800 | 0.000912622 | 0.029686298 | 0.025141714 | OPRK1/CHGA/SYT4/SYT8/CARTPT | 5 | -0.447213595 |
| BP | GO:0033273 | response to vitamin | 5/169 | 83/18800 | 0.000912622 | 0.029686298 | 0.025141714 | OTC/SFRP1/ASCL1/LEP/PENK | 5 | 1.341640786 |
| BP | GO:0050886 | endocrine process | 5/169 | 85/18800 | 0.0010163 | 0.032140497 | 0.027220207 | OPRK1/EDN3/CGA/LEP/AGTR2 | 5 | 1.341640786 |
| BP | GO:2000177 | regulation of neural precursor cell proliferation | 5/169 | 85/18800 | 0.0010163 | 0.032140497 | 0.027220207 | SOX10/ASCL1/FOXG1/TAFA3/LHX1 | 5 | 0.447213595 |
| BP | GO:0060688 | regulation of morphogenesis of a branching structure | 4/169 | 51/18800 | 0.001133177 | 0.034868163 | 0.029530303 | SOX8/SFRP1/AGTR2/LHX1 | 4 | 1 |
| BP | GO:2000179 | positive regulation of neural precursor cell proliferation | 4/169 | 51/18800 | 0.001133177 | 0.034868163 | 0.029530303 | SOX10/ASCL1/FOXG1/LHX1 | 4 | 0 |
| BP | GO:0042476 | odontogenesis | 6/169 | 130/18800 | 0.001149186 | 0.034889292 | 0.029548197 | NGFR/FGF4/EDAR/MMP20/OSR1/KLK5 | 6 | 1.632993162 |
| BP | GO:0021801 | cerebral cortex radial glia-guided migration | 3/169 | 24/18800 | 0.001257067 | 0.035699528 | 0.030234397 | CDK5R2/FOXG1/RELN | 3 | 0.577350269 |
| BP | GO:0022030 | telencephalon glial cell migration | 3/169 | 24/18800 | 0.001257067 | 0.035699528 | 0.030234397 | CDK5R2/FOXG1/RELN | 3 | 0.577350269 |
| BP | GO:0042730 | fibrinolysis | 3/169 | 24/18800 | 0.001257067 | 0.035699528 | 0.030234397 | KRT1/FGG/FGB | 3 | -0.577350269 |
| BP | GO:0072207 | metanephric epithelium development | 3/169 | 24/18800 | 0.001257067 | 0.035699528 | 0.030234397 | SOX8/OSR1/POU3F3 | 3 | 1.732050808 |
| BP | GO:0007411 | axon guidance | 8/169 | 234/18800 | 0.001281762 | 0.035699528 | 0.030234397 | NGFR/EDN3/GFRA3/FEZF2/CDK5R2/FOXG1/RELN/LHX1 | 8 | 1.414213562 |
| BP | GO:0009306 | protein secretion | 10/169 | 350/18800 | 0.001287841 | 0.035699528 | 0.030234397 | CHGA/SFRP1/SYT4/NEUROD1/CARTPT/SLC30A8/FGG/FGB/LEP/PCSK1 | 10 | -1.897366596 |
| BP | GO:0042475 | odontogenesis of dentin-containing tooth | 5/169 | 90/18800 | 0.001313606 | 0.035699528 | 0.030234397 | NGFR/FGF4/EDAR/MMP20/KLK5 | 5 | 1.341640786 |
| BP | GO:0035592 | establishment of protein localization to extracellular region | 10/169 | 351/18800 | 0.001315523 | 0.035699528 | 0.030234397 | CHGA/SFRP1/SYT4/NEUROD1/CARTPT/SLC30A8/FGG/FGB/LEP/PCSK1 | 10 | -1.897366596 |
| BP | GO:0097485 | neuron projection guidance | 8/169 | 235/18800 | 0.001316979 | 0.035699528 | 0.030234397 | NGFR/EDN3/GFRA3/FEZF2/CDK5R2/FOXG1/RELN/LHX1 | 8 | 1.414213562 |
| BP | GO:0045666 | positive regulation of neuron differentiation | 5/169 | 91/18800 | 0.001380026 | 0.036968448 | 0.031309062 | LIN28A/ASCL1/FEZF2/NEUROD1/FOXG1 | 5 | -0.447213595 |
| BP | GO:0003002 | regionalization | 10/169 | 354/18800 | 0.001401496 | 0.037107038 | 0.031426435 | ALX4/SFRP1/ASCL1/OSR1/FEZF2/NEUROD1/IRX1/FOXG1/RELN/LHX1 | 10 | 1.264911064 |
| BP | GO:0043588 | skin development | 9/169 | 296/18800 | 0.001464205 | 0.038321773 | 0.03245521 | ALX4/NGFR/EDAR/KLK5/KRT1/KRT79/LCE1A/LCE2A/LORICRIN | 9 | 3 |
| BP | GO:0007586 | digestion | 6/169 | 137/18800 | 0.001504416 | 0.038616203 | 0.032704567 | OPRK1/CCKBR/CHRM3/CHIA/NEUROD1/LEP | 6 | 1.632993162 |
| BP | GO:0060993 | kidney morphogenesis | 5/169 | 93/18800 | 0.001520253 | 0.038616203 | 0.032704567 | SOX8/OSR1/IRX1/AGTR2/LHX1 | 5 | 1.341640786 |
| BP | GO:0071692 | protein localization to extracellular region | 10/169 | 359/18800 | 0.001554923 | 0.038616203 | 0.032704567 | CHGA/SFRP1/SYT4/NEUROD1/CARTPT/SLC30A8/FGG/FGB/LEP/PCSK1 | 10 | -1.897366596 |
| BP | GO:0031644 | regulation of nervous system process | 6/169 | 138/18800 | 0.001561301 | 0.038616203 | 0.032704567 | OPRK1/SOX10/CARTPT/OPRPN/SMR3B/RELN | 6 | 1.632993162 |
| BP | GO:0030318 | melanocyte differentiation | 3/169 | 26/18800 | 0.001593677 | 0.038616203 | 0.032704567 | SOX10/OCA2/EDN3 | 3 | 1.732050808 |
| BP | GO:0045745 | positive regulation of G protein-coupled receptor signaling pathway | 3/169 | 26/18800 | 0.001593677 | 0.038616203 | 0.032704567 | CHGA/KLK5/KLK6 | 3 | 0.577350269 |
| BP | GO:0001657 | ureteric bud development | 5/169 | 94/18800 | 0.001594169 | 0.038616203 | 0.032704567 | SOX8/SFRP1/OSR1/AGTR2/LHX1 | 5 | 1.341640786 |
| BP | GO:0072163 | mesonephric epithelium development | 5/169 | 95/18800 | 0.001670694 | 0.039527214 | 0.033476114 | SOX8/SFRP1/OSR1/AGTR2/LHX1 | 5 | 1.341640786 |
| BP | GO:0072164 | mesonephric tubule development | 5/169 | 95/18800 | 0.001670694 | 0.039527214 | 0.033476114 | SOX8/SFRP1/OSR1/AGTR2/LHX1 | 5 | 1.341640786 |
| BP | GO:0035296 | regulation of tube diameter | 6/169 | 141/18800 | 0.001741814 | 0.039527214 | 0.033476114 | EDN3/CHRM3/FGG/FGB/LEP/AGTR2 | 6 | 0.816496581 |
| BP | GO:0070555 | response to interleukin-1 | 6/169 | 141/18800 | 0.001741814 | 0.039527214 | 0.033476114 | SFRP1/SLC30A8/FGG/FGB/PCSK1/CCL14 | 6 | -0.816496581 |
| BP | GO:0097746 | blood vessel diameter maintenance | 6/169 | 141/18800 | 0.001741814 | 0.039527214 | 0.033476114 | EDN3/CHRM3/FGG/FGB/LEP/AGTR2 | 6 | 0.816496581 |
| BP | GO:0051953 | negative regulation of amine transport | 3/169 | 27/18800 | 0.001781123 | 0.039527214 | 0.033476114 | CHGA/SYT4/LEP | 3 | -0.577350269 |
| BP | GO:0007626 | locomotory behavior | 7/169 | 192/18800 | 0.001782524 | 0.039527214 | 0.033476114 | SEZ6/OPRK1/PAK5/FEZF2/PENK/NPY2R/RELN | 7 | 1.889822365 |
| BP | GO:0045664 | regulation of neuron differentiation | 7/169 | 192/18800 | 0.001782524 | 0.039527214 | 0.033476114 | SOX8/SFRP1/LIN28A/ASCL1/FEZF2/NEUROD1/FOXG1 | 7 | 0.377964473 |
| BP | GO:0021537 | telencephalon development | 8/169 | 247/18800 | 0.001802823 | 0.039527214 | 0.033476114 | ASCL1/FEZF2/NEUROD1/CDK5R2/FOXG1/RELN/POU3F3/LHX1 | 8 | 0 |
| BP | GO:0035150 | regulation of tube size | 6/169 | 142/18800 | 0.001805371 | 0.039527214 | 0.033476114 | EDN3/CHRM3/FGG/FGB/LEP/AGTR2 | 6 | 0.816496581 |
| BP | GO:0061351 | neural precursor cell proliferation | 6/169 | 143/18800 | 0.001870673 | 0.040079827 | 0.033944129 | SOX10/ASCL1/FOXG1/TAFA3/POU3F3/LHX1 | 6 | 0.816496581 |
| BP | GO:1903531 | negative regulation of secretion by cell | 6/169 | 143/18800 | 0.001870673 | 0.040079827 | 0.033944129 | OPRK1/CHGA/SFRP1/SYT4/CARTPT/LEP | 6 | 0 |
| BP | GO:0021549 | cerebellum development | 5/169 | 98/18800 | 0.001916469 | 0.040079827 | 0.033944129 | SEZ6/NEUROD1/CDK5R2/GDF10/LHX1 | 5 | -1.341640786 |
| BP | GO:0051592 | response to calcium ion | 6/169 | 144/18800 | 0.001937749 | 0.040079827 | 0.033944129 | SYT4/SYT8/FGG/FGB/PCSK1/PENK | 6 | -0.816496581 |
| BP | GO:0006826 | iron ion transport | 4/169 | 59/18800 | 0.001952109 | 0.040079827 | 0.033944129 | FTHL17/TTYH1/SCARA5/FTMT | 4 | 2 |
| BP | GO:0014072 | response to isoquinoline alkaloid | 3/169 | 28/18800 | 0.001981776 | 0.040079827 | 0.033944129 | OPRK1/PCSK1/PENK | 3 | 0.577350269 |
| BP | GO:0035116 | embryonic hindlimb morphogenesis | 3/169 | 28/18800 | 0.001981776 | 0.040079827 | 0.033944129 | ALX4/FGF4/OSR1 | 3 | 0.577350269 |
| BP | GO:0043278 | response to morphine | 3/169 | 28/18800 | 0.001981776 | 0.040079827 | 0.033944129 | OPRK1/PCSK1/PENK | 3 | 0.577350269 |
| BP | GO:0001823 | mesonephros development | 5/169 | 99/18800 | 0.002003979 | 0.040079827 | 0.033944129 | SOX8/SFRP1/OSR1/AGTR2/LHX1 | 5 | 1.341640786 |
| BP | GO:0072006 | nephron development | 6/169 | 145/18800 | 0.002006632 | 0.040079827 | 0.033944129 | SOX8/OSR1/IRX1/AGTR2/POU3F3/LHX1 | 6 | 1.632993162 |
| BP | GO:0050796 | regulation of insulin secretion | 6/169 | 147/18800 | 0.002149937 | 0.04256876 | 0.036052039 | CHGA/SFRP1/NEUROD1/CARTPT/SLC30A8/LEP | 6 | -0.816496581 |
| BP | GO:0000041 | transition metal ion transport | 5/169 | 101/18800 | 0.002187709 | 0.042943218 | 0.036369173 | FTHL17/SLC30A8/TTYH1/SCARA5/FTMT | 5 | 1.341640786 |
| BP | GO:0007405 | neuroblast proliferation | 4/169 | 61/18800 | 0.002207451 | 0.042960398 | 0.036383722 | SOX10/ASCL1/FOXG1/TAFA3 | 4 | 1 |
| BP | GO:0030902 | hindbrain development | 6/169 | 150/18800 | 0.002379219 | 0.045910868 | 0.038882514 | SEZ6/ASCL1/NEUROD1/CDK5R2/GDF10/LHX1 | 6 | -1.632993162 |
| BP | GO:0022600 | digestive system process | 5/169 | 104/18800 | 0.002485918 | 0.047566679 | 0.040284842 | OPRK1/CCKBR/CHRM3/NEUROD1/LEP | 5 | 1.341640786 |
| BP | GO:0072171 | mesonephric tubule morphogenesis | 4/169 | 64/18800 | 0.002632278 | 0.049947469 | 0.042301164 | SOX8/OSR1/AGTR2/LHX1 | 4 | 1 |
| BP | GO:0071295 | cellular response to vitamin | 3/169 | 31/18800 | 0.002666091 | 0.05017099 | 0.042490467 | SFRP1/LEP/PENK | 3 | 1.732050808 |
| BP | GO:0017156 | calcium-ion regulated exocytosis | 4/169 | 65/18800 | 0.002785502 | 0.051565758 | 0.043671714 | ZP4/SYT4/SYT8/CDK5R2 | 4 | 0 |
| BP | GO:1905330 | regulation of morphogenesis of an epithelium | 4/169 | 65/18800 | 0.002785502 | 0.051565758 | 0.043671714 | SOX8/SFRP1/AGTR2/LHX1 | 4 | 1 |
| BP | GO:0003338 | metanephros morphogenesis | 3/169 | 32/18800 | 0.002922633 | 0.053668033 | 0.045452158 | SOX8/AGTR2/LHX1 | 3 | 0.577350269 |
| BP | GO:0051930 | regulation of sensory perception of pain | 3/169 | 33/18800 | 0.003193858 | 0.057717574 | 0.048881768 | OPRK1/OPRPN/SMR3B | 3 | 1.732050808 |
| BP | GO:0051931 | regulation of sensory perception | 3/169 | 33/18800 | 0.003193858 | 0.057717574 | 0.048881768 | OPRK1/OPRPN/SMR3B | 3 | 1.732050808 |
| BP | GO:0021987 | cerebral cortex development | 5/169 | 112/18800 | 0.003424512 | 0.058585063 | 0.049616456 | ASCL1/CDK5R2/FOXG1/RELN/POU3F3 | 5 | 0.447213595 |
| BP | GO:0008343 | adult feeding behavior | 2/169 | 10/18800 | 0.003447694 | 0.058585063 | 0.049616456 | CARTPT/LEP | 2 | 0 |
| BP | GO:0021548 | pons development | 2/169 | 10/18800 | 0.003447694 | 0.058585063 | 0.049616456 | ASCL1/CDK5R2 | 2 | -1.414213562 |
| BP | GO:0021892 | cerebral cortex GABAergic interneuron differentiation | 2/169 | 10/18800 | 0.003447694 | 0.058585063 | 0.049616456 | ASCL1/FEZF2 | 2 | 0 |
| BP | GO:0070091 | glucagon secretion | 2/169 | 10/18800 | 0.003447694 | 0.058585063 | 0.049616456 | CARTPT/LEP | 2 | 0 |
| BP | GO:0070092 | regulation of glucagon secretion | 2/169 | 10/18800 | 0.003447694 | 0.058585063 | 0.049616456 | CARTPT/LEP | 2 | 0 |
| BP | GO:0072070 | loop of Henle development | 2/169 | 10/18800 | 0.003447694 | 0.058585063 | 0.049616456 | IRX1/POU3F3 | 2 | 1.414213562 |
| BP | GO:0090184 | positive regulation of kidney development | 2/169 | 10/18800 | 0.003447694 | 0.058585063 | 0.049616456 | SOX8/AGTR2 | 2 | 1.414213562 |
| BP | GO:1902692 | regulation of neuroblast proliferation | 3/169 | 34/18800 | 0.003480027 | 0.058696454 | 0.049710794 | SOX10/FOXG1/TAFA3 | 3 | 1.732050808 |
| BP | GO:0003407 | neural retina development | 4/169 | 70/18800 | 0.003643872 | 0.060562748 | 0.051291383 | SOX8/RPE65/NEUROD1/LHX1 | 4 | 0 |
| BP | GO:0042698 | ovulation cycle | 4/169 | 70/18800 | 0.003643872 | 0.060562748 | 0.051291383 | OPRK1/PTX3/LEP/GDF10 | 4 | 2 |
| BP | GO:0035137 | hindlimb morphogenesis | 3/169 | 35/18800 | 0.003781392 | 0.062392967 | 0.052841419 | ALX4/FGF4/OSR1 | 3 | 0.577350269 |
| BP | GO:0045665 | negative regulation of neuron differentiation | 4/169 | 72/18800 | 0.004032349 | 0.064016285 | 0.054216228 | SOX8/ASCL1/FEZF2/FOXG1 | 4 | 1 |
| BP | GO:0030216 | keratinocyte differentiation | 6/169 | 167/18800 | 0.0040431 | 0.064016285 | 0.054216228 | KLK5/KRT1/KRT79/LCE1A/LCE2A/LORICRIN | 6 | 2.449489743 |
| BP | GO:0051048 | negative regulation of secretion | 6/169 | 167/18800 | 0.0040431 | 0.064016285 | 0.054216228 | OPRK1/CHGA/SFRP1/SYT4/CARTPT/LEP | 6 | 0 |
| BP | GO:0050931 | pigment cell differentiation | 3/169 | 36/18800 | 0.004098194 | 0.064016285 | 0.054216228 | SOX10/OCA2/EDN3 | 3 | 1.732050808 |
| BP | GO:1905332 | positive regulation of morphogenesis of an epithelium | 3/169 | 36/18800 | 0.004098194 | 0.064016285 | 0.054216228 | SOX8/AGTR2/LHX1 | 3 | 0.577350269 |
| BP | GO:0002551 | mast cell chemotaxis | 2/169 | 11/18800 | 0.004189032 | 0.064016285 | 0.054216228 | CHGA/VEGFD | 2 | 0 |
| BP | GO:0032096 | negative regulation of response to food | 2/169 | 11/18800 | 0.004189032 | 0.064016285 | 0.054216228 | CARTPT/LEP | 2 | 0 |
| BP | GO:0032099 | negative regulation of appetite | 2/169 | 11/18800 | 0.004189032 | 0.064016285 | 0.054216228 | CARTPT/LEP | 2 | 0 |
| BP | GO:0032105 | negative regulation of response to extracellular stimulus | 2/169 | 11/18800 | 0.004189032 | 0.064016285 | 0.054216228 | CARTPT/LEP | 2 | 0 |
| BP | GO:0032108 | negative regulation of response to nutrient levels | 2/169 | 11/18800 | 0.004189032 | 0.064016285 | 0.054216228 | CARTPT/LEP | 2 | 0 |
| BP | GO:0032276 | regulation of gonadotropin secretion | 2/169 | 11/18800 | 0.004189032 | 0.064016285 | 0.054216228 | OPRK1/LEP | 2 | 1.414213562 |
| BP | GO:0033555 | multicellular organismal response to stress | 4/169 | 73/18800 | 0.004236694 | 0.064313015 | 0.054467533 | TAC1/PENK/GABRA5/RELN | 4 | 2 |
| BP | GO:0014046 | dopamine secretion | 3/169 | 37/18800 | 0.004430664 | 0.066372511 | 0.056211747 | OPRK1/SYT4/SYT8 | 3 | 0.577350269 |
| BP | GO:0014059 | regulation of dopamine secretion | 3/169 | 37/18800 | 0.004430664 | 0.066372511 | 0.056211747 | OPRK1/SYT4/SYT8 | 3 | 0.577350269 |
| BP | GO:0010038 | response to metal ion | 9/169 | 351/18800 | 0.004563186 | 0.067910951 | 0.057514672 | OTC/SYT4/ASCL1/SYT8/SLC30A8/FGG/FGB/PCSK1/PENK | 9 | -1 |
| BP | GO:0042445 | hormone metabolic process | 7/169 | 230/18800 | 0.004847174 | 0.068546708 | 0.058053102 | RPE65/CGA/SLC30A8/KLK6/LEP/PCSK1/ADH4 | 7 | 0.377964473 |
| BP | GO:0061045 | negative regulation of wound healing | 4/169 | 76/18800 | 0.004891429 | 0.068546708 | 0.058053102 | CLDN19/KRT1/FGG/FGB | 4 | 0 |
| BP | GO:0042063 | gliogenesis | 8/169 | 291/18800 | 0.004893834 | 0.068546708 | 0.058053102 | SOX8/SOX10/LIN28A/ASCL1/CDK5R2/FOXG1/PENK/RELN | 8 | 0.707106781 |
| BP | GO:0046887 | positive regulation of hormone secretion | 5/169 | 122/18800 | 0.00492639 | 0.068546708 | 0.058053102 | EDN3/SLC30A8/FGG/FGB/LEP | 5 | -0.447213595 |
| BP | GO:0010587 | miRNA catabolic process | 2/169 | 12/18800 | 0.004997257 | 0.068546708 | 0.058053102 | LIN28A/LIN28B | 2 | 0 |
| BP | GO:0021527 | spinal cord association neuron differentiation | 2/169 | 12/18800 | 0.004997257 | 0.068546708 | 0.058053102 | ASCL1/LHX1 | 2 | -1.414213562 |
| BP | GO:0021781 | glial cell fate commitment | 2/169 | 12/18800 | 0.004997257 | 0.068546708 | 0.058053102 | SOX8/ASCL1 | 2 | 0 |
| BP | GO:0043696 | dedifferentiation | 2/169 | 12/18800 | 0.004997257 | 0.068546708 | 0.058053102 | FEZF2/MIR145 | 2 | 1.414213562 |
| BP | GO:0043697 | cell dedifferentiation | 2/169 | 12/18800 | 0.004997257 | 0.068546708 | 0.058053102 | FEZF2/MIR145 | 2 | 1.414213562 |
| BP | GO:0046541 | saliva secretion | 2/169 | 12/18800 | 0.004997257 | 0.068546708 | 0.058053102 | OPRK1/CHRM3 | 2 | 1.414213562 |
| BP | GO:0072178 | nephric duct morphogenesis | 2/169 | 12/18800 | 0.004997257 | 0.068546708 | 0.058053102 | OSR1/LHX1 | 2 | 0 |
| BP | GO:0072182 | regulation of nephron tubule epithelial cell differentiation | 2/169 | 12/18800 | 0.004997257 | 0.068546708 | 0.058053102 | OSR1/LHX1 | 2 | 0 |
| BP | GO:0097531 | mast cell migration | 2/169 | 12/18800 | 0.004997257 | 0.068546708 | 0.058053102 | CHGA/VEGFD | 2 | 0 |
| BP | GO:0032570 | response to progesterone | 3/169 | 39/18800 | 0.005143481 | 0.06971254 | 0.059040461 | SOX10/CSN1S1/DSG1 | 3 | 1.732050808 |
| BP | GO:0032941 | secretion by tissue | 3/169 | 39/18800 | 0.005143481 | 0.06971254 | 0.059040461 | OPRK1/CHRM3/LACRT | 3 | 0.577350269 |
| BP | GO:0046660 | female sex differentiation | 5/169 | 124/18800 | 0.005275046 | 0.071072659 | 0.060192364 | COL9A3/SFRP1/PTX3/LEP/LHX1 | 5 | 1.341640786 |
| BP | GO:0048511 | rhythmic process | 8/169 | 297/18800 | 0.005519507 | 0.073131959 | 0.061936412 | NGFR/OPRK1/RPE65/PTX3/CARTPT/SOX14/LEP/GDF10 | 8 | 2.121320344 |
| BP | GO:0017158 | regulation of calcium ion-dependent exocytosis | 3/169 | 40/18800 | 0.005524241 | 0.073131959 | 0.061936412 | SYT4/SYT8/CDK5R2 | 3 | -0.577350269 |
| BP | GO:0051954 | positive regulation of amine transport | 3/169 | 40/18800 | 0.005524241 | 0.073131959 | 0.061936412 | OPRK1/SYT4/CARTPT | 3 | -0.577350269 |
| BP | GO:0030073 | insulin secretion | 6/169 | 179/18800 | 0.005651676 | 0.074386509 | 0.062998906 | CHGA/SFRP1/NEUROD1/CARTPT/SLC30A8/LEP | 6 | -0.816496581 |
| BP | GO:0021766 | hippocampus development | 4/169 | 80/18800 | 0.005865556 | 0.074684568 | 0.063251337 | FEZF2/NEUROD1/CDK5R2/RELN | 4 | 0 |
| BP | GO:0021954 | central nervous system neuron development | 4/169 | 80/18800 | 0.005865556 | 0.074684568 | 0.063251337 | ASCL1/FEZF2/LEP/FOXG1 | 4 | 1 |
| BP | GO:0021819 | layer formation in cerebral cortex | 2/169 | 13/18800 | 0.005871119 | 0.074684568 | 0.063251337 | CDK5R2/RELN | 2 | 0 |
| BP | GO:0033604 | negative regulation of catecholamine secretion | 2/169 | 13/18800 | 0.005871119 | 0.074684568 | 0.063251337 | CHGA/SYT4 | 2 | -1.414213562 |
| BP | GO:0033605 | positive regulation of catecholamine secretion | 2/169 | 13/18800 | 0.005871119 | 0.074684568 | 0.063251337 | OPRK1/CARTPT | 2 | 0 |
| BP | GO:0097154 | GABAergic neuron differentiation | 2/169 | 13/18800 | 0.005871119 | 0.074684568 | 0.063251337 | ASCL1/FEZF2 | 2 | 0 |
| BP | GO:0021879 | forebrain neuron differentiation | 3/169 | 41/18800 | 0.005921495 | 0.074906906 | 0.063439637 | ASCL1/FEZF2/FOXG1 | 3 | 0.577350269 |
| BP | GO:0001822 | kidney development | 8/169 | 303/18800 | 0.006204553 | 0.077850119 | 0.065932283 | SOX8/SFRP1/LIN28A/OSR1/IRX1/AGTR2/POU3F3/LHX1 | 8 | 1.414213562 |
| BP | GO:2000027 | regulation of animal organ morphogenesis | 5/169 | 129/18800 | 0.006222539 | 0.077850119 | 0.065932283 | SOX8/NGFR/SFRP1/AGTR2/LHX1 | 5 | 1.341640786 |
| BP | GO:0031670 | cellular response to nutrient | 3/169 | 42/18800 | 0.006335424 | 0.078829296 | 0.066761561 | SFRP1/LEP/PENK | 3 | 1.732050808 |
| BP | GO:0045921 | positive regulation of exocytosis | 4/169 | 83/18800 | 0.006675493 | 0.081605137 | 0.069112457 | SYT4/CDK5R2/FGG/FGB | 4 | -2 |
| BP | GO:0001709 | cell fate determination | 3/169 | 43/18800 | 0.006766204 | 0.081605137 | 0.069112457 | ASCL1/FEZF2/FOXG1 | 3 | 0.577350269 |
| BP | GO:0016339 | calcium-dependent cell-cell adhesion via plasma membrane cell adhesion molecules | 3/169 | 43/18800 | 0.006766204 | 0.081605137 | 0.069112457 | CDH19/DSG1/CDH12 | 3 | 1.732050808 |
| BP | GO:0044058 | regulation of digestive system process | 3/169 | 43/18800 | 0.006766204 | 0.081605137 | 0.069112457 | OPRK1/NEUROD1/LEP | 3 | 0.577350269 |
| BP | GO:0031054 | pre-miRNA processing | 2/169 | 14/18800 | 0.006809388 | 0.081605137 | 0.069112457 | LIN28A/LIN28B | 2 | 0 |
| BP | GO:0038166 | angiotensin-activated signaling pathway | 2/169 | 14/18800 | 0.006809388 | 0.081605137 | 0.069112457 | AGTR2/MIR145 | 2 | 1.414213562 |
| BP | GO:0072160 | nephron tubule epithelial cell differentiation | 2/169 | 14/18800 | 0.006809388 | 0.081605137 | 0.069112457 | OSR1/LHX1 | 2 | 0 |
| BP | GO:0030900 | forebrain development | 9/169 | 376/18800 | 0.007066907 | 0.084247895 | 0.071350644 | ASCL1/FEZF2/NEUROD1/CDK5R2/PCSK1/FOXG1/RELN/POU3F3/LHX1 | 9 | -0.333333333 |
| BP | GO:0015850 | organic hydroxy compound transport | 7/169 | 248/18800 | 0.007238327 | 0.085842029 | 0.072700736 | OPRK1/CHGA/SYT4/SYT8/CARTPT/LEP/ABCA13 | 7 | 0.377964473 |
| BP | GO:0072001 | renal system development | 8/169 | 312/18800 | 0.007351059 | 0.086727259 | 0.073450449 | SOX8/SFRP1/LIN28A/OSR1/IRX1/AGTR2/POU3F3/LHX1 | 8 | 1.414213562 |
| BP | GO:0034116 | positive regulation of heterotypic cell-cell adhesion | 2/169 | 15/18800 | 0.007810849 | 0.09074134 | 0.076850027 | FGG/FGB | 2 | -1.414213562 |
| BP | GO:0048484 | enteric nervous system development | 2/169 | 15/18800 | 0.007810849 | 0.09074134 | 0.076850027 | SOX8/SOX10 | 2 | 1.414213562 |
| BP | GO:0072378 | blood coagulation, fibrin clot formation | 2/169 | 15/18800 | 0.007810849 | 0.09074134 | 0.076850027 | FGG/FGB | 2 | -1.414213562 |
| BP | GO:0021517 | ventral spinal cord development | 3/169 | 46/18800 | 0.008161253 | 0.094116885 | 0.07970882 | ASCL1/RELN/LHX1 | 3 | -0.577350269 |
| BP | GO:0045104 | intermediate filament cytoskeleton organization | 4/169 | 88/18800 | 0.008184077 | 0.094116885 | 0.07970882 | KRT1/SYNM/KRT79/KRT14 | 4 | 2 |
| BP | GO:0009582 | detection of abiotic stimulus | 5/169 | 139/18800 | 0.008465306 | 0.096408931 | 0.081649984 | TAC1/NGFR/RPE65/PHF24/RGR | 5 | 2.236067977 |
| BP | GO:0007589 | body fluid secretion | 4/169 | 89/18800 | 0.008510406 | 0.096408931 | 0.081649984 | OPRK1/CHRM3/CSN2/LACRT | 4 | 1 |
| BP | GO:0045103 | intermediate filament-based process | 4/169 | 89/18800 | 0.008510406 | 0.096408931 | 0.081649984 | KRT1/SYNM/KRT79/KRT14 | 4 | 2 |
| BP | GO:0032008 | positive regulation of TOR signaling | 3/169 | 47/18800 | 0.008660999 | 0.097148246 | 0.082276119 | LIN28A/LEP/RELN | 3 | 0.577350269 |
| BP | GO:0048066 | developmental pigmentation | 3/169 | 47/18800 | 0.008660999 | 0.097148246 | 0.082276119 | SOX10/OCA2/EDN3 | 3 | 1.732050808 |
| BP | GO:0032095 | regulation of response to food | 2/169 | 16/18800 | 0.008874304 | 0.098091211 | 0.083074728 | CARTPT/LEP | 2 | 0 |
| BP | GO:0072176 | nephric duct development | 2/169 | 16/18800 | 0.008874304 | 0.098091211 | 0.083074728 | OSR1/LHX1 | 2 | 0 |
| BP | GO:2000696 | regulation of epithelial cell differentiation involved in kidney development | 2/169 | 16/18800 | 0.008874304 | 0.098091211 | 0.083074728 | OSR1/LHX1 | 2 | 0 |
| CC | GO:0062023 | collagen-containing extracellular matrix | 16/179 | 429/19594 | 2.15862E-06 | 0.000494324 | 0.000454446 | COL17A1/COL9A3/SFRP1/COL9A1/ZP4/RTBDN/LOXL4/LEFTY2/KRT1/FGG/FGB/ANGPTL7/SNORC/EMILIN3/COL11A2/GDF10 | 16 | 2.5 |
| CC | GO:0031045 | dense core granule | 4/179 | 26/19594 | 8.60405E-05 | 0.009851637 | 0.009056894 | CHGA/SYT4/SYT8/PENK | 4 | 0 |
| CC | GO:0098992 | neuronal dense core vesicle | 3/179 | 13/19594 | 0.000200449 | 0.015300926 | 0.014066583 | CHGA/SYT4/PENK | 3 | -0.577350269 |
| CC | GO:0043025 | neuronal cell body | 13/179 | 482/19594 | 0.000504123 | 0.028861038 | 0.026532786 | TAC1/SEZ6/NGFR/OPRK1/SYT4/ASCL1/CPLX2/GRIA4/PDE1C/S100B/PCSK1/PENK/GABRA5 | 13 | 0.832050294 |
| CC | GO:0005581 | collagen trimer | 5/179 | 86/19594 | 0.001153007 | 0.045896864 | 0.042194313 | COL17A1/COL9A3/COL9A1/C1QL2/COL11A2 | 5 | 2.236067977 |
| CC | GO:0044306 | neuron projection terminus | 6/179 | 129/19594 | 0.001202538 | 0.045896864 | 0.042194313 | OPRK1/NTS/CPLX2/SCRG1/PCSK1/PENK | 6 | 0 |
| MF | GO:0048018 | receptor ligand activity | 18/173 | 489/18410 | 8.5833E-07 | 0.000178867 | 0.000165023 | FGF4/CHGB/EDN3/IL17B/NTS/CGA/LACRT/LEFTY2/TSLP/CARTPT/VEGFD/CMTM5/LEP/PENK/TAFA3/OSTN/GDF10/CCL14 | 18 | 1.414213562 |
| MF | GO:0030546 | signaling receptor activator activity | 18/173 | 496/18410 | 1.05216E-06 | 0.000178867 | 0.000165023 | FGF4/CHGB/EDN3/IL17B/NTS/CGA/LACRT/LEFTY2/TSLP/CARTPT/VEGFD/CMTM5/LEP/PENK/TAFA3/OSTN/GDF10/CCL14 | 18 | 1.414213562 |
| MF | GO:0005179 | hormone activity | 8/173 | 122/18410 | 2.00428E-05 | 0.002271518 | 0.002095704 | CHGB/EDN3/NTS/CGA/CARTPT/LEP/PENK/OSTN | 8 | 0 |
| MF | GO:0005201 | extracellular matrix structural constituent | 8/173 | 172/18410 | 0.000225474 | 0.019165248 | 0.017681869 | COL17A1/COL9A3/COL9A1/ZP4/FGG/FGB/EMILIN3/COL11A2 | 8 | 1.414213562 |
| MF | GO:0071855 | neuropeptide receptor binding | 4/173 | 36/18410 | 0.000350806 | 0.023854816 | 0.022008468 | TAC1/CCKBR/EDN3/NTS | 4 | 1 |
| MF | GO:0030020 | extracellular matrix structural constituent conferring tensile strength | 4/173 | 41/18410 | 0.000581481 | 0.032950578 | 0.030400223 | COL17A1/COL9A3/COL9A1/COL11A2 | 4 | 2 |
