# Supplementary material for: Identification and validation of DNA methylation-driven gene OSR1 as a novel tumor suppressor for the diagnosis and prognosis of breast cancer
Source: Front Genet. 2025 Jul 7;16:1583620. doi: 10.3389/fgene.2025.1583620 (PMC12277919; doi:10.3389/fgene.2025.1583620)
Supplement: Supplementary file 5 [file Table3.docx]

**Supplementary Table Sample information about the amount of OSR1 expressed**

| sample_id | express | rank | group |
| --- | --- | --- | --- |
| TCGA-A8-A08B-01A-11R-A00Z-07 | 0 | 1 | Low |
| TCGA-BH-A0EE-01A-11R-A034-07 | 0 | 2 | Low |
| TCGA-C8-A12Z-01A-11R-A115-07 | 0 | 3 | Low |
| TCGA-D8-A1XJ-01A-11R-A14M-07 | 0.028569152 | 4 | Low |
| TCGA-BH-A204-01A-11R-A157-07 | 0.032524066 | 5 | Low |
| TCGA-B6-A0IC-01A-11R-A034-07 | 0.041102772 | 6 | Low |
| TCGA-UU-A93S-01A-21R-A41B-07 | 0.046281503 | 7 | Low |
| TCGA-S3-AA12-01A-11R-A41B-07 | 0.04949137 | 8 | Low |
| TCGA-AO-A03P-01A-11R-A00Z-07 | 0.057692867 | 9 | Low |
| TCGA-AC-A23H-01A-11R-A157-07 | 0.060462488 | 10 | Low |
| TCGA-A8-A06N-01A-11R-A00Z-07 | 0.061292338 | 11 | Low |
| TCGA-A8-A07W-01A-11R-A00Z-07 | 0.068326861 | 12 | Low |
| TCGA-BH-A1F5-01A-12R-A13Q-07 | 0.073271836 | 13 | Low |
| TCGA-A8-A09W-01A-11R-A00Z-07 | 0.073820233 | 14 | Low |
| TCGA-A2-A3Y0-01A-11R-A239-07 | 0.074231394 | 15 | Low |
| TCGA-A8-A094-01A-11R-A00Z-07 | 0.078473209 | 16 | Low |
| TCGA-B6-A0X5-01A-21R-A109-07 | 0.085152681 | 17 | Low |
| TCGA-D8-A1JE-01A-11R-A13Q-07 | 0.086647913 | 18 | Low |
| TCGA-A8-A075-01A-11R-A084-07 | 0.08841301 | 19 | Low |
| TCGA-A2-A3XU-01A-12R-A22U-07 | 0.092748686 | 20 | Low |
| TCGA-BH-A5IZ-01A-11R-A27Q-07 | 0.095991913 | 21 | Low |
| TCGA-E2-A14O-01A-31R-A115-07 | 0.096801582 | 22 | Low |
| TCGA-D8-A1JA-01A-11R-A13Q-07 | 0.096936483 | 23 | Low |
| TCGA-AQ-A04L-01B-21R-A10J-07 | 0.099497201 | 24 | Low |
| TCGA-BH-A0HW-01A-11R-A034-07 | 0.100035721 | 25 | Low |
| TCGA-D8-A1X6-01A-11R-A14M-07 | 0.104202449 | 26 | Low |
| TCGA-E9-A5UO-01A-11R-A28M-07 | 0.104739216 | 27 | Low |
| TCGA-C8-A133-01A-32R-A12D-07 | 0.111832587 | 28 | Low |
| TCGA-A7-A0DC-01A-11R-A00Z-07 | 0.113700499 | 29 | Low |
| TCGA-UL-AAZ6-01A-11R-A41B-07 | 0.115299644 | 30 | Low |
| TCGA-EW-A6SD-01A-12R-A33J-07 | 0.121148056 | 31 | Low |
| TCGA-A7-A4SF-01A-11R-A266-07 | 0.122208862 | 32 | Low |
| TCGA-C8-A12M-01A-11R-A115-07 | 0.125783332 | 33 | Low |
| TCGA-D8-A1JS-01A-11R-A13Q-07 | 0.125915549 | 34 | Low |
| TCGA-AN-A0AK-01A-21R-A00Z-07 | 0.128821281 | 35 | Low |
| TCGA-E9-A1RE-01A-11R-A157-07 | 0.129085148 | 36 | Low |
| TCGA-E9-A54Y-01A-11R-A466-07 | 0.129744606 | 37 | Low |
| TCGA-A7-A56D-01A-11R-A27Q-07 | 0.132642643 | 38 | Low |
| TCGA-A8-A08J-01A-11R-A00Z-07 | 0.135666197 | 39 | Low |
| TCGA-D8-A1JG-01B-11R-A13Q-07 | 0.136322654 | 40 | Low |
| TCGA-C8-A26Y-01A-11R-A16F-07 | 0.137634672 | 41 | Low |
| TCGA-D8-A1XC-01A-11R-A14D-07 | 0.138552375 | 42 | Low |
| TCGA-C8-A131-01A-11R-A115-07 | 0.140647793 | 43 | Low |
| TCGA-AO-A12H-01A-11R-A115-07 | 0.141040345 | 44 | Low |
| TCGA-AO-A0J3-01A-11R-A034-07 | 0.141171172 | 45 | Low |
| TCGA-B6-A0I2-01A-11R-A034-07 | 0.143001506 | 46 | Low |
| TCGA-D8-A1JK-01A-11R-A13Q-07 | 0.143393419 | 47 | Low |
| TCGA-AR-A24Z-01A-11R-A169-07 | 0.149649582 | 48 | Low |
| TCGA-C8-A1HF-01A-11R-A137-07 | 0.151599082 | 49 | Low |
| TCGA-A8-A09I-01A-22R-A034-07 | 0.153027043 | 50 | Low |
| TCGA-BH-A1FJ-01A-11R-A13Q-07 | 0.153935011 | 51 | Low |
| TCGA-E2-A10C-01A-21R-A10J-07 | 0.154583209 | 52 | Low |
| TCGA-E2-A574-01A-11R-A29R-07 | 0.155360663 | 53 | Low |
| TCGA-C8-A137-01A-11R-A115-07 | 0.159758338 | 54 | Low |
| TCGA-A8-A09Q-01A-11R-A00Z-07 | 0.160016608 | 55 | Low |
| TCGA-AO-A0J2-01A-11R-A034-07 | 0.168642036 | 56 | Low |
| TCGA-BH-A1FM-01A-11R-A13Q-07 | 0.168770384 | 57 | Low |
| TCGA-E2-A15E-06A-11R-A12D-07 | 0.170566057 | 58 | Low |
| TCGA-BH-A1FC-01A-11R-A13Q-07 | 0.172359498 | 59 | Low |
| TCGA-E9-A1RH-01A-21R-A169-07 | 0.172359498 | 60 | Low |
| TCGA-C8-A12L-01A-11R-A115-07 | 0.173639164 | 61 | Low |
| TCGA-C8-A12P-01A-11R-A115-07 | 0.175045486 | 62 | Low |
| TCGA-AR-A250-01A-31R-A169-07 | 0.176450439 | 63 | Low |
| TCGA-C8-A274-01A-11R-A16F-07 | 0.177216201 | 64 | Low |
| TCGA-E2-A152-01A-11R-A12D-07 | 0.178109076 | 65 | Low |
| TCGA-C8-A275-01A-21R-A16F-07 | 0.180784391 | 66 | Low |
| TCGA-AR-A1AS-01A-11R-A12P-07 | 0.181038924 | 67 | Low |
| TCGA-D8-A1JI-01A-11R-A13Q-07 | 0.183327706 | 68 | Low |
| TCGA-C8-A12X-01A-11R-A115-07 | 0.184216808 | 69 | Low |
| TCGA-BH-A18U-01A-21R-A12D-07 | 0.187261005 | 70 | Low |
| TCGA-AO-A03N-01B-11R-A10J-07 | 0.189033824 | 71 | Low |
| TCGA-E2-A15J-01A-11R-A12P-07 | 0.190172345 | 72 | Low |
| TCGA-C8-A12U-01A-11R-A115-07 | 0.190298792 | 73 | Low |
| TCGA-E9-A1NA-01A-11R-A144-07 | 0.191183609 | 74 | Low |
| TCGA-AO-A0JM-01A-21R-A056-07 | 0.195221593 | 75 | Low |
| TCGA-E2-A155-01A-11R-A12D-07 | 0.196607044 | 76 | Low |
| TCGA-A8-A06Z-01A-11R-A00Z-07 | 0.199625236 | 77 | Low |
| TCGA-A8-A081-01A-11R-A00Z-07 | 0.201131967 | 78 | Low |
| TCGA-A8-A076-01A-21R-A00Z-07 | 0.203263813 | 79 | Low |
| TCGA-AQ-A1H2-01A-11R-A13Q-07 | 0.205392513 | 80 | Low |
| TCGA-A7-A3RF-01A-11R-A22K-07 | 0.207518077 | 81 | Low |
| TCGA-D8-A1J8-01A-11R-A13Q-07 | 0.209515751 | 82 | Low |
| TCGA-BH-A18S-01A-11R-A12D-07 | 0.215740713 | 83 | Low |
| TCGA-AN-A0FV-01A-11R-A00Z-07 | 0.216361735 | 84 | Low |
| TCGA-AO-A12D-01A-11R-A115-07 | 0.221938932 | 85 | Low |
| TCGA-D8-A1Y2-01A-11R-A157-07 | 0.223916751 | 86 | Low |
| TCGA-D8-A1JF-01A-11R-A13Q-07 | 0.226138561 | 87 | Low |
| TCGA-BH-A18N-01A-11R-A12D-07 | 0.231432408 | 88 | Low |
| TCGA-AR-A0TW-01A-11R-A084-07 | 0.231678162 | 89 | Low |
| TCGA-EW-A1OX-01A-11R-A144-07 | 0.235604533 | 90 | Low |
| TCGA-E2-A56Z-01A-12R-A29R-07 | 0.236094579 | 91 | Low |
| TCGA-BH-A18V-01A-11R-A12D-07 | 0.237686079 | 92 | Low |
| TCGA-AC-A3YJ-01A-11R-A22U-07 | 0.239520247 | 93 | Low |
| TCGA-E2-A159-01A-11R-A115-07 | 0.239520247 | 94 | Low |
| TCGA-EW-A2FW-01A-11R-A17B-07 | 0.239886801 | 95 | Low |
| TCGA-D8-A1X9-01A-12R-A157-07 | 0.240741731 | 96 | Low |
| TCGA-E2-A1LB-01A-11R-A144-07 | 0.241596155 | 97 | Low |
| TCGA-AR-A0U2-01A-11R-A109-07 | 0.241718175 | 98 | Low |
| TCGA-D8-A73W-01A-22R-A352-07 | 0.243303487 | 99 | Low |
| TCGA-E9-A1N6-01A-11R-A144-07 | 0.245739032 | 100 | Low |
| TCGA-BH-A1FD-01A-11R-A13Q-07 | 0.246468895 | 101 | Low |
| TCGA-A8-A09M-01A-11R-A00Z-07 | 0.247319935 | 102 | Low |
| TCGA-AC-A8OR-01A-21R-A41B-07 | 0.249506021 | 103 | Low |
| TCGA-BH-A1F8-01A-11R-A13Q-07 | 0.252052275 | 104 | Low |
| TCGA-AN-A0AJ-01A-11R-A00Z-07 | 0.252900029 | 105 | Low |
| TCGA-LL-A5YM-01A-11R-A28M-07 | 0.252900029 | 106 | Low |
| TCGA-BH-A0E7-01A-11R-A034-07 | 0.254835883 | 107 | Low |
| TCGA-AO-A0J8-01A-21R-A034-07 | 0.25918208 | 108 | Low |
| TCGA-A8-A08P-01A-11R-A00Z-07 | 0.260868739 | 109 | Low |
| TCGA-A2-A0D1-01A-11R-A034-07 | 0.262793937 | 110 | Low |
| TCGA-D8-A27E-01A-11R-A16F-07 | 0.267236142 | 111 | Low |
| TCGA-EW-A1J6-01A-11R-A13Q-07 | 0.268434394 | 112 | Low |
| TCGA-A2-A25B-01A-11R-A169-07 | 0.273098089 | 113 | Low |
| TCGA-C8-A1HL-01A-11R-A137-07 | 0.273336847 | 114 | Low |
| TCGA-A2-A0CW-01A-21R-A115-07 | 0.277627748 | 115 | Low |
| TCGA-A2-A4S0-01A-21R-A266-07 | 0.277627748 | 116 | Low |
| TCGA-D8-A1XV-01A-11R-A14M-07 | 0.282143229 | 117 | Low |
| TCGA-E2-A9RU-01A-11R-A41B-07 | 0.285224645 | 118 | Low |
| TCGA-A8-A09X-01A-11R-A00Z-07 | 0.289480386 | 119 | Low |
| TCGA-E9-A1RC-01A-11R-A157-07 | 0.290778251 | 120 | Low |
| TCGA-E2-A109-01A-11R-A10J-07 | 0.292781749 | 121 | Low |
| TCGA-E2-A153-01A-12R-A12D-07 | 0.293252756 | 122 | Low |
| TCGA-C8-A1HG-01A-11R-A137-07 | 0.294076649 | 123 | Low |
| TCGA-A8-A0AD-01A-11R-A056-07 | 0.295958068 | 124 | Low |
| TCGA-E2-A10A-01A-21R-A115-07 | 0.298071735 | 125 | Low |
| TCGA-A2-A04X-01A-21R-A034-07 | 0.299244658 | 126 | Low |
| TCGA-AN-A0FZ-01A-11R-A034-07 | 0.301119353 | 127 | Low |
| TCGA-D8-A1XT-01A-11R-A14M-07 | 0.307778227 | 128 | Low |
| TCGA-LL-A442-01A-11R-A24H-07 | 0.309059743 | 129 | Low |
| TCGA-BH-A1ES-06A-12R-A24H-07 | 0.309292623 | 130 | Low |
| TCGA-D8-A1XR-01A-11R-A14M-07 | 0.30987466 | 131 | Low |
| TCGA-D8-A1JN-01A-11R-A13Q-07 | 0.318461465 | 132 | Low |
| TCGA-E9-A1R6-01A-11R-A14D-07 | 0.319964694 | 133 | Low |
| TCGA-C8-A27A-01A-11R-A169-07 | 0.320426911 | 134 | Low |
| TCGA-BH-A0DL-01A-11R-A115-07 | 0.321004474 | 135 | Low |
| TCGA-AN-A0XP-01A-11R-A109-07 | 0.324349789 | 136 | Low |
| TCGA-GM-A2DM-01A-11R-A180-07 | 0.326422296 | 137 | Low |
| TCGA-A7-A3J0-01A-11R-A213-07 | 0.327227468 | 138 | Low |
| TCGA-PL-A8LX-01A-11R-A41B-07 | 0.329410671 | 139 | Low |
| TCGA-C8-A278-01A-11R-A169-07 | 0.329525485 | 140 | Low |
| TCGA-A7-A0CD-01A-11R-A00Z-07 | 0.333996118 | 141 | Low |
| TCGA-AR-A24H-01A-11R-A169-07 | 0.334911461 | 142 | Low |
| TCGA-E2-A156-01A-11R-A12D-07 | 0.339479485 | 143 | Low |
| TCGA-A8-A09C-01A-11R-A00Z-07 | 0.344714894 | 144 | Low |
| TCGA-E9-A1RG-01A-11R-A14D-07 | 0.34619103 | 145 | Low |
| TCGA-A8-A0A9-01A-11R-A00Z-07 | 0.346644922 | 146 | Low |
| TCGA-AN-A041-01A-11R-A034-07 | 0.34857237 | 147 | Low |
| TCGA-A8-A096-01A-11R-A00Z-07 | 0.351176003 | 148 | Low |
| TCGA-BH-A0BZ-01A-31R-A12P-07 | 0.351741389 | 149 | Low |
| TCGA-AR-A0TV-01A-21R-A084-07 | 0.353436218 | 150 | Low |
| TCGA-A8-A08A-01A-11R-A32Y-07 | 0.358171175 | 151 | Low |
| TCGA-BH-A202-01A-11R-A14M-07 | 0.364236231 | 152 | Low |
| TCGA-E9-A1NG-01A-21R-A14M-07 | 0.366140336 | 153 | Low |
| TCGA-D8-A1X5-01A-11R-A14D-07 | 0.366923649 | 154 | Low |
| TCGA-EW-A2FR-01A-11R-A21T-07 | 0.368600747 | 155 | Low |
| TCGA-A2-A0SV-01A-11R-A084-07 | 0.370275897 | 156 | Low |
| TCGA-A8-A06X-01A-21R-A00Z-07 | 0.374510926 | 157 | Low |
| TCGA-D8-A1X7-01A-11R-A14M-07 | 0.374955996 | 158 | Low |
| TCGA-C8-A26X-01A-31R-A16F-07 | 0.380507819 | 159 | Low |
| TCGA-E9-A3Q9-01A-11R-A21T-07 | 0.380729449 | 160 | Low |
| TCGA-AN-A0XR-01A-11R-A109-07 | 0.380951044 | 161 | Low |
| TCGA-D8-A1JJ-01A-31R-A14M-07 | 0.382611923 | 162 | Low |
| TCGA-A8-A07I-01A-11R-A00Z-07 | 0.388575307 | 163 | Low |
| TCGA-S3-AA0Z-01A-11R-A41B-07 | 0.390777727 | 164 | Low |
| TCGA-C8-A1HN-01A-11R-A137-07 | 0.390997784 | 165 | Low |
| TCGA-A8-A08L-01A-11R-A00Z-07 | 0.397145797 | 166 | Low |
| TCGA-AR-A0TT-01A-31R-A084-07 | 0.399335183 | 167 | Low |
| TCGA-A8-A082-01A-11R-A00Z-07 | 0.399553938 | 168 | Low |
| TCGA-E9-A1R7-01A-11R-A14M-07 | 0.401084302 | 169 | Low |
| TCGA-EW-A1P0-01A-11R-A144-07 | 0.401630467 | 170 | Low |
| TCGA-AR-A0TR-01A-11R-A084-07 | 0.40294042 | 171 | Low |
| TCGA-E9-A24A-01A-11R-A169-07 | 0.40729836 | 172 | Low |
| TCGA-E9-A22E-01A-11R-A157-07 | 0.410992287 | 173 | Low |
| TCGA-A2-A0CQ-01A-21R-A034-07 | 0.412944073 | 174 | Low |
| TCGA-A7-A0DB-01C-02R-A277-07 | 0.413702389 | 175 | Low |
| TCGA-B6-A0RM-01A-11R-A084-07 | 0.415326009 | 176 | Low |
| TCGA-A8-A08C-01A-11R-A00Z-07 | 0.418567779 | 177 | Low |
| TCGA-BH-A1EX-01A-11R-A13Q-07 | 0.420832692 | 178 | Low |
| TCGA-AO-A0J6-01A-11R-A034-07 | 0.425029548 | 179 | Low |
| TCGA-A2-A0SW-01A-11R-A084-07 | 0.427069755 | 180 | Low |
| TCGA-A8-A079-01A-21R-A00Z-07 | 0.430071128 | 181 | Low |
| TCGA-A8-A06Q-01A-11R-A034-07 | 0.430392334 | 182 | Low |
| TCGA-E9-A249-01A-11R-A169-07 | 0.432638773 | 183 | Low |
| TCGA-AR-A24R-01A-11R-A169-07 | 0.434774991 | 184 | Low |
| TCGA-EW-A1OZ-01A-11R-A144-07 | 0.435095152 | 185 | Low |
| TCGA-B6-A0WZ-01A-11R-A109-07 | 0.435521921 | 186 | Low |
| TCGA-A2-A04V-01A-21R-A034-07 | 0.436055206 | 187 | Low |
| TCGA-AN-A0FK-01A-11R-A034-07 | 0.43839932 | 188 | Low |
| TCGA-C8-A12T-01A-11R-A115-07 | 0.43839932 | 189 | Low |
| TCGA-A8-A08I-01A-11R-A00Z-07 | 0.439037963 | 190 | Low |
| TCGA-D8-A1XF-01A-11R-A14D-07 | 0.439995398 | 191 | Low |
| TCGA-AN-A0AS-01A-11R-A00Z-07 | 0.440739631 | 192 | Low |
| TCGA-B6-A0IA-01A-11R-A034-07 | 0.445938537 | 193 | Low |
| TCGA-A8-A07L-01A-11R-A00Z-07 | 0.44794941 | 194 | Low |
| TCGA-D8-A1JD-01A-11R-A13Q-07 | 0.448372395 | 195 | Low |
| TCGA-AR-A24K-01A-11R-A169-07 | 0.448478121 | 196 | Low |
| TCGA-E9-A22D-01A-11R-A157-07 | 0.451540833 | 197 | Low |
| TCGA-E2-A1LA-01A-11R-A144-07 | 0.453333197 | 198 | Low |
| TCGA-E9-A1RF-01A-11R-A157-07 | 0.458906907 | 199 | Low |
| TCGA-AN-A0AM-01A-11R-A034-07 | 0.459431619 | 200 | Low |
| TCGA-AR-A2LK-01A-11R-A180-07 | 0.45964145 | 201 | Low |
| TCGA-E2-A1L9-01A-11R-A13Q-07 | 0.461319002 | 202 | Low |
| TCGA-PL-A8LV-01A-21R-A41B-07 | 0.462680579 | 203 | Low |
| TCGA-OL-A5RW-01A-11R-A28M-07 | 0.466235562 | 204 | Low |
| TCGA-A8-A08X-01A-21R-A00Z-07 | 0.467488174 | 205 | Low |
| TCGA-C8-A12O-01A-11R-A115-07 | 0.468531186 | 206 | Low |
| TCGA-A8-A09R-01A-11R-A00Z-07 | 0.468635446 | 207 | Low |
| TCGA-A1-A0SK-01A-12R-A084-07 | 0.470094293 | 208 | Low |
| TCGA-BH-A0E0-01A-11R-A056-07 | 0.471967788 | 209 | Low |
| TCGA-A2-A0EQ-01A-11R-A034-07 | 0.472903624 | 210 | Low |
| TCGA-AN-A0G0-01A-11R-A034-07 | 0.474773476 | 211 | Low |
| TCGA-AQ-A04H-01B-11R-A10J-07 | 0.47736648 | 212 | Low |
| TCGA-EW-A1P6-01A-11R-A144-07 | 0.481195593 | 213 | Low |
| TCGA-D8-A1Y3-01A-11R-A157-07 | 0.481712262 | 214 | Low |
| TCGA-BH-A0HB-01A-11R-A056-07 | 0.482332021 | 215 | Low |
| TCGA-A8-A07P-01A-11R-A00Z-07 | 0.482538548 | 216 | Low |
| TCGA-A8-A09D-01A-11R-A00Z-07 | 0.483364361 | 217 | Low |
| TCGA-D8-A1J9-01A-11R-A13Q-07 | 0.48357074 | 218 | Low |
| TCGA-E9-A54X-01A-11R-A266-07 | 0.483880254 | 219 | Low |
| TCGA-C8-A26V-01A-11R-A16F-07 | 0.484705299 | 220 | Low |
| TCGA-E2-A1LG-01A-21R-A14M-07 | 0.485426827 | 221 | Low |
| TCGA-AN-A0FT-01A-11R-A034-07 | 0.488720653 | 222 | Low |
| TCGA-D8-A140-01A-11R-A115-07 | 0.489542936 | 223 | Low |
| TCGA-A2-A0CS-01A-11R-A115-07 | 0.491493981 | 224 | Low |
| TCGA-OL-A5RZ-01A-11R-A28M-07 | 0.496206666 | 225 | Low |
| TCGA-AR-A0U4-01A-11R-A109-07 | 0.496820231 | 226 | Low |
| TCGA-BH-A0B8-01A-21R-A056-07 | 0.503246954 | 227 | Low |
| TCGA-E9-A2JS-01A-11R-A180-07 | 0.506906555 | 228 | Low |
| TCGA-C8-A273-01A-11R-A16F-07 | 0.509138416 | 229 | Low |
| TCGA-LL-A8F5-01A-11R-A36F-07 | 0.511366829 | 230 | Low |
| TCGA-A2-A3XV-01A-21R-A239-07 | 0.513490746 | 231 | Low |
| TCGA-E2-A1B0-01A-11R-A12P-07 | 0.513490746 | 232 | Low |
| TCGA-C8-A12W-01A-11R-A115-07 | 0.518736548 | 233 | Low |
| TCGA-A7-A26J-01A-11R-A277-07 | 0.519239949 | 234 | Low |
| TCGA-BH-A0AW-01A-11R-A056-07 | 0.522256668 | 235 | Low |
| TCGA-A7-A0CJ-01A-21R-A00Z-07 | 0.523060062 | 236 | Low |
| TCGA-D8-A27W-01A-11R-A16F-07 | 0.529371205 | 237 | Low |
| TCGA-A8-A092-01A-11R-A00Z-07 | 0.533164621 | 238 | Low |
| TCGA-E9-A1RD-01A-11R-A157-07 | 0.533264313 | 239 | Low |
| TCGA-E2-A1IH-01A-11R-A13Q-07 | 0.534161233 | 240 | Low |
| TCGA-A8-A09N-01A-11R-A00Z-07 | 0.545177635 | 241 | Low |
| TCGA-E2-A14V-01A-11R-A12D-07 | 0.548041987 | 242 | Low |
| TCGA-AR-A254-01A-21R-A169-07 | 0.549915554 | 243 | Low |
| TCGA-D8-A27N-01A-11R-A16F-07 | 0.550309679 | 244 | Low |
| TCGA-A2-A0SX-01A-12R-A084-07 | 0.550408193 | 245 | Low |
| TCGA-EW-A1J1-01A-11R-A13Q-07 | 0.550802184 | 246 | Low |
| TCGA-E9-A226-01A-21R-A157-07 | 0.555325359 | 247 | Low |
| TCGA-A8-A08G-01A-11R-A00Z-07 | 0.555619857 | 248 | Low |
| TCGA-AO-A0J7-01A-11R-A034-07 | 0.556797247 | 249 | Low |
| TCGA-B6-A0RL-01A-11R-A084-07 | 0.559051228 | 250 | Low |
| TCGA-JL-A3YW-01A-12R-A239-07 | 0.559442866 | 251 | Low |
| TCGA-BH-A203-01A-12R-A169-07 | 0.559638645 | 252 | Low |
| TCGA-D8-A13Y-01A-11R-A115-07 | 0.560812761 | 253 | Low |
| TCGA-E2-A15D-01A-11R-A115-07 | 0.560812761 | 254 | Low |
| TCGA-AO-A128-01A-11R-A10J-07 | 0.560910561 | 255 | Low |
| TCGA-A7-A26F-01B-04R-A22O-07 | 0.563060483 | 256 | Low |
| TCGA-BH-A0HU-01A-11R-A034-07 | 0.564914659 | 257 | Low |
| TCGA-B6-A0IB-01A-11R-A034-07 | 0.5663768 | 258 | Low |
| TCGA-3C-AAAU-01A-11R-A41B-07 | 0.567642792 | 259 | Low |
| TCGA-C8-A12N-01A-11R-A115-07 | 0.569685512 | 260 | Low |
| TCGA-EW-A1IZ-01A-11R-A13Q-07 | 0.575021826 | 261 | Low |
| TCGA-A8-A083-01A-21R-A00Z-07 | 0.577440913 | 262 | Low |
| TCGA-AC-A62Y-01A-11R-A29R-07 | 0.577537593 | 263 | Low |
| TCGA-E2-A15K-06A-11R-A12P-07 | 0.578600636 | 264 | Low |
| TCGA-A2-A0T1-01A-21R-A084-07 | 0.581977846 | 265 | Low |
| TCGA-AR-A0TY-01A-12R-A115-07 | 0.582941313 | 266 | Low |
| TCGA-A2-A0EU-01A-22R-A056-07 | 0.584866318 | 267 | Low |
| TCGA-E2-A15A-01A-11R-A12D-07 | 0.587268968 | 268 | Low |
| TCGA-E9-A22B-01A-11R-A157-07 | 0.590051039 | 269 | Low |
| TCGA-A2-A0ET-01A-31R-A034-07 | 0.592827755 | 270 | Low |
| TCGA-E9-A1N3-01A-12R-A157-07 | 0.599413018 | 271 | Low |
| TCGA-A8-A09G-01A-21R-A00Z-07 | 0.599603448 | 272 | Low |
| TCGA-EW-A1OV-01A-11R-A144-07 | 0.601125981 | 273 | Low |
| TCGA-E9-A1NF-01A-11R-A14D-07 | 0.60767356 | 274 | Low |
| TCGA-B6-A0RI-01A-11R-A056-07 | 0.614756952 | 275 | Low |
| TCGA-C8-A135-01A-11R-A115-07 | 0.614945367 | 276 | Low |
| TCGA-B6-A0IO-01A-11R-A034-07 | 0.623773238 | 277 | Low |
| TCGA-B6-A0IK-01A-12R-A056-07 | 0.6332917 | 278 | Low |
| TCGA-AO-A0JE-01A-11R-A056-07 | 0.635429371 | 279 | Low |
| TCGA-B6-A0IM-01A-11R-A034-07 | 0.639602631 | 280 | Low |
| TCGA-D8-A1JT-01A-31R-A13Q-07 | 0.640250721 | 281 | Low |
| TCGA-B6-A0RP-01A-21R-A084-07 | 0.641546029 | 282 | Low |
| TCGA-BH-A0HO-01A-11R-A034-07 | 0.641730978 | 283 | Low |
| TCGA-BH-A6R8-01A-21R-A33J-07 | 0.642655368 | 284 | Low |
| TCGA-AN-A04C-01A-21R-A034-07 | 0.645424989 | 285 | Low |
| TCGA-C8-A1HO-01A-11R-A13Q-07 | 0.648557478 | 286 | Low |
| TCGA-A2-A04Y-01A-21R-A034-07 | 0.650764559 | 287 | Low |
| TCGA-D8-A143-01A-11R-A115-07 | 0.652601218 | 288 | Low |
| TCGA-AN-A0FL-01A-11R-A034-07 | 0.655168618 | 289 | Low |
| TCGA-E2-A14T-01A-11R-A115-07 | 0.655626601 | 290 | Low |
| TCGA-A2-A0YC-01A-11R-A109-07 | 0.656359073 | 291 | Low |
| TCGA-3C-AALJ-01A-31R-A41B-07 | 0.656999681 | 292 | Low |
| TCGA-LL-A50Y-01A-11R-A266-07 | 0.659833246 | 293 | Low |
| TCGA-E9-A22A-01A-11R-A157-07 | 0.6617496 | 294 | Low |
| TCGA-E2-A15A-06A-11R-A12D-07 | 0.66475588 | 295 | Low |
| TCGA-BH-A0DZ-01A-11R-A00Z-07 | 0.665756583 | 296 | Low |
| TCGA-E9-A1R3-01A-31R-A14M-07 | 0.667483436 | 297 | Low |
| TCGA-E9-A1R4-01A-21R-A14D-07 | 0.671927385 | 298 | Low |
| TCGA-C8-A26Z-01A-11R-A16F-07 | 0.674279851 | 299 | Low |
| TCGA-A8-A06R-01A-11R-A00Z-07 | 0.678522677 | 300 | Low |
| TCGA-AN-A0FF-01A-11R-A034-07 | 0.678522677 | 301 | Low |
| TCGA-GM-A2DO-01A-11R-A18M-07 | 0.679694025 | 302 | Low |
| TCGA-AN-A03Y-01A-21R-A00Z-07 | 0.680054248 | 303 | Low |
| TCGA-BH-A18K-01A-11R-A12D-07 | 0.681674142 | 304 | Low |
| TCGA-AO-A03O-01A-11R-A00Z-07 | 0.683920981 | 305 | Low |
| TCGA-AR-A24X-01A-11R-A169-07 | 0.684998222 | 306 | Low |
| TCGA-E9-A1NI-01A-11R-A14D-07 | 0.687956494 | 307 | Low |
| TCGA-A7-A6VW-01A-21R-A33J-07 | 0.692070051 | 308 | Low |
| TCGA-A8-A06P-01A-11R-A00Z-07 | 0.69233792 | 309 | Low |
| TCGA-C8-A27B-01A-11R-A169-07 | 0.693765712 | 310 | Low |
| TCGA-A8-A07Z-01A-11R-A00Z-07 | 0.69777382 | 311 | Low |
| TCGA-B6-A0WT-01A-11R-A109-07 | 0.701327257 | 312 | Low |
| TCGA-BH-A209-01A-11R-A157-07 | 0.704075164 | 313 | Low |
| TCGA-3C-AALI-01A-11R-A41B-07 | 0.710966189 | 314 | Low |
| TCGA-A8-A06O-01A-11R-A00Z-07 | 0.713959698 | 315 | Low |
| TCGA-BH-A18L-01A-32R-A12D-07 | 0.714135594 | 316 | Low |
| TCGA-AR-A24S-01A-11R-A169-07 | 0.715454127 | 317 | Low |
| TCGA-EW-A1OY-01A-11R-A144-07 | 0.716771456 | 318 | Low |
| TCGA-BH-A18H-01A-11R-A12D-07 | 0.716859236 | 319 | Low |
| TCGA-B6-A0IN-01A-11R-A034-07 | 0.729444007 | 320 | Low |
| TCGA-BH-A1EY-01A-11R-A13Q-07 | 0.729531018 | 321 | Low |
| TCGA-BH-A0DH-01A-11R-A084-07 | 0.73135705 | 322 | Low |
| TCGA-AC-A5EH-01A-11R-A28M-07 | 0.732746762 | 323 | Low |
| TCGA-AC-A3QQ-01B-06R-A22O-07 | 0.735608823 | 324 | Low |
| TCGA-GM-A2DC-01A-11R-A18M-07 | 0.740539049 | 325 | Low |
| TCGA-E2-A2P5-01A-11R-A19W-07 | 0.742437445 | 326 | Low |
| TCGA-AC-A3W5-01A-11R-A22K-07 | 0.743040957 | 327 | Low |
| TCGA-A2-A0CX-01A-21R-A00Z-07 | 0.743299528 | 328 | Low |
| TCGA-AC-A23C-01A-12R-A169-07 | 0.744591687 | 329 | Low |
| TCGA-A8-A099-01A-11R-A00Z-07 | 0.750349241 | 330 | Low |
| TCGA-AC-A7VB-01A-11R-A352-07 | 0.751806465 | 331 | Low |
| TCGA-BH-A0HL-01A-11R-A10U-07 | 0.752834209 | 332 | Low |
| TCGA-BH-A0C0-01A-21R-A056-07 | 0.753176627 | 333 | Low |
| TCGA-A2-A0EV-01A-11R-A034-07 | 0.755058478 | 334 | Low |
| TCGA-A8-A0A1-01A-11R-A00Z-07 | 0.760519037 | 335 | Low |
| TCGA-A8-A07B-01A-11R-A00Z-07 | 0.764600936 | 336 | Low |
| TCGA-E9-A243-01A-21R-A169-07 | 0.764600936 | 337 | Low |
| TCGA-BH-A0HX-01A-21R-A056-07 | 0.765280131 | 338 | Low |
| TCGA-AN-A0XT-01A-11R-A109-07 | 0.765619608 | 339 | Low |
| TCGA-AC-A2BM-01A-11R-A21T-07 | 0.775176996 | 340 | Low |
| TCGA-A1-A0SN-01A-11R-A144-07 | 0.775682702 | 341 | Low |
| TCGA-PL-A8LZ-01A-31R-A36F-07 | 0.776861994 | 342 | Low |
| TCGA-B6-A0I8-01A-11R-A034-07 | 0.778040322 | 343 | Low |
| TCGA-C8-A3M8-01A-11R-A213-07 | 0.788936229 | 344 | Low |
| TCGA-B6-A0IG-01A-11R-A034-07 | 0.789186704 | 345 | Low |
| TCGA-A8-A09T-01A-11R-A00Z-07 | 0.793105148 | 346 | Low |
| TCGA-D8-A141-01A-11R-A115-07 | 0.795600729 | 347 | Low |
| TCGA-BH-A1FG-01A-11R-A13Q-07 | 0.796514695 | 348 | Low |
| TCGA-E2-A154-01A-11R-A115-07 | 0.801986364 | 349 | Low |
| TCGA-A7-A6VV-01A-22R-A33J-07 | 0.805375011 | 350 | Low |
| TCGA-E2-A15K-01A-11R-A12P-07 | 0.806942665 | 351 | Low |
| TCGA-E9-A3HO-01A-11R-A213-07 | 0.808838074 | 352 | Low |
| TCGA-BH-A0GZ-01A-11R-A056-07 | 0.810155153 | 353 | Low |
| TCGA-BH-A0BV-01A-11R-A00Z-07 | 0.814837498 | 354 | Low |
| TCGA-E9-A1RB-01A-11R-A157-07 | 0.816558749 | 355 | Low |
| TCGA-A2-A0D3-01A-11R-A115-07 | 0.817623258 | 356 | Low |
| TCGA-AR-A24L-01A-11R-A169-07 | 0.817868802 | 357 | Low |
| TCGA-A2-A0EY-01A-11R-A034-07 | 0.828550347 | 358 | Low |
| TCGA-BH-A0H7-01A-13R-A056-07 | 0.829768393 | 359 | Low |
| TCGA-D8-A1JM-01A-11R-A13Q-07 | 0.831796189 | 360 | Low |
| TCGA-A8-A07U-01A-11R-A034-07 | 0.83737816 | 361 | Low |
| TCGA-A2-A0CU-01A-12R-A034-07 | 0.840765337 | 362 | Low |
| TCGA-BH-A0HI-01A-11R-A084-07 | 0.841570637 | 363 | Low |
| TCGA-E9-A248-01A-11R-A169-07 | 0.843421123 | 364 | Low |
| TCGA-AN-A0AL-01A-11R-A00Z-07 | 0.843742705 | 365 | Low |
| TCGA-C8-A138-01A-11R-A115-07 | 0.844546345 | 366 | Low |
| TCGA-A2-A0CT-01A-31R-A056-07 | 0.846232534 | 367 | Low |
| TCGA-EW-A1IY-01A-11R-A13Q-07 | 0.850079296 | 368 | Low |
| TCGA-A2-A25C-01A-11R-A169-07 | 0.851679091 | 369 | Low |
| TCGA-E2-A14P-01A-31R-A12D-07 | 0.853277114 | 370 | Low |
| TCGA-E2-A15L-01A-11R-A12D-07 | 0.853596506 | 371 | Low |
| TCGA-E2-A15G-01A-11R-A12D-07 | 0.853756176 | 372 | Low |
| TCGA-E2-A15T-01A-11R-A115-07 | 0.855591108 | 373 | Low |
| TCGA-D8-A1XU-01A-11R-A14M-07 | 0.856866207 | 374 | Low |
| TCGA-A2-A0YF-01A-21R-A109-07 | 0.857423709 | 375 | Low |
| TCGA-A2-A25E-01A-11R-A169-07 | 0.857423709 | 376 | Low |
| TCGA-EW-A1P4-01A-21R-A144-07 | 0.85814018 | 377 | Low |
| TCGA-D8-A1XO-01A-11R-A14M-07 | 0.8623522 | 378 | Low |
| TCGA-B6-A0WW-01A-11R-A109-07 | 0.864017717 | 379 | Low |
| TCGA-EW-A6S9-01A-22R-A33J-07 | 0.865285394 | 380 | Low |
| TCGA-B6-A1KI-01A-11R-A14M-07 | 0.868844774 | 381 | Low |
| TCGA-A8-A06U-01A-11R-A00Z-07 | 0.869397667 | 382 | Low |
| TCGA-BH-A0EB-01A-11R-A034-07 | 0.869634556 | 383 | Low |
| TCGA-EW-A423-01A-11R-A24H-07 | 0.873734468 | 384 | Low |
| TCGA-EW-A1P5-01A-11R-A144-07 | 0.874049364 | 385 | Low |
| TCGA-E2-A15S-01A-11R-A115-07 | 0.880646348 | 386 | Low |
| TCGA-A2-A04P-01A-31R-A034-07 | 0.882056071 | 387 | Low |
| TCGA-AR-A24V-01A-21R-A169-07 | 0.886511129 | 388 | Low |
| TCGA-E2-A1L8-01A-11R-A13Q-07 | 0.88846077 | 389 | Low |
| TCGA-E2-A10B-01A-11R-A10J-07 | 0.889940737 | 390 | Low |
| TCGA-S3-A6ZG-01A-22R-A32P-07 | 0.89219671 | 391 | Low |
| TCGA-A8-A08T-01A-21R-A00Z-07 | 0.898634035 | 392 | Low |
| TCGA-C8-A1HK-01A-21R-A13Q-07 | 0.899098272 | 393 | Low |
| TCGA-A2-A1FX-01A-11R-A13Q-07 | 0.900103608 | 394 | Low |
| TCGA-E9-A22H-01A-11R-A157-07 | 0.906582788 | 395 | Low |
| TCGA-D8-A1XL-01A-11R-A14M-07 | 0.910272154 | 396 | Low |
| TCGA-EW-A1PC-01B-11R-A21T-07 | 0.913645804 | 397 | Low |
| TCGA-BH-A0H6-01A-21R-A056-07 | 0.914258349 | 398 | Low |
| TCGA-AO-A0JD-01A-11R-A056-07 | 0.919607047 | 399 | Low |
| TCGA-GM-A2DL-01A-11R-A18M-07 | 0.926606741 | 400 | Low |
| TCGA-AR-A0U3-01A-11R-A109-07 | 0.927289674 | 401 | Low |
| TCGA-AR-A1AT-01A-11R-A12P-07 | 0.929109248 | 402 | Low |
| TCGA-LL-A5YO-01A-21R-A28M-07 | 0.93319492 | 403 | Low |
| TCGA-EW-A1PE-01A-11R-A144-07 | 0.93561061 | 404 | Low |
| TCGA-A2-A04W-01A-31R-A115-07 | 0.937193711 | 405 | Low |
| TCGA-AN-A0XN-01A-21R-A109-07 | 0.937269054 | 406 | Low |
| TCGA-AR-A0TZ-01A-12R-A084-07 | 0.937495057 | 407 | Low |
| TCGA-E2-A1IN-01A-11R-A13Q-07 | 0.942232976 | 408 | Low |
| TCGA-A7-A6VY-01A-12R-A33J-07 | 0.947628757 | 409 | Low |
| TCGA-C8-A8HP-01A-11R-A36F-07 | 0.948301812 | 410 | Low |
| TCGA-AC-A23E-01A-11R-A157-07 | 0.951513196 | 411 | Low |
| TCGA-BH-A0BT-01A-11R-A12P-07 | 0.95173698 | 412 | Low |
| TCGA-AR-A24U-01A-11R-A169-07 | 0.952780844 | 413 | Low |
| TCGA-BH-A0W3-01A-11R-A109-07 | 0.954717448 | 414 | Low |
| TCGA-LL-A5YL-01A-12R-A29R-07 | 0.958954004 | 415 | Low |
| TCGA-E2-A1IE-01A-11R-A13Q-07 | 0.96036343 | 416 | Low |
| TCGA-V7-A7HQ-01A-11R-A33J-07 | 0.96303015 | 417 | Low |
| TCGA-BH-A0EI-01A-11R-A115-07 | 0.96413983 | 418 | Low |
| TCGA-BH-A0BR-01A-21R-A115-07 | 0.966430467 | 419 | Low |
| TCGA-A8-A06T-01A-11R-A00Z-07 | 0.966504298 | 420 | Low |
| TCGA-E9-A5UP-01A-11R-A28M-07 | 0.969528122 | 421 | Low |
| TCGA-A8-A093-01A-11R-A00Z-07 | 0.971368811 | 422 | Low |
| TCGA-A2-A04Q-01A-21R-A034-07 | 0.974309037 | 423 | Low |
| TCGA-AR-A1AW-01A-21R-A12P-07 | 0.975996958 | 424 | Low |
| TCGA-D8-A1Y1-01A-21R-A14M-07 | 0.976290308 | 425 | Low |
| TCGA-A8-A0AB-01A-11R-A034-07 | 0.977463111 | 426 | Low |
| TCGA-LL-A9Q3-01A-11R-A41B-07 | 0.977829417 | 427 | Low |
| TCGA-C8-A8HQ-01A-11R-A36F-07 | 0.987466402 | 428 | Low |
| TCGA-E2-A1IG-01A-11R-A144-07 | 0.989139007 | 429 | Low |
| TCGA-AR-A0U0-01A-11R-A109-07 | 0.989502361 | 430 | Low |
| TCGA-AN-A0XS-01A-22R-A109-07 | 0.991317757 | 431 | Low |
| TCGA-AC-A5XU-01A-11R-A28M-07 | 0.99146289 | 432 | Low |
| TCGA-E9-A1N5-01A-11R-A14D-07 | 0.999061943 | 433 | Low |
| TCGA-LL-A740-01A-21R-A32P-07 | 1.000072133 | 434 | Low |
| TCGA-E2-A106-01A-11R-A10J-07 | 1.012139555 | 435 | Low |
| TCGA-A8-A07F-01A-11R-A00Z-07 | 1.013712365 | 436 | Low |
| TCGA-A1-A0SG-01A-11R-A144-07 | 1.014283871 | 437 | Low |
| TCGA-E9-A1N4-01A-11R-A14M-07 | 1.015426204 | 438 | Low |
| TCGA-BH-A0BD-01A-11R-A034-07 | 1.025879273 | 439 | Low |
| TCGA-B6-A1KF-01A-11R-A13Q-07 | 1.026658438 | 440 | Low |
| TCGA-A2-A0D4-01A-11R-A00Z-07 | 1.03273563 | 441 | Low |
| TCGA-A8-A08Z-01A-21R-A00Z-07 | 1.038506418 | 442 | Low |
| TCGA-A7-A13F-01A-11R-A12P-07 | 1.039138394 | 443 | Low |
| TCGA-C8-A12Q-01A-11R-A115-07 | 1.040191072 | 444 | Low |
| TCGA-E2-A14X-01A-11R-A115-07 | 1.040191072 | 445 | Low |
| TCGA-A2-A0YH-01A-11R-A109-07 | 1.042854423 | 446 | Low |
| TCGA-S3-A6ZF-01A-32R-A32P-07 | 1.053597947 | 447 | Low |
| TCGA-LL-A6FQ-01A-11R-A31O-07 | 1.060669995 | 448 | Low |
| TCGA-E2-A1BC-01A-11R-A12P-07 | 1.064193062 | 449 | Low |
| TCGA-A2-A25D-01A-12R-A16F-07 | 1.073203272 | 450 | Low |
| TCGA-AR-A24P-01A-11R-A169-07 | 1.073888768 | 451 | Low |
| TCGA-B6-A0IP-01A-11R-A034-07 | 1.075395714 | 452 | Low |
| TCGA-AR-A2LL-01A-11R-A180-07 | 1.076285443 | 453 | Low |
| TCGA-A7-A0D9-01A-31R-A056-07 | 1.077174623 | 454 | Low |
| TCGA-A7-A26E-01A-11R-A277-07 | 1.080657663 | 455 | Low |
| TCGA-A2-A0YG-01A-21R-A109-07 | 1.080998686 | 456 | Low |
| TCGA-AN-A0FD-01A-11R-A034-07 | 1.085968454 | 457 | Low |
| TCGA-A8-A086-01A-11R-A00Z-07 | 1.086647913 | 458 | Low |
| TCGA-A7-A26G-01A-21R-A169-07 | 1.089430353 | 459 | Low |
| TCGA-A8-A07G-01A-11R-A034-07 | 1.092139768 | 460 | Low |
| TCGA-E2-A572-01A-13R-A31O-07 | 1.096801582 | 461 | Low |
| TCGA-A7-A6VX-01A-12R-A33J-07 | 1.100641316 | 462 | Low |
| TCGA-E9-A1NH-01A-11R-A14D-07 | 1.104269556 | 463 | Low |
| TCGA-BH-A42T-01A-11R-A24H-07 | 1.106415327 | 464 | Low |
| TCGA-C8-A1HM-01A-12R-A137-07 | 1.10701825 | 465 | Low |
| TCGA-A8-A09V-01A-11R-A034-07 | 1.108290261 | 466 | Low |
| TCGA-AO-A1KP-01A-11R-A13Q-07 | 1.108691716 | 467 | Low |
| TCGA-A8-A0A2-01A-11R-A034-07 | 1.108959291 | 468 | Low |
| TCGA-EW-A1PB-01A-11R-A144-07 | 1.110496882 | 469 | Low |
| TCGA-EW-A1J3-01A-11R-A13Q-07 | 1.113967146 | 470 | Low |
| TCGA-E9-A247-01A-11R-A169-07 | 1.118492626 | 471 | Low |
| TCGA-E2-A2P6-01A-11R-A19W-07 | 1.121347017 | 472 | Low |
| TCGA-BH-A1FR-01A-11R-A13Q-07 | 1.123202659 | 473 | Low |
| TCGA-OL-A66O-01A-11R-A31O-07 | 1.124526657 | 474 | Low |
| TCGA-BH-A0HY-01A-11R-A056-07 | 1.125651102 | 475 | Low |
| TCGA-S3-A6ZH-01A-22R-A32P-07 | 1.125849442 | 476 | Low |
| TCGA-E9-A1RA-01A-11R-A14D-07 | 1.132379425 | 477 | Low |
| TCGA-AN-A03X-01A-21R-A00Z-07 | 1.133826527 | 478 | Low |
| TCGA-BH-A1EO-01A-11R-A137-07 | 1.137110008 | 479 | Low |
| TCGA-A2-A1FW-01A-11R-A13Q-07 | 1.137831372 | 480 | Low |
| TCGA-A8-A07O-01A-11R-A00Z-07 | 1.144242197 | 481 | Low |
| TCGA-A8-A06Y-01A-21R-A00Z-07 | 1.148088083 | 482 | Low |
| TCGA-B6-A40B-01A-11R-A239-07 | 1.149259365 | 483 | Low |
| TCGA-AN-A0XW-01A-11R-A109-07 | 1.151014509 | 484 | Low |
| TCGA-GM-A2DB-01A-31R-A18M-07 | 1.155231116 | 485 | Low |
| TCGA-B6-A0WV-01A-11R-A109-07 | 1.162661211 | 486 | Low |
| TCGA-B6-A40C-01A-11R-A239-07 | 1.168064328 | 487 | Low |
| TCGA-B6-A0RG-01A-11R-A056-07 | 1.169155359 | 488 | Low |
| TCGA-BH-A0GY-01A-11R-A056-07 | 1.169219511 | 489 | Low |
| TCGA-A1-A0SI-01A-11R-A144-07 | 1.170437869 | 490 | Low |
| TCGA-GM-A2DH-01A-11R-A180-07 | 1.174789893 | 491 | Low |
| TCGA-E2-A14S-01A-11R-A12D-07 | 1.177279996 | 492 | Low |
| TCGA-B6-A409-01A-11R-A24H-07 | 1.177407578 | 493 | Low |
| TCGA-C8-A1HE-01A-11R-A13Q-07 | 1.185929959 | 494 | Low |
| TCGA-D8-A1XW-01A-11R-A14M-07 | 1.195599575 | 495 | Low |
| TCGA-A8-A090-01A-11R-A00Z-07 | 1.202323681 | 496 | Low |
| TCGA-AO-A0JL-01A-11R-A056-07 | 1.202386375 | 497 | Low |
| TCGA-AO-A0JC-01A-11R-A056-07 | 1.206268125 | 498 | Low |
| TCGA-Z7-A8R6-01A-11R-A41B-07 | 1.207018226 | 499 | Low |
| TCGA-B6-A2IU-01A-32R-A18M-07 | 1.20720569 | 500 | Low |
| TCGA-AR-A0TX-01A-11R-A084-07 | 1.207268173 | 501 | Low |
| TCGA-BH-A1FN-01A-11R-A13Q-07 | 1.208891775 | 502 | Low |
| TCGA-BH-A0H9-01A-11R-A056-07 | 1.211136827 | 503 | Low |
| TCGA-A2-A04N-01A-11R-A115-07 | 1.216672146 | 504 | Low |
| TCGA-A8-A09Z-01A-11R-A00Z-07 | 1.217354814 | 505 | Low |
| TCGA-BH-A18I-01A-11R-A12D-07 | 1.217789071 | 506 | Low |
| TCGA-E2-A107-01A-11R-A10J-07 | 1.221382181 | 507 | Low |
| TCGA-D8-A1XY-01A-11R-A14M-07 | 1.225151512 | 508 | Low |
| TCGA-A2-A0ER-01A-21R-A034-07 | 1.22527493 | 509 | Low |
| TCGA-BH-A0HN-01A-11R-A10U-07 | 1.231125158 | 510 | Low |
| TCGA-B6-A408-01A-12R-A24H-07 | 1.23155529 | 511 | Low |
| TCGA-E9-A228-01A-31R-A157-07 | 1.232722147 | 512 | Low |
| TCGA-A7-A0CH-01A-21R-A00Z-07 | 1.232783534 | 513 | Low |
| TCGA-BH-A6R9-01A-21R-A32P-07 | 1.243059706 | 514 | Low |
| TCGA-BH-A0HQ-01A-11R-A034-07 | 1.24895981 | 515 | Low |
| TCGA-BH-A18R-01A-11R-A12D-07 | 1.249627373 | 516 | Low |
| TCGA-AO-A0J9-01A-11R-A034-07 | 1.251385833 | 517 | Low |
| TCGA-B6-A0I9-01A-11R-A034-07 | 1.252900029 | 518 | Low |
| TCGA-B6-A0WS-01A-11R-A115-07 | 1.257674467 | 519 | Low |
| TCGA-A8-A07S-01A-11R-A034-07 | 1.259061529 | 520 | Low |
| TCGA-E2-A1B5-01A-21R-A12P-07 | 1.260146127 | 521 | Low |
| TCGA-AC-A3OD-01B-06R-A22O-07 | 1.262312878 | 522 | Low |
| TCGA-C8-A26W-01A-11R-A16F-07 | 1.262312878 | 523 | Low |
| TCGA-A8-A095-01A-11R-A00Z-07 | 1.264236151 | 524 | Low |
| TCGA-BH-A0BS-01A-11R-A12P-07 | 1.267056318 | 525 | Low |
| TCGA-B6-A0I1-01A-11R-A21T-07 | 1.267895303 | 526 | Low |
| TCGA-E2-A1LL-01A-11R-A144-07 | 1.270528942 | 527 | Low |
| TCGA-A2-A0CL-01A-11R-A115-07 | 1.271306144 | 528 | Low |
| TCGA-BH-A1F2-01A-31R-A13Q-07 | 1.276675317 | 529 | Low |
| TCGA-OL-A66P-01A-11R-A31O-07 | 1.285816472 | 530 | Low |
| TCGA-E9-A229-01A-31R-A157-07 | 1.285934808 | 531 | Low |
| TCGA-BH-A0E2-01A-11R-A056-07 | 1.286171451 | 532 | Low |
| TCGA-BH-A1ES-01A-11R-A137-07 | 1.289067184 | 533 | Low |
| TCGA-E2-A15C-01A-31R-A12D-07 | 1.290247448 | 534 | Low |
| TCGA-E2-A10F-01A-11R-A10J-07 | 1.290483384 | 535 | Low |
| TCGA-B6-A1KN-01A-11R-A13Q-07 | 1.292781749 | 536 | Low |
| TCGA-LL-A7SZ-01A-32R-A352-07 | 1.292840634 | 537 | Low |
| TCGA-D8-A27R-01A-11R-A16F-07 | 1.293429344 | 538 | Low |
| TCGA-E2-A15R-01A-11R-A115-07 | 1.299244658 | 539 | Low |
| TCGA-D8-A27T-01A-11R-A16F-07 | 1.299889359 | 540 | Low |
| TCGA-BH-A5J0-01A-11R-A27Q-07 | 1.302114295 | 541 | Low |
| TCGA-BH-A0DS-01A-11R-A056-07 | 1.303693088 | 542 | Low |
| TCGA-BH-A0BW-01A-11R-A115-07 | 1.305562016 | 543 | Low |
| TCGA-AC-A2FM-01A-11R-A19W-07 | 1.307603387 | 544 | High |
| TCGA-BH-A18P-01A-11R-A12D-07 | 1.312374747 | 545 | High |
| TCGA-BH-A0AU-01A-11R-A12P-07 | 1.314696526 | 546 | High |
| TCGA-BH-A0W7-01A-11R-A115-07 | 1.318056482 | 547 | High |
| TCGA-A7-A5ZV-01A-11R-A28M-07 | 1.32313945 | 548 | High |
| TCGA-E2-A150-01A-11R-A12D-07 | 1.324465006 | 549 | High |
| TCGA-AN-A0XO-01A-11R-A109-07 | 1.327457434 | 550 | High |
| TCGA-A2-A0EM-01A-11R-A034-07 | 1.327859788 | 551 | High |
| TCGA-LL-A5YN-01A-11R-A28M-07 | 1.329238433 | 552 | High |
| TCGA-B6-A0RO-01A-22R-A084-07 | 1.329525485 | 553 | High |
| TCGA-A7-A0DB-01A-11R-A277-07 | 1.334396652 | 554 | High |
| TCGA-BH-A0AY-01A-21R-A00Z-07 | 1.334568276 | 555 | High |
| TCGA-BH-A0B9-01A-11R-A056-07 | 1.33845294 | 556 | High |
| TCGA-AR-A0TQ-01A-11R-A084-07 | 1.342099565 | 557 | High |
| TCGA-E2-A1LE-01A-12R-A19W-07 | 1.345680231 | 558 | High |
| TCGA-AN-A0FY-01A-11R-A034-07 | 1.349138777 | 559 | High |
| TCGA-A2-A0EN-01A-13R-A084-07 | 1.35935249 | 560 | High |
| TCGA-A2-A0CM-01A-31R-A034-07 | 1.364348307 | 561 | High |
| TCGA-WT-AB41-01A-11R-A41B-07 | 1.365916454 | 562 | High |
| TCGA-E9-A1RI-01A-11R-A169-07 | 1.368600747 | 563 | High |
| TCGA-BH-A0DQ-01A-11R-A084-07 | 1.368768349 | 564 | High |
| TCGA-D8-A13Z-01A-11R-A115-07 | 1.377012662 | 565 | High |
| TCGA-E9-A1N9-01A-11R-A14D-07 | 1.379177329 | 566 | High |
| TCGA-AO-A1KT-01A-11R-A13Q-07 | 1.379731849 | 567 | High |
| TCGA-E2-A576-01A-11R-A31O-07 | 1.382611923 | 568 | High |
| TCGA-LQ-A4E4-01A-11R-A266-07 | 1.383441646 | 569 | High |
| TCGA-E2-A10E-01A-21R-A10J-07 | 1.389676937 | 570 | High |
| TCGA-OL-A5D6-01A-21R-A27Q-07 | 1.406155674 | 571 | High |
| TCGA-E2-A15H-01A-11R-A12D-07 | 1.408711861 | 572 | High |
| TCGA-D8-A73U-01A-11R-A33J-07 | 1.411751629 | 573 | High |
| TCGA-D8-A73X-01A-11R-A32P-07 | 1.415974946 | 574 | High |
| TCGA-A1-A0SQ-01A-21R-A144-07 | 1.425459305 | 575 | High |
| TCGA-BH-A1EV-01A-11R-A137-07 | 1.426157387 | 576 | High |
| TCGA-AO-A125-01A-11R-A10J-07 | 1.432959407 | 577 | High |
| TCGA-D8-A27G-01A-11R-A16F-07 | 1.439782689 | 578 | High |
| TCGA-AO-A1KS-01A-11R-A13Q-07 | 1.442704685 | 579 | High |
| TCGA-BH-A1ET-01A-11R-A137-07 | 1.446573853 | 580 | High |
| TCGA-AO-A126-01A-11R-A10J-07 | 1.454070583 | 581 | High |
| TCGA-BH-A1EN-01A-11R-A13Q-07 | 1.458487 | 582 | High |
| TCGA-AC-A3TN-01A-11R-A22K-07 | 1.458801942 | 583 | High |
| TCGA-AO-A0JI-01A-21R-A056-07 | 1.468061924 | 584 | High |
| TCGA-A2-A0ST-01A-12R-A084-07 | 1.474046599 | 585 | High |
| TCGA-BH-A18V-06A-11R-A213-07 | 1.475084883 | 586 | High |
| TCGA-D8-A1XD-01A-11R-A14D-07 | 1.48769214 | 587 | High |
| TCGA-A7-A26E-01B-06R-A277-07 | 1.488155062 | 588 | High |
| TCGA-AN-A0FW-01A-11R-A034-07 | 1.491493981 | 589 | High |
| TCGA-A7-A4SD-01A-11R-A266-07 | 1.496513481 | 590 | High |
| TCGA-EW-A1PA-01A-11R-A144-07 | 1.497740089 | 591 | High |
| TCGA-B6-A0X4-01A-11R-A109-07 | 1.504620392 | 592 | High |
| TCGA-B6-A0RN-01A-12R-A084-07 | 1.504823754 | 593 | High |
| TCGA-A2-A0T6-01A-11R-A084-07 | 1.51772922 | 594 | High |
| TCGA-E2-A1L7-01A-11R-A144-07 | 1.519189617 | 595 | High |
| TCGA-BH-A0H0-01A-11R-A056-07 | 1.521101004 | 596 | High |
| TCGA-AR-A1AY-01A-21R-A12P-07 | 1.522608208 | 597 | High |
| TCGA-A2-A0T3-01A-21R-A115-07 | 1.52371249 | 598 | High |
| TCGA-AR-A2LE-01A-11R-A180-07 | 1.525467563 | 599 | High |
| TCGA-AN-A0XU-01A-11R-A109-07 | 1.527670916 | 600 | High |
| TCGA-E2-A105-01A-11R-A10J-07 | 1.533762671 | 601 | High |
| TCGA-EW-A1P8-01A-11R-A144-07 | 1.534260856 | 602 | High |
| TCGA-AN-A0FN-01A-11R-A034-07 | 1.534509884 | 603 | High |
| TCGA-E9-A3QA-01A-61R-A22K-07 | 1.53465928 | 604 | High |
| TCGA-OL-A5D8-01A-11R-A27Q-07 | 1.535206934 | 605 | High |
| TCGA-A7-A26J-01A-11R-A169-07 | 1.554195894 | 606 | High |
| TCGA-EW-A1J5-01A-11R-A13Q-07 | 1.555963361 | 607 | High |
| TCGA-AR-A1AR-01A-31R-A137-07 | 1.558659484 | 608 | High |
| TCGA-B6-A0I5-01A-11R-A034-07 | 1.562230211 | 609 | High |
| TCGA-EW-A1IX-01A-12R-A144-07 | 1.566571641 | 610 | High |
| TCGA-E9-A6HE-01A-11R-A31O-07 | 1.570317197 | 611 | High |
| TCGA-BH-A0BG-01A-11R-A115-07 | 1.573374526 | 612 | High |
| TCGA-BH-A18J-01A-11R-A12D-07 | 1.583278374 | 613 | High |
| TCGA-A8-A08O-01A-21R-A056-07 | 1.587701021 | 614 | High |
| TCGA-AC-A2QJ-01A-12R-A19W-07 | 1.590290621 | 615 | High |
| TCGA-A8-A07J-01A-11R-A00Z-07 | 1.599984232 | 616 | High |
| TCGA-AR-A5QQ-01A-11R-A28M-07 | 1.607957565 | 617 | High |
| TCGA-A2-A1FV-01A-11R-A13Q-07 | 1.614945367 | 618 | High |
| TCGA-BH-A0RX-01A-21R-A084-07 | 1.618426617 | 619 | High |
| TCGA-AR-A24M-01A-11R-A169-07 | 1.624147697 | 620 | High |
| TCGA-A7-A13G-01B-04R-A22O-07 | 1.630591854 | 621 | High |
| TCGA-GM-A2DA-01A-11R-A18M-07 | 1.63705374 | 622 | High |
| TCGA-A8-A0A7-01A-11R-A00Z-07 | 1.639556328 | 623 | High |
| TCGA-PE-A5DD-01A-12R-A27Q-07 | 1.644087003 | 624 | High |
| TCGA-C8-A12Y-01A-11R-A12D-07 | 1.652325868 | 625 | High |
| TCGA-A7-A2KD-01A-31R-A21T-07 | 1.652463549 | 626 | High |
| TCGA-A8-A09A-01A-11R-A00Z-07 | 1.656359073 | 627 | High |
| TCGA-AC-A4ZE-01A-11R-A41B-07 | 1.660381035 | 628 | High |
| TCGA-BH-A0DE-01A-11R-A115-07 | 1.663390165 | 629 | High |
| TCGA-E2-A1IL-01A-11R-A14D-07 | 1.663663412 | 630 | High |
| TCGA-GM-A2D9-01A-11R-A18M-07 | 1.670341831 | 631 | High |
| TCGA-AC-A3EH-01A-22R-A22K-07 | 1.680324357 | 632 | High |
| TCGA-AR-A252-01A-11R-A169-07 | 1.682483407 | 633 | High |
| TCGA-EW-A1PF-01A-11R-A144-07 | 1.683202373 | 634 | High |
| TCGA-E2-A570-01A-11R-A29R-07 | 1.687508661 | 635 | High |
| TCGA-AR-A24N-01A-11R-A169-07 | 1.695370292 | 636 | High |
| TCGA-E9-A245-01A-22R-A16F-07 | 1.698085095 | 637 | High |
| TCGA-EW-A1P3-01A-11R-A144-07 | 1.699906932 | 638 | High |
| TCGA-D8-A1JC-01A-11R-A13Q-07 | 1.702036896 | 639 | High |
| TCGA-A2-A0CY-01A-12R-A034-07 | 1.702480243 | 640 | High |
| TCGA-AC-A2B8-01A-11R-A17B-07 | 1.708010614 | 641 | High |
| TCGA-A2-A04R-01A-41R-A109-07 | 1.717517409 | 642 | High |
| TCGA-E2-A15M-01A-11R-A12D-07 | 1.726831217 | 643 | High |
| TCGA-A2-A3XZ-01A-42R-A239-07 | 1.735868729 | 644 | High |
| TCGA-BH-A28Q-01A-11R-A16F-07 | 1.73760024 | 645 | High |
| TCGA-BH-A0HA-01A-11R-A12P-07 | 1.746957642 | 646 | High |
| TCGA-AC-A6NO-01A-12R-A33J-07 | 1.747945893 | 647 | High |
| TCGA-E2-A14Z-01A-11R-A115-07 | 1.751120896 | 648 | High |
| TCGA-A2-A0YI-01A-31R-A10J-07 | 1.757108611 | 649 | High |
| TCGA-LL-A7T0-01A-31R-A352-07 | 1.757279323 | 650 | High |
| TCGA-AO-A03V-01A-11R-A115-07 | 1.761072471 | 651 | High |
| TCGA-E9-A2JT-01A-22R-A18M-07 | 1.761838413 | 652 | High |
| TCGA-D8-A1XM-01A-21R-A14M-07 | 1.763581543 | 653 | High |
| TCGA-D8-A1XA-01A-11R-A14D-07 | 1.763921421 | 654 | High |
| TCGA-A2-A4S3-01A-21R-A266-07 | 1.764940573 | 655 | High |
| TCGA-A7-A0DB-01A-11R-A00Z-07 | 1.769814046 | 656 | High |
| TCGA-A7-A26J-01B-02R-A277-07 | 1.776651476 | 657 | High |
| TCGA-A8-A0A6-01A-12R-A056-07 | 1.784294578 | 658 | High |
| TCGA-AN-A04A-01A-21R-A034-07 | 1.794020696 | 659 | High |
| TCGA-BH-A1FL-01A-11R-A13Q-07 | 1.818809668 | 660 | High |
| TCGA-A7-A3IY-01A-21R-A21T-07 | 1.820485343 | 661 | High |
| TCGA-E9-A227-01A-11R-A157-07 | 1.8246864 | 662 | High |
| TCGA-BH-A0HK-01A-11R-A056-07 | 1.825622832 | 663 | High |
| TCGA-D8-A1XB-01A-11R-A14D-07 | 1.828631582 | 664 | High |
| TCGA-AC-A2QH-01B-04R-A22O-07 | 1.837701091 | 665 | High |
| TCGA-BH-A0W5-01A-11R-A109-07 | 1.837741452 | 666 | High |
| TCGA-AN-A049-01A-21R-A00Z-07 | 1.845510123 | 667 | High |
| TCGA-GM-A2DD-01A-11R-A180-07 | 1.847596102 | 668 | High |
| TCGA-AO-A03L-01A-41R-A056-07 | 1.852118724 | 669 | High |
| TCGA-E2-A1B4-01A-11R-A12P-07 | 1.859055154 | 670 | High |
| TCGA-A2-A0T4-01A-31R-A084-07 | 1.862550576 | 671 | High |
| TCGA-E2-A1IU-01A-11R-A14D-07 | 1.862629919 | 672 | High |
| TCGA-A7-A3IZ-01A-11R-A213-07 | 1.863779904 | 673 | High |
| TCGA-BH-A0BJ-01A-11R-A056-07 | 1.864136609 | 674 | High |
| TCGA-D8-A27L-01A-11R-A16F-07 | 1.864295117 | 675 | High |
| TCGA-A2-A1G4-01A-11R-A13Q-07 | 1.868963269 | 676 | High |
| TCGA-B6-A0RH-01A-21R-A115-07 | 1.87034499 | 677 | High |
| TCGA-AO-A12G-01A-11R-A10J-07 | 1.876173114 | 678 | High |
| TCGA-A7-A0DC-01B-04R-A22O-07 | 1.885535319 | 679 | High |
| TCGA-E2-A1BD-01A-11R-A12P-07 | 1.88631602 | 680 | High |
| TCGA-EW-A6SB-01A-12R-A32P-07 | 1.889006197 | 681 | High |
| TCGA-E2-A15O-01A-11R-A115-07 | 1.892118977 | 682 | High |
| TCGA-BH-A0BF-01A-21R-A12P-07 | 1.902652471 | 683 | High |
| TCGA-E9-A1R2-01A-11R-A14D-07 | 1.90931229 | 684 | High |
| TCGA-BH-A0B0-01A-21R-A115-07 | 1.911269736 | 685 | High |
| TCGA-E2-A1AZ-01A-11R-A12P-07 | 1.91827173 | 686 | High |
| TCGA-D8-A1JH-01A-11R-A13Q-07 | 1.925353858 | 687 | High |
| TCGA-D8-A1XZ-01A-11R-A14M-07 | 1.935497465 | 688 | High |
| TCGA-E2-A14Y-01A-21R-A12D-07 | 1.941895069 | 689 | High |
| TCGA-B6-A0RV-01A-11R-A084-07 | 1.949796369 | 690 | High |
| TCGA-B6-A1KC-01B-11R-A157-07 | 1.950319098 | 691 | High |
| TCGA-E9-A295-01A-11R-A16F-07 | 1.955163988 | 692 | High |
| TCGA-C8-A130-01A-31R-A115-07 | 1.956874444 | 693 | High |
| TCGA-E2-A1II-01A-11R-A144-07 | 1.957617488 | 694 | High |
| TCGA-A1-A0SD-01A-11R-A115-07 | 1.959584707 | 695 | High |
| TCGA-AR-A255-01A-11R-A169-07 | 1.963400138 | 696 | High |
| TCGA-A2-A0T0-01A-22R-A084-07 | 1.976473621 | 697 | High |
| TCGA-A8-A097-01A-11R-A034-07 | 1.979879012 | 698 | High |
| TCGA-BH-A0BM-01A-11R-A056-07 | 1.989211685 | 699 | High |
| TCGA-AN-A0AR-01A-11R-A00Z-07 | 2.004968732 | 700 | High |
| TCGA-AC-A8OQ-01A-11R-A41B-07 | 2.01210379 | 701 | High |
| TCGA-AC-A6IX-06A-11R-A32P-07 | 2.01346226 | 702 | High |
| TCGA-EW-A6SC-01A-12R-A32P-07 | 2.016959791 | 703 | High |
| TCGA-AN-A04D-01A-21R-A034-07 | 2.021302045 | 704 | High |
| TCGA-AC-A2QI-01A-12R-A19W-07 | 2.025489533 | 705 | High |
| TCGA-A7-A13D-01A-13R-A277-07 | 2.029099457 | 706 | High |
| TCGA-BH-A0C7-01B-11R-A115-07 | 2.032665112 | 707 | High |
| TCGA-A2-A0SU-01A-11R-A084-07 | 2.038541535 | 708 | High |
| TCGA-E2-A15F-01A-11R-A115-07 | 2.039770093 | 709 | High |
| TCGA-AC-A8OP-01A-11R-A36F-07 | 2.044673888 | 710 | High |
| TCGA-D8-A1JP-01A-11R-A13Q-07 | 2.04851521 | 711 | High |
| TCGA-A2-A0EO-01A-11R-A034-07 | 2.053285145 | 712 | High |
| TCGA-BH-A0C1-01B-11R-A12D-07 | 2.061534288 | 713 | High |
| TCGA-A7-A13E-01B-06R-A277-07 | 2.077653186 | 714 | High |
| TCGA-E2-A1B6-01A-31R-A12P-07 | 2.083213368 | 715 | High |
| TCGA-E9-A1R5-01A-11R-A14M-07 | 2.096363068 | 716 | High |
| TCGA-S3-AA17-01A-11R-A41B-07 | 2.120152843 | 717 | High |
| TCGA-5T-A9QA-01A-11R-A41B-07 | 2.12498977 | 718 | High |
| TCGA-A1-A0SH-01A-11R-A084-07 | 2.129975344 | 719 | High |
| TCGA-BH-A0DG-01A-21R-A12P-07 | 2.133695033 | 720 | High |
| TCGA-AO-A0JA-01A-11R-A056-07 | 2.139436751 | 721 | High |
| TCGA-5L-AAT1-01A-12R-A41B-07 | 2.14238076 | 722 | High |
| TCGA-GM-A3NY-01A-11R-A21T-07 | 2.154226736 | 723 | High |
| TCGA-PE-A5DE-01A-11R-A27Q-07 | 2.156493699 | 724 | High |
| TCGA-AO-A0JB-01A-11R-A32Y-07 | 2.170662191 | 725 | High |
| TCGA-D8-A27P-01A-11R-A16F-07 | 2.173831016 | 726 | High |
| TCGA-E9-A1ND-01A-11R-A144-07 | 2.183994584 | 727 | High |
| TCGA-B6-A0X0-01A-21R-A115-07 | 2.188084371 | 728 | High |
| TCGA-E2-A15P-01A-11R-A115-07 | 2.191752134 | 729 | High |
| TCGA-A7-A13D-01B-04R-A277-07 | 2.192257302 | 730 | High |
| TCGA-A2-A4S1-01A-21R-A266-07 | 2.193614063 | 731 | High |
| TCGA-OL-A66H-01A-11R-A29R-07 | 2.205392513 | 732 | High |
| TCGA-GM-A2DN-01A-11R-A180-07 | 2.212693838 | 733 | High |
| TCGA-E9-A1R0-01A-22R-A16F-07 | 2.219184012 | 734 | High |
| TCGA-E2-A1IK-01A-11R-A144-07 | 2.219369903 | 735 | High |
| TCGA-C8-A3M7-01A-12R-A21T-07 | 2.229003344 | 736 | High |
| TCGA-S3-AA15-01A-11R-A41B-07 | 2.244004127 | 737 | High |
| TCGA-BH-A1FE-06A-11R-A213-07 | 2.245465239 | 738 | High |
| TCGA-AR-A1AN-01A-11R-A12P-07 | 2.246620903 | 739 | High |
| TCGA-AN-A0FJ-01A-11R-A00Z-07 | 2.253656531 | 740 | High |
| TCGA-OL-A5RU-01A-11R-A28M-07 | 2.274231838 | 741 | High |
| TCGA-BH-A0BP-01A-11R-A115-07 | 2.290335928 | 742 | High |
| TCGA-AN-A046-01A-21R-A034-07 | 2.291927657 | 743 | High |
| TCGA-A2-A0YD-01A-11R-A109-07 | 2.294841271 | 744 | High |
| TCGA-A7-A4SA-01A-11R-A266-07 | 2.296721694 | 745 | High |
| TCGA-D8-A3Z6-01A-11R-A239-07 | 2.297866375 | 746 | High |
| TCGA-E2-A15I-01A-21R-A137-07 | 2.301382787 | 747 | High |
| TCGA-A7-A26E-01A-11R-A169-07 | 2.312868438 | 748 | High |
| TCGA-B6-A0WX-01A-11R-A109-07 | 2.313187794 | 749 | High |
| TCGA-BH-A0B5-01A-11R-A12P-07 | 2.316580224 | 750 | High |
| TCGA-C8-A12K-01A-21R-A115-07 | 2.317130379 | 751 | High |
| TCGA-BH-A201-01A-11R-A14M-07 | 2.321004474 | 752 | High |
| TCGA-GM-A5PX-01A-12R-A28M-07 | 2.333967504 | 753 | High |
| TCGA-B6-A0RT-01A-21R-A084-07 | 2.336711848 | 754 | High |
| TCGA-AQ-A1H3-01A-31R-A13Q-07 | 2.337054524 | 755 | High |
| TCGA-A2-A259-01A-11R-A16F-07 | 2.338681125 | 756 | High |
| TCGA-AO-A0JF-01A-11R-A056-07 | 2.346446362 | 757 | High |
| TCGA-A8-A09B-01A-11R-A00Z-07 | 2.347353842 | 758 | High |
| TCGA-AR-A5QM-01A-11R-A27Q-07 | 2.348912241 | 759 | High |
| TCGA-A2-A3KD-01A-12R-A213-07 | 2.349931373 | 760 | High |
| TCGA-D8-A27K-01A-11R-A16F-07 | 2.35199574 | 761 | High |
| TCGA-AC-A2BK-01A-11R-A21T-07 | 2.352589052 | 762 | High |
| TCGA-GM-A2DK-01A-21R-A180-07 | 2.353690271 | 763 | High |
| TCGA-E9-A1QZ-01A-21R-A169-07 | 2.355072662 | 764 | High |
| TCGA-WT-AB44-01A-11R-A41B-07 | 2.36577651 | 765 | High |
| TCGA-LD-A9QF-01A-32R-A41B-07 | 2.37114063 | 766 | High |
| TCGA-AO-A0JG-01A-31R-A084-07 | 2.380951044 | 767 | High |
| TCGA-A2-A0D0-01A-11R-A00Z-07 | 2.39196014 | 768 | High |
| TCGA-E2-A1IJ-01A-11R-A144-07 | 2.398240905 | 769 | High |
| TCGA-AO-A12C-01A-11R-A10J-07 | 2.401439332 | 770 | High |
| TCGA-A8-A0A4-01A-11R-A00Z-07 | 2.417704019 | 771 | High |
| TCGA-A1-A0SJ-01A-11R-A084-07 | 2.41773102 | 772 | High |
| TCGA-E9-A5FK-01A-11R-A27Q-07 | 2.420563246 | 773 | High |
| TCGA-OL-A66K-01A-11R-A29R-07 | 2.430900763 | 774 | High |
| TCGA-B6-A0IJ-01A-11R-A034-07 | 2.434401381 | 775 | High |
| TCGA-BH-A2L8-01A-11R-A18M-07 | 2.440420721 | 776 | High |
| TCGA-BH-A0E1-01A-11R-A056-07 | 2.45785691 | 777 | High |
| TCGA-A2-A25F-01A-11R-A169-07 | 2.459667677 | 778 | High |
| TCGA-BH-A0BC-01A-22R-A084-07 | 2.461030812 | 779 | High |
| TCGA-PE-A5DC-01A-12R-A27Q-07 | 2.461659517 | 780 | High |
| TCGA-GI-A2C9-01A-11R-A21T-07 | 2.462837601 | 781 | High |
| TCGA-AC-A62X-01A-11R-A29R-07 | 2.46296844 | 782 | High |
| TCGA-E2-A1IF-01A-11R-A144-07 | 2.467801156 | 783 | High |
| TCGA-A2-A0EP-01A-52R-A22U-07 | 2.476770502 | 784 | High |
| TCGA-BH-A0DP-01A-21R-A056-07 | 2.482022175 | 785 | High |
| TCGA-OL-A66J-01A-11R-A29R-07 | 2.486971744 | 786 | High |
| TCGA-BH-A0DK-01A-21R-A056-07 | 2.49067281 | 787 | High |
| TCGA-A8-A07E-01A-11R-A034-07 | 2.495362588 | 788 | High |
| TCGA-BH-A8FY-01A-11R-A36F-07 | 2.496411216 | 789 | High |
| TCGA-GM-A3XL-01A-11R-A22U-07 | 2.49646235 | 790 | High |
| TCGA-E2-A1LS-01A-12R-A157-07 | 2.512024566 | 791 | High |
| TCGA-A2-A0CR-01A-11R-A22K-07 | 2.514602026 | 792 | High |
| TCGA-C8-A132-01A-31R-A115-07 | 2.517628449 | 793 | High |
| TCGA-AR-A1AP-01A-11R-A12P-07 | 2.518535139 | 794 | High |
| TCGA-LL-A441-01A-11R-A24H-07 | 2.520522825 | 795 | High |
| TCGA-AO-A03U-01B-21R-A10J-07 | 2.527270557 | 796 | High |
| TCGA-B6-A0X1-01A-11R-A109-07 | 2.528446297 | 797 | High |
| TCGA-5L-AAT0-01A-12R-A41B-07 | 2.545424786 | 798 | High |
| TCGA-BH-A1EW-01A-11R-A137-07 | 2.547647242 | 799 | High |
| TCGA-E2-A1B1-01A-21R-A12P-07 | 2.547721265 | 800 | High |
| TCGA-BH-A0B1-01A-12R-A056-07 | 2.547745939 | 801 | High |
| TCGA-S3-AA11-01A-31R-A41B-07 | 2.552278691 | 802 | High |
| TCGA-EW-A1IW-01A-11R-A13Q-07 | 2.567691462 | 803 | High |
| TCGA-EW-A3E8-01B-11R-A24H-07 | 2.570025684 | 804 | High |
| TCGA-BH-A208-01A-11R-A157-07 | 2.584986545 | 805 | High |
| TCGA-A7-A4SB-01A-21R-A266-07 | 2.585587533 | 806 | High |
| TCGA-BH-A0HF-01A-11R-A056-07 | 2.610416597 | 807 | High |
| TCGA-B6-A0IE-01A-11R-A034-07 | 2.614592068 | 808 | High |
| TCGA-A2-A3XW-01A-11R-A239-07 | 2.622696128 | 809 | High |
| TCGA-BH-A1FU-01A-11R-A14D-07 | 2.623820051 | 810 | High |
| TCGA-AR-A1AI-01A-11R-A12P-07 | 2.636960969 | 811 | High |
| TCGA-BH-AB28-01A-31R-A41B-07 | 2.638467772 | 812 | High |
| TCGA-AR-A1AO-01A-11R-A12P-07 | 2.640528385 | 813 | High |
| TCGA-AO-A03M-01B-11R-A10J-07 | 2.643417544 | 814 | High |
| TCGA-BH-A1FE-01A-11R-A13Q-07 | 2.645747765 | 815 | High |
| TCGA-AR-A24T-01A-11R-A169-07 | 2.649362534 | 816 | High |
| TCGA-4H-AAAK-01A-12R-A41B-07 | 2.656450606 | 817 | High |
| TCGA-A1-A0SE-01A-11R-A084-07 | 2.667142774 | 818 | High |
| TCGA-A2-A0YE-01A-11R-A109-07 | 2.677914102 | 819 | High |
| TCGA-A7-A26I-01B-06R-A22O-07 | 2.697529199 | 820 | High |
| TCGA-B6-A0RS-01A-11R-A084-07 | 2.702413749 | 821 | High |
| TCGA-AR-A1AJ-01A-21R-A12P-07 | 2.710349106 | 822 | High |
| TCGA-A7-A13D-01A-13R-A12P-07 | 2.711076354 | 823 | High |
| TCGA-EW-A2FS-01A-11R-A17B-07 | 2.716968952 | 824 | High |
| TCGA-BH-A18F-01A-11R-A12D-07 | 2.717517409 | 825 | High |
| TCGA-AR-A1AX-01A-11R-A12P-07 | 2.724126354 | 826 | High |
| TCGA-BH-A0AZ-01A-21R-A12P-07 | 2.729879012 | 827 | High |
| TCGA-AC-A3HN-01A-11R-A213-07 | 2.746441765 | 828 | High |
| TCGA-BH-A18M-01A-11R-A12D-07 | 2.750670814 | 829 | High |
| TCGA-LL-A73Z-01A-11R-A32P-07 | 2.770152459 | 830 | High |
| TCGA-GM-A2DI-01A-31R-A18M-07 | 2.77089246 | 831 | High |
| TCGA-AR-A24O-01A-11R-A169-07 | 2.77216015 | 832 | High |
| TCGA-AC-A5XS-01A-11R-A29R-07 | 2.780667054 | 833 | High |
| TCGA-MS-A51U-01A-31R-A266-07 | 2.785027362 | 834 | High |
| TCGA-AO-A0JJ-01A-11R-A056-07 | 2.788915354 | 835 | High |
| TCGA-A2-A0T5-01A-21R-A084-07 | 2.802482761 | 836 | High |
| TCGA-D8-A27M-01A-11R-A16F-07 | 2.806592153 | 837 | High |
| TCGA-E9-A1NE-01A-21R-A14M-07 | 2.806818964 | 838 | High |
| TCGA-D8-A1Y0-01A-11R-A14M-07 | 2.820995833 | 839 | High |
| TCGA-A7-A0CG-01A-12R-A056-07 | 2.823117533 | 840 | High |
| TCGA-E2-A1IO-01A-11R-A144-07 | 2.833173463 | 841 | High |
| TCGA-AO-A12B-01A-11R-A10J-07 | 2.836893627 | 842 | High |
| TCGA-3C-AALK-01A-11R-A41B-07 | 2.838770284 | 843 | High |
| TCGA-C8-A1HJ-01A-11R-A13Q-07 | 2.839032419 | 844 | High |
| TCGA-A7-A3J1-01A-11R-A213-07 | 2.84641308 | 845 | High |
| TCGA-S3-AA14-01A-11R-A41B-07 | 2.850479411 | 846 | High |
| TCGA-AR-A1AK-01A-21R-A12P-07 | 2.856368255 | 847 | High |
| TCGA-D8-A142-01A-11R-A115-07 | 2.85939315 | 848 | High |
| TCGA-AR-A2LM-01A-11R-A180-07 | 2.862768759 | 849 | High |
| TCGA-E2-A108-01A-13R-A10J-07 | 2.871311409 | 850 | High |
| TCGA-BH-A0BA-01A-11R-A056-07 | 2.876782133 | 851 | High |
| TCGA-E9-A3X8-01A-31R-A22U-07 | 2.880685525 | 852 | High |
| TCGA-LD-A7W6-01A-81R-A352-07 | 2.892255008 | 853 | High |
| TCGA-A2-A1FZ-01A-51R-A14D-07 | 2.892527031 | 854 | High |
| TCGA-BH-A0B7-01A-12R-A115-07 | 2.895651618 | 855 | High |
| TCGA-AO-A03R-01A-21R-A034-07 | 2.902363054 | 856 | High |
| TCGA-EW-A2FV-01A-11R-A17B-07 | 2.919797706 | 857 | High |
| TCGA-A7-A0CE-01A-11R-A00Z-07 | 2.921112564 | 858 | High |
| TCGA-D8-A1XG-01A-11R-A14D-07 | 2.922159782 | 859 | High |
| TCGA-E9-A5FL-01A-11R-A27Q-07 | 2.928806145 | 860 | High |
| TCGA-LD-A7W5-01A-22R-A352-07 | 2.932363591 | 861 | High |
| TCGA-AC-A6IV-01A-12R-A33J-07 | 2.933327133 | 862 | High |
| TCGA-E2-A1LI-01A-12R-A157-07 | 2.935271148 | 863 | High |
| TCGA-A1-A0SP-01A-11R-A084-07 | 2.94069298 | 864 | High |
| TCGA-D8-A1X8-01A-11R-A14M-07 | 2.941650976 | 865 | High |
| TCGA-BH-A1EU-01A-11R-A137-07 | 2.950356428 | 866 | High |
| TCGA-BH-A0DO-01B-11R-A12D-07 | 2.957821758 | 867 | High |
| TCGA-A2-A4RW-01A-21R-A266-07 | 2.963215155 | 868 | High |
| TCGA-XX-A89A-01A-11R-A36F-07 | 2.966172029 | 869 | High |
| TCGA-JL-A3YX-01A-11R-A22U-07 | 2.972380189 | 870 | High |
| TCGA-BH-A0C3-01A-21R-A12P-07 | 2.980445796 | 871 | High |
| TCGA-AC-A23G-01A-11R-A213-07 | 2.986665774 | 872 | High |
| TCGA-BH-A0H5-01A-21R-A115-07 | 2.988339307 | 873 | High |
| TCGA-AO-A12E-01A-11R-A10J-07 | 2.997491137 | 874 | High |
| TCGA-AR-A2LH-01A-31R-A18M-07 | 3.005004675 | 875 | High |
| TCGA-A2-A0EX-01A-21R-A034-07 | 3.014373148 | 876 | High |
| TCGA-AC-A3W6-01A-12R-A22K-07 | 3.017886285 | 877 | High |
| TCGA-A2-A0SY-01A-31R-A084-07 | 3.019292707 | 878 | High |
| TCGA-OL-A5DA-01A-11R-A27Q-07 | 3.023006896 | 879 | High |
| TCGA-AC-A6IX-01A-12R-A32P-07 | 3.024833823 | 880 | High |
| TCGA-GM-A2DF-01A-11R-A180-07 | 3.030141822 | 881 | High |
| TCGA-AC-A3BB-01A-21R-A19W-07 | 3.030547964 | 882 | High |
| TCGA-AR-A2LJ-01A-12R-A19W-07 | 3.044848715 | 883 | High |
| TCGA-A1-A0SO-01A-22R-A084-07 | 3.048881347 | 884 | High |
| TCGA-OL-A97C-01A-32R-A41B-07 | 3.060358723 | 885 | High |
| TCGA-BH-A0H3-01A-11R-A12P-07 | 3.064537998 | 886 | High |
| TCGA-AN-A0XL-01A-11R-A10J-07 | 3.06474492 | 887 | High |
| TCGA-GM-A3XN-01A-12R-A22U-07 | 3.072637493 | 888 | High |
| TCGA-A2-A0CZ-01A-11R-A034-07 | 3.074505436 | 889 | High |
| TCGA-Z7-A8R5-01A-42R-A41B-07 | 3.077208811 | 890 | High |
| TCGA-D8-A1JL-01A-11R-A13Q-07 | 3.0824301 | 891 | High |
| TCGA-BH-A18T-01A-11R-A12D-07 | 3.089582893 | 892 | High |
| TCGA-BH-A8FZ-01A-11R-A352-07 | 3.113933818 | 893 | High |
| TCGA-B6-A401-01A-11R-A239-07 | 3.118143737 | 894 | High |
| TCGA-AC-A3W7-01A-11R-A22K-07 | 3.119555385 | 895 | High |
| TCGA-A2-A1G0-01A-11R-A13Q-07 | 3.131721171 | 896 | High |
| TCGA-A2-A0CK-01A-11R-A22K-07 | 3.132609743 | 897 | High |
| TCGA-BH-A0DX-01A-11R-A115-07 | 3.144780586 | 898 | High |
| TCGA-GM-A4E0-01A-12R-A266-07 | 3.151696488 | 899 | High |
| TCGA-EW-A1PG-01A-11R-A144-07 | 3.174246607 | 900 | High |
| TCGA-AC-A8OS-01A-12R-A41B-07 | 3.183311824 | 901 | High |
| TCGA-OK-A5Q2-01A-11R-A27Q-07 | 3.185707999 | 902 | High |
| TCGA-B6-A0X7-01A-11R-A10J-07 | 3.187926067 | 903 | High |
| TCGA-A2-A0CV-01A-31R-A115-07 | 3.201696586 | 904 | High |
| TCGA-AN-A0XV-01A-11R-A109-07 | 3.20614307 | 905 | High |
| TCGA-A8-A091-01A-11R-A00Z-07 | 3.235834262 | 906 | High |
| TCGA-XX-A899-01A-11R-A36F-07 | 3.236676292 | 907 | High |
| TCGA-BH-A1FB-01A-11R-A13Q-07 | 3.262297842 | 908 | High |
| TCGA-A7-A4SE-01A-11R-A266-07 | 3.264371285 | 909 | High |
| TCGA-BH-A1F0-01A-11R-A137-07 | 3.275498792 | 910 | High |
| TCGA-GM-A5PV-01A-11R-A28M-07 | 3.275528589 | 911 | High |
| TCGA-A2-A0CP-01A-11R-A034-07 | 3.283581055 | 912 | High |
| TCGA-OL-A5RX-01A-11R-A28M-07 | 3.285017449 | 913 | High |
| TCGA-AC-A2FG-01A-11R-A17B-07 | 3.287826867 | 914 | High |
| TCGA-AR-A5QP-01A-11R-A28M-07 | 3.288048431 | 915 | High |
| TCGA-BH-A0HP-01A-12R-A084-07 | 3.295884621 | 916 | High |
| TCGA-E2-A3DX-01A-21R-A213-07 | 3.29795439 | 917 | High |
| TCGA-AO-A03T-01A-21R-A034-07 | 3.303123168 | 918 | High |
| TCGA-D8-A145-01A-11R-A115-07 | 3.319299998 | 919 | High |
| TCGA-AC-A2FB-01A-11R-A17B-07 | 3.324968974 | 920 | High |
| TCGA-AR-A24W-01A-11R-A169-07 | 3.348345745 | 921 | High |
| TCGA-D8-A1JB-01A-11R-A13Q-07 | 3.34905383 | 922 | High |
| TCGA-AC-A2FE-01A-11R-A19W-07 | 3.350072863 | 923 | High |
| TCGA-BH-A0BQ-01A-21R-A115-07 | 3.350129454 | 924 | High |
| TCGA-A2-A1G1-01A-21R-A13Q-07 | 3.352108771 | 925 | High |
| TCGA-E9-A1NC-01A-21R-A26B-07 | 3.36441835 | 926 | High |
| TCGA-AR-A2LN-01A-21R-A18M-07 | 3.370708328 | 927 | High |
| TCGA-OL-A66I-01A-21R-A29R-07 | 3.375373125 | 928 | High |
| TCGA-BH-A0EA-01A-11R-A115-07 | 3.389704467 | 929 | High |
| TCGA-D8-A1XQ-01A-11R-A14M-07 | 3.412591863 | 930 | High |
| TCGA-AQ-A7U7-01A-22R-A352-07 | 3.425687561 | 931 | High |
| TCGA-GM-A3NW-01A-21R-A22K-07 | 3.43412111 | 932 | High |
| TCGA-BH-A0BL-01A-11R-A115-07 | 3.43865215 | 933 | High |
| TCGA-AR-A2LO-01A-31R-A18M-07 | 3.451870479 | 934 | High |
| TCGA-B6-A0IQ-01A-11R-A034-07 | 3.455596827 | 935 | High |
| TCGA-D8-A27V-01A-12R-A17B-07 | 3.460611523 | 936 | High |
| TCGA-AN-A0FS-01A-11R-A034-07 | 3.496679645 | 937 | High |
| TCGA-A2-A4S2-01A-12R-A266-07 | 3.500049922 | 938 | High |
| TCGA-D8-A3Z5-01A-41R-A24H-07 | 3.505027086 | 939 | High |
| TCGA-AR-A1AM-01A-41R-A22K-07 | 3.524502537 | 940 | High |
| TCGA-A2-A0YK-01A-22R-A109-07 | 3.534746421 | 941 | High |
| TCGA-W8-A86G-01A-21R-A36F-07 | 3.536699481 | 942 | High |
| TCGA-AO-A12A-01A-21R-A115-07 | 3.537333346 | 943 | High |
| TCGA-AC-A3QP-01A-11R-A22U-07 | 3.558292128 | 944 | High |
| TCGA-A2-A0EW-01A-21R-A115-07 | 3.569041395 | 945 | High |
| TCGA-A7-A13H-01A-11R-A22K-07 | 3.573398765 | 946 | High |
| TCGA-B6-A0I6-01A-11R-A034-07 | 3.600590898 | 947 | High |
| TCGA-A8-A08H-01A-21R-A00Z-07 | 3.607981229 | 948 | High |
| TCGA-B6-A3ZX-01A-11R-A239-07 | 3.621911205 | 949 | High |
| TCGA-C8-A8HR-01A-11R-A36F-07 | 3.634140177 | 950 | High |
| TCGA-AR-A1AU-01A-11R-A12P-07 | 3.643810023 | 951 | High |
| TCGA-AR-A5QN-01A-12R-A28M-07 | 3.646058945 | 952 | High |
| TCGA-E2-A14Q-01A-11R-A12D-07 | 3.681797809 | 953 | High |
| TCGA-D8-A1JU-01A-11R-A13Q-07 | 3.685704724 | 954 | High |
| TCGA-LL-A6FR-01A-12R-A31O-07 | 3.693007373 | 955 | High |
| TCGA-AR-A1AL-01A-21R-A12P-07 | 3.69841853 | 956 | High |
| TCGA-EW-A424-01A-11R-A24H-07 | 3.700473011 | 957 | High |
| TCGA-C8-A12V-01A-11R-A115-07 | 3.704628544 | 958 | High |
| TCGA-AO-A1KR-01A-12R-A144-07 | 3.709897146 | 959 | High |
| TCGA-E2-A14N-01A-31R-A137-07 | 3.713046896 | 960 | High |
| TCGA-A7-A4SC-01A-12R-A266-07 | 3.72267367 | 961 | High |
| TCGA-A2-A0T7-01A-21R-A084-07 | 3.724497482 | 962 | High |
| TCGA-OL-A66N-01A-12R-A31O-07 | 3.72707092 | 963 | High |
| TCGA-A8-A07R-01A-21R-A034-07 | 3.728704197 | 964 | High |
| TCGA-AR-A256-01A-11R-A169-07 | 3.728921827 | 965 | High |
| TCGA-OL-A5RY-01A-21R-A28M-07 | 3.732931233 | 966 | High |
| TCGA-OL-A6VR-01A-32R-A33J-07 | 3.738346314 | 967 | High |
| TCGA-BH-A0DT-01A-21R-A12D-07 | 3.741164933 | 968 | High |
| TCGA-AC-A3TM-01A-11R-A22K-07 | 3.777577522 | 969 | High |
| TCGA-A7-A26H-01A-11R-A169-07 | 3.783624278 | 970 | High |
| TCGA-A2-A3KC-01A-11R-A213-07 | 3.78966666 | 971 | High |
| TCGA-B6-A0RE-01A-11R-A056-07 | 3.794821324 | 972 | High |
| TCGA-AC-A2FF-01A-11R-A17B-07 | 3.806746801 | 973 | High |
| TCGA-B6-A402-01A-11R-A239-07 | 3.807354922 | 974 | High |
| TCGA-LD-A66U-01A-11R-A31O-07 | 3.829362492 | 975 | High |
| TCGA-OL-A5S0-01A-11R-A28M-07 | 3.834347159 | 976 | High |
| TCGA-A7-A13E-01A-11R-A277-07 | 3.840271869 | 977 | High |
| TCGA-BH-A0W4-01A-11R-A109-07 | 3.856428019 | 978 | High |
| TCGA-E2-A1LH-01A-11R-A14D-07 | 3.876006081 | 979 | High |
| TCGA-BH-A0B3-01A-11R-A056-07 | 3.894449162 | 980 | High |
| TCGA-D8-A27I-01A-11R-A16F-07 | 3.923443966 | 981 | High |
| TCGA-A2-A0D2-01A-21R-A034-07 | 3.926018401 | 982 | High |
| TCGA-AR-A251-01A-12R-A169-07 | 3.930765718 | 983 | High |
| TCGA-EW-A1J2-01A-21R-A13Q-07 | 3.948871795 | 984 | High |
| TCGA-OL-A66L-01A-12R-A31O-07 | 3.972444526 | 985 | High |
| TCGA-A2-A0YM-01A-11R-A109-07 | 3.990973007 | 986 | High |
| TCGA-E9-A244-01A-11R-A169-07 | 3.99394603 | 987 | High |
| TCGA-A2-A25A-01A-12R-A16F-07 | 3.995330744 | 988 | High |
| TCGA-BH-A0DI-01A-21R-A12P-07 | 3.999305536 | 989 | High |
| TCGA-A2-A0CO-01A-13R-A22K-07 | 4.030124161 | 990 | High |
| TCGA-A7-A425-01A-11R-A24H-07 | 4.054700901 | 991 | High |
| TCGA-A1-A0SF-01A-11R-A144-07 | 4.059597553 | 992 | High |
| TCGA-D8-A27H-01A-11R-A16F-07 | 4.086911117 | 993 | High |
| TCGA-A7-A5ZW-01A-12R-A29R-07 | 4.119397681 | 994 | High |
| TCGA-BH-A0DV-01A-21R-A12P-07 | 4.143368927 | 995 | High |
| TCGA-E2-A1L6-01A-11R-A13Q-07 | 4.175005553 | 996 | High |
| TCGA-D8-A4Z1-01A-21R-A266-07 | 4.193913641 | 997 | High |
| TCGA-A2-A0T2-01A-11R-A084-07 | 4.197480139 | 998 | High |
| TCGA-BH-A18Q-01A-12R-A12D-07 | 4.222951357 | 999 | High |
| TCGA-BH-A8G0-01A-11R-A352-07 | 4.228241497 | 1000 | High |
| TCGA-LD-A74U-01A-13R-A33J-07 | 4.233297551 | 1001 | High |
| TCGA-AO-A129-01A-21R-A10J-07 | 4.26713874 | 1002 | High |
| TCGA-BH-A42V-01A-11R-A24H-07 | 4.284736206 | 1003 | High |
| TCGA-EW-A1P7-01A-21R-A144-07 | 4.289052425 | 1004 | High |
| TCGA-AQ-A04J-01A-02R-A034-07 | 4.301719327 | 1005 | High |
| TCGA-B6-A0IH-01A-11R-A115-07 | 4.311335993 | 1006 | High |
| TCGA-OL-A5D7-01A-11R-A27Q-07 | 4.316413688 | 1007 | High |
| TCGA-BH-A1FH-01A-12R-A13Q-07 | 4.322303147 | 1008 | High |
| TCGA-A2-A3XX-01A-21R-A239-07 | 4.333853043 | 1009 | High |
| TCGA-AC-A2FO-01A-11R-A180-07 | 4.33945098 | 1010 | High |
| TCGA-B6-A0WY-01A-11R-A109-07 | 4.346176843 | 1011 | High |
| TCGA-BH-A18G-01A-11R-A12D-07 | 4.377679059 | 1012 | High |
| TCGA-A7-A13E-01A-11R-A12P-07 | 4.400264666 | 1013 | High |
| TCGA-AC-A3YI-01A-21R-A239-07 | 4.403622216 | 1014 | High |
| TCGA-AR-A2LQ-01A-22R-A18M-07 | 4.407821789 | 1015 | High |
| TCGA-D8-A1XK-01A-21R-A14M-07 | 4.422179168 | 1016 | High |
| TCGA-PL-A8LY-01A-11R-A41B-07 | 4.428926251 | 1017 | High |
| TCGA-AO-A0J4-01A-11R-A034-07 | 4.439004707 | 1018 | High |
| TCGA-B6-A0RQ-01A-11R-A115-07 | 4.442379574 | 1019 | High |
| TCGA-OL-A6VQ-01A-12R-A41B-07 | 4.444693567 | 1020 | High |
| TCGA-A8-A07C-01A-11R-A034-07 | 4.521364878 | 1021 | High |
| TCGA-HN-A2NL-01A-11R-A18M-07 | 4.529464913 | 1022 | High |
| TCGA-AC-A6IW-01A-12R-A33J-07 | 4.543273152 | 1023 | High |
| TCGA-EW-A3U0-01A-11R-A22K-07 | 4.575088405 | 1024 | High |
| TCGA-BH-A0WA-01A-11R-A109-07 | 4.603774686 | 1025 | High |
| TCGA-BH-A0E6-01A-11R-A034-07 | 4.638212885 | 1026 | High |
| TCGA-EW-A1PH-01A-11R-A14M-07 | 4.638404055 | 1027 | High |
| TCGA-BH-A0BO-01A-23R-A12D-07 | 4.675420773 | 1028 | High |
| TCGA-BH-A0E9-01B-11R-A115-07 | 4.704479153 | 1029 | High |
| TCGA-A8-A08R-01A-11R-A034-07 | 4.716244669 | 1030 | High |
| TCGA-EW-A1P1-01A-31R-A14D-07 | 4.720059527 | 1031 | High |
| TCGA-AO-A1KO-01A-31R-A13Q-07 | 4.740852025 | 1032 | High |
| TCGA-BH-A42U-01A-12R-A24H-07 | 4.743197183 | 1033 | High |
| TCGA-AO-A124-01A-11R-A10J-07 | 4.786225186 | 1034 | High |
| TCGA-LL-A5YP-01A-21R-A28M-07 | 4.792558664 | 1035 | High |
| TCGA-AN-A0AT-01A-11R-A034-07 | 4.79998353 | 1036 | High |
| TCGA-D8-A147-01A-11R-A115-07 | 4.803278708 | 1037 | High |
| TCGA-E2-A15E-01A-11R-A12D-07 | 4.840599188 | 1038 | High |
| TCGA-A2-A4RY-01A-31R-A266-07 | 4.852832841 | 1039 | High |
| TCGA-A2-A3XT-01A-11R-A22U-07 | 4.872508672 | 1040 | High |
| TCGA-AC-A2FK-01A-12R-A180-07 | 4.922654566 | 1041 | High |
| TCGA-LL-A73Y-01A-11R-A33J-07 | 4.975896105 | 1042 | High |
| TCGA-AC-A7VC-01A-11R-A352-07 | 5.008141735 | 1043 | High |
| TCGA-OL-A5RV-01A-12R-A28M-07 | 5.029819476 | 1044 | High |
| TCGA-AR-A1AH-01A-11R-A12D-07 | 5.04117726 | 1045 | High |
| TCGA-AR-A1AQ-01A-11R-A12P-07 | 5.070810053 | 1046 | High |
| TCGA-AN-A0FX-01A-11R-A034-07 | 5.089900632 | 1047 | High |
| TCGA-A2-A0ES-01A-11R-A115-07 | 5.096725695 | 1048 | High |
| TCGA-E9-A22G-01A-11R-A157-07 | 5.107076854 | 1049 | High |
| TCGA-AR-A24Q-01A-12R-A169-07 | 5.113892157 | 1050 | High |
| TCGA-A2-A1G6-01A-11R-A13Q-07 | 5.179558821 | 1051 | High |
| TCGA-B6-A400-01A-11R-A239-07 | 5.18992337 | 1052 | High |
| TCGA-OL-A6VO-01A-12R-A33J-07 | 5.223831822 | 1053 | High |
| TCGA-A7-A426-01A-22R-A24H-07 | 5.241432176 | 1054 | High |
| TCGA-A2-A4RX-01A-11R-A266-07 | 5.257610359 | 1055 | High |
| TCGA-A2-A04T-01A-21R-A034-07 | 5.271545198 | 1056 | High |
| TCGA-BH-A1F6-01A-11R-A13Q-07 | 5.275353521 | 1057 | High |
| TCGA-BH-A0AV-01A-31R-A115-07 | 5.27735994 | 1058 | High |
| TCGA-D8-A27F-01A-11R-A16F-07 | 5.299072444 | 1059 | High |
| TCGA-A2-A04U-01A-11R-A115-07 | 5.301115694 | 1060 | High |
| TCGA-AQ-A54N-01A-11R-A266-07 | 5.316062452 | 1061 | High |
| TCGA-E2-A573-01A-11R-A29R-07 | 5.319581809 | 1062 | High |
| TCGA-E2-A14R-01A-11R-A115-07 | 5.353700855 | 1063 | High |
| TCGA-S3-AA10-01A-21R-A41B-07 | 5.452684381 | 1064 | High |
| TCGA-A7-A0DA-01A-31R-A115-07 | 5.461050463 | 1065 | High |
| TCGA-HN-A2OB-01A-21R-A27Q-07 | 5.50345687 | 1066 | High |
| TCGA-EW-A1OW-01A-21R-A144-07 | 5.59749244 | 1067 | High |
| TCGA-A2-A0YL-01A-21R-A109-07 | 5.628024062 | 1068 | High |
| TCGA-AR-A0TS-01A-11R-A115-07 | 5.670333333 | 1069 | High |
| TCGA-A2-A0YJ-01A-11R-A109-07 | 5.694743715 | 1070 | High |
| TCGA-LL-A440-01A-11R-A24H-07 | 5.774528801 | 1071 | High |
| TCGA-AR-A0TU-01A-31R-A109-07 | 5.905225727 | 1072 | High |
| TCGA-B6-A0RU-01A-11R-A084-07 | 5.948196668 | 1073 | High |
| TCGA-AR-A2LR-01A-12R-A18M-07 | 5.95044975 | 1074 | High |
| TCGA-A2-A3XY-01A-11R-A239-07 | 6.013102658 | 1075 | High |
| TCGA-E2-A14U-01A-11R-A22K-07 | 6.142025345 | 1076 | High |
| TCGA-A2-A3XS-01A-11R-A22U-07 | 6.156813216 | 1077 | High |
| TCGA-A7-A5ZX-01A-12R-A29R-07 | 6.190774844 | 1078 | High |
| TCGA-BH-A28O-01A-11R-A22K-07 | 6.238093236 | 1079 | High |
| TCGA-AR-A0TP-01A-11R-A084-07 | 6.292652931 | 1080 | High |
| TCGA-E9-A1N8-01A-11R-A144-07 | 6.342108456 | 1081 | High |
| TCGA-AO-A12F-01A-11R-A115-07 | 6.42736481 | 1082 | High |
| TCGA-C8-A134-01A-11R-A115-07 | 6.44178058 | 1083 | High |
| TCGA-GM-A3XG-01A-31R-A24H-07 | 6.463503121 | 1084 | High |
| TCGA-E2-A1LK-01A-21R-A14D-07 | 6.591182717 | 1085 | High |
| TCGA-A1-A0SB-01A-11R-A144-07 | 6.889791105 | 1086 | High |
| TCGA-E2-A158-01A-11R-A12D-07 | 7.068733143 | 1087 | High |
