# Supplementary material for: Identification and validation of DNA methylation-driven gene OSR1 as a novel tumor suppressor for the diagnosis and prognosis of breast cancer
Source: Front Genet. 2025 Jul 7;16:1583620. doi: 10.3389/fgene.2025.1583620 (PMC12277919; doi:10.3389/fgene.2025.1583620)
Supplement: Supplementary file 6 [file Table6.docx]

**Supplementary Table** KEGG pathway functional enrichment for *OSR1*-related DEGs
